# Genomic DNA Sequences from Mastodon and Woolly Mammoth Reveal Deep Speciation of Forest and Savanna Elephants

**DOI:** 10.1371/journal.pbio.1000564

**Published:** 2010-12-21

**Authors:** Nadin Rohland, David Reich, Swapan Mallick, Matthias Meyer, Richard E. Green, Nicholas J. Georgiadis, Alfred L. Roca, Michael Hofreiter

**Affiliations:** 1 Department of Genetics, Harvard Medical School, Boston, Massachusetts, United States of America; 2 Broad Institute of MIT and Harvard, Cambridge, Massachusetts, United States of America; 3 Max Planck Institute for Evolutionary Anthropology, Leipzig, Germany; 4 Department of Biomolecular Engineering, University of California, Santa Cruz, United States of America; 5 Director of Conservation, Ishawooa Mesa Ranch, Cody, Wyoming, United States of America; 6 Department of Animal Sciences and Institute for Genomic Biology, University of Illinois at Urbana-Champaign, Urbana, Illinois, United States of America; 7 Department of Biology, University of York, York, United Kingdom; Massey University, New Zealand

## Abstract

To elucidate the history of living and extinct elephantids, we generated 39,763 bp of aligned nuclear DNA sequence across 375 loci for African savanna elephant, African forest elephant, Asian elephant, the extinct American mastodon, and the woolly mammoth. Our data establish that the Asian elephant is the closest living relative of the extinct mammoth in the nuclear genome, extending previous findings from mitochondrial DNA analyses. We also find that savanna and forest elephants, which some have argued are the same species, are as or more divergent in the nuclear genome as mammoths and Asian elephants, which are considered to be distinct genera, thus resolving a long-standing debate about the appropriate taxonomic classification of the African elephants. Finally, we document a much larger effective population size in forest elephants compared with the other elephantid taxa, likely reflecting species differences in ancient geographic structure and range and differences in life history traits such as variance in male reproductive success.

## Abstract 2

This study compares three extant elephants species - forest, savanna, and Asian - to extinct mammoth and mastodon. Surprisingly, forest and savanna elephants in Africa today are as distinct from each other as mammoth and Asian elephants.

## Author Summary

The living elephants are the last survivors of a once highly successful mammalian order, the Proboscidea, which includes extinct species such as the iconic woolly mammoth (*Mammuthus primigenius*) and the American mastodon (*Mammut americanum*). Despite numerous studies, the phylogenetic relationships of the modern elephants to the woolly mammoth, as well as the taxonomic status of the African elephants of the genus *Loxodonta*, remain controversial. This is in large part due to the fact that both the woolly mammoth and the American mastodon (the closest outgroup to elephants and mammoths available for genetic studies) are extinct, posing considerable technical hurdles for comparative genetic analysis. We have used a combination of modern DNA sequencing and targeted PCR amplification to obtain a large data set for comparing American mastodon, woolly mammoth, Asian elephant, African savanna elephant, and African forest elephant. We unequivocally establish that the Asian elephant is the sister species to the woolly mammoth. A surprising finding from our study is that the divergence of African savanna and forest elephants—which some have argued to be two populations of the same species—is about as ancient as the divergence of Asian elephants and mammoths. Given their ancient divergence, we conclude that African savanna and forest elephants should be classified as two distinct species.

## Introduction

The technology for sequencing DNA from extinct species such as mastodons (genus *Mammut*) and mammoths (genus *Mammuthus*) provides a powerful tool for elucidating the phylogeny of the Elephantidae, a family that originated in the Miocene and that includes Asian elephants (genus *Elephas*), African elephants (genus *Loxodonta*), and extinct mammoths [1]–[8]. In the highest resolution study to date, complete mitochondrial DNA (mtDNA) genomes from three elephantid genera were compared to the mastodon outgroup. The mtDNA analysis suggested that mammoths and Asian elephants form a clade with an estimated genetic divergence time of 5.8–7.8 million years ago (Mya), while African elephants diverged from an earlier common ancestor 6.6–8.8 Mya [8]. However, mtDNA represents just a single locus in the genome and need not represent the true species phylogeny since a single gene tree can differ from the consensus species tree of the taxa in question [9]–[11]. Generalizing about species relationships based on mtDNA alone is especially problematic for the Elephantidae because their core social groups (“herds”) are matrilocal, with females rarely, if ever, dispersing across groups [12]. This results in mtDNA genealogies in both African [13],[14] and Asian elephants [15] that exhibit deeper divergence and/or different phylogeographic patterns than the nuclear genome.

These observed discrepancies between the phylogeographic patterns of nuclear and mtDNA sequences have led to a debate about the appropriate taxonomic status of African elephants. Most researchers have argued, based on morphology and nuclear DNA markers, that forest (*Loxodonta cyclotis*) and savanna (*Loxodonta africana*) elephants should be considered separate species [13],[16]–[19]. However, this notion has been contested [20] based on mtDNA patterns, which reveal some haplogroups with coalescent times of less than half a million years [21] that are shared across forest and savanna elephants, indicating relatively recent gene flow among the ancestors of these taxa. Taxonomies for African elephants based on mtDNA phylogeographic patterns have suggested anywhere from one to four species [20],[22],[23], whereas analysis of morphology and nuclear data sets has suggested two species [13],[16]–[19].

The study of large amounts of nuclear DNA sequences has the potential to resolve elephantid phylogeny, but due to technical challenges associated with obtaining homologous data sets from fossil DNA, no sufficiently large nuclear DNA data set has been published to date. Although a draft genome is available for woolly mammoth (*Mammuthus primigenius*) [5] and savanna elephant (loxAfr; http://www.broadinstitute.org/ftp/pub/assemblies/mammals/elephant/), comparative sequence data are lacking for Asian (*Elephas maximus*) and forest elephant, as well as for a suitable outgroup like the American mastodon (*Mammut americanum*). Using a combination of next generation sequencing and targeted multiplex PCR, we obtained the first substantial nuclear data set for comparing these species.

## Results

### Data Set

**Figure 1:**
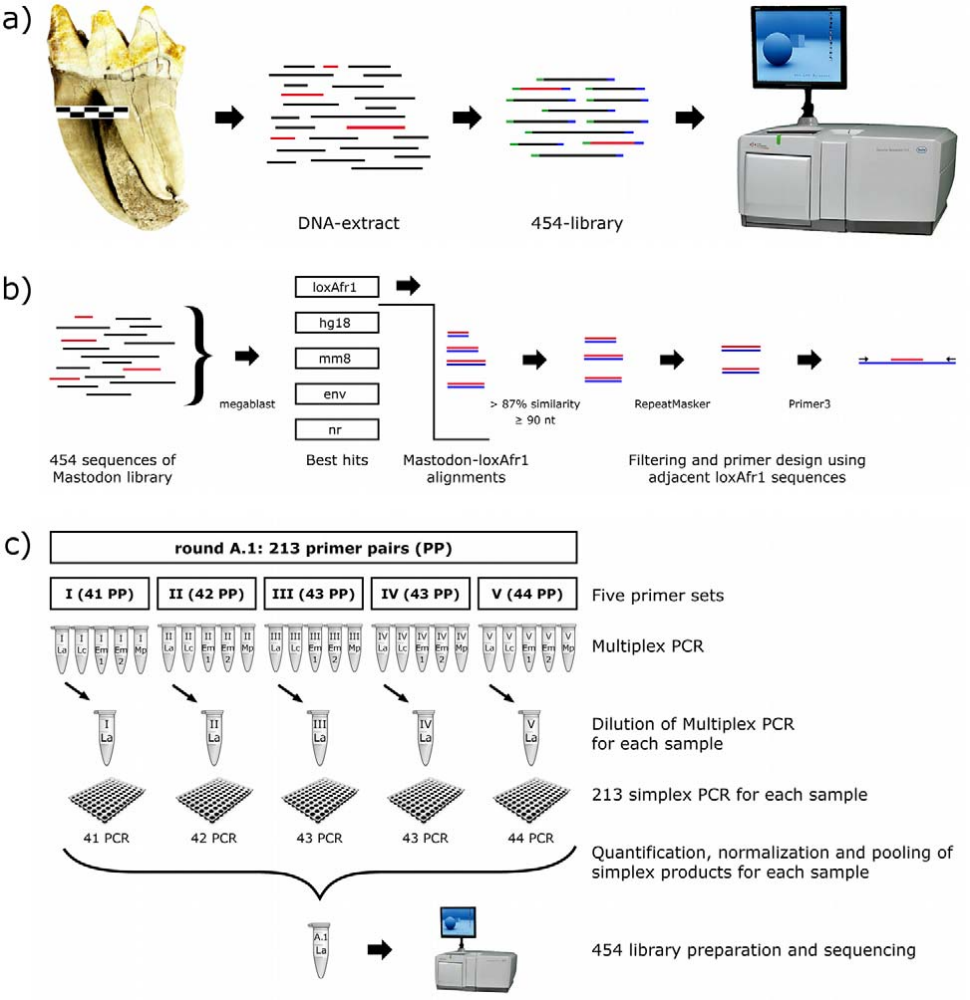
Strategy for obtaining overlapping DNA from four elephantids and a mastodon. (a) *Mastodon shotgun 454 sequencing.* We ligated 454-adaptors (green and blue) to the ends of the DNA molecules and sequenced the libraries on a Roche 454 GS. (b) *Bioinformatic analysis of shotgun 454 sequences.* To identify proboscidean sequence, we compared the sequences to databases consisting of the savanna elephant draft genome (loxAfr1), the human genome (hg18), the mouse genome (mm8), NCBI's nucleotide database of environmental samples (env), and NCBI's non-redundant nucleotide database (nr). The 454 sequences with a best match to loxAfr1 (in red) were aligned to loxAfr1. Alignments of at least 90 bp in length and with a similarity higher than 87% were used for primer design after filtering out known repeat elements (using the UCSC RepeatMasker database). Primers were based on loxAfr1 sequence flanking the mastodon sequence. (c) *Multiplex PCR and sequencing of the targeted loci in modern elephants and mammoth.* We show the protocol for the first of four rounds of the project (Table S3 provides details of the further rounds). A total of 213 primer pairs were randomly divided into 5 multiplex primer mixes with 41–44 primer pairs per mix. These mixes were used for the first step of the two-step multiplex PCR approach, for each of the 5 samples (La, *Loxodonta africana*; Lc, *L. cyclotis*; Em 1, *Elephas maximus* 1; Em 2, *E. maximus* 2; Mp, *Mammuthus primigenius*). Dilutions of these products were used as templates to amplify the loci individually in the second step (shown for *L. africana*), resulting in 213 distinct products per sample. These products were quantified, normalized, and merged into one pool per sample. A 454 library was prepared and sequenced on 1/16^th^ of a picotiter plate of a Roche 454 GS.

We carried out shotgun sequencing of DNA from an American mastodon with a Roche 454 Genome Sequencer (GS), using the same DNA extract from a 50,000–130,000-yr-old tooth that we previously used to generate a complete mtDNA genome sequence from the mastodon [8]. After comparing the 45 Mb of shotgun DNA data that we obtained to the Genbank database, and only retaining reads for which the best match was to sequences of the savanna elephant draft sequence (loxAfr1), we were left with 1.76 Mb of mastodon sequence (Figure 1 and Figure S1).

To amplify the same set of loci across all species, we designed PCR primers flanking the regions of mastodon-elephant alignment, using the loxAfr1 savanna elephant sequence as a template (Figure 1) (a full list of the primers is presented in Dataset S1). We used these primers in a multiplexed protocol [24] to amplify one or two Asian elephants, one African forest elephant, one woolly mammoth, and one African savanna elephant unrelated to the individual used for the reference sequence (Figure 1 and Table S1). We then sequenced the products on a Roche 454 GS to a median coverage of 41-fold and assembled a consensus sequence for each individual by restricting to nucleotides with at least 3-fold coverage. After four rounds of amplification and sequencing, we obtained 39,763 base pairs across 375 loci with data from all five taxa (Text S1; Figure S2; Table S2, Table S3). We identified 1,797 nucleotides in this data set in which two different alleles were observed and used these sites for the majority of our analyses (the genotypes are provided in Dataset S2). A total of 549 of these biallelic sites were polymorphic among the elephantids, while the remaining sites were fixed differences compared to the mastodon sequence.

To assess the utility of the data for molecular dating and inference about demographic history, we carried out a series of relative rate tests, searching for an excess of divergent sites in one taxon compared to another since their split, which could reflect sequencing errors or changes in the molecular clock [25]. None of the pairs of taxa showed a significant excess of divergent sites compared with any other (Table 1). When we compared the data within taxa, we found that the savanna reference genome loxAfr1 had a significantly higher number of lineage-specific substitutions than the savanna elephant we sequenced (nominal P = 0.03 from a two-sided test without correcting for multiple hypothesis testing). This is consistent with our data being of higher quality than the loxAfr1 reference sequence, presumably due to our high read coverage.

**Table 1:** Genetic divergence and heterozygosity estimates for the elephantids.

First Taxon	Second Taxon	Genetic Divergence (Heterozygosity If Within Taxa) Normalized by Savanna-Asian Genetic Divergence	±1 Standard Deviation	Rate Test for More Substitutions in One Taxon Than the Other
*Across taxa*				
Savanna	Forest	74%	6%	*p* = 0.97
Savanna	Mammoth	92%	5%	*p* = 0.86
Savanna	Asian	100%	n/a	*p* = 0.32
Forest	Mammoth	96%	7%	*p* = 0.83
Forest	Asian	103%	5%	*p* = 0.27
Mammoth	Asian	65%	5%	*p* = 0.33
*Within taxa (heterozygosity)*				
Savanna	Savanna	8%	2%	n/a
Forest	Forest	30%	4%	n/a
Mammoth	Mammoth	9%	2%	n/a
Asian	Asian	15%	3%	n/a

We calculated genetic divergences based on 549 sites that are polymorphic among the elephantids, normalizing by savanna-Asian elephant genetic divergence. Standard errors are from a Weighted Jackknife (calculated in this way, savanna-Asian genetic divergence has no uncertainty since it is used for normalization). The results show that savanna and forest elephants are sister groups (>4 standard deviations less diverged than savanna-Asian) and that Asian elephants and mammoths are also sister groups (>6 standard deviations less diverged than savanna-Asian).

In contrast to our elephantid data, our mastodon data had a high error rate, as expected given that it was derived from shotgun sequencing data providing only 1-fold coverage at each position. To better understand the effect of errors in the mastodon sequence, we PCR-amplified a subset of loci in the mastodon, obtaining high-quality mastodon data at 1,726 bases (Text S2). Of the *n* = 23 sites overlapping these bases that we knew were polymorphic among the elephantids, the mastodon allele call always agreed between the PCR and shotgun data, indicating that our mastodon data are reliable for the purpose of determining an ancestral allele (the main purpose for which we use the mastodon data). However, only 38% of mastodon-elephantid divergent sites validated, which we ascribe to mastodon-specific errors, since almost all the discrepancies were consistent with C/G-to-T/A misincorporations (the most prominent error in ancient DNA) [26]–[28], or mismapping of some of the short mastodon reads (2). Thus, our raw estimate of mastodon-elephantid divergence is too high, making it inappropriate to use mastodon for calibrating genetic divergences among the elephantids, as we previously did for mtDNA where we had high-quality mastodon data [8].

### Genetic Diversity and Phylogenetic Relationships among Elephantid Taxa

We estimated the relative genetic diversity across elephantids by counting the total number of heterozygous genotypes in each taxon, and normalizing by the total number of sites differing between (S)avanna and (A)sian elephants (*t*_SA_). Within-species genetic diversity as a fraction of savanna-Asian divergence is estimated to be similar for savanna elephants (8±2%) and mammoths (9±2%), higher for Asian elephants (15±3%), and much higher for forest elephants (30±4%) (standard errors from a Weighted Jackknife; Methods). This supports previous findings of a higher average time to the most recent common genetic ancestor in forest compared to savanna elephants (Table 1) [13],[17]. We caution that these diversity estimates are based on analyzing only a single individual from each taxon, which could produce a too-low estimate of diversity in the context of recent inbreeding. Encouragingly, however, in Asian elephants where two individuals were sequenced for some loci, genetic diversity estimates are consistent whether measured across (18±5%) or within samples (15±3%). A further potential concern is “allele specific PCR”, whereby one allele is preferentially amplified causing truly heterozygous sites to go undetected [29]. However, we do not believe that this is a concern since we preformed an experiment in which we re-amplified about 5% of our loci using different primers and obtained identical genotypes at all sites where we had overlapping data (Text S2).

We next inferred a nuclear phylogeny for the elephantids using the Neighbor Joining method (Methods and Figure S3). This analysis suggests that mammoths and Asian elephants are sister taxa, consistent with the mtDNA phylogeny [8], and that forest and savanna elephants are also sister taxa. We estimate that forest-savanna genetic divergence normalized by savanna-Asian is *t*_FS_/*t*_SA_ = 74±6%, while Asian-mammoth genetic divergence normalized by savanna-Asian *t*_AM_/*t*_SA_ = 65±5% (Table 1). These numbers are all significantly lower than savanna-mammoth (*t*_SM_/*t*_SA_ = 92±5%), forest-Asian (*t*_FA_/*t*_SA_ = 103±5%), and forest-mammoth (*t*_FM_/*t*_SA_ = 96±7%) normalized by savanna-Asian genetic divergence, which are all consistent with 100% as expected if they reflect the same comparison across sister groups (Table 1).

An intriguing observation is that the ratio of forest-savanna elephant genetic divergence to Asian-mammoth divergence *t*_FS_/*t*_AM_ is consistent with unity (90% credible interval 90%–138%), which is interesting given that forest and savanna elephants are sometimes classified as the same species, whereas Asian elephants and mammoth are classified as different genera [20],[30]. To further explore this issue, we focused on regions of the genome where the genealogical tree is inconsistent with the species phylogeny, a phenomenon known as “incomplete lineage sorting” (ILS) [8],[11],[31]. Information about the rate of ILS can be gleaned from the rate at which alleles are observed that cluster taxa that are not most closely related according to the overall phylogeny. For example, in a four-taxon alignment of (S)avanna, (F)orest, (E)urasian, and mastodon, “SE” and “FE” alleles that cluster savanna-Eurasian or forest-Eurasian, to the exclusion of the other taxa, are likely to be at loci with ILS (in what follows, we use the term “Eurasian elephants” to refer to woolly mammoths and Asian elephants, while recognizing that the range of the lineage ancestral to each species included Africa as well). Similarly, in a four-taxon alignment of (A)sian, (M)ammoth, (L)oxodonta (forest plus savanna), and mastodon, “AL” or “ML” sites reveal probable ILS events. We find a higher rate of inferred ILS in forest and savanna elephants than in Asian elephants and mammoths: (FE+SE)/(AL+ML) = 3.1 (P = 4×10^−8^ for exceeding unity; Table 2), indicating that there are more lineages where savanna and forest elephants are unrelated back to the African-Eurasian speciation than is the case for Asian elephants and mammoths (Table 2). This could reflect a history in which the savanna-forest population divergence time *T*_FS_ is older than the Asian-mammoth divergence time *T*_AM_, a larger population size ancestral to the African than to the Eurasian elephants, or a long period of gene flow between two incipient taxa. (We use upper case “*T*” to indicate population divergence time and lower case “*t*” to indicate average genetic divergence time (*t*≥*T*)).

**Table 2:** Incomplete lineage sorting: More deeply coalescing lineages between forest-savanna than Asian-mammoth.

4-taxon alignment: 1-2-3-Mastodon	1 (only)	2 (only)	3 (only)	12 (cluster)	13 (cluster)	23 (cluster)		Mastodon	Genetic Divergence of 1 & 2 (Divided by Savanna-Asian Divergence)		Rate of 13+23 Sites Suggesting ILS (Divided by Savanna-Asian Divergence)
Savanna-Forest-Eurasian-Mastodon	84.8	89.7	124.3	39.0	15.4	12.1	1,257.2		74%±6%	0.082±0.020	
Asian-Mammoth-Loxodonta-Mastodon	91.5	74.8	121.2	55.6	4.4	7.3	1,264.8		65%±5%	0.027±0.009	
*Savanna-Forest rate divided by Asian-Mammoth rate; 2-sided p value for a difference* *									*1.14 (*p = *0.23)*	*3.1 (*p = *0.0003)*	

The outgroup is the mastodon. To calculate the rate of any class of sites, we used the product of the relevant allele frequencies; for example, the expected rate of a “12” site where 1 and 2 share the derived allele to the exclusion of 3 is (f_1_)(f_2_)(1−f_3_). Values are summed over 1,775 sites. Standard errors are from a Weighted Jackknife.

*p values for the Asian-mammoth divergence being less than that of forest-savanna are based on a Weighted Jackknife. The Incomplete Lineage Sorting analysis in the last column, which is based on rare “13” and “23” divergent site classes, shows that there is a significantly higher probability of forest and savanna elephant alleles being unrelated all the way back to the time of their common ancestry with the Eurasian elephantids than is the case for Asian elephants and mammoth.

### Fitting a Model of Population History to the Data

**Figure 2:**
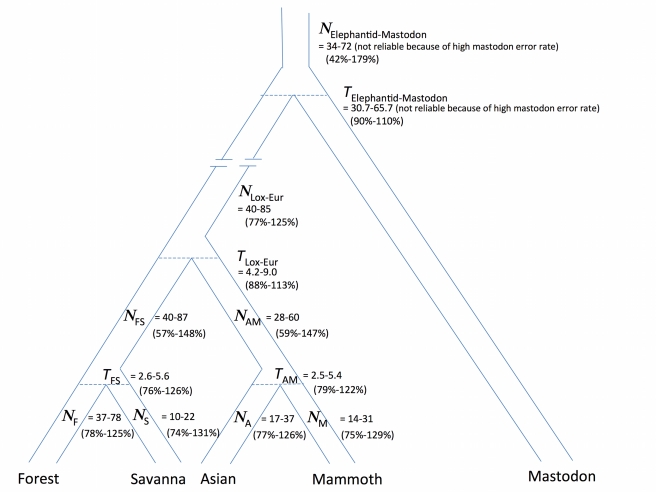
Demographic model for the history of the Elephantidae. Demographic model that is fit by MCMCcoal, in which all population splits are instantaneous (without subsequent gene flow), and all population sizes are assumed to be constant over intervals. Here, *T*_FS_ refers to forest-savanna elephant population divergence time, *T*_AM_ refers to Asian elephant-mammoth population divergence time, *T*_Lox-Eur_ refers to African-Eurasian population divergence time, and *T*_Elephantid-Mastodon_ refers to elephantid-mastodon population divergence time, presented here in millions of years. The *Ν* quantities refer to constant diploid effective population sizes ancestral to each of these splits (in thousands). For obtaining estimates of years and population sizes, we assume that the elephantids have an average of 31 years per generation, based on estimates of 17–20 years for females [53],[54] and 40–49 years for males [43],[55]. A lower or higher number of years per generation would produce a proportionate effect on the population size estimates. For each parameter, two sets of numbers are shown. The upper set shows the range consistent with the fossil record, calibrating to an assumed African-Eurasian population split of *T*_Lox-Eur_ = 4.2–9 Mya (justified in Text S5). For example for forest-savanna population divergence, this leads to *T*_FS_ = 2.6–5.6 Mya given that MCMCcoal estimates *T*_FS_ */T*_Lox-Eur_ = 62%. The lower set of numbers (in parentheses) provides MCMCcoal's 90% credible interval for the parameters as a fraction of the best estimate (e.g. 76%–126% for *T*_FS_). In the main text, we conservatively quote a range that combines the uncertainty from the fossil record and from MCMCcoal (e.g. *T*_FS_ = 1.9–7.1 Mya).

To further understand the history of the elephantids, we fit a population genetic model to the data (input file—Dataset S3) using the MCMCcoal (Markov Chain Monte Carlo coalescent) method of Yang and Rannala [32]. We fit a model in which the populations split instantaneously at times *Τ*_FS_ (forest-savanna), *Τ*_AM_ (Asian-mammoth), *Τ*_Lox-Eur_ (African-Eurasian), and *Τ*_Elephantid-Mastodon_, with constant population sizes ancestral to these speciation events of *Ν*_FS_, *Ν*_AM_, *Ν*_Lox-Eur_, and *Ν*_Elephantid-Mastodon_, and (after the final divergences) of *Ν*_F_, *Ν*_S_, *Ν*_A_, and *Ν*_M_ (Figure 2). We recognize that elephantid population sizes likely varied within these time intervals, given recurrent glacial cycles [33], changes in geographic ranges documented in the fossil record [15],[30],[34],[35], and mtDNA patterns suggesting ancient population substructure [13],[15]. Nevertheless, the constant population size assumption is useful for inferring average diversity and obtaining an initial picture of elephantid history. MCMCcoal then makes the further simplifying assumptions that our short (average 106 bp) loci experienced no recombination and that they are unlinked (the latter assumption is justified by the fact that when we mapped the loci to scaffolds from the loxAfr3 genome sequence, all but one pair were at least 100 kilobases apart; Text S3). MCMCcoal then infers the joint distribution of the “*T*” and “*N*” parameters that is consistent with the data, as well as the associated credible intervals (Table 3; Text S4).

**Table 3:** Estimates of demographic parameters from MCMCcoal.

Quantity	Populations Analyzed	Estimate (90% Credible Interval)
*Population split times*		
*τ*_FS_ (*T*_FS_ *µ*)	Forest-Savanna	0.00135 (0.00102, 0.00170)
*τ*_AM_ (*T*_AM_ *µ*)	Asian-Mammoth	0.00131 (0.00104, 0.00161)
*τ*_Lox-Eur_ (*T*_Lox-Eur_ *µ*)	African-Eurasian	0.00220 (0.00193, 0.00248)
*Effective population sizes*		
*θ*_F_ (4*N*_F_ *µg*)	Forest (current)	0.00238 (0.00185, 0.00298)
*θ*_S_ (4*N*_S_ *µg*)	Savanna (current)	0.00068 (0.00050, 0.00089)
*θ*_A_ (4*N*_A_ *µg*)	Asian (current)	0.00113 (0.00087, 0.00142)
*θ*_M_ (4*N*_M_ *µg*)	Mammoth (current)	0.00093 (0.00070, 0.00119)
*θ*_AM_ (4*N*_AM_ *µg*)	Asian-Mammoth (ancestral)	0.00181 (0.00107, 0.00265)
*θ*_FS_ (4*N*_FS_ *µg*)	Forest-Savanna (ancestral)	0.00263 (0.00152, 0.00384)
*θ*_Lox-Eur_ (4*N*_Lox-Eur_ *µg*)	African-Eurasian (ancestral)	0.00259 (0.00200, 0.00323)

All estimates are from MCMCcoal and are scaled in coalescent units; that is, a demographic parameter times a mutation rate. Abbreviations: F, forest elephant; S, savanna elephant; A, Asian elephant; M, woolly mammoth; Lox, *Loxodonta* (African forest and savanna elephant); Eur, “Eurasian” (Asian elephant and mammoth).

The MCMCcoal analysis infers that the initial divergence of forest and savanna elephant ancestors occurred at least a couple of Mya. The first line of evidence for this is that forest-savanna elephant population divergence time is estimated to be comparable to that of Asian elephants and mammoths: *Τ*_AM_/*Τ*_FS_ = 0.96 (0.69−1.36) (Table 4). Secondly, MCMCcoal infers that the ratio of forest-savanna to African-Eurasian elephant population divergence is at least 45%: *Τ*_FS_/*Τ*_Lox-Eur_ = 0.62 (0.45−0.79) (Table 4). Given that African-Eurasian genetic divergence (*T*_Lox-Eur_) can be inferred from the fossil record to have occurred 4.2–9.0 Mya (Text S5), this allows us to conclude that forest-savanna divergence occurred at least 1.9 Mya (4.2 Mya × 0.45). We caution that because MCMCcoal fits a model of instantaneous population divergence, our results do not rule out some forest-savanna gene flow having occurred more recently, as indeed must have occurred based on the mtDNA haplogroup that is shared among some forest and savanna elephants. However, such gene flow would mean that the initial population divergence must have been even older to explain the patterns we observe.

**Table 4:** Relative values of population divergence times estimated by MCMCcoal.

	*Τ*_FS_	*Τ*_AM_	*Τ*_Lox-Eur_
***Τ*** **_FS_**	1	0.96 (0.69–1.36)	1.67 (1.27–2.21)
***Τ*** **_AM_**	1.05 (0.73–1.42)	1	1.70 (1.36–2.15)
***Τ*** **_Lox-Eur_**	0.62 (0.45–0.79)	0.60 (0.46–0.74)	1

All quantities are expressed as a column-to-row ratio with a 90% credible interval. For example, the ratio of forest-savanna population split time *T*_FS_ in years to African-Eurasian split time *T*_Lox-Eur_ is *Τ*_FS_/*Τ*_Lox-Eur_ = 0.62 (0.45–0.79). We do not include the *T*_Elephantid-Mastodon_ parameter ancestral to elephant-mastodon divergence as it is not estimated in a stable way. Abbreviations: F, forest elephant; S, savanna elephant; A, Asian elephant; M, woolly mammoth; Lox, *Loxodonta* (African forest plus savanna elephant); Eur, “Eurasian” (Asian elephant plus mammoth).

We also used the MCMCcoal results to learn more about the timing of the divergences among the elephantids (Figure 2). To be conservative, we quote intervals that take into account the full range of uncertainty from both the fossil calibration of African-Eurasian population divergence (T_Lox-Eur_ = 4.2–9.0 Mya; Text S5), and the 90% credible intervals from MCMCcoal (*T*_FS_/*T*_Lox-Eur_ = 45%–79% and *T*_AM_/*T*_Lox-Eur_ = 46%–74%; Table 4). Thus, we conservatively estimate *T*_FS_ = 1.9–7.1 Mya and *T*_AM_ = 1.9–6.7 Mya. Our inference of *T*_AM_ is somewhat less than the mtDNA estimate of genetic divergence of 5.8–7.8 Mya [8]. However, this is expected, since genetic divergence time is guaranteed to be at least as old as population divergence but may be much older, especially as deep-rooting mtDNA lineages are empirically observed to occur in matrilocal elephantid species.

## Discussion

Our study of the extant elephantids provides support for the proposed classification of the Elephantidae by Shoshani and Tassy, which divides them into the tribe Elephantini (including *Elephas*—the Asian elephant and fossil relatives—and the extinct mammoths *Mammuthus*) and the tribe Loxodontini (consisting of *Loxodonta*: African forest and savanna elephants and extinct relatives) [36]. This classification is at odds with previous suggestions that the extinct mammoths may have been more closely related to African than to Asian elephants [37].

Our study also infers a strikingly deep population divergence time between forest and savanna elephant, supporting morphological and genetic studies that have classified forest and savanna elephants as distinct species [13],[16]–. The finding of deep nuclear divergence is important in light of findings from mtDNA, which indicate that the F-haplogroup is shared between some forest and savanna elephants, implying a common maternal ancestor within the last half million years [21]. The incongruent patterns between the nuclear genome and mtDNA (“cytonuclear dissociation”) have been hypothesized to be related to the matrilocal behavior of elephantids, whereby males disperse from core social groups (“herds”) but females do not [13],[38]. If forest elephant female herds experienced repeated waves of migration from dominant savanna bulls, displacing more and more of the nuclear gene pool in each wave, this could explain why today there are some savanna herds that have mtDNA that is characteristic of forest elephants but little or no trace of forest DNA in the nuclear genome [13],[14],[39],[40]. In the future, it may be possible to distinguish between models of a single ancient population split between forest and savanna elephants, or an even older split with longer drawn out gene flow, by applying methods like Isolation and Migration (IM) models to data sets including more individuals [41]. Our present data do not permit such analysis, however, as IM requires multiple samples from each taxon to have statistical power, and we only have 1–2 samples from each taxon.

Our study also documents the highly variable population sizes across recent elephantid taxa and in particular indicates that the recent effective population size of forest elephants in the nuclear genome (*N*_F_) has been significantly larger than those of the other elephantids (*N*_S_, *N*_A_, and *N*_M_) (Table 5) [13],[17],[19]. This is not likely due to the “out of Africa” migration of the ancestors of mammoths and Asian elephants as these events occurred several Mya [35], and any loss of diversity due to founder effects would have been expected to be offset by subsequent accumulation of new mutations in the populations. The high effective population size in forest elephants could reflect a history of separation of populations into distinct isolated tropical forest refugia during glacial cycles [33], which would have been a mechanism by which ancestral genetic diversity could have been preserved before the population subsequently remixed [1],[2],[23]. A Pleistocene isolation followed by remixing would also be consistent with the patterns observed in Asian elephants, which carry two deep mtDNA clades and where there is intermediate nuclear diversity. Intriguingly, our estimate of recent forest effective population size is on the same order as the ancestral population sizes (*N*_FS_, *N*_AM_, and *N*_Lox-Eur_) (Table 5), providing some support for the hypothesis that forest elephant population parameters today may be typical of the ancestral populations (a caveat, however, is that MCMCcoal may overestimate ancestral population sizes since unmodeled sources of variation across loci may inflate estimates of ancestral population size). An alternative hypothesis that seems plausible is that the large differences in intra-species genetic diversity across taxa could reflect differences in the variance of male reproductive success [42] (more male competition in mammoth and savanna elephant than among forest elephants, with the Asian elephant being intermediate [43]).

**Table 5:** Relative values of effective population sizes estimated by MCMCcoal.

	*Ν*_F_	*Ν*_S_	*Ν*_A_	*Ν*_M_	*Ν*_FS_	*Ν*_AM_	*Ν*_Lox-Eur_
*Ν*_F_	1	0.29 (0.20–0.40)	0.48 (0.34–0.67)	0.40 (0.27–0.55)	1.15 (0.57–1.89)	0.78 (0.44–1.22)	1.11 (0.78–1.52)
*Ν*_S_	3.58 (2.53–4.92)	1	1.71 (1.15–2.42)	1.40 (0.93–2.01)	4.03 (2.01–6.69)	2.74 (1.49–4.33)	3.92 (2.65–5.54)
*Ν*_A_	2.15 (1.50–2.96)	0.62 (0.41–0.87)	1	0.84 (0.59–1.14)	2.38 (1.26–3.65)	1.65 (0.87–2.69)	2.34 (1.63–3.22)
*Ν*_M_	2.63 (1.80–3.66)	0.75 (0.50–1.07)	1.24 (0.88–1.68)	1	2.91 (1.57–4.54)	2.02 (1.05–3.30)	2.86 (1.96–4.00)
*Ν*_FS_	1.00 (0.53–1.75)	0.28 (0.15–0.50)	0.47 (0.27–0.77)	0.38 (0.22–0.64)	1	0.74 (0.38–1.26)	1.08 (0.59–1.89)
*Ν*_AM_	1.42 (0.83–2.31)	0.41 (0.23–0.67)	0.68 (0.37–1.15)	0.56 (0.30–0.95)	1.55 (0.80–2.62)	1	1.56 (0.86–2.66)
*Ν*_Lox-Eur_	0.94 (0.66–1.28)	0.27 (0.18–0.38)	0.45 (0.31–0.61)	0.37 (0.25–0.51)	1.05 (0.53–1.71)	0.72 (0.38–1.16)	1

All quantities are expressed as a column-to-row ratio with a 90% credible interval. For example, the ratio of effective population sizes of forest to savanna populations = *N*_F_/*N*_S_ is 3.58 (2.53–4.92). We do not include the *N*_Elephantid-Mastodon_ parameter ancestral to elephant-mastodon divergence as it is not estimated in a stable way. Abbreviations: F, forest elephant; S, savanna elephant; A, Asian elephant; M, woolly mammoth; Lox, *Loxodonta* (African forest plus savanna elephant); Eur, “Eurasian” (Asian elephant plus mammoth).

The results of this study are finally intriguing in light of fossil evidence that forest and savanna lineages of *Loxodonta* may have been geographically isolated until recently. The predominant elephant species in the fossil record of the African savannas for most of the Pliocene and Pleistocene belonged to the genus *Elephas* [30],[34],[35]. Some authors have suggested that the geographic range of *Loxodonta* in the African savannas may have been circumscribed by *Elephas*, until the latter disappeared from Africa towards the Late Pleistocene [30],[34],[35]. We hypothesize that the widespread distribution of *Elephas* in Africa may have created an isolation barrier that separated savanna and forest elephants, so that gene flow became common only much later, contributing to the patterns observed in mtDNA. Further insight into the dynamics of forest-savanna elephant interaction will be possible once more samples are analyzed from all the taxa, and high-quality whole genome sequences of forest and savanna elephants are available and can be compared with sequences of Asian elephants, mammoths, and mastodons.

## Methods

### Data Collection

For our sequencing of mastodon, we used the same DNA extract that was previously used to generate the complete mitochondrial genome of a mastodon [8]. We sequenced the extract on a Roche 454 GS, resulting in 45 Mb of sequences that we deposited in the NCBI short read archive (accession: SRA010805). By comparing these reads to the African savanna elephant genome (loxAfr1) using MEGABLAST, we identified 1.76 Mb of mastodon sequences with a best hit to loxAfr1 that we then used in downstream analyses.

To re-sequence a subset of these loci in the living elephants and the woolly mammoth, we used *Primer3* to design primers surrounding the longest mastodon-African elephant alignments. A two-step multiplex PCR approach [24] was used to attempt to sequence 746 loci in 1 mammoth, 1 African savanna elephant, 1 African forest elephant, and 1–2 Asian elephants. After the simplex reactions for each sample, the PCR products were pooled in equimolar amounts for each sample and then sequenced on a Roche 454 GS, resulting in an average read coverage of 41× per nucleotide (Text S1). We carried out four rounds of PCR in an attempt to obtain data from as many loci as possible and to fill in data from loci that failed or gave too few sequences in previous rounds (Text S1).

To analyze the data, we sorted the sequences from each sample according to the PCR primers (746 primer pairs in total) and then aligned the reads to the reference genome (loxAfr1), disregarding sequences below 80% identity. Consensus sequences for each locus and each individual were called with the settings described by Stiller and colleagues [44], with a minimum of three sequences required in order to call a nucleotide and a maximum of three polymorphic positions allowed per locus (to filter out false-positive divergent sites due to paralogous sequences that occur in multiple loci in the genome). We finally generated multiple sequence alignments for each locus and called divergent sites when at least one allele per species was available. In the first experimental round we were not able to call consensus sequences for more than half of the loci, a problem that we found was correlated with primer pairs that had multiple BLAST matches to loxAfr1, suggesting alignment to genomic repeats. Primer pairs for subsequent experimental rounds were excluded if in silico PCR (http://genome.ucsc.edu/cgi-bin/hgPcr) suggested that they could anneal at too many loci in the savanna elephant genome.

### Filtering of 22 Divergent Sites That Have a High Probability of Having Arisen Due to Recurrent Mutation

Of the 1,797 biallelic divergent sites that were identified, we removed 22 to produce Tables 1 and 2. The justification for removing these sites is that derived alleles were seen in both African and Eurasian elephants, which is unlikely to be observed in the absence of sequencing errors or recurrent mutation. For the MCMCcoal analysis we did not remove these divergent sites, since the method explicitly models recurrent mutation.

### Weighted Jackknife

To obtain standard errors, we omitted each of the 375 loci in turn and recomputed the statistic of interest. To compute a normally distributed standard error, we measured the variability of each statistic of interest over all 375 dropped loci, weighted by the number of divergent sites at the locus that had been dropped in order to take account of the variable amount of data across loci. This can be converted into a standard error using the theory of the Weighted Jackknife as described in [45].

### Estimates of Genetic Diversity, Relative Rate Tests, and ILS

For our relative rate tests, we compute the difference in the number of divergent sites between two taxa since they split, normalized by the total number of divergent sites. The number of standard errors (computed from a Weighted Jackknife) by which this differs from zero represents a *z* score that should be normally distributed under the null hypothesis and thus can be converted into a *p* value for consistency of the data with equal substitution rates on either lineage.

### Phylogenetic Tree

To construct a Neighboring Joining tree relating the proboscideans in Figure S3, we used MEGA4 [46] with default settings (10,000 bootstrap replicates).

### MCMCcoal Analysis

To prepare a data set for MCMCcoal, we used input files containing the alignments in PHYLIP format (Dataset S3) [47], restricting analysis to the loci for which we had diploid data from at least one individual from each of the elephantids we resequenced (we did not use data from the loxAfr1 draft savanna genome, or from the second Asian elephant we sequenced at only a small fraction of loci). The diploid data for each taxon were used to create two sequences from each of the elephantids, allowing us to make inferences about effective population size in each taxon since its divergence from the others.

We ran MCMCcoal with the phylogeny ((((Forest_1,_Forest_2_), (Savanna_1_,Savanna_2_)), ((Asian_1_,Asian_2_), (Mammoth_1_,Mammoth_2_))) Mastodon). Since MCMCcoal is a Bayesian method, it requires specifying a prior distribution for each parameter; that is, a hypothesis about the range of values that are consistent with previously reported information (such as the fossil record). For the effective population sizes in each taxa (*N*_F_, *N*_S_, *N*_A_, *N*_M_, *N*_FS_, *N*_AM_, *N*_Lox-Eur_, and *N*_Elephantid-Mastodon_) we used prior distributions that had their 5^th^ percentile point corresponding to the lowest diversity seen in present-day elephants (savanna) and their 95^th^ percentile point corresponding to the highest diversity seen in elephantids (forest). For the mastodon-elephantid population divergence time *T*_Elephantid-Mastodon_ we used 24–30 Mya [30],[35],[48]–[50]. For the African-Eurasian population divergence time *Τ*_Lox-Eur_ we used 4.2–9 Mya [30],[35],[51]. For the Asian-mammoth population divergence time *Τ*_AM_ we used 3.0–8.5 Mya [30],[35],[52]. The taxonomic status of forest and savanna elephants is contentious. To allow us to test the hypotheses of both recent and ancient divergence while being minimally affected by the prior distribution, we use an uninformative prior distribution of *T*_FS_ = 0.5–9 Mya. This prior distribution has substantial density at <1 million years, allowing us to test for recent divergence of forest and savanna elephants. A full justification for the prior distributions is given in Text S5.

MCMCcoal also requires an assumption about the mutation rate, which is poorly measured for the elephantids. We thus ran MCMCcoal under varying assumptions for the mutation rate, to ensure that our key results were stable in the face of uncertainty about this parameter. For each of the three mutation rates that we tested, MCMCcoal was run three times starting from different random number seeds with 4,000 burn-in and 100,000 follow-on iterations. Estimates of all parameters that were important to our inferences were consistent across runs suggesting stability of the inferences despite starting at different random number seeds (we did observe instability for the parameters corresponding to mastodon-elephantid divergence, but this was expected because of the high rate of mastodon errors and is not a problem for our analysis as this divergence is not the focus of this study). We computed the autocorrelation of each sampled parameter over MCMC iterations to assess the stickiness of the MCMC. Parameters appear to be effectively uncorrelated after a lag of 200 iterations. Given that we ran each chain over 100,000 iterations, we expect to have at least 500 independent points from which to sample, which is sufficient to compute 90% credible intervals. The detailed parameter settings and results are presented in Text S4.

## Supporting Information

Dataset S1 **All primers used in this study.**
(0.27 MB PDF)

Dataset S2 **Table with polymorphic positions.**
(1.49 MB XLS)

Dataset S3 **Input file (PHYLIP) for MCMCcoal.**
(0.10 MB PDF)

Figure S1 **Mastodon shotgun results.** (a) A histogram of read length (in nucleotides) of all putative mastodon sequences gathered in this study by shotgun sequencing. The longest sequence is 202 nucleotides long, and only the longer sequences (to the right of the black line) were used for primer design. (b) Percent identity of all mastodon-loxAfr1 alignments. The mean percent identity is 95%. Only sequences with an identity of more than 87% (to the right of the black line) were used for primer design.
(0.21 MB DOC)

Figure S2 **Analysis of 454-sequence data to build multiple alignments.** Sequences were sorted according to their barcode to identify the sample, and then the sequences (now per individual) were further sorted by the 5′-primer and aligned to the reference (loxAfr1) using a similarity threshold of 80%. Consensus sequences were called per individual and consensus sequences of the various individuals were merged into multiple sequence alignments including the mastodon shotgun sequence (red).
(0.14 MB DOC)

Figure S3 **A Neighbor Joining tree built with the software MEGA4 supports the topology (((Savanna, Forest),(Asian, Mammoth)), Mastodon).**
(0.04 MB DOC)

Table S1 **Samples used in this study.**
(0.04 MB DOC)

Table S2 **Summary of loci that we attempted to amplify.**
(0.03 MB DOC)

Table S3 **Target performance for different rounds of the experiment.**
(0.11 MB DOC)

Text S1 **Data collection.**
(0.07 MB DOC)

Text S2 **Error Rate Assessment.**
(0.04 MB DOC)

Text S3 **Genomic distribution of loci.**
(0.03 MB DOC)

Text S4 **MCMCcoal analysis to infer population parameters.**
(0.11 MB DOC)

Text S5 **Justification for prior distributions for MCMCcoal.**
(0.06 MB DOC)

## Abbreviations

ILS: incomplete lineage sorting
IM: Isolation and Migration
mtDNA: mitochondrial DNA
Mya: million years ago
