# Supplementary material for: Genomic DNA Sequences from Mastodon and Woolly Mammoth Reveal Deep Speciation of Forest and Savanna Elephants
Source: PLoS Biol. 2010 Dec 21;8(12):e1000564. doi: 10.1371/journal.pbio.1000564 (PMC3006346; doi:10.1371/journal.pbio.1000564)
Supplement: Dataset S1 — All primers used in this study. (0.27 MB PDF) [file pbio.1000564.s001.pdf]

A.1

Target and primer information for experimental round A.1: read ID corresponds to the read in the short read archive SRA010805, beside primer sequences PCR product length with and without primers is given, as well as the percent identity of the respective mastodon-loxAfr1 alignment, "# of hits isPCR" gives a number how often the respective primer pair anneals to loxAfr1, and a "yes" in the row "consensus for all taxa" means, that sequence information is available for all taxa. The last two rows show scaffold ID and position of the targets used in the MCMCcoal analysis aligned to loxAfr3.

target ID	read ID GenBank SRA010805	F-Primer 5'-3'	R-Primer 5'-3'	product length in bp amplicon	product length in bp w/out primer	Mastodon-loxAfr1 alignment % identity	# of hits isPCR loxAfr1	consen-sus for all taxa	aligned loxAfr3 scaffold ID	loxAfr3 scaffold position
A001	EK3X9LI01BLRFR	TGGTCTCGAAGTCAGACAAGAG	TCAGGACTTGCCTGTTCAGTT	186	143	0.98	2	yes	50	16,857,885
A002	EK3X9LI02F3RBB	GCATTTTGATTGCATGTTTGA	AAATTTTGAGATCTGTCCCGAA	190	147	0.98	6			
A003	EK3X9LI02HKE1R	TATAGTTCCAGGCTCCACGG	ATAAGCAGTCAAGAGCCCGA	161	121	0.95	0	yes	13	31,772,950
A004	EK3X9LI01DC8H2	TGTTGTTAGGTGCCATCGAG	CGGAAAAGAGGAAGACCCTC	161	121	0.97	64			
A005	EK3X9LI01BHMHV	GCCCATGTCAATCAACAGAA	TGGCCCCTCTCTCTATGTGT	194	154	0.92	93			
A006	EK3X9LI01A2O1J	AGGCGAGAATACGCTCAAAC	GCTCCTCTACTCGGGCCTAT	184	144	0.89	435			
A007	EK3X9LI01C7BYB	TTTTCCTGTGTTTGTGCATGT	AGCTAATGGAAGAAATGATGAAGT	172	127	0.90	21	yes		
A008	EK3X9LI01CV00E	GAACACGGTTTCCAGGAATG	GGGTCCTAGACAGAGTGGGA	161	121	0.90	3			
A009	EK3X9LI01C7XBW	TTTCTATTCTCCATGAATGAACCA	CAAACAAATTGAGAAAACAGTGC	156	109	0.92	4	yes	11	58,944,724
A010	EK3X9LI01DQL4E	AGATGAAACACGGGAGGCTT	GCAGCAGCGGAATTATTCTT	181	141	0.98	27			
A011	EK3X9LI01AQJCF	GCTTCATTCTGTTGTGCGTG	ACTCTGCTCTGTGATGCTGG	136	96	0.98	8	yes	12	23,353,795
A012	EK3X9LI01BCGQI	CACTCCCAGCACACCAGAC	GAGTGGGTTCATTGTCACCA	152	113	0.98	1	yes	4	2,618,296
A013	EK3X9LI02G5E9S	CAATGCCCTGAACCAGTCC	GTAGGCGGAGCTTATGATGG	188	149	0.96	807			
A014	EK3X9LI01AIIP7	CTTTCTCTGGGCAAAGTCCA	CATCCTCCCAACCTTTTCAA	145	105	0.87	0	yes	4	598,729
A015	EK3X9LI02JWLAQ	GAGTTACCGAGAGGGGGAAG	TCATCTCAGTGACCAAACTTGAA	192	149	0.88	1			
A016	EK3X9LI01EN54P	TCGGACAACAGGACCCTTAC	CTGTGCCATGTGGAAACAAA	151	111	0.99	4	yes	34	682,004
A017	EK3X9LI02I9G0E	CTGCTCTGCCACCAATCTTC	CAGTCTGTGGGCATGTCTGT	177	137	0.98	2	yes	3	71,685,234
A018	EK3X9LI02GC7NK	CCATGGAGGACAGGTTTCAG	AAGGCAGCTCTTTCTCAGTCA	193	152	0.94	334			
A019	EK3X9LI01C0K93	AACCACCATTGTCCACTTTG	CAGCTGCCAGCAACAATG	145	107	0.99	1	yes		
A020	EK3X9LI02F9G0Q	GAATAACATCAGACGGTGCG	TAGCTGGAACTCTTGCCTGG	173	133	0.97	0			
A021	EK3X9LI01CTJZY	GCTTACCACACACAATCCCC	AGAAATGTACCATGAGCGGG	166	126	0.93	3	yes	3	18,742,916
A022	EK3X9LI02H02GU	ATACAGGAGGGATCCTTGGG	ACTCACATATCCCCTGGTGG	163	123	0.96	1233			
A023	EK3X9LI02GCTKJ	TGACATGTGCAAAGAGCTGG	TGGTTCCTGCGTCGTTTAAT	149	109	0.96	269			
A024	EK3X9LI01EVQLG	TGTCCTCTCCCTCCACTCTC	GATTGGACTGGACAATGGGT	172	132	0.94	1322			
A025	EK3X9LI01C4P1K	GAATTATTTTGCCAATTGACTTAGA	GGTGAAGGGAAAGACAACACTC	170	123	0.96	2257			
A026	EK3X9LI02FFWKW	CCCAGCACTTAACAAATACTTAAAA	AACTAACATGGTCAGCCTTGC	198	152	0.98	3	yes		
A027	EK3X9LI01CDWFL	CGTGGGTAGAGAAGAGCAGG	ATTATTCGTCGCCGGAGG	133	95	0.97	800			
A028	EK3X9LI02IGAJD	CACAGCCAGAAGCCTCCC	GGATATCAGTAGGCAAGGCG	158	120	0.93	1807			
A029	EK3X9LI02H3CND	CGTTGCTTGCTGAAAGTGAA	TCCTTGACAACTTCAATCTTTTCTC	166	121	1.00	0			
A030	EK3X9LI02HH3QY	AGCAAAAATTACTCAAGTGGAAA	GTGAAAATGGAGCCCAAGAG	164	121	0.93	55			
A031	EK3X9LI02GL1UM	TGTTGAAAAGCAAAGATGTCACT	TTTGCCAACACCATAATTCAA	150	106	0.93	270			
A032	EK3X9LI02G4ANS	AATAGGCCCGAAGGGAAGT	TTTCTTCCGAAAATCTCCGTT	156	116	0.94	5			
A033	EK3X9LI01B83XL	TGACTGACAACAGCAAGGCT	GCAACATCATGATAAGTGGAGAA	195	152	0.97	1	yes	146	1,412,236
A034	EK3X9LI01AEV4R	GGTACCAAACCCTTGCCAT	GCTGTGTGGAGTTAGGGACC	170	131	0.98	1	yes	14	7,155,272
A035	EK3X9LI01A6LR5	CACCTTACTCCGTACTCCCA	CACTTGAGACTGTGTTGGCA	145	105	0.93	748			
A036	EK3X9LI01AHAIX	CATGAAAAATGCTGTTAAAACCC	TCACTCTAATTCTTCATCCTTCCA	170	123	0.99	5	yes	3	65,357,915
A037	EK3X9LI01DFI3G	CAGTTGGTCATACTGCGGTG	GACTGCTAGGCCCTTCTTCC	149	109	0.94	1			
A038	EK3X9LI02IMKVX	AGGGATCAAATGTTGTTGTTCA	TCTTCCCCATTTCTCAGAGG	135	93	0.97	2	yes	9	48,548,100
A039	EK3X9LI02IRFTU	TCCCAAGTCAACTAGATAACCTCA	GGAAAAGGCTCCTTTCTCTGT	194	149	0.95	2			
A040	EK3X9LI02JSOT0	AAGGCCACTCTTGGCTTTCT	GTTTGCTTCAAAGCTGTGGG	174	134	0.97	2			
A041	EK3X9LI01AIALH	TGTGTGCAGGAAAGTATGTGC	ATTCCGCTGCTGCTTTGTAT	155	114	0.94	895			
A042	EK3X9LI01BE9T9	GACATCCAAACATTTTCAGGC	TGGAAGGTGCTCAGTGCTTA	186	145	0.99	0	yes	23	26,582,346
A043	EK3X9LI02FIY9W	GAAGAGACTGGGAGAGCAGC	CACACTATGTCGTCAGGGCA	176	136	0.90	28			
A044	EK3X9LI02GFDDD	GTGGGTCTACAAATCACCGC	CACAGTGATTGAAAGCGGAA	147	107	0.95	0	yes		
A045	EK3X9LI02I66QU	TGGACTGCCAAAAGAATGAA	CCATCTCATTAAAGGCCTTCCT	190	148	0.96	11			
A046	EK3X9LI01A3SP9	GCTGCAGCTGCTTTGAATAA	TGATGCAGTGGTTGAGGTGT	184	144	0.99	1	yes	29	18,322,968
A047	EK3X9LI01AZEP9	GGGACTCCTAGGACAATCCAG	AAACTGCACCTGGCCTTATG	151	110	0.93	1	yes	7	8,254,015
A048	EK3X9LI02GRG6Q	AGGGGAACCATGTCCTTGAT	GAACTGGACAGCCTGTGGTT	158	118	0.96	1	yes	86	2,349,909
A049	EK3X9LI02HZ5M7	TGTTGTTGTTAGGTGCTGTGG	GACCCTCAAGGAGATGGTTG	154	113	0.87	15			
A050	EK3X9LI02JDDAK	AGGAATCGGGTGAGTGAGTG	CCACACTAAAAGGGACCCAA	146	106	0.98	1	yes	27	20,268,499
A051	EK3X9LI01AX9UU	TTGTGATCAGAGTTTTGCGG	ACACTGCAGGGGCTCAAC	191	153	0.95	1			
A052	EK3X9LI02G8P4Z	GTCTCTTCTGATGGTGGGCT	CATGTGGAGAACTTCCTTTGC	157	116	0.98	2	yes	1	78,994,943
A053	EK3X9LI01E2Y6Z	TTGCCAACATCACAATTCAAA	TGTTAAAAACCAAAGATGTCACC	149	105	0.96	4			
A054	EK3X9LI01E21TS	CGACTCAACACATCAAGCAAA	TCCCTCAATTCCAACAGCAT	163	122	0.96	0	yes	37	16,375,505
A055	EK3X9LI02JORZ9	GCTGAGCAAATAATCTGAGAACC	CTGAAGGCTGTGGTCTTTGA	168	125	0.97	0			
A056	EK3X9LI02JVJWB	TTTGGGTTAAATGGACAGCC	TTCCTGCAATAAAGATTGGC	147	107	0.99	0	yes	22	15,681,214
A057	EK3X9LI01DC6HA	AGCTCCAAGACCTATGCTGG	CTCGGCTGACTTTCACTTCC	191	151	0.96	0	yes	75	5,427,047
A058	EK3X9LI01CQTZA	AACCCATCTGCGTATACTGAAAG	TCTTCATTTGGGAAAGATTTTGA	189	143	0.94	76			
A059	EK3X9LI01BDI18	GCCAGAATGCTCCTTAGATGC	ATGCTTGAGCCCATTATTGC	158	117	0.99	0			
A060	EK3X9LI02FQL3R	GCCAAAGTGGCACGTTTTTA	TAGGGAGCCAATGAGAGCAT	172	132	0.91	5			
A061	EK3X9LI02HQFHE	TGGTTAAGAGCTCAGGCTGC	ACTCACTGCCATCGAGTCAA	134	94	0.92	20			
A062	EK3X9LI02JWOIR	TTTTGTTTGGTTTCGGTGGT	GCTCCCCAAATGATTAAGTGTT	177	135	0.95	5			
A063	EK3X9LI01BCKUW	ATGGCTGCAGTAGTGCATTG	CACACAGGTGAATGTCTTTGC	175	134	0.90	1			
A064	EK3X9LI02HHRV7	TAGCAAACTCTTGGCTGCCT	CTGCAGTCAGTCCTTGCAGA	149	109	0.97	1			
A065	EK3X9LI01C7PXN	ATGGTGATGGGAGCTTTGAC	AGAAAACAGACTGCGTGCCT	166	126	0.98	1			
A066	EK3X9LI01C28PT	TCTAGGGACTGGACCCTCCT	TGCATAAAAAGATGGACAATGAA	181	138	0.92	1			
A067	EK3X9LI01BV10S	ACTATCTCGCCAGGGCAAC	GTTTTAGCTGGAAGGGCTTG	183	144	0.91	4693			
A068	EK3X9LI01BBLJX	TGGAGGAACTGACTACGGCT	GCACCCAGGAATATAGACAAAGTT	170	126	0.97	89			
A069	EK3X9LI02II2C9	TCAGTTAACACCCACATCTGTCT	GCCTTCCTTTGCTTTCCTTT	202	159	0.94	3	yes	5	70,359,926
A070	EK3X9LI01AG83P	ATTTCTCTGGGCAGCGTGT	GTCTCTTTTCTTTCGGCCTG	174	135	0.91	31			
A071	EK3X9LI02HHLB0	CCAATGCCATTAAAATCAGAAA	TTATGAAAGTGAATTAGACACGAAA	181	134	0.98	3	yes	0	116,134,150
A072	EK3X9LI02JM0Q8	CTGACGAAAGCGGTTAGAGG	CAAAGCGAGCCTTCACATTA	181	141	0.97	4158			
A073	EK3X9LI01C26R9	ACTGCATGTGCGTCACTTCT	CGTGGAGCTCTAGCGGAG	155	117	0.96	1652			
A074	EK3X9LI02HUAO9	GCTCAGAGTTTGTGACCCGT	CCGACATCCTTTCTTTCGTC	169	129	0.96	749			
A075	EK3X9LI01DSVG0	CACCCTGAGGGACTGAGTTG	TTTGCTACAGAAAAGATCAAAACA	171	127	0.90	4	yes	41	12,064,693
A076	EK3X9LI02FHZ2J	CAAATTTCACTGGCATTCATCA	AGATCAGCCCAAAGGGTTTT	197	155	0.95	3	yes	4	65,387,475
A077	EK3X9LI01CPQ0Z	GGACTGGAAAGCAAAACCCT	GCTTAAGGCATTTGTGAAATCAT	206	163	0.99	3	yes	4	86,446,334
A078	EK3X9LI01C26GN	GCGGATTCCTGTGTATGGTT	TGCTGAGAGTGTGTGTGTGC	167	127	0.94	6	yes	19	27,050,379
A079	EK3X9LI02H07QM	GCAGACTCTGGACTGCTGG	AAAGGAGAGCACAGGCAAAG	155	116	0.90	1848			
A080	EK3X9LI01B9LJB	ATAGGCCCGAGTAGAGGAGC	ATTCTGGCCTGTGGGACTCT	160	120	0.92	768			
A081	EK3X9LI01DKF6W	GAGCGATGTTTCTCCACCC	ATGAAGGATTCGGGACCTTG	162	123	0.99	1272			
A082	EK3X9LI02GF929	AAAACCAGAGCCAAGTGGTG	CAGATCTTGAGATGGGCCAC	157	117	1.00	2	yes	6	941,096
A083	EK3X9LI01D541S	TCTTGCTTTGGGACGGATAC	ATGACGGGGCTTCTTTCTTT	171	131	0.97	0	yes	69	2,270,628
A084	EK3X9LI01BVALO	GCATGTGTGAGCTCTTCCCT	GCAGGATTCTTGCTTGCTTC	148	108	0.97	2	yes	123	1,527,368
A085	EK3X9LI02FPCGC	GCTCCACTGTGTGAGTCATCAT	TGCTGCAGAGACAGAAGGAA	165	123	0.92	1	yes	173	542,484
A086	EK3X9LI01BLH35	ACACCTCTGAGCTTCCCTGA	TGCTGTGTCGACTCCTCTTG	154	114	0.98	0	yes		
A087	EK3X9LI02HII8H	AATTTGCAAGGCTCAGTGCT	CTGCCGGTTCTCTACTCAGG	169	129	0.95	1	yes	11	66,322,536

target ID	read ID GenBank SRA010805	F-Primer 5'-3'	R-Primer 5'-3'	product length in bp amplicon	product length in bp w/out primer	Mastodon-loxAfr1 alignment % identity	# of hits isPCR loxAfr1	consen-sus for all taxa	aligned loxAfr3 scaffold ID	loxAfr3 scaffold position
A088	EK3X9LI02JBXZW	TGCTGTTGTCAAAGCCTGAG	AACGGCAATCCCATTTTACA	154	114	1.00	1	yes		
A089	EK3X9LI02IOGIR	TGCAGGTATCAGGAAGACCC	TTTACTGCCTGCACTTCGTG	185	145	0.89	1			
A090	EK3X9LI01AVFGZ	TACATTCGCCTACCTCCCTG	TGGGCAGGATTCAATAGGAG	174	134	0.95	1	yes		
A091	EK3X9LI02HYKZI	CCTTCTGCACCCTGGAAGTA	CATTTCCACCCAGATTCCAC	154	114	0.96	1	yes	48	3,590,814
A092	EK3X9LI02GY7B7	GTAGGCTTTCCCAGGGTGTG	CATGGAGACAGTCCCACCAC	148	108	0.98	1548			
A093	EK3X9LI02F4X94	GACTTCTCCTACCGGCTGC	CGAACTACTACGGGGAAGCA	163	124	0.99	239			
A094	EK3X9LI01ET8NT	CAGACATCAGGAGTCTGGAGG	TCCATGGGGTAACTTTTTGAA	161	119	0.99	2	yes	61	591,902
A095	EK3X9LI01C591E	GGAAGCAAGAGGTCATTAAAAA	TGGGCTAGGCACACCTTAGT	177	135	0.96	10			
A096	EK3X9LI02ITBQL	TTGGAGCTTTGATGTCCCAT	AACACCCTTGCCCCTCTAAG	167	127	0.96	2			
A097	EK3X9LI02JWQH1	ATGCCCCCTCCTGAGATAAT	TCTGCGGTACATAGGGCTG	170	131	0.96	1	yes	13	5,005,567
A098	EK3X9LI01A8RJG	CACCCATGGAAGAAGCAGTC	CCTTATTCATTGTCCAGCATCA	180	138	0.99	1			
A099	EK3X9LI02IKMWQ	AGCAGGTGGTTGAGTGTGTG	GTGCTTGGCCTAGGAAACAA	181	141	0.94	1	yes	44	3,564,342
A100	EK3X9LI01CU5LO	GAGGTTGCATCTGCCCAC	GAGAGAGCCTAGAGACGGCA	168	130	0.90	515			
A101	EK3X9LI02IYRPJ	AGAAGTCACTGTAAGTGCGGAA	ACTGCCTTTTGGCAAATTCA	186	144	0.96	2	yes	4	15,013,685
A102	EK3X9LI01EAN2W	GGCTAGACATTTGGTTTTCAGT	CCCTTGCCCATGTCTGTTAT	176	134	0.99	5			
A103	EK3X9LI01DG8OA	GGTGTGTGCTGAGAAGGTGA	ATTCCGCTGCTGCTTTGTAT	143	103	0.94	1353			
A104	EK3X9LI01D826E	TTCGAGTAGCTTCTGGCTCC	GCCAACTCTTCAGACAGGGA	156	116	1.00	32			
A105	EK3X9LI02HLU5N	GGGGAAGAATCTAGGGTCCA	ACTAGCTGTCGCACCCACTT	176	136	0.97	1	yes	11	7,758,960
A106	EK3X9LI01DD6LB	TCAGCTGATCTGAGTGTCACAA	TACAGGATGCACGAGGCATA	170	128	0.95	5			
A107	EK3X9LI02JTIFO	GAAGCATTGAAATAGCTGCCT	TTGGACTTTTTAAACCATCTACCTG	172	126	0.98	1	yes	34	1,459,684
A108	EK3X9LI02GWFA6	CACTTGCCAGAGCAGAACAG	GGCCACACAGAACTCACAGA	180	140	0.97	23			
A109	EK3X9LI01CREZQ	GCAGGCACACAAAGGGGTCT	GGCGCGGGGCCTAGATTT	133	95	0.93	1	yes	34	3,759,193
A110	EK3X9LI01D0Z43	TAGATGAAAACCCGCCATTC	GCTGGGTCAACTTAATTGGAATA	183	140	0.97	0			
A111	EK3X9LI02HJ5E6	CGTGTGTTGGACCAGCTGTA	GCCTCTGGATCTTGGTTTGA	171	131	0.98	1			
A112	EK3X9LI01D2RKE	GTCTGCTTTGAAGGGAGCAG	CCCATAGGGTTTCCAAGGAG	161	121	0.98	0	yes	75	7,727,987
A113	EK3X9LI02G11JT	GACCTCACTGGTCATTGGCT	CTTGGCAAGAGCAGTGTTCA	176	136	0.97	2	yes	53	15,437,941
A114	EK3X9LI02FWX64	CTAGTTCTCCACAGCCCTCG	TTTTGGCTGAGCTAATTGGG	175	135	0.98	1			
A115	EK3X9LI01ANZ5L	ATAAGCCCCGCCTACTCTTC	ACCGGCCGACATTAATTTTT	150	110	0.96	15			
A116	EK3X9LI01BAG0E	GGAAGGTCAGCCTAATTGGA	TGGGATGCAGAATGTTTTCA	166	126	0.98	235			
A117	EK3X9LI01EFDHQ	CAGGAGCCAAGAGTTGGAAA	TCTGTGTTGCACCTGTACCC	178	138	0.99	1	yes	86	4,337,419
A118	EK3X9LI02ITWWD	ACCTTGAGCACCTTTCCCTT	TCCTTAGGTCCCTGTTGTGG	176	136	0.98	335			
A119	EK3X9LI01AOCZE	GCTGCTGCAGTCTGAAATTG	TTGCCGTTTATGTTGGTGAA	159	119	1.00	1652			
A120	EK3X9LI01BVY86	CCCACACTCACATATCCCCT	TTCACTGCCTCGCCATTC	145	107	0.93	70			
A121	EK3X9LI02F5KTB	GCTGCTTTCCCAGTCTCTTC	GACTTCACCCAGCCCTCTG	194	155	0.92	147			
A122	EK3X9LI01ETJ69	GTTCCAACTCACAGCAACCC	CTGCAGGACCCAGGAATTTA	151	111	0.98	3	yes	3	37,511,028
A123	EK3X9LI02I0H9I	GTGGAGCGCGTTCTCTGA	ACGCGATGAGTTATGGTGGT	186	148	0.99	1	yes	94	3,410,871
A124	EK3X9LI02IJEMP	CGAACCCAAGATTGGTAGGA	GGGCTTTTGCTAGTTCTGGA	171	131	0.93	9			
A125	EK3X9LI01EHT4U	TGATCAGGGCTCCTAGGTTG	TGGTCAGTTGAGCAGACTTTG	174	133	0.97	1			
A126	EK3X9LI01EBDG8	AACTAAAACAAGACGGCCCC	TCTCTCTGCTGAGACTGCCA	189	149	0.97	1			
A127	EK3X9LI01BJV3L	TGCGATTTCTCAAAATGAAAGA	TCATATGCTGCCTCCTGAAA	184	142	0.95	4	yes		
A128	EK3X9LI02FXPPF	GGTTTGAGTCCATCCAGAGG	TGTGTTCTGTGTGTGTGACCA	165	124	0.92	4	yes	190	544,216
A129	EK3X9LI02IM99N	CTGACCTAGGCTGCCTTCTG	CAGAGAAGCCTCAGCCCC	169	131	0.88	279			
A130	EK3X9LI02H1ZVD	TCCCATGTGGTTCTGACAGT	AGGTTAGTACCCCACCCTCC	161	121	0.88	2			
A131	EK3X9LI01C1TEE	ATAGCCTGTTTCCAGGAGGC	CCTTGATGAGCTGAGGGAAC	163	123	0.97	1			
A132	EK3X9LI02JZRK0	TCCAAGTCATTAAGGCCGAC	TCTGAAAAGCATTAGCCAGTGA	150	108	0.96	52			
A133	EK3X9LI02F6FN8	TTCTGATCTATGGCTGCGTG	TTATGCCTTTTTAGAAGGGGG	163	122	0.96	1	yes		
A134	EK3X9LI01DOCPA	TGGCATTAGTGGTTGGTGTG	TGCCAGGAATGACAAATTAAA	173	132	0.93	507			
A135	EK3X9LI01C8NQ2	CTGTGTTTCCCCACAGGAAT	AAAGGACGCTTTTCTCCCTC	157	117	0.98	4129			
A136	EK3X9LI02I36U7	CTAGCAGGCTGGGGCTGT	CCCCCAGAAGTCACTTTGTT	184	146	0.97	0			
A137	EK3X9LI01EDFZR	ATGTCAGATGGATCCTGGCT	AGGTTCCTCATGAGCACAATTA	163	121	0.94	1232			
A138	EK3X9LI02GH9IC	CCCAGCCACCCTAGACATT	GGTTCACAGGTCTTCGCTGT	166	127	0.93	3	yes		
A139	EK3X9LI01DWNEV	CAGCAGTGTCACAAGGCAGT	TGGCCTTCACTTCACCTACC	181	141	0.97	701			
A140	EK3X9LI02INCWL	AATCTTAGCTCCTCAAAGGCG	TGGCTGACCCAATACAGCTA	159	118	0.97	1	yes	9	48,345,794
A141	EK3X9LI02GMTFT	CCCAACTATGCCAGGACCTT	TTTTAACCTCTGAGCCCTGC	170	130	0.95	1	yes	69	9,882,985
A142	EK3X9LI02GNGGB	ACTCTGCAAGGCGGTACATC	ATCAGCATAATACGCCCGAC	165	125	0.99	1			
A143	EK3X9LI02GKZUQ	TTATTGGCCCAGTTGTGAGG	TTGAAGAAGAATGGGGCATC	189	149	0.96	70			
A144	EK3X9LI02GYYTB	ATGGGCTGCAAAATAAGTGG	GATCCGGACTTAGGAGCCC	184	145	0.98	1	yes	5	24,165,720
A145	EK3X9LI02G8NUG	CCCCTCTCCTGTGGTTTGTA	GATGTGTTTCATCAAAGACTCAGTA	153	108	0.95	5	yes	3	70,689,859
A146	EK3X9LI01E5Q3R	TGAGTATCTTCACCCCTGGC	GCAGCCAGCCAAATAGGTAG	162	122	0.96	1	yes		
A147	EK3X9LI02IQHZQ	AACATAGGCCCGAACGGT	CTTGTTTCCTGCACGGCT	161	125	0.91	61			
A148	EK3X9LI01BADUP	AGGGCCCTAGGATTTTCAGA	GCTGGCTACACTGAACCACA	194	154	0.96	3	yes	72	1,311,673
A149	EK3X9LI01CEAIR	GTCCTGATGGGGAACACAAC	TGTCTGGGAATGGTTCCTGT	174	134	0.94	100			
A150	EK3X9LI02FF96G	GGTTCCTCATGAGCACAATTAAG	GATCCTGGCTGAAAGCAGAG	152	109	0.96	17			
A151	EK3X9LI01EOTRO	TGTACGTGGCAAAGAGGTGA	AAGGAGAGCACAGGCAGAGT	191	151	0.92	1527			
A152	EK3X9LI01EPXIX	TCTTTGAATTATGGTGTTGGTGA	TCCAAGGAGTGGTCTCTTCTG	169	125	0.93	6			
A153	EK3X9LI02INS8N	GGAGAAAAGTGGGATGCAGA	CATGCCTCACCAGTATCATTTT	193	151	0.91	196			
A154	EK3X9LI02FZCNA	CCGCAATTAAAATGAGTCTGG	GCTAGCTCTGAATGAACGGG	176	135	0.95	3	yes		
A155	EK3X9LI02G11L8	ACACTGGAGCACAGGCAAG	CACGTACCTCAAGGTGCTCA	183	144	0.93	0	yes	8	42,387,873
A156	EK3X9LI01CD6WH	GTCCCCCAGTCCTGCTCT	ATGGGGGCACAGTGTTTAAG	183	145	0.93	2	yes	97	3,528,068
A157	EK3X9LI02GUVFP	AGGCAAGAATAGAGGAGCCC	TGCCTGTGGGACTCTATTGG	183	143	0.88	780			
A158	EK3X9LI02GM7LC	CTTGGGTCAGGTGCAACTTT	GAATCCACAATTGACACCCA	151	111	0.91	6			
A159	EK3X9LI01B76F7	CAGCGCCTCCCTCGCCGAC	GCGTCGCCAGGGGACACCAC	147	108	0.96	10			
A160	EK3X9LI02IKVA4	ATAGCAACCCTTGCACAACA	GACACAGTGGCTGCAACAAT	162	122	0.96	0			
A161	EK3X9LI02F8K36	ATTCTTGCTCCGGGAATCG	CATACGCCTGAACGGAGATT	190	151	0.92	1076			
A162	EK3X9LI02HLJ9S	GGTCAGGCCCACCTTAGTCT	CAAGGATTTCATTTTGCTTGG	156	115	0.99	14			
A163	EK3X9LI02I2Z3L	CTGTGTTGGCATCTCCTGTC	ACAAATCAAGTCAGTCTGTCACAT	172	128	0.95	1503			
A164	EK3X9LI02FL5QN	AACTCGGCATTATTCCGTTG	TCGGCTACATGAATTAAGCAA	189	148	0.90	0			
A165	EK3X9LI02GFCPO	CATAATCCCCACGATATGCAG	TGCCCTGAAGTTACAAATGATG	163	120	0.98	1	yes	32	18,723,414
A166	EK3X9LI02JIUKM	ATGGCTGTTGTTGGAAATGG	AGCAGGTGACTGGGGTACTG	160	120	0.97	3			
A167	EK3X9LI02I4GH3	GCCCAGAGAAATCCTAGCAA	AATTGGCAACAGCCTCACTT	146	106	0.94	1			
A168	EK3X9LI02GZ29S	GGACTGATCCCTCCTGACAA	CCTTTGAATTGTGGTGTTGG	164	124	0.97	0			
A169	EK3X9LI01CFYE6	CAAGTCTTGCAACTCCTCCC	CCTTCTGGTGAACCTCTGACA	162	121	0.92	3			
A170	EK3X9LI02I8SIM	GGTTGAGAGGAGGTTGGGAT	AATACGGTAGGGGCGGAC	187	149	0.97	3			
A171	EK3X9LI01CDP0M	CCTGTACGTGGCAAAGAGGT	AAGTCAACTGAAGCCCGTGT	177	137	0.94	1734			
A172	EK3X9LI01B941C	TAAAGTCCCGCTCCAGAGAA	GACACCGTTCGGGTCTATTC	164	124	0.95	557			

target ID	read ID GenBank SRA010805	F-Primer 5'-3'	R-Primer 5'-3'	product length in bp amplicon	product length in bp w/out primer	Mastodon-loxAfr1 alignment % identity	# of hits isPCR loxAfr1	consen-sus for all taxa	aligned loxAfr3 scaffold ID	loxAfr3 scaffold position
A173	EK3X9LI01COG9U	GGACAAGTAGTCCAATGTTGCC	TCGGGAATATGCTCTCAACA	157	115	0.97	1	yes	7	41,888,057
A174	EK3X9LI02FPNPZ	CACAAGGTAATTATGAGCCCAA	ACTGCTGTCCTGCTCCTTCA	156	114	0.98	185			
A175	EK3X9LI02HKA5F	CCGAGCAGTACCTCAGAAGA	GTCAACAGGCCTTCCTTCC	181	142	0.94	130			
A176	EK3X9LI02JYAKH	CGGGGCTCTGCTATTCAG	GTCCCGCAGACCACGATAG	164	127	0.96	22			
A177	EK3X9LI01DNN7S	TCCCCATCCTTTCTCTTGAA	TGACCAACCAACAAAACAGG	186	146	0.96	2			
A178	EK3X9LI01A38EU	CAATTGTGGAAACATCAAAACTG	CCATTGGCTAGTCCCAACAT	160	117	0.96	3	yes		
A179	EK3X9LI01DLN0U	GGCTATGGTGTCAATCCACC	GCCGAACGAAGGAGAAAATC	184	144	0.97	1	yes	20	38,002,468
A180	EK3X9LI02GECMC	TGACAGATAAAGGGCAAGTGG	TGCCTCCCTCTTGATTATGG	151	110	0.95	3	yes	53	5,839,203
A181	EK3X9LI02JOYBY	TAGGATCTCCCTTTGGGTTG	TGTGCAGGGTGTTATGTGCT	185	145	0.92	1			
A182	EK3X9LI01BKX6S	AGGACTGGAGCTCGGTAACA	CAAGGTGATGGAGTACCCTGA	170	129	1.00	0	yes		
A183	EK3X9LI02JX96K	GCTGGTGAGGAGTGAGCTTC	TCTCCCCTAATGTGACAGCC	161	121	0.96	95			
A184	EK3X9LI02IDCDD	GCTTAAAATGAGCCATCCCA	CAGTCTCGTGGTCTCCTGGT	165	125	0.90	11			
A185	EK3X9LI01D32G7	AGGTCAGAAAGCCCCGAG	ATTCTTGGTCCGGGAATCTC	165	127	0.96	6			
A186	EK3X9LI01BIKBU	CCCCAGGCAATTTAGCTTCT	AGTCTGCATCATAGCCAGGG	187	147	0.95	0	yes	83	4,825,814
A187	EK3X9LI02FRKY4	GATGCTCAGGATTTGAGGGA	CACCCTCTGCATTATTGGCT	180	140	0.98	1			
A188	EK3X9LI01DOVZU	ATTTCTGGCAGTTCCCTGTG	GGAAGAAGTCCAAGCCACAC	184	144	0.94	152			
A189	EK3X9LI02JN1NO	AGCCCTTGGAGCAGTGTTTA	ATCTCCAGACAGGAGTTGCC	140	100	0.97	2			
A190	EK3X9LI01A0OZO	GACCCAAGTGACCCCTCTCT	CATGGAGCTGCAGTGAGTTG	151	111	0.96	1	yes	22	39,379,180
A191	EK3X9LI02ILR5V	AACTGACTGACAGGGTGCG	GGGATGGTCCCATAGATCAC	173	134	0.96	0	yes	14	6,513,068
A192	EK3X9LI01BZ6AP	TATCCCCAATGGTTTGTGCT	TGTTGTTGTTAGTTGCCACAGA	175	133	0.98	7			
A193	EK3X9LI01BF8WX	CCTGCTGTACGAGGCAAGAC	TCAAGATTTTGCCCTCTGCT	157	117	0.97	639			
A194	EK3X9LI02HHLCV	GGGCCTAAGGCTTCTTTCTC	GATTTTGTGGCCAAGGACAG	186	146	0.96	1165			
A195	EK3X9LI01D9AK9	ACTTGTGTCCGGGGATAGC	AGGCCGAAGGAGTAGGTGTT	174	135	0.91	1	yes	3	20,250,612
A196	EK3X9LI02I4J7D	CCCCCTATGGGATACCTTGA	GCAAAAGCTCAGACTCTTTGTAA	139	96	0.95	1	yes		
A197	EK3X9LI01ES0QL	TCCTGATGCTGCATTCTTCTT	CATTCAAACAGAACAACGGG	157	116	0.92	82			
A198	EK3X9LI02GB3Q1	TGCTGCGGTTTTTCTTTCTT	TGTAGGGATGAAAGCCCAAG	179	139	0.96	130			
A199	EK3X9LI02I3BZM	CACAGCTGAGGGAAGAGAGG	CACATGGCCTAACCAAGTTAAT	181	139	0.98	3	yes	9	82,155,052
A200	EK3X9LI02I9BMY	CTCAGCCCAGGAACACATTA	CTCTCAGCCCTCATGGCA	177	139	0.97	1	yes	28	6,496,546
A201	EK3X9LI02J0BXR	TGAAAGAGGTAGAGGCAGGC	TAAAACCACGGACCTTTTGG	153	113	0.98	0	yes	23	11,204,208
A202	EK3X9LI02GX158	GAAAGCAAAGAAGTCACCTTGA	TCTCCAACACCACAATTCAAA	144	101	0.88	1			
A203	EK3X9LI02HICTO	CGGGGTTAGTTCAGGAGG	AACAAGTGAATCGATCACATACAT	179	137	1.00	1288			
A204	EK3X9LI01B6NX6	TGAACATGTAATCAAGATGCATTG	GCCGTTTACGTTGGTGAAAA	187	143	0.87	36			
A205	EK3X9LI01DH4GJ	GGTGATACTCTTGCTAAATAAGTGG	TGCTGTAAATAAAACCATTTCTCTC	162	112	0.96	5	yes	3	24,751,797
A206	EK3X9LI01EYOLN	CGAGGAGACACTGCCATTTT	GTCCCCACGTCCATAAACC	148	109	0.94	1346			
A207	EK3X9LI01AG3ZZ	TGCCATACACTCCTGATTGC	TCTTGGATCAGGTGGACACA	164	124	0.97	6			
A208	EK3X9LI01CGJW5	CACCCTGCAAGAGAGGAGAG	GTGTTTGGACTCCCAGGATG	197	157	0.92	0	yes	8	60,121,961
A209	EK3X9LI02J346Q	AAAGGCTTCTTGGGGTAGGA	TGACAGCATGTTTTCAAGTTTCT	180	137	0.97	6			
A210	EK3X9LI02F0NW3	ATAGGCCCGAGTAGAGGAGC	AATTCTGGCCTGTGGGACT	161	122	0.90	955			
A211	EK3X9LI02HVV0D	CTATCGTGAAGGCTCGCTT	GAGTCCTCCCTTTCACCGAG	171	132	0.93	89			
A212	EK3X9LI02HAW50	AAGTAATGGCATGCGGAATC	AGAAGCCCCTATCCTTCCAA	171	131	0.97	2	yes	43	4,093,612
A213	EK3X9LI02GE17J	AGGCAAGAGATGCTGAGTGG	CACACCCTGGGAAAGGCTAC	144	104	0.97	2182			

Target and primer information for experimental round A.2: read ID corresponds to the read in the short read archive SRA010805, beside primer sequences PCR product length with and without primers is given, as well as the percent identity of the respective mastodon-loxAfr1 alignment, "# of hits isPCR" gives a number how often the respective primer pair anneals to loxAfr1, and a "yes" in the row "consensus for all taxa" means, that sequence information is available for all taxa. The last two rows show scaffold ID and position of the targets used in the MCMCcoal analysis aligned to loxAfr3.

target ID	read ID GenBank SRA010805	F-Primer 5'-3'	R-Primer 5'-3'	product length in bp amplicon	product length in bp w/out primer	Mastodon-loxAfr1 alignment % identity	# of hits isPCR loxAfr1	consen-sus for all taxa	aligned loxAfr3 scaffold ID	loxAfr3 scaffold position
A214	EK3X9LI01A6YMK	GAAAGCCAGGTTCTCAGCAT	TCCATTAACCCCCAAACAGA	161	121	0.97	2	yes	97	479,593
A215	EK3X9LI01DAICP	CCGGCTTAACCAGTAGGTTG	TGTGGCTTTATGCAGATTGG	183	143	0.99	2	yes	110	1,274,053
A216	EK3X9LI01BAUW5	GGATGAATAGGGGCAATCAA	TGAGTTTGCCCTCATCAAAA	177	137	0.92	1	yes	13	16,032,688
A217	EK3X9LI01C10D3	CAGGACTCGCTTTCTCCACT	CACACACCACACGACTGCT	176	137	0.99	1	yes	92	2,467,920
A218	EK3X9LI02JOZ1A	GAGGTCCTCACCACCTCTCA	GGCAGTCACCTCACAGTCCT	182	142	0.92	2			
A219	EK3X9LI01CI39V	CAGCCTTCAGCCCAGTTCTA	ATGGGAGTTGAACCAAGCCT	148	108	0.99	2	yes	84	5,711,232
A220	EK3X9LI01EW96T	ACAGTCGCTTCATCCCTCTG	TAGGCAGGGAATCTTGAGGA	197	157	0.89	1	yes	53	8,525,170
A221	EK3X9LI01AZE43	CCTCCCCTCTCTAAAATCTCC	CAGGAAGACAGAAGGAAAGGAA	151	108	0.98	2			
A222	EK3X9LI02JH9W9	TCCCTCTAGCCAGATGCTGA	AAAGCCTACCAGACCCCATC	168	128	0.96	0	yes	0	59,325,869
A223	EK3X9LI02FUTMC	ACTTGGTGCTCTGTGGGAAA	TGGCCGACACAACTACTCTCT	154	113	0.97	1	yes	7	81,324,383
A224	EK3X9LI01DAMAT	CCAGTTTCCAGTTGTGGTGA	CTCCATAGCAGTACAAAAACTGTC	175	131	0.90	2	yes	30	24,735,311
A225	EK3X9LI02GKBWR	AGGGGCGGCCGAAGGACT	CTGCCCCTGCCCCTACCC	148	112	0.95	1	yes		
A226	EK3X9LI01BJT69	TGGAAGGTGGTGATGACTGA	GAGAGCACCACCCAAGAGTT	160	120	0.99	2			
A227	EK3X9LI01DR5D4	AGGAAGGGCCTGGAAGAATA	GCTGAGTCTGTGCCTTCCTT	170	130	0.99	1	yes		
A228	EK3X9LI01CSRAY	CTTTTGGCATTCCCGAAGTA	CCAACTCCCTCATCTGGTGT	189	149	0.88	1			
A229	EK3X9LI02GJUXQ	GCAAGACCAGTAATAACAGGCA	GAATATGGACTTGGGAGACAGA	196	152	0.98	1	yes	15	50,273,886
A230	EK3X9LI02F7W3P	GACCCCTCCAGGATCTAACG	GCCAGAGAAATGCCCAATTA	187	147	0.96	0			
A231	EK3X9LI02FMHVI	CAGGATCAGGATCAACTTGGA	TAGTACTTGCCCCAAGCAGG	162	121	0.98	1			
A232	EK3X9LI02JG8V9	GTGTCCGCATCATCCCTTAC	CCAATTCCACTTTTGAGCCT	171	131	0.97	1	yes	12	31,489,001
A233	EK3X9LI01C2UN0	GCCTTAGAACCTTTATGACACAAGA	TCCTCTTCACTTCGCAGGAT	172	127	0.96	1	yes	18	26,323,125
A234	EK3X9LI01BQPYQ	TCTTATTCTTCCTCGTCCCG	CAGTTCTGGAGTTTGTGGGG	166	126	0.98	1	yes	28	12,171,137
A235	EK3X9LI01EUZ9D	ACGGGCATGTGTTAGGATTT	GTGGCCTCTAGCTCCACTCA	173	133	0.94	1	yes	25	12,455,405
A236	EK3X9LI01AHPM0	GGACTGGGTGGTGTTCATTC	GAAGCTGTCAAGGATTGACCT	163	122	0.95	1	yes	57	4,986,525
A237	EK3X9LI02H57OX	TGCCAAACCACTGAGAATCA	TCAGGTGGATGTCCCTGAGT	166	126	0.95	2	yes	12	52,770,539
A238	EK3X9LI02ICHTZ	CAGTGGATTTGCAGCCCTAT	TTTCCTAATAATACTCCCACTGCC	151	107	0.98	1	yes	137	501,356
A239	EK3X9LI01AZKE2	CAACAACAACAGCACCAACC	GGTAGCCTGACCTCACACCT	171	131	0.95	2	yes	12	34,447,175
A240	EK3X9LI01BNVRE	CAGATTTGCCCAATGCAAG	TGGATTGCACCTCAACCTAA	168	129	0.94	1			
A241	EK3X9LI01CS96W	TGGAATAAGTGAGCAAGAGCA	GCACTGCAGTAGAAGATGCAA	158	116	0.96	2	yes	5	2,099,580
A242	EK3X9LI01DBT28	GTCATGGTGCTCGGGAAATA	GGACTGCTCCATCCCCAC	168	130	0.90	1			
A243	EK3X9LI02GXGJC	TGGTCACCATACTGCCTTCT	GCCTAGCTAATGATCATCAAATGTT	184	139	0.99	2	yes	2	96,260,218
A244	EK3X9LI01C5BXG	GAGATGATCAGCCAGGGTTC	AAGGTGAAAATTCATTAATTCCAGA	171	126	0.98	1	yes	31	3,190,302
A245	EK3X9LI02HHH91	CCCTCTGTGATGTGCAGTGT	TGTCCAGTTCGTCTGGGAAC	201	161	0.93	1	yes	55	10,557,275
A246	EK3X9LI01CUAQF	ACCATTTCCTGTTTCCGTCA	CCCTGAGTGGTGCAAAAAGT	200	160	0.96	2	yes	101	4,362,706
A247	EK3X9LI02HHXNO	AGGCATCCAGAATGCTCTC	AGTGAAGCCTGTGCTGGG	142	105	0.98	2	yes	12	20,904,563
A248	EK3X9LI01C88FU	ACCAAACCCATTGCCATCTA	AGTGGTTAAGAGCTCGGCTG	151	111	0.96	0			
A249	EK3X9LI01C4JXA	TAGCAGCTTTTGGTTTGCCT	ACAAGCACACTGAGAGATGTGT	182	140	0.97	2	yes	97	2,757,461
A250	EK3X9LI02HQGIE	CCTTCAGAACGCCTATGAAA	ATCTTATTCCGATGGGGGAG	163	123	0.97	1			
A251	EK3X9LI01EVMCB	GTCCGTTCTGACTCATGGTG	AGTCCCTAGATTGCCTGGGT	197	157	0.99	1	yes		
A252	EK3X9LI02IUY0W	AACAACCAGGGTAGCCACAA	TTCTCCTCTCTAGGGGGCAT	159	119	0.94	1	yes	19	9,704,021
A253	EK3X9LI01DMCVW	AGACCACAGCCTTCAGTATGG	TTTCTTGACTGCTGCTTCCA	167	126	0.99	0			
A254	EK3X9LI01DE3SN	GGACTTACAGCAGTTAATTCAGGA	ACGTTTGGGACTGCAGCATA	167	123	0.94	1	yes	68	3,515,241
A255	EK3X9LI01E3ANB	ACTTGGGGACCCCTGTTTAG	CTGGCCAGAAAGGAGGACTA	168	128	0.98	1	yes	0	90,319,470
A256	EK3X9LI02HCP5E	AGGGAACTGCCAGAAATTCA	GATCTGCACAGTCAAATGCC	150	110	0.94	0			
A257	EK3X9LI02IXJ6P	TATAGCTTGGGGGCTTTCAG	TGCAGAGGAGAAACAGTTTGG	177	136	0.98	1	yes	6	19,705,800
A258	EK3X9LI01B5YLH	TAAGTCAGGCCTCCTTTGG	CTGATTTCAAAAGCCAAGCA	178	139	0.96	1	yes	18	25,385,818
A259	EK3X9LI01BYU3U	AACCACTATGCCACCAGGTT	CTGGGAGTCTAACCCACCAC	189	149	0.95	0	yes	8	62,591,951
A260	EK3X9LI01B2CXX	GTATGATGTCCTTCGCCAGG	TTTGAATTGTGTTGTTGGCG	180	140	0.92	2			
A261	EK3X9LI01CO4UJ	CTCGAGGGCTCGGCTCGG	GTGTGCTCCGCAGGATGGG	188	151	0.96	0			
A262	EK3X9LI01D78CW	GCTAATCGCAAATACGTAAGCA	CCAAATCAATGATATCCACTCC	186	142	0.97	1	yes	4	100,302,354
A263	EK3X9LI01A36MQ	TGGATACCAGAGGTGTCTTCAA	TGAGGCAGAAAAACATTCCC	190	148	0.89	2			
A264	EK3X9LI01C31CQ	TGTGGTAGCATGGTCTTTATGG	CATTCAGAAACTGAAAGACTGGT	172	127	0.98	1	yes		
A265	EK3X9LI01AXUGS	GGTTGAACCTGAATGAGCGT	GCAGCCTCCTGGAGTTCCT	166	127	0.97	1	yes		
A266	EK3X9LI02IP8OQ	GCCTCATGGGAACTGAAAAC	ATGGATCAAAGGTGACCAGG	155	115	0.96	1	yes	16	15,855,345
A267	EK3X9LI01DW9XM	AAGCAATGTCTTACTACCTGGTGA	TGCAACAAGCAACAACATGA	138	94	0.96	0	yes	14	50,048,147
A268	EK3X9LI01BA2KU	CTTTTCTCTGAGCCTAGCCC	TCCACCATCACTTCAAATGG	137	97	0.97	2	yes	11	27,188,476
A269	EK3X9LI01EQMZH	ATGAGCTGAGCTGGAAGCAT	GGAAGGACTCTCGTTTCCTG	136	96	0.99	1	yes		
A270	EK3X9LI02GJE0M	TTTCTGCGGCTTCTCCTAGT	TGCAGGGTGACATTTAACGA	154	114	0.98	0	yes		
A271	EK3X9LI01AG73T	TCTCAGTGTAGGGACCCTGG	CCTTCCAACGGCATCATTTA	156	116	0.91	1	yes	4	89,117,088
A272	EK3X9LI01DSCFL	TCCGGTACAGGCTTCTATGG	TGTGTGTCAGAGTAGACTGGGTG	157	114	0.98	1	yes	11	9,150,261
A273	EK3X9LI01E0MTC	AAGTGGCAGCATCGAGAGAC	CTCTGCCCACAAGTGCCT	184	146	0.95	0	yes	45	4,466,640
A274	EK3X9LI02HISXL	CTTCCCTCAGAGGAATAACCA	CTTCCTGTCAGCAACTGCCT	203	162	0.95	1			
A275	EK3X9LI01EMD03	AGTAGGACAGTGTGTGGGGC	CTCTCGTGGTGAGGGCAC	174	136	0.96	1			
A276	EK3X9LI01C612J	GCACCCTGGGGAAGGTATAG	CTGCCTGAGATTTACTGCGA	171	131	0.98	0			
A277	EK3X9LI02F2KAX	AGGTGCCTGCATCTGAGC	GACAGCGGCAGACACAGT	170	134	0.93	0	yes	5	79,715,844
A278	EK3X9LI02GYCYN	TTTGTGTACCTGCAAGGTCG	TTTTCTGTATGTCATATATGCTGCT	156	111	0.94	1	yes	23	18,612,615
A279	EK3X9LI01BL8TH	CTCCAATCAACAGTGACCCC	CCACCGGAGGCAGATAGG	142	104	0.96	0	yes	8	78,972,721
A280	EK3X9LI02IM216	TACTGATTGGAAAAAGGGGG	ATGGCGAGAGCCAATTACAG	159	119	0.92	0	yes		
A281	EK3X9LI02JRGTA	AGGAAGAAAGGCCTGGTGAT	GCTGCTGCCTAGTCACTCCT	164	124	0.98	0			
A282	EK3X9LI02IDB6R	GGCTGAGGTCCTCTTGTTGT	TTTTTCTTCCTTATCCCTTGGA	170	128	0.96	1	yes	18	34,832,978
A283	EK3X9LI02GUAC5	ACGCCTGGAGACAGACAGTT	TGCACACCTTGAAGCATCTC	159	119	0.98	0	yes	90	5,612,392
A284	EK3X9LI01E0SQ3	TCAAAGACCACAGCCTTCAG	GCGTCTTTCGGTTTCTTGAC	181	141	0.89	0			
A285	EK3X9LI02HCAWO	AAATGACGAAAAGTTTGGCCT	TAAAACCTTTCCTGTTGGGG	152	111	0.99	1	yes	27	33,846,061
A286	EK3X9LI01APTF1	TCTTCTCCACATGCCTTTTT	GCAGCATTCTTGAGGTGACA	175	135	0.87	1	yes	14	51,320,467
A287	EK3X9LI01DKQC1	CAGTCTTCCCTGTGTCCTGG	GCTGTAGAGTGCTTGGCACA	190	150	0.97	0			
A288	EK3X9LI02I5B92	GTGGAGCGCGTTCTCTGA	ACGCGATGAGTTATGGTGGT	186	148	0.99	1			
A289	EK3X9LI02H7DWQ	CCAGGATGACTTTAAGGGATTTT	TGGTAAGGTGTGAAGTGGGAG	182	138	0.99	1	yes	2	91,796,234
A290	EK3X9LI01EL68Y	GGGTACAGAGCCTTACCGATG	GTAGGCCCCTCCAGCACC	179	140	0.92	0			
A291	EK3X9LI02FGJW8	CAGGAATCCAGGCAGCTAAA	GGTGTGAGCACCAACTGAGA	185	145	0.96	1	yes	26	25,202,384
A292	EK3X9LI01CEPH4	TGGCCACAGGTATGACTCAG	GACGAGCATCTTGCACCAC	186	147	0.96	1			
A293	EK3X9LI01ESOP1	CACTCCTGGCTCCTGCTG	CCCTCTTGGCACCACTTAAA	176	138	0.99	1			
A294	EK3X9LI01EFXA0	CCTGGCTCTGAGTAGAGGATTT	CCTGACCTTCAAAAATCACCA	200	157	0.98	0	yes	31	2,675,757
A295	EK3X9LI02IW1WN	GACTTGCTGGCATTTCCCT	AGGGTGCACCTTCAGGAGT	196	158	0.94	1			
A296	EK3X9LI02HERSZ	TATCTTTCCTGGCCCTTCCT	TGGAATAGACCCCACTCCTG	175	135	0.97	1			
A297	EK3X9LI01DZ6IC	TGGTTTGCAACAAGCCATTA	ACCATATGGGGAAGGATGGT	150	110	0.87	1			
A298	EK3X9LI01EKU3Q	GTGGGAAACGTTAGTGGTGG	ACCGAGCCATAATGCTGC	174	136	1.00	0			
A299	EK3X9LI01EGAXM	ACCCTCCTCACCAACCCT	ACACTGGCTGAAAGACCCAG	165	127	0.95	1	yes	8	44,369,514
A300	EK3X9LI02IFNCG	CCTCTCCGTGATGAGCCTAC	CCCCATGTTATGAATGAGGAA	157	116	0.96	0	yes	48	1,875,618
A301	EK3X9LI01DWCAZ	GATCTACTCTTGGTTATAAGCCTGC	CCACACAAATGATGCCTGAA	165	120	0.96	1	yes		
A302	EK3X9LI02G0MB9	TCTGGCCTTAGGTGCTTTGT	AAAGGGTCGCCAGTTCAAAT	165	125	0.95	0	yes	96	1,758,331

target ID	read ID GenBank SRA010805	F-Primer 5'-3'	R-Primer 5'-3'	product length in bp amplicon	product length in bp w/out primer	Mastodon-loxAfr1 alignment % identity	# of hits isPCR loxAfr1	consen-sus for all taxa	aligned loxAfr3 scaffold ID	loxAfr3 scaffold position
A303	EK3X9LI02GXPG5	GGTGCACCTTAGTCTTCAAGG	GCAACATCATGATAAATGGAGAA	189	145	0.92	0			
A304	EK3X9LI01AP2KK	ACACGTCTTCGAGTGCAGAG	AAATATAACCCCCACGAGCC	178	138	0.93	1	yes	2	67,326,190
A305	EK3X9LI02F2WI4	ACCTTCCCAGGAACTTGGAT	CGGGCCAGACTAGGTAAGGT	155	115	0.95	1	yes	31	11,774,220
A306	EK3X9LI01B6D8U	TGGAATCCAGTGGAAAGGAG	TGAGTTTGCCCAGTAGCCTT	166	126	0.95	1	yes	55	11,051,588
A307	EK3X9LI02JVNVQ	TCCCTGGCTTACCAGATCAT	CTGGGTAATCCGTCCATGTT	174	134	0.98	1			
A308	EK3X9LI02H2Y5V	TGTCCTGACCTCCTCCAGTT	TATATTTTACCGCACCCCCG	177	137	0.94	0			
A309	EK3X9LI01BVTSL	TTCCAACTCATAGCAACCCA	CTGTGATGTTTCTGGAGCCC	185	145	0.92	0	yes	17	30,851,947
A310	EK3X9LI02HEM7N	GTCCTCTCCTACCCCTGCTG	AGGCCCAGGGGTACAACTTA	188	148	0.91	1			
A311	EK3X9LI02JSHAS	CCTTTGAAGGGTGGCTAATG	AGAAAATGTGAGCGCAGGAG	156	116	0.98	0	yes		
A312	EK3X9LI01BATY4	GTCATGGGCAGAGAGGTCAC	CTGCCCGTGCTCTCCACT	164	126	0.99	1	yes	5	11,867,225
A313	EK3X9LI01D26AB	AAAGCCCAATAGATTAGCATTTG	ACCACCCAGATCTAGAGCCC	147	104	0.98	1	yes	13	9,741,987
A314	EK3X9LI01C5EIW	GGGTTACCCTCTATGGCCTC	GGAGGTGGGACTCAGAGGA	152	113	0.98	0	yes	19	37,637,919
A315	EK3X9LI02I7G1A	ATCTCTGAGTCCTCCAGGGG	AACATCAGTTCAGGGCACAA	185	145	0.96	0	yes	3	2,970,325
A316	EK3X9LI01B1O1E	AAGCAGAAAAGGTGTCAGGG	TCCAAGTTCTCATTACTTTCAGCA	175	131	0.98	0			
A317	EK3X9LI02H6HIK	CATGAAGTGGGAGGCTGAAC	CAGCTCGTTTCTTGCTCCTC	168	128	0.96	1	yes	123	1,762,385
A318	EK3X9LI02JWOZV	AAACCCTGGTGGTGTAGTGG	TCTAAAAACTCAACTTAGTGCCG	164	121	0.96	0			
A319	EK3X9LI02GZSQ8	CCCGGTTGGTTATGGTAATG	CCAGCACCCAAGGGTAAGT	170	131	0.98	1	yes		
A320	EK3X9LI02JNZU2	TGGAACCAGCTGAACTAGGG	AGGCTCCCCCTATTTTTCAA	144	104	0.98	1	yes	114	1,404,004
A321	EK3X9LI01BN7F6	GAGATTCACCCTTCTCCTGCT	GAGCTGGGAGGTGCAGAT	164	125	0.95	1	yes	16	34,910,293
A322	EK3X9LI02HJXEE	TTGGGCATGAAGGCTATTGT	GCTCTGCCTCATTCCTGAAC	198	158	0.96	1			
A323	EK3X9LI02IHQWE	AAGAAAGCCTGTCACCCTCC	TCCAGCCAGGAGTAGTAGCC	180	140	0.98	1			
A324	EK3X9LI01C3BAK	AAATTGAGAAGCCAAGGCAA	TCATCTGGGACATTGTAGCG	180	140	0.99	1	yes	13	28,007,158
A325	EK3X9LI01BJ5ZM	AGGGGCTAACCATGTCTCCT	GCAGAAGGCAGGTAGGTGG	171	132	0.88	1			
A326	EK3X9LI02I1MLE	GCATGTCTTGAGTAACTCTTTTGC	CCACTGTGCACAAGTCACCT	189	145	0.94	1	yes	73	4,835,113
A327	EK3X9LI01A6DKU	ATCAAGAGGCAGTTGTTCGG	TCTTCCTCCAATCCTGATGC	173	133	0.94	0			
A328	EK3X9LI02I1GYF	TCCTCGCAAGTAAAGGGAAA	GCAGATAAGCAGCCTGTTCC	167	127	0.95	0	yes	132	1,763,628
A329	EK3X9LI02HAYZJ	CTCCTTGCCGGGCTTCAC	CTTCTTCTCCGGCCTCCTC	151	114	0.90	1			
A330	EK3X9LI02HO6PN	ATGGTAGGCCACTGATGACC	ATTCTGGTTTCATTTCCCCC	165	125	0.98	0	yes	140	582,722
A331	EK3X9LI02GJELN	GCAAATGAAATGAGCACTTTG	CTCAAATCAACCCCATGTGA	180	139	0.99	1	yes	0	64,893,892
A332	EK3X9LI01DT9PM	TGAAGGCCTCCAGTATCACA	AATGACACCTTGGGCCTGTA	170	130	0.98	0	yes	6	64,713,931
A333	EK3X9LI01ATT8N	TTTTTGAGGCTTATTGACGGTT	CTGTTTCTTCCCATCCTCCA	154	112	0.98	0	yes	102	646,152
A334	EK3X9LI02JCVGO	CTGGAGAGAACCAGAGCCAC	GCCTCTTGAACTTCTTCCTCA	191	150	0.95	1	yes	48	17,101,706
A335	EK3X9LI02J1RUJ	CTTCGCCAACACTGCATAAA	CACTGCACATCAGACAGGCT	150	110	0.98	1	yes		
A336	EK3X9LI02GKDNX	CGAGCAGTACGAAACAGCCT	GCCCTGCCTCATGACATAAT	171	131	0.95	1	yes		
A337	EK3X9LI01D9NMD	AGAAGCTACTGTTGGGCTGC	TCTCTCTTTCCAGCAGTGCC	152	112	0.99	1	yes		
A338	EK3X9LI01C3W4S	AGCCTGTTGACAGCTGGAAT	TGGATATGTATTAACAGGCAGAAAA	188	143	0.96	1	yes	5	66,366,555
A339	EK3X9LI02FW3PT	AGCGACACATCTGTTAAGCG	TGATTCACACCCAAACTCCA	161	121	0.97	1	yes	11	10,327,481
A340	EK3X9LI02HFC44	TGACCCAAGCCATGGTATTT	CCTCTAAGGAGCATTCTGGC	189	149	0.93	0			
A341	EK3X9LI02I1H3N	TAGCCGTATTCTGGGAGTGG	CTGTGCGCTCTCTATTTGGA	172	132	0.94	0			
A342	EK3X9LI01DZ625	TCTGCTAACCAAAAGGTCGG	GACCCCAGGCTTTGCTATTT	177	137	0.95	0	yes	49	3,448,120
A343	EK3X9LI01CV1K0	GGCCCCTAGGTACTTTCCTATT	CACAGTTCAGATCCCCCAAG	198	156	0.96	1	yes	33	23,320,683
A344	EK3X9LI01BQZYB	CTTTCAATTCATGGCAGCCT	CTTGCCTATTGCATTCCACA	171	131	0.99	1	yes	11	21,526,888
A345	EK3X9LI01AHLK0	AGTCATTTTCACTCGCGCTC	TTTCTGGCCTTTGGAATTTG	172	132	0.94	1	yes		
A346	EK3X9LI01CGF48	CCCTGGTGGCATAGAGGTTA	AACCAAACCAAAGTCGTTGC	160	120	0.94	0			
A347	EK3X9LI02HHBR7	GAGGGCTTTTTCATTACACAGG	TGGTGGTGCAGTGGTTAAGA	182	140	0.94	0	yes	71	5,176,199
A348	EK3X9LI02I5TH7	CAAGAAATCAAAAGACGCATTG	TTCCTTATTCATTGTCCAGCTT	167	123	0.97	0			
A349	EK3X9LI01AGNDJ	TGGCGTAGTGGTTAAGTGCT	CAAACAAAATGAACCAGGGC	192	152	0.91	0			
A350	EK3X9LI01DOOZ2	CTGGAATTCCCATTCTTTGC	ATTGCTGATGACGGATGGAT	142	102	0.96	0			
A351	EK3X9LI01C4567	TAGCACAACAACCAACCCAA	AAAAAGCCCTGCAATCATTT	185	145	0.97	0	yes		
A352	EK3X9LI01CSK5F	ACCATGGACTGCCAAAAGAG	GGCCCTCTGCTTTACCAAG	161	122	0.95	0			
A353	EK3X9LI01EEV1K	GGGGGATTCAGAAAAACAGG	GGATCTGACCACGGCTTTAG	152	112	0.96	7			
A354	EK3X9LI02ICLCR	CGGCCATTTTTGAATGATCT	TGCCAAGAACTTTGGAAGACA	166	125	0.93	7			
A355	EK3X9LI01DY4ZE	AGCCCAAGGGTAAGCAGAAT	CATCTTTGTTTCTCTCGGCAG	149	108	0.99	2	yes	19	44,681,437
A356	EK3X9LI01AOG35	TATCTTGGGCAGGAAGTTGG	GAAAAGAGAAGCAGGGAATGA	146	105	0.97	2			
A357	EK3X9LI02GVD2Y	ACCATCCACACATTCACCCT	CCATTCCTCACCCTCACATT	164	124	0.97	2	yes	4	201,844
A358	EK3X9LI01BZFRW	AGCCTTCAGGTGCTTCTGC	CATTTGCAAAATGGACACCTT	166	126	0.92	2	yes	72	7,242,987
A359	EK3X9LI02JGWHQ	ATACGCCTGAACGGAGATTG	GGGCCTCTACTTTCCTGCAC	167	127	0.97	14			
A360	EK3X9LI01CSTA6	GGTAGCACTTCACCTCACCG	GCAGCCCCTCCTGTAATTTT	166	126	0.98	2	yes	9	72,773,335
A361	EK3X9LI02F2DRH	AACACACCATCACAGCAACC	GAGTCAGGCTGGAGATTTGG	194	154	0.98	4	yes		
A362	EK3X9LI01CT3DB	TACCAGGGACTGCCAAAAGA	GCATGGTATCCTTCTTCAGGG	146	105	0.96	6			
A363	EK3X9LI01AUJL8	GGTGTTGCTCTGATGCTGAA	AATGGAATGAAACACTGCCC	168	128	0.97	5			
A364	EK3X9LI02H1MD3	TGTATTTCCATGGTGCCTCC	TGTCCAAACAAGAACAGCCA	151	111	0.95	2	yes	18	474,687
A365	EK3X9LI01ES7D3	CTCGGGAAGCAAATATCTCG	TGTTTGTTGTTCAGGGTTCG	196	156	0.95	6			
A366	EK3X9LI02FHQAQ	GGACCCAGCCTGTCAGATAA	TTGGTGGGGCTTTCAAAAT	201	162	0.93	2	yes	58	7,346,355
A367	EK3X9LI02I6LIN	ACTGCCAGAGAGCCCAGTAA	GTCCTTTTCCCCACCATTCT	173	133	0.97	2			
A368	EK3X9LI02JX8L2	GGAAACGCAGTCTGTTGTCA	GATTAAAAGCATATGTTCCCCAA	165	122	0.97	2	yes	8	76,341,113
A369	EK3X9LI02GWT1S	GACCTCTCCTGTTGGCTCAG	GTCATTGGCCGACATTCTTT	191	151	0.96	23			
A370	EK3X9LI02JBOQT	CAACATTCAGCCATTTCAGC	AGTAAGCATTTCCAGTGCCG	156	116	0.90	9			
A371	EK3X9LI02GSG4U	CCTACCCAACTGACTGGAAGA	TTTCTGGGAGTTCCTGGTTG	171	130	0.91	13			
A372	EK3X9LI01DD81C	TGTGTCGATGCAACTACAAGG	AGCGGCCAAGAAAACAACA	155	115	0.98	17			
A373	EK3X9LI02GJN13	GGCCTGTGACTCAGCACTTT	TGCACCACCATTTCCATTTT	191	151	0.94	2	yes	57	5,155,828
A374	EK3X9LI02HM0KE	TGCAGTCTTCCAGCAACATC	TGGCCTTGGAGAAGGAATAA	189	149	0.92	6			
A375	EK3X9LI01CNIJ4	ACCCTCGACTTTACCAAGCA	CCATGAACTGCCAAAAGAAA	167	127	0.97	2			
A376	EK3X9LI01DYFL4	TGAAATGTGCAAGAACCTGG	CAATGGCTCTGTGTGTTCCTT	185	144	0.97	7			
A377	EK3X9LI01DFG2C	GCGGAAGATTGATGACAAGG	TGCTGCTATAACAGAAATACCACAC	159	114	0.88	5			
A378	EK3X9LI02F97QQ	AGCCTACAGCGACTTCGG	GTAGGCTGACAGACCTGGCT	183	145	0.92	8			
A379	EK3X9LI02JNZE5	CCTTTGAATAATGGTGTTGGC	TTCCTTCTGATAACATGTCCAAA	157	113	0.98	11			
A380	EK3X9LI02IIZCH	TCTCTGGCACTTCAAAAGGC	TGATGGAGAAAAGGGGTCTG	169	129	0.99	2	yes	186	543,457
A381	EK3X9LI01CQR52	CGAGACTTCGTCTTGCACAC	AATGAAACACTGCCCAGTCC	166	126	0.94	9	yes	12	1,357,538
A382	EK3X9LI02HT88N	GGCTGTTGACTGCTGTTGAA	TTGGGGTTAAACACTTCCTGA	154	113	0.95	1	yes		
A383	EK3X9LI01DKL12	TGACAAGCTAGGAGCTCTGAA	CAGGGGATTGTGCCTGAG	144	105	0.96	5			
A384	EK3X9LI02G2N3R	TTGAGCACCTTTCCCTTTTG	TGAGTAGGTGGGGACCTTG	156	117	0.94	6			
A385	EK3X9LI02I70NA	TCCACACTTATCCACAGGGG	TAGAAGCTGAGGGGGAGGTT	136	96	0.96	1	yes	146	1,412,413
A386	EK3X9LI01BNWGF	CCCACTTTCACAGGCTAGGA	TTTGCTTTTAATAACCGACCAGA	192	149	0.98	1	yes	74	2,692,157
A387	EK3X9LI01EVFH1	CAAGTTGTTCCCTTTGTTCTGA	CCACTGATTGTGATTGACTCG	167	124	0.89	1	yes	10	24,477,942
A388	EK3X9LI02INT62	TGGTAAGCCACCTGCTTCAT	ATTCTGACCTGGGCCATTCT	168	128	0.96	2	yes	41	6,205,165
A389	EK3X9LI01EW5JW	GGCCGCAATAGTTGACATTC	ATGTGGCTGTCTGTAACCCC	144	104	0.98	2	yes		
A390	EK3X9LI02FEYRB	CTCACGTGCCAGGAAATAGG	TTCTGCATCTTTTACGCGCT	192	152	0.99	1	yes	39	17,610,294
A391	EK3X9LI01BR66V	GACAAGGGAAAACCTCTGGG	CAAGTTTTCTGGGAGCTGGA	170	130	0.98	1			
A392	EK3X9LI01DS7ZY	ATTGGACGAGACAATGGGAT	TACCCCTAACTGCTCTCCCC	204	164	0.89	2			
A393	EK3X9LI01EGYU4	GATTGTGTGGCTCCCAATGT	ATGGCTCAGATCCTTGCAGT	176	136	0.94	1	yes	74	4,368,719
A394	EK3X9LI02I8NU3	AACCAAACCTGTTTCCGTTG	ACCCTGGTGGCACAGTAGTT	155	115	0.97	8			
A395	EK3X9LI01BROUI	ACAATTGTGAGGATGGCACA	TGTGGCTTGGGTTGTTGTTA	157	117	0.91	10			
A396	EK3X9LI02H9XCH	GTTCCACACATCAAAAGGGG	TGGAGTGAAAAAGACACATGG	162	121	0.97	2	yes	15	33,021,381
A397	EK3X9LI02I4R9E	GGAAATGCTCTTTGCCAACT	AGGTTGAGACCAACTGATGC	185	145	0.97	2			
A398	EK3X9LI01EJ2CR	CAGAAATACCCACCATGCTCA	GCCTGGGTCTGCTCCCTC	179	140	0.93	1	yes	15	22,588,500

A.2

target ID	read ID GenBank SRA010805	F-Primer 5'-3'	R-Primer 5'-3'	product length in bp amplicon	product length in bp w/out primer	Mastodon-loxAfr1 alignment % identity	# of hits isPCR loxAfr1	consen-sus for all taxa	aligned loxAfr3 scaffold ID	loxAfr3 scaffold position
A399	EK3X9LI01EU2OA	GACTAAATGGGCACACCAGC	TCCATTTCATTTTTGACAACTTTC	168	124	0.97	7	yes	18	2,631,071
A400	EK3X9LI02ISJEZ	CTCCCAGCCCCCACTTTT	GGTCTTGGAGGAAGGAAAGC	192	154	0.98	4			
A401	EK3X9LI02FGAXJ	TCCAGAAGGGACATGGTAATG	CTCCTGAAGCCGTGTCTCTC	176	135	0.95	1	yes	4	66,397,755
A402	EK3X9LI02HLL0U	GTGAAAAGCTGCATGCTGAA	CCAAGGTTACTCCAGGGGTT	163	123	0.91	7			
A403	EK3X9LI02HN8L1	AAGGTGACCCCTATGTAAGGC	TGCAATTTAGGCTGTCACTTG	182	140	0.97	1			
A404	EK3X9LI02GSBFJ	AGTGGAATGTGGTGGGTGAT	TCCTGGGTTTATTTGGGTTG	144	104	0.99	2	yes		
A405	EK3X9LI01CSU1O	TCCAGAGCCAACACAGACAG	TCATTCTCTGTTCTCTGTCACTTTC	177	132	0.98	19	yes	14	2,295,033
A406	EK3X9LI02HCWKX	ACTCCTGACTGTGATGGCCT	TGTGCAGAAGGACAACTTCAA	172	131	0.98	3			
A407	EK3X9LI01BR7SA	TGCTCCTCCAACCTCAGAGT	ATCTAGCCCTGGCAGCAGT	148	109	0.90	2	yes	4	25,548,875
A408	EK3X9LI01CG92M	TCCTAGGGCCCTGACTGAG	GAGGTGTCACTGAGGCCG	177	140	0.99	1			
A409	EK3X9LI02I4LVA	CACGTCACATCCACCCTCT	CGAGTGGTGGTGACAGAGAA	175	136	0.98	1	yes	1	55,090,741
A410	EK3X9LI02I6U6P	TAGTTCTTCCTGTCCCCAGC	TGGAAGCATGATGTAGCAGC	174	134	0.98	1	yes	1	26,114,494
A411	EK3X9LI01DY40E	GAAAAGTTTCCTCGCACAAAA	TATGGCTTTGCCAGGATTTC	143	102	0.99	1			
A412	EK3X9LI01D7D35	GAAATAAGCTGATGAGAGGAAACC	GGACAGAGAGCCAACCACAG	181	137	0.96	1	yes	5	71,661,785
A413	EK3X9LI02HY1W8	GCCTGTCTCAAAAGGCTCAT	AGAAGCAGAAAGCTGGCAGT	172	132	0.93	24			
A414	EK3X9LI01CR2K5	TGAGAAATTGTGGGATAAAGGG	AGCCACTCTAACATCACTGGG	167	124	0.96	1	yes	43	7,175,416
A415	EK3X9LI02FZ53G	TACCCAATGAGTCAGCAGCC	GCAGATCCCAAGAACGTAGTG	146	105	0.92	6	yes	22	13,947,214
A416	EK3X9LI02F0KPQ	ACAAATCAACTCTGTCTCTCACAT	TGTTTGCATCTCCTGTCTGG	186	142	0.96	10			
A417	EK3X9LI02FK9G5	CCCTCTGCACAGCTGAAAC	CTGGAAACAGACCAGAAGCG	176	137	0.88	4			
A418	EK3X9LI02ICEX4	AGATGAGCTCCCCAGGCT	TGAATGTTTGCTGCTATTGCTT	158	118	0.97	3	yes	6	46,290,310
A419	EK3X9LI01AXH9B	ATCGTTGGTGGCTATTGTGC	CCTGATGGATCAGAACAAAACA	165	123	0.95	3			
A420	EK3X9LI01CN0BK	GCAGGGAGCATCAAAAGAAG	TTTTTGCCAAAGCCTTCAGT	153	113	0.92	3			
A421	EK3X9LI02F7KM5	CACATGGCTATGCCAACAAA	TTGCTTTTGAATGATCTCCAG	172	131	0.92	5	yes	72	2,595,307
A422	EK3X9LI01CWGJL	CTTCTCTGGAGCTCTGGTGC	AAATCCGTTGCTGCTGAGTC	161	121	0.87	7			
A423	EK3X9LI02FQPRM	CCACAAAGTTGCCCAAATATC	CAGGCAAGAAAGAAAGACCTTG	189	146	0.97	3	yes	9	16,167,935
A424	EK3X9LI01D0RFU	GACCAAGGAAAGGTCATGGA	CCACTTTAAGACCCCAGACG	182	142	0.94	4			
A425	EK3X9LI01BLD35	GTCAGCTACAGCGACTTCGG	GACCTGGCTGGTGGGGTC	174	136	0.89	8			
A426	EK3X9LI01ANCQO	ACTGGCTGATTCTTTTCGGA	CCCCCACATGTCTGTCAGTT	175	135	0.90	16			
A427	EK3X9LI02FKADD	AAGAATTCTCCATCATCACGTT	GAGCCTTCAGAGCTGGTTGT	197	155	0.99	13			
A428	EK3X9LI02JA4H2	GGGAGAGAACACGCAAAGTC	GCAGACTCTGGACTGCTGG	152	113	0.92	5			
A429	EK3X9LI02I2ZDJ	GGAAGGTCAGCCTAATTGGA	TGGGATGTGGAATGTTTTCA	166	126	0.98	21			
A430	EK3X9LI02ISJ77	GCTGACACTCTGTCTTATCAAGC	CCATGGACTGCCAAGAGAAT	165	122	0.97	3			
A431	EK3X9LI02JHJB0	TCATTCCAACAGAACAAGAGGA	TGCAGGTTGTTAATATGTCATCC	156	111	0.98	26	yes	1	93,100,472
A432	EK3X9LI02H27IT	TGTCAGGGTCAACACCAGTC	CTTTCTTTGCTGGGGAGGT	143	104	0.98	3	yes	15	49,396,357
A433	EK3X9LI02H51BQ	GCGCACCTTAGTCTTCAAGG	CGAAGAAAAGATTGAAGTTGTCA	172	129	0.91	5			
A434	EK3X9LI02HK8QF	GGAAGAGGTCCAAACTGCAC	ATTTCTTCCAGCTGGTTGGC	167	127	0.94	3			
A435	EK3X9LI01C7EDZ	TGATACTGGTGAGGAAATCTTGAA	CATGGTATATTCATTACAGGGCA	179	132	0.91	2	yes	88	692,684
A436	EK3X9LI01EG2O2	TATGTACTGGAGCCCTGGTG	TACCCAACCCATTGTCATCC	166	126	0.88	7			
A437	EK3X9LI02HV0XV	TAAACACTAGCAAGGGGCCA	TCTGTTGTACCCAGGCATCA	181	141	0.94	2			
A438	EK3X9LI02JXGA3	ATAGGGACCATTTCCTTGGC	AAAGTGGCCATGATTTGGAG	166	126	0.94	2	yes	81	531,445
A439	EK3X9LI01A679X	TGGATTCCACAGAAAAAGCA	AATCCTCCAACTCACAGGCT	179	139	0.98	3	yes	43	2,511,708
A440	EK3X9LI01BCLJT	TGGGTGACATCCAGCAAGT	TGTCCAAAACACTGATTAATAACAA	191	147	0.96	6			
A441	EK3X9LI02GSTFP	CAGAGGGAAGGTTGAAGGAA	GGACAAATCACTTGGCTGCT	180	140	0.92	2			
A442	EK3X9LI02HBFQX	TGTTCTTACTCTGTCTTAGCTCCA	TGGTTGATTGCTCTTAGAGTGTG	173	126	0.92	3	yes	23	4,746,893
A443	EK3X9LI01B6FSD	TCTTTTTACTTTCTCCAGTAAACCA	ATCTGAGGGCTGAGCTGCTA	152	107	0.96	3	yes		
A444	EK3X9LI02GB2O7	TGGAGTAAATTTCCCAGCAG	CCTACAGCTCCATTTTCCCA	145	105	0.87	2			
A445	EK3X9LI01AXKEV	AGGCAACAAGGACAAAGCTAA	AAAGCAAACAAAATGTGGGG	154	113	0.98	3	yes	54	1,474,214
A446	EK3X9LI01AT135	TAGCGTTTGTTCTTGGACCC	CCTGAAGCCTAACTTCAACTGT	175	133	0.93	4			
A447	EK3X9LI02G13TL	TTTCACCCACATGAACAGTGA	GCCTTTGAGCAGTTGATGGT	165	124	0.91	2			
A448	EK3X9LI01EV5K6	CATTGGTCCAGTTGTGAGGA	GGCATCAGCATTGGAAGAAG	177	137	0.98	2			
A449	EK3X9LI01BMRPD	CTGCTAGTGAGGGAAGGCAC	GTAAATGACCTCAGCCGCTT	180	140	0.98	3			
A450	EK3X9LI02I4W2R	GCAGGCCTACATTTAAAGTTCA	AAGGGTCCACAACTGAATGC	148	106	0.99	2	yes	87	1,987,911
A451	EK3X9LI01DTYJ5	AGCACTTGGAATTCCCTTCA	TTCAAGTGAAAGGAAGGGTCTC	176	134	0.91	5	yes		
A452	EK3X9LI02HI63G	GGGTCCCCTGGTGATGTAG	TCTTCCTTCTCCCCCTGAGT	183	144	0.99	26			
A453	EK3X9LI02HIM9M	TGTGCCATCCTCACAATCAT	GAATGCACTTTAGAAGCGAGG	184	143	0.97	10			
A454	EK3X9LI01CFAU6	TGTATGGTTCTTGCTGGACG	TACATTAAAACTACTGTCACAGGGA	181	136	0.95	2	yes	1	53,667,867
A455	EK3X9LI01BU2XL	GGGGTTTACAAGTAGGAAGTTCA	CCATGCTGACATTCAGGATAAC	187	142	0.99	26			
A456	EK3X9LI02JBDAS	TCTCTGATCTGTGCTACTAAATTGG	AACCCCCAAATTGATCCAAG	181	136	0.96	2	yes	59	4,864,244
A457	EK3X9LI02FUTXB	AAAACATAGTGAGCTTCCCTGA	ACCATGAAACTGTGGCCTCT	171	129	0.97	4			
A458	EK3X9LI01D2PV3	AGGTGAAGTATTTGCTGGGTG	GACACACTCACGCTCTCCAA	148	107	0.98	14			

Target and primer information for experimental round B: read ID corresponds to the read in the short read archive SRA010805, beside primer sequences PCR product length with and without primers is given, as well as the percent identity of the respective mastodon-loxAfr1 alignment, "# of hits isPCR" gives a number how often the respective primer pair anneals to loxAfr1, and a "yes" in the row "consensus for all taxa" means, that sequence information is available for all taxa. The last two rows show scaffold ID and position of the targets used in the MCMCcoal analysis aligned to loxAfr3.

target ID	read ID GenBank SRA010805	F-Primer 5'-3'	R-Primer 5'-3'	product length in bp amplicon	product length in bp w/out primer	Mastodon-loxAfr1 alignment % identity	# of hits isPCR loxAfr1	consen-sus for all taxa	aligned loxAfr3 scaffold ID	loxAfr3 scaffold position
B001	FBUJGPD11GZ51K	CGGAGGGGTCATATAGAGGG	CTGCTCCCAAGCCATACCTA	279	239	0.97	0			
B002	FBHG2QE12HD6LJ	CACGCCTTAGTCTTCAAGGTG	GCCAAAAGTGAGTGAGGAGG	299	258	0.95	2			
B003	FBHG2QE07EDHIV	TGAGTGAGACAACAGCATCCA	GATCTGATCCACAACCAGTCA	253	211	0.89	2	yes	98	2,965,472
B004	FBHG2QE16JWWC2	GCCTTTGCATAGTCAATAAAACA	TATTTGTGCCCGTTCCAAAG	258	215	0.97	0			
B005	FBUJGPD11GXMVE	ATCCTGGCTGAAAGCAGAGA	GGATTATTTGCTCAGCATCG	285	245	0.92	0			
B006	FBHG2QE09FLWZM	CCTTGTCGTGCAGAAACAAA	AGCAAAAAGGTACACCAGGG	269	229	0.97	1			
B007	FBUJGPD16J0WE2	TGATGAACAAGCCACAGACA	CAAATCTGGATGGTAATCAGTGTT	247	203	0.90	1			
B008	FBHG2QE09FMB5Z	CCCAAAGGGGTATCGTTACTT	CCTGGGCCTTTTTCCTAGTC	257	216	0.99	1	yes	58	11,372,720
B009	FBHG2QE16JSW1W	GAAGCTGTCAGGACGTGTGT	GACTGTCACTTGCTGGAGCC	267	227	0.91	0			
B010	FBUJGPD12HGXOD	TTACTGGTTCATGGAGCCCT	TTCCTAGTCAATGTAACCGAATGA	221	177	0.90	1	yes		
B011	FBUJGPD14II254	CTGAATGTCTATCCCAGGGC	CCCTGCTGATTTTCCTCTTG	255	215	0.97	1	yes	58	11,826,278
B012	FBHG2QE11GSC9C	GGAGTGAGGAAGCTGGTTTG	ACGCTGAGTTTGTTGGGAAA	249	209	0.92	1			
B013	FAGAMMR16JW3QK	CTGGTGCAAGCCAATAGTGA	CCTGCGAGACGTCAACAGTA	229	189	0.91	2			
B014	FBHG2QE07D419I	TCCTGGAGGGAACTTCAGTG	GTCACTTCCTGTGCGTTTGG	255	215	0.97	1	yes	1	12,744,769
B015	FBHG2QE16JWYU8	GGAGCATGCTGGTTGTACTTC	GCATTGCGTTAGGCAAATCT	263	222	0.91	1			
B016	FBUJGPD12HN83D	GGCAATAGCAAGGGAAGCTC	GCGATCCAGAGTCAAAGGAG	224	184	0.96	1			
B017	FAGAMMR16JSX5T	TGGCTTCGAGAATGGTTTTC	AAAGAGAAGGAGGGCCACAT	252	212	0.97	2	yes	36	23,445,001
B018	FBHG2QE12HLRU	ACACAGGCAGCCCTGAAC	ATATGGGGATGGGGATGAG	241	204	0.96	2			
B019	FBUJGPD10F6XEK	AAGGCACCATGAATTTGGTC	GTGAATTCTCCTGCCTGCTC	252	212	0.95	2			
B020	FBHG2QE08EPYQS	TCACAACTGGACCAATAAGCA	TTGTTTTGCTTTCACATGCC	261	220	0.93	0			
B021	FBHG2QE12HB8LV	TCCCATTGTCCTCAGTTTCC	TTCCAACTCATAGCGACCCT	242	202	0.95	0			
B022	FAGAMMR16JU5ST	ACTAGTCTGGCAGAGTTGGTTT	TCAGGGTCCAGAATGAAAGG	244	202	0.99	1	yes	7	83,494,142
B023	FBHG2QE12HM1Z6	CAGTGAACACACCCATATATCCA	GAGAGGCTCACTGATCCAGG	244	201	0.92	1	yes		
B024	FBUJGPD12HFLXV	AGAGTCCCTCTCTGTTTCTGACA	TACCGCCTGAGTCAACCTCT	242	199	0.95	2			
B025	FBUJGPD11GYZSZ	TTTATTTCATCTTATTGAGGAGCA	CCAGCAGGAATGTGTCGTT	243	200	0.97	2	yes	19	35,638,888
B026	FBHG2QE07EC90R	CTTGATGTGCTCCCAGGAAT	GCACTGTCTATTTGGCCCTC	245	205	0.99	0			
B027	FAGAMMR16JY40W	CATAGGGACTGTGTGGGACA	TGTCATAGGCCTTTGGATGT	232	192	0.99	1			
B028	FBHG2QE07D4IFW	TTGTGGCCACTGTCAATGAG	GGTTCAGTGTGGCTGTCACT	257	217	0.96	2			
B029	FBUJGPD11GPK4Y	ATGAGCTGCTGGAAACATCC	GCAGTGTGGAACCAGACAAA	241	201	0.96	2	yes	10	20,603,229
B030	FBHG2QE09FJ6P0	TTGATACTGACTCCTGGCGA	GCAACCGTATAGGGCAGAGA	233	193	0.92	2	yes		
B031	FBUJGPD10GCK4V	GCAAAACAACCTTCAAATCCA	GTGGTTTGACACAGTGGCTG	238	197	0.96	2			
B032	FBHG2QE11G06BN	AAAACAATCAGCACCCGAAG	TGGATGTCCAGGTCCTTCTT	240	200	0.98	2			
B033	FBHG2QE11GXV6K	AGGTTGGTGTTCACCCAGG	CCAGCCATCTGGTAAGAGCA	231	192	0.97	1	yes	28	34,196,601
B034	FBUJGPD14IQ7WJ	GATGATCCCAGTAGCCTGGA	GGAAGCACTCAAGGAAGGAA	242	202	0.95	0			
B035	FBHG2QE16JZZ3C	TGGCTTGGACTCAAAGGAAA	AGCAGCTCACCCCCAGGT	242	204	0.94	1	yes	9	77,702,870
B036	FBHG2QE09FOE7E	CACAACTCTGAAAATGGGCA	GCGTATTCCGAAGACCTGTT	261	221	0.98	2	yes	84	991,369
B037	FBUJGPD16JWUO0	GGGGATATCCACGCTGACT	GATGATTGAGGTGGGGAATG	245	206	0.99	1	yes	31	18,100,580
B038	FBUJGPD11GZYE3	CCACCCACTCCTCTAGGGTC	TGATGAGCCCATCTGCTGT	223	184	0.96	1	yes		
B039	FBHG2QE11GW4A7	GCCAAAGCTAGGCCAAATAA	TCCTGGGCAAAACCTTTTTA	241	201	0.88	1			
B040	FBUJGPD14IOUNS	CTGTCACTGTTTGTGATTCTGC	GAGCTGAAGTGAAGGGATCG	218	176	0.98	1	yes	29	28,748,390
B041	FBHG2QE07D8ZHF	GTACTGCCTGTGGTGTGTGG	ACAGCTGCCTGCACAATCT	213	174	0.98	2	yes	32	27,727,719
B042	FBHG2QE14IVSKF	CCCATTGTTGCAGCCACT	CCATGGACTACCAAAAGAACG	221	182	0.91	0			
B043	FBHG2QE06DG3PO	GGCAAGCTAGTCGCAATGTT	GCTCAGGTCACCTCCACTTT	233	193	0.97	2			
B044	FBUJGPD13H5NWL	CCAGGGACCTCTGTCTTAGC	CTGATCAGCTCTGATTGTCCC	241	200	0.99	2	yes		
B045	FAGAMMR16JSCSH	CCCAGACTGGCTTACCTCTG	AGTGGACTGCCTGTTTCCTG	232	192	0.98	0	yes		
B046	FBUJGPD12HL41T	CCCACCCACCCACTCTTTCC	GGGTCCAGCCAGCGCTCC	224	186	0.98	0			
B047	FBHG2QE09FTUFS	CTACCTTGTAGCCCTGGTGG	TGGCAATGATTTAGAAAGCTTAGA	239	195	0.98	1			
B048	FBHG2QE12HNQFY	TGCTACCTTTTGAAGCTGATTG	CGCTCTTGTTAATGCTCACA	227	185	0.99	1	yes	34	14,004,436
B049	FBHG2QE12HNFYE	GCTTTGCAACTCCAGGTAGG	ACCAATCTCCAACAACCCAA	220	180	0.95	2			
B050	FBUJGPD14IO8KG	CTTCTGGGCTACCAGCACTC	CCATGTGTCATCACATCCCT	244	204	0.92	0	yes	72	8,627,248
B051	FBHG2QE14IOW8D	GCTTCAAGTCCTCTTCACTTTCA	CAAGAGGCAGTTGTTCAGACA	238	194	0.94	0			
B052	FBHG2QE06DPH8U	GGAGTTGTCAAGGATTTCATTTT	TTCCTTATTCACTGCCCAGC	226	183	0.89	1			
B053	FBHG2QE10F9B25	AGGAGTCCAGTTTCCGGTCT	CCAGGTCGACAAAGGTGTTA	233	193	0.87	1			
B054	FBHG2QE15I5HEY	AGCACCAGACATCACCCTTC	CACTCACAACACAGGATGCC	230	190	0.94	1	yes		
B055	FBHG2QE15JFTE6	GGATGCTGTTCCCCGTATTA	AGCCCTGTTTGTTGTAACGG	232	192	0.99	2	yes		
B056	FBHG2QE15I9ECF	AAACGTTACCATGTGCCAGG	AGTCAGGGTAAGGCACTGGA	217	177	0.96	2	yes		
B057	FBHG2QE07EA7D4	ATGGTTAAGCGCTTGGCTG	CGTTTAGCATGGATTGCGTA	221	182	0.91	0	yes	0	94,946,559
B058	FAGAMMR16JY0BL	TCTTTCACCTAAAGCACAGAAAAA	TGATGGCACCTAACAACAACA	230	185	0.98	2			
B059	FAGAMMR16J28ZZ	GAGGAGACTCTTGCTGCCAT	GGCTGCTGTGGCTTACTTTT	255	215	0.97	0			
B060	FBHG2QE09FK8YZ	AACATCGCCCAGGAACAG	GACTTTCATGCCTTGACAGATG	237	197	0.92	0	yes	0	95,646,240
B061	FBHG2QE14IQCGK	TACTGGTCAGTTTGTGGCCC	GGGCTCCTTCTGGTTCTCTT	227	187	0.98	0			
B062	FBHG2QE12HEX4Q	ATTCCCTCTGAGGTCCCCTA	AAAGGCACACATAAGCAGGG	242	202	0.96	1			
B063	FBUJGPD16J0V4R	TTCAAGCTGGTTTCACAAGAGA	TATTTGAAGCCCGGCCATA	237	196	0.98	1	yes		
B064	FBHG2QE07D6YR8	CCCTGAGCACTCTATCCCAC	CAAGGCCTCTTGAACTAAGGG	225	184	0.99	1			
B065	FBUJGPD13H8C94	CCTTTGCAGCAGATTTACCC	TTAACGGATCGCAACCTTAAT	229	188	0.94	1			
B066	FBHG2QE12HEX4Q	TGCAGGTTGTTAATGGGTCA	TGTGCTCAGGAGGACCTGTA	215	175	0.96	1			
B067	FBHG2QE16JT29W	TGGACCAATAAGCCACATCA	AAAATACCACGGATGGGTCA	220	180	0.90	2			
B068	FBUJGPD16J21D6	ACTCAGCTGGTGGCTCTCAC	CCGAGAGGATGGACTAGAGG	203	163	0.97	2			
B069	FBHG2QE07EBJ5T	GGACCCCGTTTGACTGTG	GGGAAAATATCCCACTCACTT	218	179	0.96	0			
B070	FBUJGPD10F58F6	GTTGTCGTGGATTTCGCTTT	GGCCTTCCTATTCATTGTCC	213	173	0.94	1			
B071	FBUJGPD11GZQ8X	ATAAATACCCCAGACCCGAG	AAGTCAGTCAGTGCAGGGCT	209	169	0.94	1			
B072	FBHG2QE06DI9V4	TAGTGGCCACAGGATTTGAG	AAGCTGTGAGATGACTGGGG	241	201	0.93	2			
B073	FBUJGPD11GRPKL	CCACTGTTACCTAACGGATGAA	TGTCTTCCCACTAATTTGGCT	264	221	0.97	1			
B074	FBHG2QE06DM9RM	GGGCAATAGCAGAGGAAAAA	GGAAGGCAATTTCTGTGGAA	262	222	0.96	2			
B075	FBHG2QE15JAS8I	CCCTTCCTTGGTAGGTGGAG	ACTGGAGGCACAGGCAGTAT	264	224	0.90	1			
B076	FBHG2QE12HEW2T	TCTCTCCTTAGGTTTTTGCACTG	CAAAGCACCATACAATGTTCAA	234	189	0.91	2			
B077	FBHG2QE16JVLHW	GGCACACACGTTTCTCTTGA	CGCACTTAAATACCAGGGACA	237	196	0.97	0			
B078	FBHG2QE10F6FS2	CCCAAACTTGGTGTGGTAGC	CCCATAAAGGTCACCTCCAA	267	227	0.97	1			
B079	FBHG2QE12HGBVQ	GAGGAGTGCCGGTTGGTG	AACCTCCCTCTTACAGAGACCC	249	209	0.98	2			
B080	FBHG2QE07D6807	CTCGTTTCCCCACAGATCAC	TCTTTGGATTCACTCACAGTGC	222	180	0.99	0			
B081	FBHG2QE12HGIHL	AAATTCCTGAGGGCACTGAA	CTCTGAGGCCACAATTAGCC	237	197	0.98	2	yes		
B082	FBHG2QE11GSXFB	TTTCTATATTGATTTCCCAGAAAAA	GGGTCTGATGTTCCCACAAG	261	216	0.98	1	yes	57	12,897,274
B083	FBUJGPD11GQSME	TGAGGGGAGAAAAGCAATCT	GTTGGAAGGTTATGGGCAAA	258	218	0.99	2	yes	19	34,250,944
B084	FBHG2QE15JCO8Y	CCTGGCTTGGCTGACCTG	ACACCAGCAACCTAAACATCG	232	193	0.98	0	yes	47	1,420,135
B085	FBUJGPD16J2J79	CTGCACCCCTTCTTCTGTGT	CTCATGTGTGTGCCTCATCC	247	207	0.94	0			
B086	FBUJGPD16JVC7N	GATGTGTCAGGTCCCCACTT	AAGAGGTGAAGATTGAGCAGG	224	183	0.96	1	yes	60	8,341,199
B087	FAGAMMR16J25MU	AGGACAACCTGAACACTGGC	GGTTGCATTTCTGCTCACTTT	242	201	0.94	2			
B088	FBUJGPD11GUBBF	TGCATATCAGGAAACCCTCA	TGTGGTACACTGCCTGAAAA	226	186	0.97	2	yes	45	15,439,266
B089	FBUJGPD13HYV38	GAAGGCACGCGAGTATATGG	CCCTCAAGGAGATGGATTGA	234	194	0.90	1			
B090	FBHG2QE09FIKG6	TCGAACACACTTGGGGAAAC	GACAAGTTGGTCCTCAGGGA	236	196	0.94	1	yes		

target ID	read ID GenBank SRA010805	F-Primer 5'-3'	R-Primer 5'-3'	product length in bp amplicon	product length in bp w/out primer	Mastodon-loxAfr1 alignment % identity	# of hits isPCR loxAfr1	consen-sus for all taxa	aligned loxAfr3 scaffold ID	loxAfr3 scaffold position
B091	FBUJGPD16JTCBG	ACCCCAAGGCCTCAGAAC	CTCCCACTTCCGGAGCAG	249	213	0.95	0	yes		
B092	FBUJGPD11GXNO9	GTTTCGTAGGGTGGCTTGAG	TTCCATCTTCTTTCGATGCC	209	169	0.89	0	yes		
B093	FBUJGPD10GB1KI	GCAATGCTCACAAGGTTTGAC	AATAACAAGTAGCAAACAATGCGT	236	191	0.95	1			
B094	FBUJGPD10F96MU	GAAACCCCTTGGTTTAAGGC	GGAAGGGATTCAAGACACAA	236	196	0.88	1	yes	750	2,039
B095	FBUJGPD14IM969	CCTGCATTGTGTAAGGCTGA	TGTTATTGTCTTGCTGTCAGGG	236	194	0.99	0			
B096	FBUJGPD16JV99F	TTCACCTAAATCACAATTGAAAAA	TATCAGCCCAATTGCCTTTC	257	213	0.88	1	yes		
B097	FBUJGPD15JD9FZ	AGCACTGCACGTGGACCT	AAGTGTGTAAGGTCAGCCAGG	228	189	0.97	0			
B098	FBHG2QE12HGRZ5	TCAAATCTGCTGCAAAAGACC	TTCTTTGGGTAGTCCATGGTATG	216	172	0.96	0			
B099	FBHG2QE08EZLLY	ACAAAGCTATCCGCCAGAGA	CCATCTTTGACCGTCACGTA	244	204	0.93	1	yes		
B100	FBUJGPD10GEEQ4	GCATAGAGGGCTCTGAGGTG	TAAGGTCTGTCAGGGAGGCT	223	183	0.98	1	yes		
B101	FBHG2QE13H59FF	TGATGACACCATTTTGAGGC	CTACCACCAGGTCATCCAGC	216	176	0.97	0	yes	1	75,524,245
B102	FBHG2QE13H9Q2Q	AATGGCCAGGCTATGAGAGA	GCATAATCCTCCCCCATTCT	243	203	0.97	1	yes	10	34,024,629
B103	FBHG2QE16J11CA	CCGGCACATTATGAACTTCC	TGAACAGGGTGAGGAGGTGT	230	190	0.99	1			
B104	FBHG2QE06DQBLA	CACGAGCTTTAGACAGGGGA	CTGTGCAGGGTGATGCTG	223	185	0.95	2			
B105	FBHG2QE09FSDM3	ACCTAAAGCTCGTTGGTTCG	TGAATTCTGCTCAAAAGACTACG	221	178	0.97	1	yes	9	74,552,276
B106	FBHG2QE16JU6ES	GCTCCAGGTAGGCCTTCAGT	TCCTGAGGCCCAGAGTAAAA	233	193	0.94	1	yes	64	2,463,063
B107	FBHG2QE09FVL3O	TCAACATTGTTCAACATCCCA	CCTCACTCAGCCCCTGAA	208	169	0.91	2	yes	17	27,878,148
B108	FBHG2QE06DGS5W	CCTGATTTCTCTGCTTTGGAA	GGCCATCAAATCAGCTTCAG	235	194	0.97	2			
B109	FBHG2QE06DO7NO	CATCAGGAACAAGCCAAAGG	CCCCAGGCCATATGTGATAC	234	194	0.88	1	yes	10	49,857,693
B110	FBHG2QE07ECR9G	GGATGAAAAATTCAGCCACC	GTGTAGGTCCCTGCATCCC	227	188	1.00	1	yes	5	95,301,740
B111	FAGAMMR16JZ0NT	CTCCTTCTCTGCACATTCCA	AGGGACAGGAATTAAGGGGA	231	191	0.99	1	yes	27	12,707,099
B112	FBHG2QE16J1QTV	CCCAGACCTGTGATGAAAGAA	TTATTCCCTGAAATGGGGCT	231	190	0.91	2	yes	1	17,440,473
B113	FBUJGPD12HMUFW	GGAAGGGAGATTCCAAATCA	CAAGGCCTTTTATATGAGTCCAA	249	206	0.98	1			
B114	FBHG2QE14IKKAG	CTTTACCTGGGCTGATCCAA	TTCAAGTCACCTATCAACCCA	207	166	0.91	1	yes	1	50,767,865
B115	FBUJGPD13H3H2E	TTTGTGTGTCAATCTGCTATTTCA	TACTGACCGCTCTGAACAGG	201	157	0.93	1			
B116	FBHG2QE06DNXY7	AGTCACACTACCCCCTGCTG	CCTCTCAACCCATGCTGTTT	220	180	0.96	1	yes	19	7,098,600
B117	FBHG2QE09FRUFI	TTCTAAGGAGCATTCTGGCTG	TTTAAAGTGTTAAAAAGCAAAGACG	237	191	0.93	0			
B118	FBHG2QE10GE4QZ	TGTTCCTCTTGGCTTTGAGA	TTCACAACAAATCCCTTCCC	226	186	0.96	1	yes		
B119	FBHG2QE11GRZ0R	CCAAGTGCACATGAGCAAAC	CAGCCTAGGGTTGTTTCCAA	230	190	0.96	1			
B120	FBUJGPD10F7MV4	GGGTCAGGCTCCTCACTCTT	TGAAGAAGGAACCACAGCAA	232	192	0.96	1	yes	4	47,945,658
B121	FBHG2QE06DTLO1	CAGAACAGTCCAAAGGGCAT	CCGGGCTGAACCTTTGTT	211	173	0.96	0	yes	12	32,920,038
B122	FBHG2QE13H8PO8	ATAACCTTTGCCCAGACTCG	GTGGTCAAGTGGAAAGGGAA	221	181	0.96	0	yes	9	74,542,366
B123	FBUJGPD12HB5AH	CAGGTGTCTGGAAGGAGGG	GGCAGAGATGGTCAAGTTCC	216	177	0.91	0	yes		
B124	FBHG2QE13HXT7R	CACCTTTGTCGCGAGGAG	CCGTGATGTTCCGCTCTTTA	225	187	0.98	0			
B125	FBHG2QE08ERFJ9	GGAGGAAAACCCTGTAGTTCTG	GACTCCTTCCTAAGCCTCCA	248	206	0.95	1			
B126	FBHG2QE08EY9HT	GTGGAGCTTGGCTCACTAGC	TTTGCAAGTGGCCAGAAAAT	203	163	0.95	1	yes	13	36,001,336
B127	FBHG2QE07EA6ID	AAATTTTGCTCTGTCCGACG	TAACGGCCGACTTCAGAAAC	242	202	0.92	2			
B128	FBUJGPD15I63MK	TTCCAGGACTCCAGGCCC	GTCTTTGCCATGATAAGCATCTTT	209	167	0.92	2			
B129	FBHG2QE13H3JXD	CTACAGGCCCCGGCAAAC	ATGTGGCCTGCATCCAATAG	218	180	0.87	1			
B130	FBHG2QE07D8BBZ	CGGTACTTAATGGGTTTACAATACA	CCGTGGATTACCCATTGTTC	213	168	0.96	1	yes	117	1,875,586
B131	FBHG2QE16JVU2K	CTCAAGTCTAGTGGAGGCCA	GCTCAGGTAGCATAACATGGCT	212	170	0.93	1			
B132	FBUJGPD10GBST7	TAGCGCTAGAAGAAGACCCG	TGTTACATGAGTGATCGAGTTAAAA	213	168	0.99	1			
B133	FAGAMMR16JU5Z4	GGCTTAGAAAAGGCCCTCAG	TGCATAAATTTTACTGGCGAA	220	179	0.98	2			
B134	FBHG2QE06DPANW	TTGGACAGTATCCTGCTGTGA	GGGAAGTCCACAACCTATCTG	226	184	0.98	0			
B135	FBUJGPD14IT092	TAATTGGCCTGGCCTAAAAG	TCCTGTTGATGTGCCCTACA	219	179	0.95	0	yes	22	30,306,397
B136	FBHG2QE06DPQ7U	TGCAGACAGACTATGGGCAC	TTGGCAGATTGCAACTGTTT	208	168	0.98	1			
B137	FBUJGPD16JQ2KC	AGTTCTCTAACACACACGGGG	TGGCAATGCTTTACTGAGGA	215	174	0.98	2	yes	121	1,727,613
B138	FBHG2QE07D5K9N	GAGGAAACTGGACAAGTCAGC	AGCAATCACAGAGCCAAACC	230	189	0.89	2			
B139	FBUJGPD16JSZOZ	TCGGAGATCACAGCAAACAC	CTGCTAGGCTGAGCCAAAAT	216	176	1.00	2	yes	5	32,465,128
B140	FBHG2QE12HJC1A	TCTTTTAAAGGCCACGCAGT	GCTTTAGCCTGATCCCTTCA	213	173	0.99	0	yes	13	51,371,453
B141	FBUJGPD16JZZUT	TCTAATCAGGAAGAGGGAGGG	TGTCACAGAATGAGGACCCA	190	149	0.95	1	yes		
B142	FBHG2QE13H9BA4	CTGCATAATTGTGGACAGCC	AACGCAATTAACCAGCTTGC	240	200	0.96	1			
B143	FBHG2QE15I6EUU	GGCGAGCCACCAATTATG	GAAATTGATCGAATGTGCAA	207	169	0.95	1	yes	5	16,516,502
B144	FBHG2QE16JT4ZQ	GAAGCTGTTCAGAACCCAGG	GGGCACTAGCACCTTTGAGT	225	185	0.94	2			
B145	FBHG2QE08EYAEJ	TGCTGTGACTTCCCAAAGTG	CATGAACATCAGGCAGGCTA	217	177	0.97	2			
B146	FBHG2QE08EQ74W	TCGCTTGCTGAAAGTGAAGA	TGCAACGCATTGTTTGATTT	234	194	0.93	0			
B147	FBHG2QE14IPW59	CCTTTGAATTGTGGTGTTGG	TTCCTCTTTTCTGCTGACCC	216	176	0.96	0			
B148	FBUJGPD14IPHVA	CACTGGAATCTTGTGGCTTG	TCAAACAACTTCCCCCACTT	212	172	0.88	2			
B149	FBUJGPD14IO6H1	AGTAGGCTGCAGGGCAGAT	AACTGGTTCACTGTCCAGAGG	227	187	0.91	2			
B150	FAGAMMR16JVO3I	CCCTCAGCTTGGGTAACTTC	ATTGTCAAGCTGTGGTGCAG	216	176	0.96	2			
B151	FBHG2QE12HME5J	CATACAGGCACCAAATCCCT	GTCCTGTTGAACTGGCTGCT	231	191	0.90	0			
B152	FBHG2QE09FND0V	TGATGCTCTACCATAGCACTTCAT	CCAATGGCTGTAACTTTGTGG	217	172	0.95	1	yes	6	53,782,150
B153	FBHG2QE10F52CK	CACCTCCCTCCTTACTTCCC	GGCACTGCAGGCTCTATTCT	213	173	0.96	2			
B154	FBUJGPD13H1JEF	GGCTTCACATTTTCCTCCAG	TTTCTGAGTGCTGGGATATTGA	224	182	0.97	2	yes	64	5,960,942
B155	FBHG2QE15I9JM5	CCCCTGCTCCCCCTATAAT	CCAGGATCAGAATGCAATGA	213	174	0.99	0			
B156	FAGAMMR16JV2WU	TCAACTTAGCTTCAGGGCGT	AGGGGTGTCCAAGGGAGAT	206	167	0.97	1	yes	47	16,627,053
B157	FBHG2QE12HIO88	GTTCATGGGGAGGAAAATTG	GGGCATCAGAGCTGAAACAT	226	186	0.98	2	yes	63	9,693,741
B158	FBHG2QE08EZHEP	CCATGTTAGGCGGTGGTAG	CTTGTTTTTGAGTAAGAAGTTGACA	226	182	0.96	0	yes	18	21,157,709
B159	FBHG2QE12HF0FW	CCTTGCTTTCAAGAAGATGAA	CTCTCTCCTCGACCCAGATG	234	193	0.97	0	yes	45	18,085,112
B160	FBHG2QE11GZUFX	TAACCAAAAGGTTGGTGGCT	TAACTAAGCCTGAATGCCGC	214	174	0.96	0			
B161	FBHG2QE16J13WZ	CTCAAGTGGTTGATATGGCG	CTGGACGGTCTGTCAGGAAT	237	197	1.00	1			
B162	FBHG2QE13HZKRS	TCAAGACGTGGGCTTAGTCA	AAGGGGAACTGAGCAACAGA	248	208	0.98	2			
B163	FBHG2QE08EQTPH	AAGACTGTCAACTGCAAAGGG	GACACCATTTTGAGGTTCCC	226	185	0.99	0	yes	33	21,481,153
B164	FBHG2QE16JUNV8	AAAATTCTCCAACAGCCCAG	CGATACAAGGTTAGCGCCTC	214	174	0.97	0			
B165	FBHG2QE09FLS0H	AGGCTGTGTGCAATCCTTTC	TCGGAAATCCAGGAACTACC	212	172	0.96	1			
B166	FBHG2QE09FRKHR	TGCCTGTAAGGGACTTCTGG	CCACACAAAGGAGACAGAGC	195	155	0.98	1	yes	29	31,534,560
B167	FBHG2QE14ISS79	TGAACAAGGTTAAGGCTCACC	GCTGTACAACTTCTTGGGGG	227	186	0.96	1	yes	65	11,353,589
B168	FBHG2QE13HXK49	CACTCCAGTCGAAGCTGTCA	TTGCTCCTTCTTTTTCTTTAGC	211	169	0.99	2			
B169	FBHG2QE10F65PN	TGTTTTGGGATTTGCCTTTT	TGGGAAACCTTTATGGAGCA	240	200	0.97	1	yes	6	54,694,957
B170	FBHG2QE16JZVQN	AAGTCACAGGGGACACCATC	AGAAGATTTGCCCAAAACCA	187	147	0.96	1	yes	67	5,778,372
B171	FBHG2QE11GRHMU	CATCTGCTACCCTAAGCCCA	GCAAGGTGCATAGGATGTGA	210	170	0.98	1	yes	1	101,862,519
B172	FBUJGPD14IUNDT	AAACCAGGTTCTCAGAAGGGA	GAGAATTCACGATTAGCGACG	219	177	0.97	1	yes	18	34,656
B173	FBHG2QE10F6O1J	GCATTGGGCTTTTGGTTTTA	TATCAGGAATCCACCAGCCT	242	202	0.98	0			
B174	FBUJGPD10F6USF	TGGTGGCACAGTGGTTAAGA	TCTGTTTGTTTGGCTTATGTGAA	224	181	1.00	0	yes	50	9,445,613
B175	FBHG2QE15JGYUA	ATGAACTGATGGAGGGATGG	CACTGACGCTCTTGTGGCTA	216	176	0.99	0	yes	3	78,448,534
B176	FBUJGPD10GGSAK	TTGTGAATTGCAGAGGCAAC	TTCCAAGTCAAGAGTTAGGAAGTG	235	191	0.96	0	yes	19	35,050,101
B177	FBHG2QE14IUJWE	TCTGCTGCTTGTCTTGCATT	TAACAATTCTCCCCTCTGCC	223	183	0.96	1			
B178	FBHG2QE12HNAEL	CAGGAAGGCCATTGTCTGTG	TCGAAATAGTCCAGCCCTGT	194	154	0.98	2	yes	99	715,849
B179	FBUJGPD10F5PKB	CCAGCTCTGGAACCATCTGT	CCCACAGGTGGCAAATATAGA	219	178	0.98	2	yes	21	15,471,258
B180	FBUJGPD10GB711	TTATCGTGGGAGAGTTTGGC	AACCTTTGTCACACGGCTTC	218	178	0.97	2	yes	58	10,358,110
B181	FBHG2QE08ESODZ	AGAGGCTTGTGCGTTGCTAT	GGTGGCGTGATAGCTAGGAG	213	173	0.93	1	yes	18	11,408,461
B182	FBUJGPD15I845X	GCAAGATAGGTTAGGGGCTTG	CTGAGAGGCAGAGGGAGAAA	209	168	0.93	1			
B183	FBUJGPD15JENB8	ACAATGCCCGTACCTGAAAG	GCCAAGCTTCTTGCTTCCTA	204	164	0.99	1			
B184	FBHG2QE15I6HZ8	TTTTCCCAAGTTGATGGGAG	CCTTACCAGCCTTGTCTCCA	232	192	0.95	2	yes	37	19,442,139
B185	FBUJGPD10F4FGQ	TGGACAAAGAATAAGGAAGACCA	ATGTCCTTCTCCAGGGACTG	212	169	0.93	0			
B186	FBHG2QE13H08E0	TCCAGCTCACTTATGAGGCA	TGGAATTGAATATTCCCCCA	225	185	0.94	1			

target ID	read ID GenBank SRA010805	F-Primer 5'-3'	R-Primer 5'-3'	product length in bp amplicon	product length in bp w/out primer	Mastodon-loxAfr1 alignment % identity	# of hits isPCR loxAfr1	consen-sus for all taxa	aligned loxAfr3 scaffold ID	loxAfr3 scaffold position
B187	FBHG2QE09FLVCG	AGCTCCCAGATCAAACGAGA	GGGGTTCATCCAGCCTCTAT	233	193	0.96	1	yes		
B188	FBHG2QE14IS2J2	TCCACTTGAGGCATTGAAGA	TGAAGTTCCACAGCCATCAG	232	192	0.99	1	yes	8	28,743,344
B189	FBHG2QE09FKAVU	CCTGGACTGGCTACAGGCTA	AACACAGGGCACCCAAGA	199	161	0.99	2	yes	64	10,988,385
B190	FBUJGPD12HEBCW	AGCCACTGAAGTGTGTCCG	GTCTGTCACTGGGAGGGTGT	208	169	0.96	2			
B191	FBHG2QE07EDNG2	AATTTCAGGAGGTTAATGGCTT	TCTGTCCTGTTTAGGTTTTCAGC	218	173	0.96	2	yes	30	7,557,074
B192	FBHG2QE14IK2L7	ATTGATCCTCCATGACCACC	GTCTCTGGGGAAAGATTGGG	221	181	1.00	0	yes	18	30,278,164
B193	FBHG2QE10F6LXI	AAGCGTGCGTATGTGTGTGT	ACCTAGATTTCTGCAGGGGA	215	175	0.94	0	yes	27	13,377,102
B194	FBUJGPD11G0IU9	GCAGGTAAGAGAGCAGGCAG	CCTCCATGATGCAATCTGATA	229	188	0.91	0	yes	18	40,691,157
B195	FBHG2QE14II3IP	TCCTATTGTGAAACATCTTCCTCT	TGAACGTGAGGCTAGTTTGG	212	168	1.00	1	yes	12	34,660,356
B196	FBUJGPD16JYHN2	TCAGTGGTCTTTGACTCTGTGG	CCTGGCTCCTTAACTGCCTA	214	172	0.97	2	yes	16	24,765,073
B197	FBUJGPD14IKZ7Q	AAACCACCCTGCAACTATGG	CTCATGCCTCCTGAACCCT	204	165	0.95	1	yes	3	9,545,026
B198	FBHG2QE07EE7F1	TGTGTGTCTGACCACCCATAG	ATTAAGGAGACAGGACGGCA	193	152	0.99	1	yes	28	12,702,582
B199	FBHG2QE06DMMPQ	TGGTACCCTTTGTACCTCGG	GGACAATGTGGATGAGGGAG	210	170	0.98	1			
B200	FBUJGPD14IKWYZ	CCAGGAGCCTGGTATCTGTC	GACACTCAAGGTGGACAGGC	218	178	0.97	1	yes	29	26,771,641
B201	FBHG2QE16JWNC8	CAATTGGTGGCACAGTTTGA	GGCCTAGGACACAACGAAAT	216	176	0.98	0			
B202	FBUJGPD13HW1Y2	GCAGGCAGTTAAGCAGCAGT	ACCAAAAACCAAACCCAGTG	212	172	0.95	0			
B203	FBHG2QE06DIG65	GGGGACTCCACAGAGAATGT	TGGCAACTGTCAGGAAGAGA	209	169	0.98	1	yes	122	2,400,993
B204	FBHG2QE11G0GDB	TTGGCAGAAAATTGAAGCCT	AGTGGCTACGTGGACTCAGG	213	173	0.99	1			
B205	FAGAMMR16JY3WD	AACCCGCAATTTGTGGTAAC	GAGTCATCCCCATGACTGGT	211	171	0.98	0	yes	3	30,984,529
B206	FBHG2QE15JB5B3	TTAATGTACCGTCAACCCCC	CTCAGCTGGAGGAAACAGGT	209	169	0.96	0			
B207	FBUJGPD13H9UOT	ACAAGTTCCCTGATCTCCCA	CTGGCCACTCATGCTCCT	226	188	0.92	1	yes	319	48,206
B208	FBHG2QE08EPZY4	TTCAAGGTCATGCACTATCTTTC	GCACTTAAACAGTCTTGGATGC	192	147	0.92	1			
B209	FBHG2QE12HGN7U	TCAGCAGATCTCCAGTGTCG	AGCCAACAACTCTGAGAGGG	205	165	0.95	2	yes	42	9,116,261
B210	FBHG2QE13H5MMY	GCTTCTTCTCCACTTCTGGG	TGTGGTCATTATGCCTTCACA	229	188	0.99	2	yes		
B211	FBUJGPD12HJ3JY	CCCAGCAAAGAAGGCTTTTA	GGGATTAATCTGGGCATCAA	241	201	0.99	1	yes		
B212	FBHG2QE10GCBE5	GTGGCAACTGTTCACGTCAC	CATCCTTCCCAACAGTCTGC	218	178	0.96	1	yes	14	47,737,118
B213	FBUJGPD13H01SL	TTCTTTCACCTGGATCTGTGG	TTACTTGGATGACAAGGGGC	221	180	1.00	2	yes		
B214	FBUJGPD13H2RBJ	ACTTCCCCTCATCCCTTCAC	TCACATGAGGTAAAAATTGGCA	237	195	0.96	2	yes	36	21,174,749
B215	FBHG2QE12HM20S	CCTGTACTGAGTCCCAAGGC	CTTCACATCCACATCTCAGCA	196	155	0.96	1	yes	127	820,250
B216	FBHG2QE09FMQNN	GTCCACAAAGTGCCAAGACC	GCATTGAGGCGAGGTCTAAA	191	151	0.96	1			
B217	FBHG2QE15JE7E6	TATTTTGAAGGGAGGAGGGG	TCAGCGCACATTAGCTGTTC	216	176	0.99	0	yes	65	11,061,277
B218	FBHG2QE08EV9NS	TTGTGTTCATGTGGAAGGTG	TCTTCCTCCAATCCTGATGC	199	159	0.90	0			
B219	FBHG2QE13H4M8U	CCTGGGGCAGACTCCAGCTT	GGGTGCGGGAAGCAGCCT	207	169	1.00	1	yes	6	93,344,124
B220	FBHG2QE10F8IHA	TGCATGTAAAGGATGAGTGAGC	TTTGCTGGTAACTGCACATAATCT	205	159	0.95	1	yes	22	10,273,074
B221	FBHG2QE11G1LGC	CGTGTCTATCCAAAGCTCCC	GTGGGACCATGAGAAGCAGT	225	185	0.94	1	yes	72	7,687,676
B222	FBHG2QE06DNX08	GACAAACTGCATTCTGACTTGC	GACCCATGTCTAGGGCTCTG	224	182	0.95	1	yes	28	3,604,923
B223	FBHG2QE11GTYCW	GATTAGCTGAGGGAGGCAGA	GGCACATTTGGAGGAGATGT	227	187	0.99	2	yes		
B224	FBUJGPD15I7STP	CCATCTTGGACTCACCGAAA	TGCAGCGTGTTCCCTTAAGTA	237	196	0.95	2			
B225	FBHG2QE10GAJZB	GAGTGATGTAAAGCCTGCCC	TGGTGCATGTTTCTGTGTTC	207	167	0.98	2			
B226	FBUJGPD14IMZ37	TTCAACAAATAGATGCAATGCTG	TCGATGTACCGCTGAAAGC	220	178	0.99	0	yes	102	4,393,297
B227	FBUJGPD11GXMS7	TCAGGCACACCTTAGTCCTT	AAAGAAAACAAAAATCCTCACAA	225	182	0.93	0			
B228	FAGAMMR16JU81N	TTGCTGTTGCACTTACCCAG	AAAAGGAATGGGTGGACTG	236	197	0.98	1			
B229	FBHG2QE10GEREW	TCTGGGCCAGCTCTGTATTT	AACGTTCCTTGGAATCTGACA	211	170	0.93	1	yes	507	66,245
B230	FAGAMMR16JZF2Q	GTGGTGGTCACAGCCTCG	GTCTGGGCAGTCTAGCTGGG	203	165	0.96	1	yes	71	941,543
B231	FBHG2QE14ISADM	GAAGGCTTCCTCTCCCTGTC	GGCCTGCATCACCTCTTATC	233	193	0.95	1			
B232	FBHG2QE06DH180	GCATGACGTGGGAGGTGTAT	CCAGCAGCACCTCACTCAG	226	187	0.95	1			
B233	FBHG2QE12HK8W8	CGCTGGTAACGTGAGAGTCA	CCTGAAGCGCCTTTCTCTG	242	203	0.99	1	yes		
B234	FBHG2QE06DHUJT	TCTAGCCCTGGACCTCACAC	GCACCAGGTAGACCCAGAGA	150	110	0.93	1	yes	9	83,036,813
B235	FBUJGPD15JG92V	GGCCCTGTCCCTAATCAAAG	AGTGACATCAGAGTTGGTCCTC	206	164	0.93	1			
B236	FBUJGPD12HDZ32	GGAATGTGGAAAAGCAGAGC	GGACATGGTATTTCCTGGCT	192	152	0.96	2	yes	9	40,645,422
B237	FBUJGPD14IMZAP	TTCATCCTTGTCAGAAGATCAGA	GCATCTGACATTCAGCTCCTT	224	180	0.96	2	yes	50	5,580,361
B238	FBHG2QE15JDH47	ATTTCCATTGGCATCGGTAG	GAAGTTCCTGCCTAGACCCC	204	164	0.99	2			
B239	FBUJGPD10F88N4	TGGATGAAAGCAAAGAAGAGA	GATTCTGACTCATGTGTATCGGAG	227	182	0.98	2			
B240	FBHG2QE07D7KLN	TTCCTGTACCACCTTGACACA	TGATAGCTTGTTCCTCACCCA	216	174	0.98	0	yes	151	80,293
B241	FBHG2QE10F3Y9Z	CTGGGACTCTTCCTCTGGG	GCAATTCAGGGAGAAGCCA	210	172	0.91	1	yes		
B242	FBHG2QE10GA3BL	CCGTGCCATTTTTACAAGGA	TCGAAAGCTAACGGGAAATG	244	204	0.93	1	yes	127	1,455,825
B243	FBHG2QE14IVSUR	GTGTGCCAGTCTCCATGCTA	TAGTAGCCTGGCTGCCTCTG	204	164	0.95	1	yes	5	94,097,177
B244	FBHG2QE13H1STR	CACCAAGAACCCTACTCATTCA	AACCCTTACTAGGCCGTGCT	212	170	0.96	1	yes	129	1,478,153
B245	FBUJGPD10F35ZZ	TGCTTCAGCACCTTCTTGTG	AAGCCATCCTGGTTCCTTTT	201	161	0.96	1	yes	49	296,180
B246	FBHG2QE08ET82O	ACACACCTGCTCAAGCTCCT	CGGGTCAGAGCTCACAGAG	200	161	0.99	1	yes	27	367,552
B247	FBUJGPD13HWCT3	CCCAGTCTGGCAAAAATACC	CTCCCCATAGGAACGTCTCA	197	157	0.99	2	yes	8	39,229,258
B248	FBUJGPD10F7U4S	TGCTCCCTGTATGCTGTGTC	AGCCTGGGTAAGAGGAGAGG	223	183	0.99	2	yes	11	49,000,000
B249	FBHG2QE16JUHCY	GGTAAACGACCTTTTCCTTCCT	GAAAAAGTGGGAGGAAGCTG	231	189	0.99	2			
B250	FBHG2QE08EWP1T	CAGGGGTACAGTTTCACTGATG	TGTGAGAAGACAATGTGTAGGCA	199	154	0.98	2	yes	11	27,980,216
B251	FBHG2QE09FKOSU	TGGGTGAACGAACAGTTTCA	CCATGATTTATGCCCTCTTCC	191	150	0.98	0	yes	47	12,040,065
B252	FBUJGPD12HMH24	AATACCATGGCTTGGGTCAG	CCGGACCAATAAGCAACATC	220	180	0.94	0			
B253	FBHG2QE09FI2XG	CCGTCTGCCTCCATCATTA	TGAGGGCCTCTCAACCTTTT	230	191	0.97	0	yes	3	10,675,793
B254	FBHG2QE11GTIJ6	GCATGACCTCACCTGTTTACC	AGCCATCGCTTGAGACATTT	206	165	0.94	0			
B255	FBUJGPD11GVQJB	TAGGGGACTGATTCCACAGC	GGTTTGGAAGTGGAAGGACA	189	149	0.89	1			
B256	FBHG2QE06DQMJ2	GGGACGGGTGATGAGTAATG	CAGGGCCAATTTAAAAAGCA	196	156	0.96	1	yes	86	3,693,404
B257	FBHG2QE10F6PFZ	TCAAGAGGTTTGTTACCAAGGG	ATAGGGCTTTTGCAGAGGGT	224	182	0.97	1			
B258	FBUJGPD11GUOQ6	AGACATCAGCCGTAACTCGG	TCAGACCTCACTGTGCAAGAA	233	192	0.98	1			
B259	FAGAMMR16J1N29	GCCCCACCCTACTGATGTTAT	TTGAGTTGATTCTGACTCGTAGTG	199	154	0.87	1	yes	99	1,861,855
B260	FBUJGPD12HD8EE	TCTGGCAGTCTCCTGAAAGC	CAGCAAGCAAGGTCTTGTGA	215	175	0.88	1	yes	20	10,655,556
B261	FBHG2QE07D6R67	TTCCTCTGCGTTCTTTTCTCA	TCCAGAGGTGGGCTTCATAG	237	196	0.98	1	yes	1	5,314,690
B262	FBUJGPD15JGOXJ	CTAGATGCAAATTCGGGGAG	CTCTCCGGGTGGCTAGAAC	238	199	0.93	1	yes		
B263	FBHG2QE13H4KT3	TTTCCGACTCATAGTGACCCT	AAGGATGGCGAGACTTCATC	238	197	0.91	1			
B264	FBHG2QE12HEUHS	CCTTTCTCCATCCAGGACAC	TGGAGGAACCCTTTGTCAAC	155	115	0.96	2	yes	21	4,340,823
B265	FAGAMMR16JS6GX	TTTTATAAAGGGGAGGTGGC	CACTCCTCCATCCCTGAAGA	197	157	0.98	2	yes	63	10,535,568
B266	FBUJGPD10GFGEX	CATATTTTGGGTTGCAAGGAA	GCACAGGGGGTAACTGTCAT	195	154	0.99	2			
B267	FAGAMMR16JTFND	CTCCAGCTCCATCACTCCAT	AGAGGGGCCCTATTGAGACT	215	175	0.95	0	yes		
B268	FBHG2QE09FMKEK	TGCCACCAAGTCAATTCTGA	CATAATGAATGGGAGCCCTG	201	161	0.99	0			
B269	FBHG2QE09FVFT2	CCTCACCACATCCTCCACTT	GCCAGATGCTTTTCGAGTTT	205	165	0.97	1	yes	9	45,296,098
B270	FBUJGPD15JEIXC	GCTCCTCACCAAGCACAGTT	CCTGTCTGAACGCTAAGGCT	226	186	1.00	1			
B271	FBHG2QE09FHSI6	TGAGACCATAGCACTGGACTTG	AAGTTCCCTATCCTGAACCCT	217	174	0.87	1	yes	9	31,042,083
B272	FBUJGPD11G1BQO	CTGCCAATTGATTGTGTAAAGA	GGTTGCCCAGTGGTTAATTG	231	189	0.97	1			
B273	FBHG2QE16JWZPH	CATGGGGTCAAAAAGGAAGA	GCAACCCCAAACCCTAAAGT	211	171	0.93	1	yes	113	2,459,782
B274	FBHG2QE06DMSFL	TTCATATGACCCTGAAGAACTTG	TCAGGGTAGTAACCATAGGCTCA	228	182	0.99	1			
B275	FBHG2QE10GCX19	GAGAAGGAAGACGCAGATGG	AGGTGTCAGCATCGGTAAGC	219	179	0.98	1			
B276	FBHG2QE13H66FP	GGACTGGCTCCTCCTGATAA	ACGCAGGCAGGATTTACAAC	229	189	1.00	2			
B277	FBUJGPD15JAI0D	TGCTGAAATAAGAACCCCTCA	CCCTCCAGAGGAAATCGTAG	206	165	0.91	2	yes	43	11,627,841
B278	FBHG2QE13H2TW1	GAAGTTGGCCTTATTTTTCTTTCA	GAGCAAGGAGACTCCACAGG	220	176	0.94	2	yes	16	43,716,062
B279	FBUJGPD16JR8RU	AGGAAGGTTCTTCCATGGGT	AACCAGTCATGGGAAGGGAT	186	146	0.98	2	yes		
B280	FBHG2QE07D5ZS6	TGCAATTGTTGCCATGAGA	GGTGGTGGATTTCTAGGACA	228	189	0.99	2			
B281	FBHG2QE09FIDOD	AGGTCTGCCAACCTAGGGAC	ACAGTTCAGCCTCAGGCATC	225	185	0.98	1	yes		
B282	FBHG2QE10F9I2L	CAAAGCATAGACGAGCCCAT	CAGAGATAGCTTCCCGTTGG	232	192	0.89	1			

target ID	read ID GenBank SRA010805	F-Primer 5'-3'	R-Primer 5'-3'	product length in bp amplicon	product length in bp w/out primer	Mastodon-loxAfr1 alignment % identity	# of hits isPCR loxAfr1	consen-sus for all taxa	aligned loxAfr3 scaffold ID	loxAfr3 scaffold position
B283	FAGAMMR16JZKFE	GTGGGGTTATTGGAAGGGTT	TGCCTGCTCAACAGAAGATG	214	174	0.95	1	yes	25	21,228,071
B284	FBHG2QE15I8O26	ACTGGGTGGGCTCTAGGTTT	TTTGATTTTCCAGCCAGGAG	219	179	0.95	1	yes	11	55,035,949
B285	FBUJGPD11G0VYR	CTGTTCCACAACCAAGCCTC	AATGACATTCGATGGAAAAGAA	194	152	0.95	1	yes	64	7,081,948
B286	FBHG2QE07D9OIX	TGGAACAGTTGACTGGGTTT	TGTTGGCAATTATGGCAATG	195	155	0.99	1	yes	9	52,027,714
B287	FBUJGPD13H6T03	ATGTTTTGAAGTGCCAGGCT	TTACGTCCTATGGTTCCCCC	217	177	0.93	1	yes		
B288	FBHG2QE15I88EI	CATGGAGATGGTTCATAACACAC	TGTGAATTCGTTCCAACAAGA	215	171	0.94	1	yes		

A.1 – long

Target and primer informtion used for sequencing error rate estimation using direct Sanger sequencing, read ID corresponds to the read in the short read archive SRA010805, PCR product length with and withoutprimers is given.

target ID	read ID GenBank SRA010805	F-Primer 5'-3'	R-Primer 5'-3'	product length in bp amplicon	product length in bp w/out primer
A002	EK3X9LI02F3RBB	GCCTTCTTCTGGCTGATGAT	AGCTTTCTGGGCCTTCTTTC	746	706
A004	EK3X9LI01DC8H2	GAAGTGCCGGTTATACACCAA	GCGAAAGCTGGACAATGAAT	749	708
A005	EK3X9LI01BHMHV	GCAGTGTTTCCTCGATTTCC	CTGAGCCAACAGGAGAGGTC	750	710
A008	EK3X9LI01CV00E	TTCCTCTTCCTGCCTTCTCA	GGACATCTACATCAGCTGGC	749	709
A010	EK3X9LI01DQL4E	ACGTGGACAGGTTCGACATA	GCAAACAGGTCATCCGAGTC	750	710
A012	EK3X9LI01BCGQI	CTGGTGCTAACAACCAGGATT	TGAGTTGGACTTGACAGCCC	746	705
A013	EK3X9LI02G5E9S	ACTCAGCCCAGGTATTGCAT	CCTTTATCCCAGATGCTGACA	746	705
A015	EK3X9LI02JWLAQ	TTTGTTTGATGTTTGCAGGTG	TTGGTATCACGTGGGTATAGCTT	743	699
A016	EK3X9LI01EN54P	TGACAACAGAGTTACCCACACC	TGCTGGGTAATACCAGTATGAGG	732	687
A019	EK3X9LI01C0K93	CATGCTCGTTGCTCTGTCTC	GCTAAAGGGCCATGCATTT	749	710
A020	EK3X9LI02F9G0Q	ACTCAAATCTGTCTCAGAATGGC	AATAGATTGCACCTGCCCAC	737	694
A021	EK3X9LI01CTJZY	TGACGTCAGGAGTCTCTCCC	ACCTGAAACACCCAATCAGG	749	709
A022	EK3X9LI02H02GU	ACACACACCTTTCCTGCACA	CTCTGCCATTGGCTCAGTCT	748	708
A023	EK3X9LI02GCTKJ	CAAGAGGCAAGAGGTCATTAAA	TCTGGCATTTCCCTGTCAAT	750	708
A026	EK3X9LI02FFWKW	AAACACACTCTTCCAAGCCC	CAGGCAGTCTGTCTCCTTGG	748	708
A028	EK3X9LI02IGAJD	GCAGTGTTTCCTCGATTTCC	CTGAGCCAACAGGAGAGGTC	750	710
A030	EK3X9LI02HH3QY	ATAGTGGTGCTGCTGCCTTT	GTCTCGGGTCAACGAAGACA	750	710
A032	EK3X9LI02G4ANS	GAACGGAGATTGTCGGAGG	CTTATTCTTGCTTGCCGGAC	748	709
A034	EK3X9LI01AEV4R	GGTCAGGGTTTGAGTTGGTC	GGAGGCAGATAGGAGAGGCT	742	702
A035	EK3X9LI01A6LR5	TGGACCAATGAGCGACATTA	TCACACGACAACTAAGAGCCC	748	707
A036	EK3X9LI01AHAIX	CATTGACAATCGTCTGAGCTTT	TGTCGTCTTGGTGGATTGAC	748	706
A038	EK3X9LI02IMKVX	ACCTATCTGGGAAATGCTCG	AACAATTGTGAGGACGGCTC	740	700
A039	EK3X9LI02IRFTU	TCATCAATACAACTGCCGCT	AAAGGCCTCGGGTAAGAGTC	750	710
A040	EK3X9LI02JSOT0	GGGTTGTAGGTAAGGGCCAC	ATTCCCAGGTCAGGACAGC	750	711
A041	EK3X9LI01AIALH	ATGTGGGAAACATGTCATCG	TTCGACATACTGGGACAGCA	747	707
A043	EK3X9LI02FIY9W	ACCCAGCTTCTCTGAGCAAA	GGGTCTGCTGAGATTTCTTCC	748	707
A044	EK3X9LI02GFDDD	CTTGGAAACCCTATGGAGCA	CAGCAGGAGCTAAGGAAACC	750	710
A048	EK3X9LI02GRG6Q	TTAACATGCTTGGTCCCTGC	AGGTCAGTCCCACAGTCCC	750	711
A049	EK3X9LI02HZ5M7	GCGAAGCATGGTTTGATAGC	ATATACCACGGACTGCCAGC	746	706
A051	EK3X9LI01AX9UU	TAGGGAGAGCCCTTGTCCTT	AAGCGCTAGAGCCAATGAGA	750	710
A052	EK3X9LI02G8P4Z	ACTTCCGGCAATAGAGCCTT	GGAGGGCTGGGTTTCTCTTA	747	707
A054	EK3X9LI01E21TS	TCTGGGATCATCTCCCTCTG	CGACTCGATGGCACTAGGTT	750	710
A055	EK3X9LI02JORZ9	GAACCTTTACATAGATCAAGAGGCA	TGAAATCCTTGAAAACTTCAATCTT	391	341
A057	EK3X9LI01DC6HA	CCTGGAGCATGAGAAACTGG	AATGTGTGAATGGAAACGGAG	750	709
A059	EK3X9LI01BDI18	TGGAAGGAGCAGTCAAGAAA	GCCATCAAGGAAATGCAAAT	750	710
A061	EK3X9LI02HQFHE	TTTCCTTTAGCCCTTTCCTTT	GGGTCTCAGATGAGTAAGGCA	750	708
A062	EK3X9LI02JWOIR	ATTCCAACTTCCAGTGCAGG	TTGGTCCTCCTCACTGATCC	750	710
A063	EK3X9LI01BCKUW	TTGGAAGAGCTCGCTTGTTT	TTGCTTTCTTTATGTTGAGGTG	750	708
A064	EK3X9LI02HHRV7	TTGTTCCAACAGCCACTCAT	ACTCTGGCATTGAAACAGCA	750	710
A070	EK3X9LI01AG83P	TGAACGCTAACACTGCATACG	CAAAGAAGAGAGCGCACAGG	750	709
A072	EK3X9LI02JM0Q8	AGGGATTCTTCCAGAGGAGC	CCTGCTTTGCTCAGAGGACT	750	710
A075	EK3X9LI01DSVG0	TGAGTGACACACGTTAATCACAA	TCTTTGGCTGAAGGGTGAAC	750	707
A078	EK3X9LI01C26GN	GGAGTTGCACTGGCTGATTT	TGTGTGAAGCCTTCCTTGTG	746	706
A079	EK3X9LI02H07QM	ACTCAGAGTGGTCGGAGCTG	ACTTCACACCTTGGAGTGCC	746	706
A081	EK3X9LI01DKF6W	GGACTGAAGATCCCAACCTG	CATGAGCTCCACCCACTCTT	750	710
A082	EK3X9LI02GF929	AACCACCAGTCTTTGTGACAGTT	CAGCTTCACTGCGGTTACAC	750	707
A088	EK3X9LI02JBXZW	TGAAAGAAGAGGCACCAACA	CCAGCCCTCAAACTGTTCTT	749	709
A090	EK3X9LI01AVFGZ	CAGGCAGGAGGAGCTATGAG	GCTGACAAATGGGAAGCAAT	750	710
A091	EK3X9LI02HYKZI	AGGTTGGCCATTAGGCTAGG	TAACGCATCCCTGGGTTAAG	745	705
A092	EK3X9LI02GY7B7	TTCAGAGAACGAACACATCCA	CGATGACATGTTTCCCACAT	720	679
A093	EK3X9LI02F4X94	CCTCCCAGGGACTTCTCCTA	AAAGGGTTGGAGGATCAAGG	743	703
A094	EK3X9LI01ET8NT	AGTCGGTCTGGAACAACAGC	TGTGCCAAGGACTGTGCTAA	749	709
A097	EK3X9LI02JWQH1	CGTCCTCTTCTGAATCTGGC	CTCCTTTGAAGCAAGGATGG	750	710
A099	EK3X9LI02IKMWQ	TGGGAGTGAGGATGAAGCTC	TTGGTTGGTCCGGAGTGTAT	750	710
A101	EK3X9LI02IYRPJ	CCCTACTATATTGTCTGTCGGG	TAACGGCCCATTAGTCCTCA	750	708
A102	EK3X9LI01EAN2W	GTTGCCATGAGATGGAACCT	CTGCTTGCCTTGATGTGATG	746	706
A103	EK3X9LI01DG8OA	CACGTCCATAAACCACTGGA	CAGTAGGCAACCCGTGATGT	750	710
A104	EK3X9LI01D826E	AGTTCTCTTGATCCTGGCCC	TGCATCAATTGGTCTCAACC	737	697
A106	EK3X9LI01DD6LB	GTGAAGCAGGGTGACAAGGA	TGGTTAACTGAAAGGTGGATGA	748	706
A107	EK3X9LI02JTIFO	TGTTTCTGGTGTCTCGGATG	CCCAACTGAACCAGAAGGAG	746	706
A111	EK3X9LI02HJ5E6	GATGTGGCAGTCTGCTTCTG	CTGGCCTCCAGTCTCTCACT	747	707
A113	EK3X9LI02G11JT	TAGGAGGTAACTTGCCACGG	CCAGATGAACTGAGAGCTTGG	750	709
A115	EK3X9LI01ANZ5L	ACCAGATCTTGTCTGCCCAT	AGCGATGTTTCTCCACCCAT	750	710
A118	EK3X9LI02ITWWD	CCTGAGGTAGACCCTGGTGA	TTCACCTTTGGAGCTCTGGT	743	703
A120	EK3X9LI01BVY86	GCTGACCCTCACTCAGACCT	AATTCCGCTGCTGCTTTCTA	747	707
A121	EK3X9LI02F5KTB	CACACAATCTGATGTGGCCT	GCAGTGTTTGCGTTCAGCTA	749	709
A124	EK3X9LI02IJEMP	TGGGCACTGATCAATAAGGA	ATACGCCTGGACATCTGCAA	750	710
A125	EK3X9LI01EHT4U	GAGTAGGCTGTGTGGCTGG	GAGTTTGGTGTTTACTGGGCA	750	710
A126	EK3X9LI01EBDG8	CACCAGGGCTTCTGACACAT	TTGCAAACATAGTGGAGGGA	737	697
A127	EK3X9LI01BJV3L	TGTCAAGGTGGATCCCAAAC	CCCTGTTGATGTAGTGCTGC	750	710
A129	EK3X9LI02IM99N	AGCTCCACCACGTCCACTAC	CTCTAGGCCCTCTGTCAGGC	750	710
A130	EK3X9LI02H1ZVD	GTCAGCCACCAGGGATTCT	GGACCAGGACCTGATTGAGA	749	710
A131	EK3X9LI01C1TEE	GAAGTCCCTTCCACAGGTGTT	GCTGAGCTTTCTCCTGAACC	749	708
A133	EK3X9LI02F6FN8	CAGCTTTCAGGTAGCATGGG	TTCCTCCCACCTTCTGGTC	750	711
A137	EK3X9LI01EDFZR	GGGACCGATTGAGATGTTTC	TCCATACTGGAGGCTGTGGT	750	710
A138	EK3X9LI02GH9IC	TGCAGTCATCTCAAAGGGCT	AGAGAGAAGGCGCTTGCATA	750	710
A139	EK3X9LI01DWNEV	CCCAGCAGTCCAGTGGTTAC	AGGCAAGAGATGCTGAGTGG	747	707
A141	EK3X9LI02GMTFT	TGTTTGTTGTTCAGGGTTCG	GGATGGGCTCCATCATTAAG	746	706
A144	EK3X9LI02GYYTB	CTCGGCTGCTAACCGAAATA	GATGGCAACGAGCTTGGTAT	749	709
A146	EK3X9LI01E5Q3R	GGAAACTGAGTTCAGCTCCAT	AGCTCCCTACCTCCTTGCTA	750	709
A149	EK3X9LI01CEAIR	GCACACCTGGATTAACCGAA	ATACTCCCAACTGCTCTCCC	750	710
A151	EK3X9LI01EOTRO	CTCCATGGTGATTGGGAAAG	TGACACATACCTGCCAGACG	745	705
A152	EK3X9LI01EPXIX	ATGATGGGCTCAGGCATAAC	GGCTAGTGACGAGGCAACTT	749	709

A.1 – long

target ID	read ID GenBank SRA010805	F-Primer 5'-3'	R-Primer 5'-3'	product length in bp amplicon	product length in bp w/out primer
A153	EK3X9LI02INS8N	AGTCAGAACCGACTCAACGG	TGTGTCGTCTTAATCTCCTCCA	750	708
A154	EK3X9LI02FZCNA	GAAGGACTACAGTGGAGGACTGA	CCACCATTCGCTAGAACCTG	749	706
A158	EK3X9LI02GM7LC	TTAGGTGCCGTTGAGTTGGT	TTGCTTGCCAAGAGTGAAGA	750	710
A160	EK3X9LI02IKVA4	TCCCATGTGGTGTCACAAAC	GCTCCTCCATGTGGAATACG	750	710
A164	EK3X9LI02FL5QN	CAGAGTCTGCTGTGTGTGGC	AAGCTTCCAATAGACATTTGCT	750	708
A165	EK3X9LI02GFCPO	GGTCTTGTGGCCTGACATTT	CTCTAGGAAACACGCCAACA	750	710
A166	EK3X9LI02JIUKM	ACACACATGGGTGGGATTCT	CCCACCAGCAATGAACAAG	750	711
A168	EK3X9LI02GZ29S	CTGTCGAGTCGGTTTGGACT	CACAACCTTGCTTGCTGAAA	750	710
A169	EK3X9LI01CFYE6	AGGTTAGTGGAGCTGGCAGA	TCGTCACCAACACATGTTCA	746	706
A170	EK3X9LI02I8SIM	CCCACGTCCATAAACCATTG	ATGTGGACAGGTTCGACACA	739	699
A171	EK3X9LI01CDP0M	TGACCTGTGACACTGCAGAC	CTCTGGCTCTACTGGTTGCC	750	710
A177	EK3X9LI01DNN7S	CACATCGCAGATCAGTGGAC	CAGTGTGGCCATGAAGACTG	750	710
A179	EK3X9LI01DLN0U	CTCTGTCCTGCTTGTCTCCC	TGGAAACAGAAGCCAACAGAT	749	708
A180	EK3X9LI02GECMC	AGAAGGGCCACAGAGTCTCA	AAATCGAATGCTTCAGGGTG	750	710
A181	EK3X9LI02JOYBY	ATTGGAACACTTGCCCAGAC	CCCAGTCTCTGAGCCACG	655	617
A184	EK3X9LI02IDCDD	CCCAGTGCCTGTCTCTGAGT	TCGGACTCTCTCAGTCCCTC	750	710
A186	EK3X9LI01BIKBU	GACACTGCCTGGTGAAGGAC	AGTCTGGAAGCTCCACCAAA	745	705
A187	EK3X9LI02FRKY4	GGGACCCAAACAAGCAAATA	TCCTCATGGCTATCACAAGC	749	709
A188	EK3X9LI01DOVZU	CCAATCCTGATGCTCTGTTC	CAAGTTGAAAGAACGGAAGAAA	750	708
A190	EK3X9LI01A0OZO	TGTTTGTGCCACCTAGGCTT	AAGGCACCACAGAAGAAAGG	748	708
A192	EK3X9LI01BZ6AP	CCCTTCTCTCTTCTTGCTGC	TGCTCAGCCTTCTGAAAGAAA	750	709
A195	EK3X9LI01D9AK9	GTCGGAGGCGTAGGAAGTG	TTGATGTTCACAGGATTGGG	750	711
A200	EK3X9LI02I9BMY	CTTTCTGTCCGTCCCTCTCA	TGTCTCAGCTCCTGTAGCCC	749	709
A206	EK3X9LI01EYOLN	ATAATTCCGCTGCTGCTTTG	CTTGCTGACCCTCACTCAGA	750	710
A207	EK3X9LI01AG3ZZ	CTGAAATGCTTTGATGGCAA	AGAACGCCAAAGACACAAGG	749	709
A211	EK3X9LI02HVV0D	TCAGCTATTGTGAAGGCTCG	CACACTATCTCGTCAGGGCA	749	709
A213	EK3X9LI02GE17J	TGAGTTGCTGGAGACCAGG	GGCCAGGTAGGTGAAGTCAA	750	711

Target and primer information used for sequencing error rate estimations of the mastodon shotgun sequencing: read ID corresponds to the read in the short read archive SRA010805, PCR product length with and without primers is given. Targets (see round A.1) were divided into two targets, primer set α uses the already existing forward primer from round A.1 and primer set β utilizes the existing reverse primer from round A.1, the corrsponding (sometimes degenerated) primer per pair was designed using all availabe sequence information (modern elephants, mastodon and mammoth).

target ID	primer set	read ID GenBank SRA010805	F-Primer 5'-3'	R-Primer 5'-3'	product length in bp amplicon	product length in bp w/out primer
A001	α	EK3X9LI01BLRFR	TGGTCTCGAAGTCAGACAAGAG	TGTCCAGAATTGCTTCCTTG	96	54
A002	α	EK3X9LI02F3RBB	GCATTTTGATTGCATGTTTGA	ATATTATCCAAATTTATAAACCAACCA	112	64
A003	α	EK3X9LI02HKE1R	TATAGTTCCAGGCTCCACGG	TGGAGTATTTCAGGCAGAGAGA	122	80
A007	α	EK3X9LI01C7BYB	TTTTCCTGTGTTTGTGCATGT	GGRTRRCTCAAGGAGGCAAAA	123	81
A009	α	EK3X9LI01C7XBW	TTTCTATTCTCCATGAATGAACCA	TGGCAATTCACAGAAAACTCA	122	77
A011	α	EK3X9LI01AQJCF	GCTTCATTCTGTTGTGCGTG	AGCTGGCTTCCTGTTGTTGT	76	36
A012	α	EK3X9LI01BCGQI	CACTCCCAGCACACCAGAC	AGGTGCAGTTTGCCGAGTAG	103	64
A014	α	EK3X9LI01AIIP7	CTTTCTCTGGGCAAAGTCCA	YATYCTTGYGYTTGATGTTR	101	61
A015	α	EK3X9LI02JWLAQ	GAGTTACCGAGAGGGGGAAR	TGCAAACAAAAGACTCAAAGGA	128	86
A016	α	EK3X9LI01EN54P	TCGGACAACAGGACCCTTAC	RGATGAATGGGTCACTACGG	109	69
A017	α	EK3X9LI02I9G0E	CTGCTCTGCCACCAATCTTC	GGCTGTGAGGAAGCCAGA	100	62
A019	α	EK3X9LI01C0K93	AACCACCATTGTCCACTTTG	GGCAGTTGAGAACTTTGCACT	103	62
A020	α	EK3X9LI02F9G0Q	GAATAACATCAGACGGTGCG	AGCCACCCCCAYCTGACT	103	65
A021	α	EK3X9LI01CTJZY	GCTTACCACACACAATCCCC	TGTGAGTTTTGACCYATTAGAGTG	110	66
A026	α	EK3X9LI02FFWKW	CCCAGCACTTAACAAATACTTAAAA	AAACACCAAAGCTGTTAACCA	101	55
A033	α	EK3X9LI01B83XL	TGACTGACAACAGCAAGGCT	GGAAGCWGCAGTCAGGAAAT	118	78
A034	α	EK3X9LI01AEV4R	GGTACCAAACCCTTGCCAT	RCCAGGGCTTAGCTAGAGAG	108	69
A036	α	EK3X9LI01AHAIX	CATGAAAAATGCTGTTAAAACCC	TCTGTTTCACATRGTTTCTTGG	112	67
A038	α	EK3X9LI02IMKVX	AGGGATCAAATGTTGTTGTTCA	CATTTCTGAGCTGCGTTGTG	101	59
A040	α	EK3X9LI02JSOT0	AAGGCCACTCTTGGCTTTCT	GCAGAAGGATGRGTCTCARAGC	128	86
A042	α	EK3X9LI01BE9T9	GACATCCAAACATTTTCAGGC	TCTGGRATGAAGGGACATCA	112	71
A044	α	EK3X9LI02GFDDD	GTGGGTCTACAAATCACCGC	GCTTGGCACCATTTGCTTAG	107	67
A046	α	EK3X9LI01A3SP9	GCTGCAGCTGCTTTGAATAA	GTGCCAGTTCCAAAGAAAGG	111	71
A047	α	EK3X9LI01AZEP9	GGGACTCCTAGGACAATCCAG	GTCCCACCTGCCCCTGTAG	99	59
A048	α	EK3X9LI02GRG6Q	AGGGGAACCATGTCCTTGAT	GAGAGTCTGTGCTGTTTGGACA	113	71
A049	α	EK3X9LI02HZ5M7	TGTTGTTGTTAGGTGCTGTGG	CAAYGATTGTGAGKATGGTK	101	60
A050	α	EK3X9LI02JDDAK	AGGAATCGGGTGAGTGAGTG	CTTCCCAGGACAGGACACTG	99	59
A051	α	EK3X9LI01AX9UU	TTGTGATCAGAGTTTTGCGG	GGRGTGAGGTCCCCCAAG	126	88
A052	α	EK3X9LI02G8P4Z	GTCTCTTCTGATGGTGGGCT	ATGACTGGTGGGGRAAGTT	99	60
A054	α	EK3X9LI01E21TS	CGACTCAACACATCAAGCAAA	CAACTCAAACTCTTTACAAAAGCAA	125	79
A056	α	EK3X9LI02JVJWB	TTTGGGTTAAATGGACAGCC	TTGGTAGCTGGTGGTATTTGAA	98	56
A057	α	EK3X9LI01DC6HA	AGCTCCAAGACCTATGCTGG	CCTYYAAACCCATCSAGAAG	125	85
A061	α	EK3X9LI02HQFHE	TGGTTAAGAGCTCAGGCTGC	GGGCCCATAAGACAGGGTAG	101	61
A064	α	EK3X9LI02HHRV7	TAGCAAACTCTTGGCTGCCT	TTYAYTGTGCGTTAGGGTTG	101	61
A065	α	EK3X9LI01C7PXN	ATGGTGATGGGAGCTTTGAC	CAACATTTGCCAATTCAACG	108	68
A066	α	EK3X9LI01C28PT	TCTAGGGACTGGACCCTCCT	TGCCAGAAGAATAAACAGGTCT	108	66
A069	α	EK3X9LI02II2C9	TCAGTTAACACCCACATCTGTCT	CTTSCCTTCCSTTCCCTTC	135	93
A071	α	EK3X9LI02HHLB0	CCAATGCCATTAAAATCAGAAA	AGGCTCTACAATGGGTGAGTG	108	65
A075	α	EK3X9LI01DSVG0	CACCCTGAGGGACTGAGTTG	ACYAGATGCCACTCACCAA	107	68
A076	α	EK3X9LI02FHZ2J	CAAATTTCACTGGCATTCATCA	ACATTTCTYTTTCCTCTGACTTCC	97	51
A077	α	EK3X9LI01CPQ0Z	GGACTGGAAAGCAAAACCCT	TGGCTTTAGCKGGAAAGATT	125	85
A078	α	EK3X9LI01C26GN	GCGGATTCCTGTGTATGGTT	CAGGYAGAGGCGAGACAT	108	70
A082	α	EK3X9LI02GF929	AAAACCAGAGCCAAGTGGTG	AGAGCTCCTGGGYGACAAT	108	69
A083	α	EK3X9LI01D541S	TCTTGCTTTGGGACGGATAC	AGTGGCCGAGRTGGTTTG	120	82
A084	α	EK3X9LI01BVALO	GCATGTGTGAGCTCTTCCCT	GCAGTAAAGSCTGGGAAGG	117	78
A085	α	EK3X9LI02FPCGC	GCTCCACTGTGTGAGTCATCAT	GTAGCAGGGCAGGAGAGGAG	113	71
A086	α	EK3X9LI01BLH35	ACACCTCTGAGCTTCCCTGA	GGGSCAATCCTTAYTGTGTG	95	55
A087	α	EK3X9LI02HII8H	AATTTGCAAGGCTCAGTGCT	GGGGTTTCACGCTTAGAGG	112	73
A088	α	EK3X9LI02JBXZW	TGCTGTTGTCAAAGCCTGAG	CTTGAAGGGGCTTTGAGAGA	107	67
A090	α	EK3X9LI01AVFGZ	TACATTCGCCTACCTCCCTG	TCTGTGTTACAGACTGCTTTTGG	110	67
A001	β	EK3X9LI01BLRFR	AGGGCTTTTGTAACAGTGAACAC	TCAGGACTTGCCTGTTCAGTT	140	96
A002	β	EK3X9LI02F3RBB	CAATTTCTTCATCTTTGGCTTT	AAATTTTGAGATCTGTCCCGAA	135	91
A003	β	EK3X9LI02HKE1R	AGGGAGAAGTCTTGGGACAG	ATAAGCAGTCAAGAGCCCGA	121	81
A007	β	EK3X9LI01C7BYB	CCTTGACCACTGATAACTGGAG	AGCTAATGGAAGAAATGATGAAGT	131	85
A009	β	EK3X9LI01C7XBW	AKTCCCCYRATTACTACTAAGATTGT	CAAACAAATTGAGAAAACAGTGC	108	59
A011	β	EK3X9LI01AQJCF	AGGCAGGGCTGACTCSAT	ACTCTGCTCTGTGATGCTGG	106	68
A012	β	EK3X9LI01BCGQI	AAAGGCTCTGCCCGTCA	GAGTGGGTTCATTGTCACCA	124	87
A014	β	EK3X9LI01AIIP7	GYCCTTGAAWRTCACTGMTT	CATCCTCCCAACCTTTTCAA	90	50
A015	β	EK3X9LI02JWLAQ	AACAGAAGTATGGCTTCTTTGG	TCATCTCAGTGACCAAACTTGAA	110	65
A016	β	EK3X9LI01EN54P	TGACACAGGACCCTATGAGTG	CTGTGCCATGTGGAAACAAA	110	69
A017	β	EK3X9LI02I9G0E	GGAGAGAGCCCTCTGTAGCC	CAGTCTGTGGGCATGTCTGT	123	83
A019	β	EK3X9LI01C0K93	GGAGGTCCAGGAGAGCTTTT	CAGCTGCCAGCAACAATG	109	71
A020	β	EK3X9LI02F9G0Q	GCCAGTGTGGTRGGTGACC	TAGCTGGAACTCTTGCCTGG	110	71
A021	β	EK3X9LI01CTJZY	AAAAATTTTGGTCACAACTCACTG	AGAAATGTACCATGAGCGGG	117	73
A026	β	EK3X9LI02FFWKW	TGATTGGTTATACGCTTCACTCA	AACTAACATGGTCAGCCTTGC	106	62
A033	β	EK3X9LI01B83XL	AARAGGTCTTTTGCAGCAGATT	GCAACATCATGATAAGTGGAGAA	119	74
A034	β	EK3X9LI01AEV4R	TCACCTCTCCCTCCTTAACC	GCTGTGTGGAGTTAGGGACC	120	80

target ID	primer set	read ID GenBank SRA010805	F-Primer 5'-3'	R-Primer 5'-3'	product length in bp amplicon	product length in bp w/out primer
A036	β	EK3X9LI01AHAIX	TCACACACACCACCCTGTTC	TCACTCTAATTCTTCATCCTTCCA	115	71
A038	β	EK3X9LI02IMKVX	TTCTCTGCYCTTCAAATTCA	TCTTCCCCATTTCTCAGAGG	94	54
A040	β	EK3X9LI02JSOT0	TGTGTGCTATCAGCCCTCAC	GTTTGCTTCAAAGCTGTGGG	125	85
A042	β	EK3X9LI01BE9T9	GTGAAGCTCTGTGAGGAGCA	TGGAAGGTGCTCAGTGCTTA	119	79
A044	β	EK3X9LI02GFDDD	AACCRGCCCTTCTGRTTY	CACAGTGAYTGAAAGCGGAA	92	54
A046	β	EK3X9LI01A3SP9	AATTTCCTCACTCTGTTGGATCA	TGATGCAGTGGTTGAGGTGT	116	73
A047	β	EK3X9LI01AZEP9	GCTRCRYGTGCTAGTGACTG	AAACTGCACCTGGCCTTATG	90	50
A048	β	EK3X9LI02GRG6Q	AATGTCCYACAAAGGAGCTG	GAACTGGACAGCCTGTGGTT	118	78
A049	β	EK3X9LI02HZ5M7	GCMACCCCATGTACAACAGA	GACCCTCAAGGAGATGGTTG	116	76
A050	β	EK3X9LI02JDDAK	AGGCTGTCATGGGGCTTG	CCACACTAAAAGGGACCCAA	107	69
A051	β	EK3X9LI01AX9UU	AGGGCCAGCTGCTTCAG	ACACTGCAGGGGCTCAAC	105	70
A052	β	EK3X9LI02G8P4Z	TGCCTTTCCGAGTATTCMCA	CATGTGGAGAACTTCCTTTGC	99	58
A054	β	EK3X9LI01E21TS	TCTCCCTGGTCTCATCATCA	TCCCTCAATTCCAACAGCAT	116	76
A056	β	EK3X9LI02JVJWB	TTGTGGTGAGAGCCTCCTTT	TTCCTGCAATAAAGATTGGC	100	60
A057	β	EK3X9LI01DC6HA	AAGCAAACAACARGCAGTCC	CTCGGCTGACTTTCACTTCC	115	75
A061	β	EK3X9LI02HQFHE	CAAAAGGCCRGGAATTCAGA	ACTCACTGCCATCGAGTCAA	110	70
A064	β	EK3X9LI02HHRV7	ATGTTCTTTCCTGGGCTGCT	CTGCAGTCAGTCCTTGCAGA	109	69
A065	β	EK3X9LI01C7PXN	TCACTAAATGCACMCCTGAAAAA	AGAAAACAGACTGCGTGCCT	103	60
A066	β	EK3X9LI01C28PT	CAAAGTCTTGCCATCCTCAC	TGCATAAAAAGATGGACAATGAA	127	84
A069	β	EK3X9LI02II2C9	TGGGTTCTTTGGCTAGGAAG	GCCTTCCTTTGCTTTCCTTT	117	77
A071	β	EK3X9LI02HHLB0	AAGGGTGGAGCATTCCTATG	TTATGAAAGTGAATTAGACACGAAA	120	75
A075	β	EK3X9LI01DSVG0	TGTAAAGAAAATGTTCTACATCCAA	TTTGCTACAGAAAAGATCAAAACA	108	59
A076	β	EK3X9LI02FHZ2J	GCACCATCCCTGTGATTCTC	AGATCAGCCCAAAGGGTTTT	124	84
A077	β	EK3X9LI01CPQ0Z	TGGRATGCTTGACTTCCAGA	GCTTAAGGCATTTGTGAAATCAT	101	58
A078	β	EK3X9LI01C26GN	TTTCTTGTTGCAAGTCCACTG	TGCTGAGAGTGTGTGTGTGC	113	72
A082	β	EK3X9LI02GF929	CTTTTGGCCCAATGGAAG	CAGATCTTGAGATGGGCCAC	106	68
A083	β	EK3X9LI01D541S	TTAACGCTCACAGCCTGCTA	ATGACGGGGCTTCTTTCTTT	118	78
A084	β	EK3X9LI01BVALO	AGGAAGGAGAAGAGCAGYCTTG	GCAGGATTCTTGCTTGCTTC	110	68
A085	β	EK3X9LI02FPCGC	CTCTAGCCCAGTGGCYTTC	TGCTGCAGAGACAGAAGGAA	115	76
A086	β	EK3X9LI01BLH35	AGGGTCTTGTCTRCCTTCCT	TGCTGTGTCGACTCCTCTTG	107	67
A087	β	EK3X9LI02HII8H	CATGAATTTCAAGAAGGAGATGT	CTGCCGGTTCTCTACTCAGG	115	72
A088	β	EK3X9LI02JBXZW	CATGTTGTCCCATCAGCTCTT	AACGGCAATCCCATTTTACA	120	79
A090	β	EK3X9LI01AVFGZ	GAACTCCAGTTAAATCCAATAGTAGTC	TGGGCAGGATTCAATAGGAG	128	81
