# Supplementary material for: Genomic DNA Sequences from Mastodon and Woolly Mammoth Reveal Deep Speciation of Forest and Savanna Elephants
Source: PLoS Biol. 2010 Dec 21;8(12):e1000564. doi: 10.1371/journal.pbio.1000564 (PMC3006346; doi:10.1371/journal.pbio.1000564)
Supplement: Dataset S3 — Input file (PHYLIP) for MCMCcoal. (0.10 MB PDF) [file pbio.1000564.s003.pdf]

```
347

 1 1 1 1 1
5 108

ASIAN2_Ema   GAAGGGCTTT TGTAACAGTG AACACACAGG ATCAAGKAAG CAATTCTGGA
             CAATTTTTCA ATCGGTTGAC AGGCATTTTC TTTCCTTATT TGGAATTAAG
             AGGGMATT
FOR_DS1535   GAAGGGCTTT TGTAACAGTG AACACACAGG ATCAAGGAAG CAATTCTGGA
             CAATTTTTCA ATCGGTTGAC AGGCATTTTC TTTCCTTATT TGGAATTAAG
             AGGGAATT
MAM1_A001_   GAAGGGCTTT TGTAACAGTG AACACACAGG ATCAAGGAAG CAAT-CTGGA
             CAATTTTTCA ATCGGTTGAC AGGCATTTTC TTTCCTTATT TGGAATTAAG
             AGGGAATT
MAST_A001_   GAAGGGCTTT TGTAACAGTG AACACACAAG ATCAAGGAAG CAATTCTGGA
             CAATTTTTCA ATCGTTTGAC AGGCATTTTC TTTCCTTATT TGGAATTAAG
             AGGGAATT
SAV1_SE210   GAAGGGCTTT TGTAACAGTG AACACACAGG ATCAAGGAAG CAATTCTGGA
             CAATTTTTCA ATCGGTTGAC AGGCATTTTC TTTCCTTATT TGGAATTAAG
             AGGGAATT

 1 1 1 1 1
5 103

ASIAN2_Ema   TGGGTTGCCT TTAGGGAGAA GTCTTGGGAC AGGCCCCTCC --TCGAGACA
             ATGTCCCTGG TTCACTTGAG CGCTCTCTCT GCCTGAAATA CTCCAACTCT
             TGA
FOR_DS1535   TGGGTTGCCT TTAGGGAGAA GTCTTGGGAC AGGCCCCTCC --TCGAGACA
             ATGTTCCTGG TTCACTTGAG CGCTCTCTCT GCCTGAAATA CTCCAACTCT
             TGA
MAM1_SP134   TGGGTTGCCT TTAGGGAGAA GTCTTGGGAC AGGCCCCTCC --TCGAGACA
             ATGTCCCTGG TTCACTTGAG CGCTCTCTCT GCCTGAAATA CTCCAACTCT
             TGA
MAST_A003_   TGGGTTGCCT TTACGGAGAA GTCTTGGGAC AGGCCCCTCT CCTCGAGACA
             ATGTCCCTGG TTCACTTGAG CGCTCTCTCT GCCTGAAATA CTCCAACTCT
             TAA
SAV1_SE210   TGGGTTGCCT TTAGGGAGAA GTCTTGGGAC AGGCCCCTCC --TCGAGACA
             ATGTCCCTGG TTCACTTGAG TGCTCTCTCT GCCTGAAATA CTCCAACTCT
             TGA

 1 1 1 1 1
5 97

ASIAN2_Ema   AAATAAAGAC TTGATGCCTT GACCACTGAT AACTGGAGGC CAGGTCTTGG
             AAGATTTCAT TTCATTATAA TATTTTATTT TGCCTCCTTG AGTTATC
FOR_DS1535   AAATAAAGAC TTGATGCCTT GACCACTGAT AACTGGAGGC CAGGTCTTGG
             AAGATTTCAT TTCATTCTAA TATTTTATTT TGCCTCCTTG AGCTATC
MAM1_SP134   AAATAAAGAC TTGATGCCTT GACCACTGAT AACTGGAGGC CAGGTCTTGG
             AAGATTTCAT TTCATTATAA TATTTTATTT TGCCTCCTTG AGTTATC
MAST_A007_   AAATAAAGCC TTGATGCCTT GACCACTGAT AACTGGAGGC CAGGTCTTGG
             AAGA-TTCAT T--A--AAAA TATTTTATTT TGCCTCCTTG AGCCACC
SAV1_SE210   AAATAAAGAC TTGATGCCTT GACCACTGAC AACTGGAGGC CAGGTCTTGG
             AAGATTTCAT TTCATTATAA TATTTTATTT TGCCTCCTTG AGCTATC

 1 1 1 1 1
5 102

ASIAN2_Ema   GTAAAAATAT CACAATGTGT ATTCCCCTGA TTACTACTAA GATTGTATAT
             CCTTTCTTAT ATTTATTAAT CATTTGAGTT TTCTGTGAAT TGCCAGATTA
             TG
FOR_DS1535   GTAAAAATAT CACAATGTGT ATTCCCCTGA TTACTA---A GATTGTATAT
             CCTTTCTTAT ATTTATTAAT CATTTGAGTT TTCTGTGAAT TGCCAGATTA
             TG
MAM1_SP134   GTAAAAATAT CACAATGTGT ATTCCCCTGA TTACTACTAA GATTGTATAT
             CCTTTCTTAT ATTTATTAAT CATTTGAGTT TTCTGTGAAT TGCCAGATTA
             TG
MAST_A009_   GTGAAAATAT CACAATACGT AGTCCCCCAA TTACTACTAA GATTGTACAT
             CCTTTCTTAT ATTTATTAAT CGTTTGAGTT TTCTGTGAAT TGCCAGATTA
             TG
SAV1_SE210   GTAAAAATAT CACAATGTGT ATTCCCCTGA TTACTACTAA GATTGTATAT
             CCTTTCTTAT ATTTATTAAT CATTTGAGTT TTCTGTGAAT TGCCAGATTA
```

TG

1 1 1 1 1
5 95

```
ASIAN2_Ema   GGTTTCCATG AGGCAGGGCT GACTCSATGG AAGCTAACAA CAACAGGAAG
             CCAGCTGGCA GGGGYGCCTG GGCAGTGTAG CCTGGAAAGC CCTGT
FOR_DS1535   GGTTTCCATG AGGCAGGGCT GACTCCATGG AAGCTAACAA CAACAGGAAG
             YCAGCTGGCA GGGGCGCCTG GGCAGTGTAG CCTGGAAAGC CCTGT
MAM1_SP134   GGTTTCCATG AGGCAGGGCT GACTCGATGG AAGCTAACAA CAACAGGAAG
             CCAGCTGGCA GGGGTGCCTG GGCAGTGTAG CCTGGAAAGC CCTGT
MAST_A011_   GGTTTCCATG AGGCAGGGCT GACTCGATGG AAGCTAACAA CAACAGGAAG
             CCAGCTGGCA GGGGCGCCTG GGCAGTGTAG CCTGGAAAGA CCTGT
SAV1_SE210   GGTTTCCATG AGGCAGGGCT GACTCCATGG AAGCTAACAA CAACAGGAAG
             CCAGCTGGCA GGGGCGCCTG GGCAGTGTAG CCTGGAAAGC CCTGT
```

1 1 1 1 1
5 99

```
ASIAN2_Ema   CAATGACAAA GGCTCTGCCC GTCAGAGGGA CCCTGCAGCC CGTGCCAGTG
             TGCACGCCCC GGCCTACTCG GCAAACTGCA CCTCAGCTGC CGGCCAGGG
FOR_DS1535   CAATGACAAA GGCTCTGCCC GTCAGAGGGA CCCTGCAGCC CGTGCCAGTG
             TGCACGCCCC GGCCTACTCG GCAAACTGCA CCTCAGCTGC TGGCCAGGG
MAM1_SP134   CAATGACAAA GGCTCTGCCC GTCAGAGGGA CCCTGCAGCC CGTGCCAGTG
             TGCACGCCCC GGCCTACTCG GCAAACTGCA CCTCAGCTGC CGGCCAGGG
MAST_A012_   TAATGACAAA GGCTCTGCCC GTCAGAGGGA CCCTGCAGCC CGTGCCAGTG
             TGCACGCCCC GGCCTACTCG GCAAACTGCA CCTCAGCTGC CGGCCAGGA
SAV1_SE210   CAATGACAAA GGCTCTGCCC GTCAGAGGGA CCCTGCAGCC CGTGCCAGTG
             TGCACGCCCC GGCCTACTCG GCAAACTGCA CCTCAGCTGC CGGCCAGGG
```

1 1 1 1 1
5 94

```
ASIAN2_Ema   AGCTCCTCCT CAGTGAAGAC CACAACCATG CCCTTGAATG TCACTGCTTC
             CTACAACATC AAGCGCAAGA ATG-CCCAGT CTTAGAACTA TCAC
FOR_DS1535   AGCTCCTCCT CAGTGAAGAC CACAACCRTG TCCTTGAATG TCACTGCTTC
             CTACAACATC AAGCGCAAGA ATG-CCCAGT CTTAGAACTA TCAC
MAM1_SP134   AGCTCCTCCT CAGTGAAGAC CACAACCATG CCCTTGAATG TCACTGCTTC
             CTACAACATC AAGCRCAAGA ATG-CCCAGT CTTAGAACTA TCAC
MAST_A014_   AGCTCCTCTT CAGTGAAGAC TACAGCCATG TCCTTGAAAA TCACTGATTC
             CTATAACATC AAACACAAGG ATAACCCAGT CTTAGAACTA TCAC
SAV1_SE210   AGCTCCTCCT CAGTGAAGAC CACAACCATG TCCTTGAATG TCACTGCTTC
             CTACAACATC AAGCGCAAGA ATG-CCCAGT CTTAGAACTA TCAC
```

1 1 1 1 1
5 95

```
ASIAN2_Ema   CAAGGAATGA CACAGGACCC TATGAGTGTA GAACCTGGAA CCAAGTGAAT
             GCCCGCCGTA GTGACCCATT CATCCTGAAT GTTCTCTGTG AGTAA
FOR_DS1535   CAAGGAATGA CACAGGACCC TATGAGTGTA GAACCTGGAA CCAAGTGAAT
             GCCCGCCGTA GTGACCCATT CATCCTGAAT GTTCTCTGTG AGTAA
MAM1_SP134   CAAGGAATGA CACAGGACCC TATGAGTGTA GAACCTGGAA CCAAGTGAAT
             GCCCGCCGTA GTGACCCATT CATCCTGAAT GTTCTCTGTG AGTAA
MAST_A016_   CAAGGAATGA CACAGGACCC TATGAGTGTA GAACCTGGAA CCAAGTGAAT
             GCCCGCCGTA GTGACCCATT CATCTTGAAT GTTCTCTGTG AGTAA
SAV1_SE210   CAAGGAATGA CACAGGACCC TATGAGTGTA GAACCTGGAA CCAAGTGAAT
             GCCCGCCGTA GTGACCCATT CATCCTGAAT GTTCTCTGTG AGTAA
```

1 1 1 1 1
5 99

```
ASIAN2_Ema   TTAGAAGTGA GGCGCTACCC AGGAAGGAGA GAGCCCTCTG TAGCCCAGGA
             GAGTTCTGGC TTCCTCACAG CCCCTYATCC TCCTTCAAAC CTCAGCTTT
FOR_DS1535   TTAGAAGTGA GGCGCTACCC AGGAAGGAGA GAGCCCTCTG TAGCCCAGGA
             GAGTTCTGGC TTCCTCACAG CCCCTCATCC TCCTTCAAAC CTCAGCTTT
MAM1_SP134   TTAGAAGTGA GGCGCTACCC AGGAAGGAGA GAGCCCTCTG TAGCCCAGGA
             GAGTTCTGGC TTCCTCACAG CCCCTCATCC TCCTTCAAAC CTCAGCTTT
MAST_A017_   TTAGAAGTGA GGGGCTACCC AGGAAGGAGA GAGCCCTCTG TAGCCCAGGA
             GAGTTCTGGC TTCCTCACAG CCCCTCATCC TCCTTCAAAC CTCAACTTT
SAV1_SE210   TTAGAAGTGA GGCGCTACCC AGGAAGGAGA GAGCCCTCTG TAGCCCAGGA
             GAGTTCTGGC TTCCTCACAG CCCCTCATCC TCCTTCAAAC CTCAGCTTT
```

```
 1 1 1 1 1
5 91

ASIAN2_Ema   TGGGTGGAGG TCCAGGAGAG CTTTTAAAAG GCCAAGGTTA TAGGCTCCAT
             TAGTGCAAAG TTCTCAACTG CCTCAGAATA CAGTCTGAGG A
FOR_DS1535   TGGGTGGAGG TCCAGGAGAG CTTTTAAAAG GCCAAGGTTA TAGGCTCCAT
             TAGTGCAAAG TTCTCAACTG CCTCAGAATA CAGTCTGGGG A
MAM1_SP134   TGGGTGGAGG TCCAGGAGAG CTTTTAAAAG GCCAAGGTTA TAGGCTCCAT
             TAGTGCAAAG TTCTCAACTG CCTCAGAATA CAGTCTGAGG A
MAST_A019_   TGGGTGGAGG CCCAGGAGAG CTTTTAAAAG GCCAAGGTTA TAGGCTCCAT
             TAGTGCAAAG TTCTCAACTG CCTCAGAATA CAGTCTGAGG A
SAV1_SE210   TGGGTGGAGG TCCAGGAGAG CTTTTAAAAG GCCAAGGTTA TAGGCTCCAT
             TAGTGCAAAG TTCTCAACTG CCTCAGAATA CAGTCTGAGG A

 1 1 1 1 1
5 106

ASIAN2_Ema   GACTAGCATT TCTCAAAATG TGAGTTTTGA CCCATTAGAG TGTCATGAAA
             TCAACTCAGT GAGTTGTGAC CAAAATTTTT AGAACAAAAT -GGAATTTAG
             CAGAAA
FOR_DS1535   GACTAGCATT TCTCAAAATG TGAGTTTTGA CCCATTAGAG TGTCATGAAA
             TCAACTCAGT GAGTTGTGAC CAAAATTTTT AGAACAAAAT -GGAATTTAG
             CAGAAA
MAM1_SP134   GACTAGCMTT TCTCAAAATG TGAGTTTTGA CCCATTAGAG TGTCATGAAA
             TCAACTCAGT GAGTTGTGAC CAAAATTTTT AGAACAAAAT -GGAATTTAG
             CAGAAA
MAST_A021_   GATTAGCATT TTTCAAAATG TGAGTTTTGA CCCATTAGAG TGTCATGAAA
             TCAACTCAGT GAGTTGTGAC CAAAATTTTT AGAACAAAAA CGGAATTTAA
             CAAAAA
SAV1_SE210   GACTAGCATT TCTCAAAATG TGAGTTTTGA CCTATTAGAG TGTCATGAAA
             TCAACTCAGT GAGTTGTGAC CAAAATTTTT AGAACAAAAT -GGAATTTAG
             CAGAAA

 1 1 1 1 1
5 113

ASIAN2_Ema   TCATTGTCTG GATTGCTATT TTATTACCCA TTAAGTGGTT AACAGCTTTG
             GTGTTTAAAA AAGTAATGAG TGAAGCGTAT AACCAATCAA AAA-TGATTG
             TGAGCACCAG GGA
FOR_DS1535   TCATTGTCTG GATTGCTATT TTATTACCCA TTAAGTGGTT AACAGCTTTG
             GTGTTTAAAA AAGTAATGAG TGAAGCGTAT AACCAATCAA AAA-TGATTG
             TGAGCACCAG GGA
MAM1_SP134   TCATTGTCTG GATTGCTATT TTATTACCCA TTAAGTGGTT AACAGCTTTG
             GTGTTTAAAA AAGTAATGAG TGAAGCGTAT AACCAATCAA AAA-TGATTG
             TGAGCACCAG GGA
MAST_A026_   TCATTGTCTG GATTGCTATT TTATTACCCA TTAAGTGGTT AACAGCTTTG
             GTGTTTAAAA AAGTAATGAG TGAAGTGTAT AACCAATCAA AAAATGATTG
             TGAGCACCAG GGA
SAV1_SE210   TCATTGTCTG GATTRCTATT TTATTACCCA TTAAGTGGTT AACAGCTTTG
             GTGTTTAAAA AAGTAATGAG TGAAGCRTAT AACCAATCAA AAA-TGATTG
             TGAGCACCAG GGA

 1 1 1 1 1
5 109

ASIAN2_Ema   TCATTTCATT TATCCGTAAT CAGTGTCCGC GGAAGCTGCA GTCAGGAAAT
             CAAATCTGCT GCAAAAGACC TCTTTAAAAT GGTAAAAAGC AAAGATGTCA
             CTTTAAGGA
FOR_DS1535   TCATTTCATT TATCCGTAAT CAGTGTCCGC GGAAGCAGCA GTCAGGAAAT
             CAAATCTGCT GCAAAAGACC TCTTTAAAAT GGTAAAAWGC AAAGATGTCA
             CTTTAAGGA
MAM1_SP134   TCATTTCATT TATCCGTAAT CAGTGTCCGC GGAAGCAGCA GTCAGGAAAT
             CAAATCTGCT GCAAAAGACC TCTTTAAAAT GGTAAAAAGC AAAGATGTCA
             CTTTAAGGA
MAST_A033_   TCATTTCATT TATCCGTAAT CAGTGTCCGC GGAAGCAGCA GTCAGGAAAT
             CAAATCTGCT GCAAAAGACC TTTTTAAAAT GGTAAAAAGC AAAGATGTCA
             CTTTAAGGA
SAV1_SE210   TCATTTCATT TATCCGTAAT CAGTGTCCGC GGAAGCAGCA GTCAGGAAAT
             CAAATCTGCT GCAAAAGACC TCTTTAAAAT GGTAAAAAGC AAAGATGTCA
             CTTTAAGGA
```

```
 1 1 1 1 1
5 92

ASIAN2_Ema   CTCACCTCTC CCTCCTTAAC CACTCCTGCC CCCACAGGCC TCTCTAGCTA
             AGCCCTGGCA CTCTCCCCTG CCCAGGAAGG CCAGCCCACT CT
FOR_DS1535   CTCACCTCTC CCTCCTTAAC CGCTCCTGCC CCCACAGGCC TCTCTAGCTA
             AGCCCTGGCA CTCTCCCCTG CCCAGGAAGG CCAGCCCACT CT
MAM1_SP134   CTCACCTCTC CCTCCTTAAC CACTCCTGCC CCCACAGGCC TCTCTAGCTA
             AGCCCTGGCA CTCTCCCCTG CCCAGGAAGG CCAGCCCACT CT
MAST_A034_   CTCACCTCTC CCTCCTTAAC CGCTCCTGTC CCCACAGGCC TCTCTAGCTA
             AGCCCTGGTA CTCTCCCCTG CCCAGGAAGG CCAGCCCACT CT
SAV1_SE210   CTCACCTCTC CCTCCTTAAC CGCTCCTGCC CCCACAGGCC TCTCTAGCTA
             AGCCCTGGCA CTCTCCCCTG CCCAGGAAGG CCAGCCCACT CT

 1 1 1 1 1
5 103

ASIAN2_Ema   TGCTGTTCTG GGTTCCTAGA TTGTCATCTG TGTCTGTTTC ACATGGTTTC
             TTGGTTCTTT GTTGTAGGGG AACAGGGTGG TGTGTGTGAC TAAGCCACCA
             TTT
FOR_DS1535   TGCTGTTCTG GGTTCCTAGA TTGTCATCTG TGTCTGTTTC ACATGGTTTC
             TTGGTTCTTT GTTGTAGGGG AACAGGGTGG TGTGTGTGAC TAAGCCACCA
             TTT
MAM1_SP134   TGCTGTTCTG GGTTCCTAGA TTGTCATCTG TGTCTGTTTC ACATGGTTTC
             TTGGTTCTTT GTTGTAGGGG AACAGGGTGG TGTGTGTGAC TAAGCCACCA
             TTT
MAST_A036_   TGCTGTTCTG GGTTCCTAGA TTGTCATCTG TGTCTGTTTC ACATAGTTTC
             TTGGTTCTTT GTTGTAGGGG AACAGGGTGG TGTGTGTGAC TAAGCCACCA
             TTT
SAV1_SE210   TGCTGTTCTG GGTTCCTAGA TTGTCATCTG TGTCTGTTTC ACATGGTTTC
             TTGGTTCTTT GTTGTAGGGG AACAGGGTGG TGTGTGTGAC TAAGCCACCA
             TTT

 1 1 1 1 1
5 91

ASIAN2_Ema   TCACATGAGG ACACTCCTAT TCTCTGCCCT TCAAATTCAT TCTGACCAAG
             AGAGACGCAC ACAACGCAGC TCAGAAATGC TTTAAGATTG T
FOR_DS1535   TCACATGAGG ACACTCCTAT TCTCTGCCCT TCAAATTCAT TCTGACCAAG
             AGAGACGCAC ACAACGCAGC TCAGAAATGC TTTAAGATTG T
MAM1_SP134   TCACATGAGG ACACTCCTAT TCTCTGCCCT TCAAATTCAT TCTGACCAAG
             AGAGACACAC ACAACGCAGC TCAGAAATGC TTTAAGATTG T
MAST_A038_   TTATATGAGG ACACTCCTAT TCTCTGCTCT TCAAATTCAT TCTGACCAAG
             AGAGACGCAC ACAACGCAGC TCAGAAATGC TTTAAGATTG T
SAV1_SE210   TCACATGAGG ACACTCCTAT TCTCTGCCCT TCAAATTCAT TCTGACCAAG
             AGAGACGCAC ACAACGCAGC TCAGAAATGC TTTAAGATTG T

 1 1 1 1 1
5 98

ASIAN2_Ema   AAAAGACCAT GGGAACCACT GAACCAGTAT AACTTCTGGR ATGAAGGGAC
             ATCAATAAAT GCTCCTCACA GAGCTTCACT GAGGTCATTC TGGTCTTT
FOR_DS1535   AAAAGACCAT GGGAACCACT GAACCAGTAT AACTTCTGGA ATGAAGGGAC
             ATCAATAAAT GCTCCTCACA GAGCTTCACT GAGGTCATTC TGGTCTTT
MAM1_SP134   AAAAGACCAT GGGAACCACT GAACCAGTAT AACTTCTGGA ATGAAGGGAC
             ATCAATAAAT GCTCCTCACA GAGCTTCACT GAGGTCATTC TGGTCTTT
MAST_A042_   AAAAGATCAT GGGAACCACT GAACCAGTAT AACTTCTGGA ATGAAGGGAC
             ATCAATAAAT GCTCCTCACA GAGCTTCACT GAGGTCATTC TGGTCTTT
SAV1_SE210   AAAAGACCAT GGGAACCACT GAACCAGTAT AACTTCTGGA ATGAAGGGAC
             ATCAATAAAT GCTCCTCACA GAGCTTCACT GAGGTCATTC TGGTCTTT

 1 1 1 1 1
5 96

ASIAN2_Ema   TACCAAATCT GGCTTGGCAC CATTTGCTTA GTAGAAATCA ATTCGGAATC
             AGAAGGGCCG GTTTTGCTAA TCTTGTACTG TAAGCCGTAA GATGAG
FOR_DS1535   TACCAAATCT GGCTTGGCAC CATTTGCTTA GTAGAAATCA ATTCGAAATC
             AGAAGGGCCG GTTTTGCTAA TCTTGTACTG TAAGCCGTAA GATGAG
MAM1_SP134   TACCAAATCT GGCTTGGCAC CATTTGCTTA GTAGAAATCA ATTCGGAATC
             AGAAGGGCCG GTTTTGCTAA TCTTGTACTG TAAGCCGTAA GATGAG
```

```
MAST_A044_   TACTAAATCT GGCTTGGCAC CATTTGCTTA GTAGAAATCA ATTCAGAACC
             AGAAGGGCTG GTTTTGCTAA TCTTGTACTG TAAGCCGTAA GATGAG
SAV1_SE210   TACCAAATCT GGCTTGGCAC CATTTGCTTA GTAGAAATCA ATTCGGAATC
             AGAAGGGCCG GTTTTGCTAA TTTTGTACTG TAAGCCGTAA GATGAG

 1 1 1 1 1
5 102

ASIAN2_Ema   AAGCTACTTG GCTGATTGGC TGGAAGAGAA CCATATTTGT GCCAGTTCCA
             AAGAAAGGTG ATCCAACAGA GTGAGGAAAT TATTGAACAA TATCATTAAT
             AT
FOR_DS1535   AAGCTACTTG GCTGATTGGC TGGAAGAGAA CCATATTTGT GCCAGTTCCA
             AAGAAAGGTG ATCCAACAGA GTGAGGAAAT TATTGAACAA TATCATTAAT
             AT
MAM1_SP134   AAGCTACTTG GCTGATTGKC TGGAAGAGAA CCATATTTGT GCCAGTTCCA
             AAGAAAGGTG ATCCAACAGA GTGAGGAAAT TATTGAACAA TATCATTAAT
             AT
MAST_A046_   AAGCTACTTG GCTGATTGGC TGGAAGAGAA CCCTATTTGT GCCAGTTCCA
             AAGAAAGGTG ATCCAACAGA GTGAGGAAAT TATTGAACAA TATCATTAAT
             AT
SAV1_SE210   AAGCTACTTG GCTGATTGGC TGGAAGAGAA CCATATTTGT GCCAGTTCCA
             AAGAAAGGTG ATCCAACAGA GTGAGGAAAT TATTGAACAA TATCATTAAT
             AT

 1 1 1 1 1
5 92

ASIAN2_Ema   ACATTCATCA GCCAGTCTAG TCCCCCAGTG GACAATCCTG CTACGCGTGC
             TAGTGACTGT TCCAGCGGGG GG-TTGTAGG GGCCTACAGG GG
FOR_DS1535   ACATTCATCA GCCAGTCTAG TCCCCCAGTG GACAATCCTG CTGCAYGTGC
             TAGTGACTGT TCCAGSGGGG GG-TTGTAGG GGCCTACAGG GG
MAM1_SP134   ACATTCATCA GCCAGTCTAG TCCCCCAGTG GACAATCCTG CTACACGTGC
             TAGTGACTGT TCCAGCGGGG GG-TTGTAGG GGCCTACAGG GG
MAST_A047_   ACATTCATCA GCCAGTCTAG TCCCCCAGTG GACAATCCTG CTGCATGTGC
             TAGTGACTGT TCCGGCTGGG GG-CTGTAGG GGC-TACAGG GG
SAV1_SE210   ACATTCATCA GCCAGTCTAG TCCCCCAGTG GACAATCCTG CTGCACGTGC
             TAGTGACTGT TCCAGCGGGG GGGTTGTAGG GGCCTACAGG GG

 1 1 1 1 1
5 96

ASIAN2_Ema   CACTTAATGT CCCACAAAGG AGCTGTTGGC CTTGCCCTGG CCCTGGCTCC
             TGTGTTTGTC CAAACAGCAC AGACTCTYTT CTGTTTCTTG TCCACA
FOR_DS1535   CACTTAATGT CCCACAAAGG AGCTGTTGGC CTTGCCCTGG CCCTGGCTCC
             TGTGTTTGTC CAAACAGCAC AGACTCTCTT CTGTTTCTTG TCCACA
MAM1_SP134   CACTTAATGT CCCACAAAGG AGCTGTTGGC CTTGCCCTGG CCCTGGCTCC
             TGTGTTTGTC CAAACAGCAC AGACTCTCTT CTGTTTCTTG TCCACA
MAST_A048_   CATTTAATGT CCTACAAAGG AGCTGTTGGC TTTGCCCTGG CTCTGGCTCC
             TGTGTTTGTC CAAACAGCAC AGACTCTCTT CTGTTTCTTG TCCACA
SAV1_SE210   CACTTAATGT CCCACAAAGG AGCTGTTGGC CTTGCCCTGG CCCTGGCTCC
             TGTGTTTGTC CAAACAGCAC AGACTCTCTT CTGTTTCTTG TCCACA

 1 1 1 1 1
5 97

ASIAN2_Ema   GGAAGGGCCC CCAAAGGCTG TCATGGGGCT TGGGGCACAG GGCAGTGATT
             CACTCAGTGT CCTGTCCTGG GAAGGCTCTG TGACAGTGCT CTCAATG
FOR_DS1535   GGAAGGGCCC CCAAAGGCTG TCATGGGGCT TGGGGCACAG GGCAGTGATT
             CACTCAGTGT CCTGTCCTGG GAAGGCTCTG TGACAGTGCT CTCAACG
MAM1_SP134   GGAAGGGCCC CCAAAGGCTG TCATGGGGCT TGGGGCACAG GGCAGTGATT
             CACTCAGTGT CCTGTCCTGG GAAGGCTCTG TGACRGTGCT GTCAATG
MAST_A050_   GGAAGGGCCC ACAAAGGCTG TCATGGGGCT TGGGGCACAG GGCAGTGATT
             CACTCAGTGT CCTGTCCTGG GAAGGCTCTG TGACAGTGCT TTCAATG
SAV1_SE210   GGAAGGGCCC CCAAAGGCTG TCATGGGGCT TGGGGCACAG GGCAGTGATT
             CACTCAGTGT CCTGTCCTGG GAAGGCTCTG TGACAGTGCT CTCAATG

 1 1 1 1 1
5 91

ASIAN2_Ema   GGCTGGGCCT TTCCAGGAGG GCTCTCCAAG TATTGCCTTT CCGAGTATTC
             ACAGCCAACT TCCCCCACCA GTCATGAGTT TTTCCAAGAC T
```

FOR_DS1535   GGCTGGGCCT TTCCAGGAGG GCTCTCCAAG TATTGCCTTT CCGAGTATTC
ACAGCCAACT TCCCCCACCA GTCATGAGTT TTTCCAAGAC T
MAM1_SP134   GGCTGGGCCT TTCCAGGAGG GCTCTCCAAG TATTGCCTTT CCGAGTATTC
CCAGCCAACT TCCCCCACCA GTCATGAGTT TTTCCAAGAC T
MAST_A052_   GGCTGGGCCT TTCCAGGAGG GCTCTCCAAG TATTGCCTTT CCGAGTATTC
ACAGCCAACT TTCCCCACCA GTCATGAGTT TTTCCAAAAC T
SAV1_SE210   GGCTGGGCCT TTCCAGGAGG GCTCTCCAAG TATTGCCTTT CCGAGTATTC
ACAGCCAACT TCCCCCACCA GTCATGAGTT TTTCCAAGAC T

1 1 1 1 1
5 102

ASIAN2_Ema   TTTATTCCAT TCATATTCTC CCTGGTCTCA TCATCAAATC ATGCGTTGGG
GGATGTTGAA TATTTACAGT TGCTTTTGTA AAGAGTTTGA GTTGTTTTGG
CT
FOR_DS1535   TTTATTCCAT TCATATTCTC CCTGGTCTCA TCATCAAATC ATGCGTTGGG
GGATGTTGAA TATTTACAGT TGCTTTTGTA AAGAGTTTGA GTTGTTTTGG
CT
MAM1_SP134   TTTATTCCAT TCATATTCTC CCTGGTCTCA TCATCAAATC ATGCGTTGGG
GGATGTTGAA TATTTACAGT TGCTTTTGTA AAGAGTTTGA GTTGTTTTGG
CT
MAST_A054_   TTTATTCTCT TCATATTCTC CCTGGTCTCA TCATCAAATC ATGCATTGGG
GGATGTTGAA TATTTACAGT TGCTTTTGTA AAGAGTTTGA GTTATTTTGG
CT
SAV1_SE210   TTTATTCCAT TCATATTCTC CCTGGTCTCA TCATCAAATC ATGCGTTGGG
GGATGTTGAA TATTTACAGT TGCTTTTGTA AAGAGTTTGA GTTGTTTTGG
CT

1 1 1 1 1
5 96

ASIAN2_Ema   TAGTTTTACA AGGAAATGAG AAATTTGGTA GCTGGTGGTA TTTGAATTTT
GTTGGAAAGG AGGCTCTCAC CACAAAAGGT TGTGCCTTTG ATGTGT
FOR_DS1535   TAGTTTTACA AGGAAATGAG AAATTTGGTA GCTGGTGGTA TTTGAATTTT
GTTGGAAAGG AGGCTCTCAC CACAAAAGGT TGTGCCTTTG ATGTGT
MAM1_SP134   TAGTTTTACA AGGAAATGAG AAATTTGGTA GCTGGTGGTA TTTGAATTTT
GTTGGAAAGG AGGCTCTCAC CACAAAAGGT TGTGCCTTTG ATGTGT
MAST_A056_   TAGTTTTACA AGGAAATGAG AAATTTGGTA GCTGGTGGTA TTTGAATTT-
GTTGGAAAGG AGGCTCTCAC CACAAAAGGT TGTGCCTTTG ATGTGT
SAV1_SE210   TAGTTTTACA AGGAAATGAG AAATTTGGTA GCTGGTGGTA TTTGAATTTT
GTTGGAAAGG AGGCTCTCAC CACAAAAGGT TGTGCCTTTG ATGTGT

1 1 1 1 1
5 99

ASIAN2_Ema   AGAGTTGATT CTTTGTACCC CATCATAAGC AAACAACAGG CAGTCCATAT
GGTGCCTTCT GGATGGGTTT GGAGGGGGGA CAGCCTGGGA AGGACTGCG
FOR_DS1535   AGAGTTGATT CTTTGTAYCC CATCATAAGC AAACAACAGG CAGTCCATAT
GGTGCCTTCT GGATGGGTTT GGAGGGGGGA CAGCCTGGGA AGGACTGCG
MAM1_SP134   AGAGTTGATT CTTTGTACCC CATCATAAGC AAACAACAGG CAGTCCATAT
GGTGCCTTCT GGATGGGTTT GGAGGGGGGA CAGCCTGGGA AGGACTGCG
MAST_A057_   AGAGTTGATT CTTTGTACCC CATCATAAGC AAACAACAAG CAGTCCATAT
GGTGCCTTCT GGATGGGTTT AAAGGGGGGA CAGCCTGGGA AGGACTGCG
SAV1_SE210   AGAGTTGATT CTTTGTACCC CATCATAAGC AAACAACAGG CAGTCCATAT
GGTGCCTTCT SGATGGGTTT GGAGGGGGGA CAGCCTGGGA AGGACTGCG

1 1 1 1 1
5 127

ASIAN2_Ema   TAGTCAGGCT TTGC-CTAAA AGTTCAGT-A AA-CATTT-G ATATTGGGTT
CTTTGGCTAG GAAGGAAAGG AAATGGAAGG GAACGGAAGG CAAGGCAAGG
GAAGTGAAAC AAAGAAAATG GAAGGGA
FOR_DS1535   TAGTCAGGCT TTGC-CTAAA AGTTCAGT-A AA-CATTT-G ATATTGGGTT
CTTTGGCTAG GAAGGAAAGG AAATGGAAGG GAAGGGAAGG CAAGGCAAGG
GAAGTGAAAC AAAGAAAATG GAAGGGA
MAM1_SP134   TASTCAGGCT TTGC-CTAAA AGTTCAGT-A AA-CATTT-G ATATTGGGTT
CTTTGGCTAG GAAGGAAAGG AAATGGAAGG GAAGGGAAGG CAAGGCAAGG
GAAGTGAAAC AAAGAAAATG GAAGGGA
MAST_A069_   TAGTCAGGCT TTGCATTACA AGTTCAGTTA AAACATTTTG ATATTGGGTT
CTTTGGCTAG GAAGGAAAGG AAATGGAAGG GAAGGGAAGG GAAGGCAAGG
GAAGTGAAAC AAAGAAAATG GAAGGGA

```
SAV1_SE210   TAGTCAGGCT TTGC-CTAAA AGTTCAGT-A AA-CATTT-G ATATTGGGTT
             CTTTGGCTAG GAAGGAAAGG AAATGGAAGG GAAGGGAAGG CAAGGCAAGG
             GAAGTGAAAC AAAGAAAATG GAAGGGA

 1 1 1 1 1
5 104

ASIAN2_Ema   CCTATTAGCT AACTAAGAAA CTATTCAAAA -GGGTGGAGC ATTCCTATGA
             TGCCCCCACT CACCCATTGT AGAGCCTCTA TGGGATTTCT ACTGGCCTTC
             CTAA
FOR_DS1535   CCTATTAGCT AACTAAGAAA CTACTCAAAA -GGGTGGAGC ATTCCTATGA
             TGCCCCCACT CACCCATTGT AGAGCCTCTA TGGGATTTCT ACTGGCCTTC
             CTAA
MAM1_SP134   CCTATTAGCT AACTAAGAAA CTACTCAAAA -GGGTGGAGC ATTCCTATGA
             TGCCCCCACT CACCCATTGT AGAGCCTCTA TGGGATTTCT ACTGGCCTTC
             CTAA
MAST_A071_   TCTATTAGCT AACTAAGAAA CTACTCAAAA AGGGTGGAGC ATTCCTATGA
             TGCCCCCACT CACCCATTGT AGAGCCTCTA TGGGATTTCT ACTGGCCTTC
             CTAA
SAV1_SE210   CCTATTAGCT AACTAAGAAA CTACTCAAAA -GGGTGGAGC ATTCCTATGA
             TGCCCCCACT CACCCATTGT AGAGCCTCTA TGGGATTTCT ACTGGCCTTC
             CTAA

 1 1 1 1 1
5 110

ASIAN2_Ema   TGGGAATCAT GGTCCCAGGG TCAATTGGCA AAACAAAATT TGTAAAGAAA
             ATGTTCTACA TCCAACTTTG GTGAGTGGCA TCTGGTGTCT TAAAATCTTG
             AGAGTGGCCA
FOR_DS1535   TGGGAATCAT GGTCCCAGGG TCAATTGGCA AAACAAAATT TGTMAAGAAA
             ATGTTCTACA TCCAACTTTG GTGAGTGGCA TCTGGTGTCT TAAAATCTTG
             AGAGTGGCCA
MAM1_SP134   TGGGAATCAT GGTCCCAGGG TCAATTGGCA AAACAAAATT TGTAAAGAAA
             ATGTTCTACA TCCAACTTTG GTGAGTGGCA TCTGGTGTCT TAAAATCTTG
             AGAGTGGCCA
MAST_A075_   TGGGAATCAT GGTCCCAGGG TCAATTTGCA AAACAAAATT TGTAAAGAAA
             ATGTTCTACA TCCAACTTTG GTGAGTGGCA TCTAAGGTCT TAAATGCTAG
             CAAGGGACCA
SAV1_SE210   TGGGAATCAT GGTCCCAGGG TCAATTGGCA AAACAAAATT TGTAAAGAAA
             ATGTTCTACA TCCAACTTTG GTGAGTGGCA TCTGGTGTCT TAAAATCTTG
             AGAGTGGCCA

 1 1 1 1 1
5 127

ASIAN2_Ema   ATGGGAGACT TGCCACATGC ACCCAAATAA CTGCTTCTAA CGCACCATCC
             CTGTGATTCT CTGGAAAACT GGAAGTCAGA GGAAAGAGAA ATGTTGTTTT
             TTGTTTTTTT TTTTCTA-CT GTTGTCT
FOR_DS1535   ATGGGAGACT TGCCACATGC ACCCAAATAA CTGCTTCTAA CGCACCATCC
             CTGTGATTCT CTGGAAAACT GGAAGTCAGA GGAAAGAGAA ATGTTKTTKT
             TTTTTTTTTT TTTTTTG-CT GTTGTCT
MAM1_SP134   ATGGGAGACT TGCCACATGC ACCCAAATAA CTGCTTCTAA CGCACCATCC
             CTGTGATTCT CTGGAAAACT GGAAGTCAGA GGAAAAAGAA ATGTTGTTTT
             TTTTTTT--- ------GTCT GTTGTCT
MAST_A076_   ATGGGAGACT TGCCACATGC ACCCAAATAA CTGCTTTTAA CGCACCATCC
             CTGTGATTCT CTGGAAAACT GGAAGTCAGA GGAAAGAGAA ATGTTTATTT
             TTTTTTT--- ---------T TTTGTCT
SAV1_SE210   ATGGGAGACT TGCCACATGC ACCCAAATAA CTGCTTCTAA CGCACCATCC
             CTGTGATTCT CTGGAAAACT GGAAGTCAGA GGAAAGAGAA ATGTTGTTTT
             TTTTTT---- ------G-CT GTTGTCT

 1 1 1 1 1
5 107

ASIAN2_Ema   AGGGACCTTG TAATTTTTGA GAGGGTGGAA TGCTTGACTT CCAGAATCTG
             TTTGGAAATC TTTCCCGCTA AAGCCAAATT TCTATATTTC ATAATCTAAA
             GACGCTT
FOR_DS1535   AGGGACCTTG TAATTTTTGA GAGGGTGGAA TGCTTGACTT CCAGAATCTG
             TTTGGAAATC TTTCCAGCTA AAGCCAAATT TCTATATTTC ATAATCTAAA
             GATGCTT
MAM1_SP134   AGGGACCTTG TAATTTTTGA GAGGGTGGAA TGCTTGACTT CCAGAATCTG
```

```
             TTTGGAAATC TTTCCCGCTA AAGCCAAATT TCTATATTTC ATAATCTAAA
             GACGCTT
MAST_A077_   AGGGACCTTG TAATTTTTGA GAGGGTGGGA TGCTTGACTT CCAGAATCTG
             TTTGGAAATC TTTCCAGCTA AAGCCAAATT TCTATATTTC ATAATCTAAA
             GACGCTT
SAV1_SE210   AGGGACCTTG TAATTTTTGA GAGGGTGGAA TGCTTGACTT CCAGAATCTG
             TTTGGAAATC TTTCCAGCTA AAGCCAAATT TCTATATTTC ATAATCTAAA
             GACGCTT

 1 1 1 1 1
5 95

ASIAN2_Ema   CATTTT-GTT GTTGTTTTT- GCTTT-CTTG TTGCAAGTCC ACTGTAGTAC
             AGCTGAGATG TCTCGCCTCT GCSTGAAGGT ATTGTATGTG TTGGC
FOR_DS1535   CATTTT-GTT GTTGTTTTT- GCTTT-CTTG TTGCAAGTCC ACTGTAGTAC
             AGCTGAGATG TCTCGCCTCT GCCTGAAGGT ATTGTATGTG TTGGC
MAM1_SP134   CATTTT-GTT GTTGTTTTT- GCTTT-CTTG TTGCAAGTCC ACTGTAGTAC
             AGCTGAGATG TCTCGCCTCT GCCTGAAGGT ATTGTATGTG TTGGC
MAST_A078_   TATTTTTGTT GTTGTTTTTT GCTTTTCTTG TTGCAAGTCC ACTGTAGTAC
             AGCTGAGATG TCTCGCCTCT ACCTGAAGGT ATTGTATGTG TTAGC
SAV1_SE210   CATTTT-GTT GTTGTTTTT- GCTTT-CTTG TTGCAAGTCC ACTGTAGTAC
             AGCTGAGATG TCTCGCCTCT GCCTGAAGGT ATTGTATGTG TTGGC

 1 1 1 1 1
5 106

ASIAN2_Ema   GAAAGAGCCC CTTTGTTCCA GAGCTCCTGG GTGACAATGG CAGGAGAGGG
             GAGTGTGGCT TCCATTGGGC CAAAAGGTGG CCTGGCCTTG AAGCCCCGAT
             GGCTAA
FOR_DS1535   GAAAGAGCCC CTTTGTTCCA GAGCTCCTGG GCGACAATGG CAGGAGAGGG
             GAGTGTGGCT TCCATTGGGC CAAAAGGTGG CCTGGCCTTG AAGCCCCGAT
             GGCTAA
MAM1_SP134   GAAAGAGCCC CTTTGTTCCA GAGCTCCTGG GCGACAATGG CAGGAGAGGG
             GAGTGTGGCT TCCATTGGGC CAAAAGGTGG CCTGGCCTTG AAGCCCCGAT
             GGCTAA
MAST_A082_   GAAAGAGCCC CTTTGTTCCA GAGCTCCTGG GCGACAATGG CAGGAGAGGG
             GAGTGTGGCT TCCATTGGGC CAAAAGGTGG CCTGGCCTTG AAGCCCCGAT
             GGCTAA
SAV1_SE210   GAAAGAGCCC CTTTGTTCCA GAGCTCCTGG GCGACAATGG CAGGAGAGGG
             GAGTGTGGCT TCCATTGGGC CAAAAGGTGG CCTGGCCTTG AAGCCCCGAT
             GGCTAA

 1 1 1 1 1
5 103

ASIAN2_Ema   TGTGACTCTT GTGTGCTTAA CGCTCACAGC CTGCTAGGTT AGTCGAGCCT
             CCCCACTCGG GGGAGCAAAC CACCTCGGCC ACTGGACACG GGATCAAGGG
             TCA
FOR_DS1535   TGTGACTCTT GTGTGCTTAA CGCTCACAGC CTGCTAGGTT AGTCGAGCCT
             GCCCACTCGG GGGAGCAAAC CACCTCGGCC ACTGGACACG GGATCAAGGG
             TCA
MAM1_SP134   TGTGACTCTT GTGTGCTTAA CGCTCACAGC CTGCTAGGTT AGTCGAGCCT
             CCCCACTCGG GGGAGCAAAC CAYCTCGGCC ACTGGACACG GGATCAAGGG
             TCA
MAST_A083_   TGTGATTCTT GTGTGCTTAA CGCTCACAGC CTGCTAGGTA AGTCGAGCCT
             CCCCACTCGG GGGAGCAAAC CACCTCGGCC ACTGGACACG GGATCAAGGG
             TCA
SAV1_SE210   TGTGACTCTT GTGTGCTTAA CGCTCACAGC CTGCTAGGTT AGTCGAGCCT
             GCCCACTCGG GGGAGCAAAC CACCTCGGCC ACTGGACACG GGATCAAGGG
             TCA

 1 1 1 1 1
5 101

ASIAN2_Ema   GTCTGTCAGG CTTTCTTAGG AAGGAGAAGA GCAGTCTTGT ACCCCACTGC
             ATCCCCTTAC CTGACCCCCT TCCCAGCCTT TACTGCGCTG TTGGCTAAGT
             G
FOR_DS1535   GTCTGTCAGG TTTTCTTAGG AAGGAGAAGA GCAGTCTTGT ATCCCACTGC
             ATCCCCTTAC CTGACCCCCT TCCCAGGCTT TACTGCGCTG TGGGCTAAGT
             G
MAM1_SP134   GTCTGTCAGG CTTTCTTAGG AAGGAGAAGA GCAGYCTTGT ACCCCACTGC
```

```
            ATCCCCTTAC CTGACCCCCT TCCCAGCCTT TACTGCGCTG TTGGCTAAGT
            G
MAST_A084_  GTCTGTCAGG CTTCCTTAGG AAGGAGAAGA GCAGTCTTGT ACCCCACTGC
            ATCCCCTTAC CTGACCCCCT TCCCAGCCTT TACTGTGCTG TGGGCTAAGT
            G
SAV1_SE210  GTCTGTCAGG CTTTCTTAGG AAGGAGAAGA GCAGTCTTGT ACCCCACTGC
            ATCCCCTTAC CTGACCCCCT TCCCAGGCTT TACTGCGCTG TGGGCTAAGT
            G

 1 1 1 1 1
5 93

ASIAN2_Ema  TCCTGCCTCT CTAGCCCAGT GGCCTTCCAC CATCTGTCCA TGGCCCTGCA
            CCTCCTCTCC TGCCCTGCCA CCCTGCAGCC ACCAGGAATT GTT
FOR_DS1535  TCCTGCCTCT CTAGCCCAGT GGCCTTCCAC CATCTGTCCA TGTCCCTGCA
            CCTCCTCTCC TGCCCTGCCA CCCTGCAGCC ACCAGGAATT GTT
MAM1_SP134  TCCTGCCTCT CTAGCCCAGT GGCCTTCCAC CATCTGTCCA TGGCCCTGCA
            CCTCCTCTCC TGCCCTGCCA CCCTGCAGCC ACCAGGAATT GTT
MAST_A085_  TTTTGCCTCT CTAGCCCAGT GGCTTTCCAC CATCTGTCTA TGGCCCTACC
            CCTCCTCTCC TGCCCTGCCA CCCTGCAGCC ACCAGGAATT GTT
SAV1_SE210  TCCTGCCTCT CTAGCCCAGT GGCCTTCCAC CATCTGTCCA TGGCCCTGCA
            CCTCCTCTCC TGCCCTGCTA CCCTGCAGCC ACCAGGAATT GTT

 1 1 1 1 1
5 94

ASIAN2_Ema  GAGACGTTGC TCTAGGAGGG TCTTGTCTGC CTTCCTGCCC CAAACACACA
            GTAAGGATTG GCCCCCCAGC ACCTCCTAAA GGAGATGGAC CAGT
FOR_DS1535  GAGACGTTGC TCTAGGAGGG TCTTGTCTGC CTTCCTGCCC CAAACACACA
            GTAAGGATTG SCCCCCCAGC ACCTCCTAAA GGAGATGGAC CAGT
MAM1_SP134  GAGACATTGC TCTAGGAGGG TCTTGTCTAC CTTCCTGCCC CAAACACACA
            ATAAGGATTG GCCCCCCAGC ACCTCCTAAA GGAGATGGAC CAGT
MAST_A086_  GAGATGTTGC TCTAGGAGGG TCTTGTCTGC CTTCCTGCCC CAAACACACA
            GTAAGGATTG GCCCCCCAGC ACCTTCTAAA GGAGATGGAC CAGT
SAV1_SE210  GAGACGTTGC TCTAGGAGGG TCTTGTCTGC CTTCCTGCCC CAAACACACA
            GTAAGGATTG GCCCCCCAGC ACCTCCTAAA GGAGATGGAC CAGT

 1 1 1 1 1
5 98

ASIAN2_Ema  AAAACTCAGG GCCCCTTGTT TAAAATCATG AATTTCAAGA AGGAGATGTA
            TTAAACCAAG CGTGGGGTCC CTCTAAGCGT GAAACCCCGT CCAACTGC
FOR_DS1535  AAAACTCAGG GCCCCTTGTT TAAAATCATG AATTTCAAGA AGGAGATGTA
            TTAAACCAAG CGTGGGGTCC CTCTAAGCGT GAAACCCCGT CCAACTGC
MAM1_SP134  AAAACTCAGG GCCCCTTGTT TAAAATCATG AATTTCAAGA AGGAGATGTA
            TTAAACCAAG CGTGGGGTCC CTCTAAGCGT GAAACCCCGT CCAACTGC
MAST_A087_  AAAACTCAGG GCCCCTTGTT TAAAATCACG AATTTCAAGA AGGAGATGTA
            TGAAACCAAG CGTGGGATCC CCCTAAGCGT GAAACCCTGT CCAACTGC
SAV1_SE210  AAAACTCAGG GCCCCTTGTT TAAAATCATG AATTTCAAGA AGGAGATGTA
            TTAAACCAAG CGTGGGGTCC CTCTAAGCGT GAAACCCCGT CCAACTGC

 1 1 1 1 1
5 103

ASIAN2_Ema  AGAAAATCTT AAATCTTTGT GCTTGAAGGG GCTTTGAGAG ATACCTATGT
            CCGTTCCTGG TCTTAGTGTG AGTAAGAGCT GATGGGACAA CATGGCCAGA
            GCG
FOR_DS1535  AGAAAATCTT AAATCTTTGT GCTTGAAGGG GCTTTGAGAG ATACCTATGT
            CCGTTCCTGG TCTTAGTGTG AGTAAGAGCT GATGGGACAA CATGGCCAGA
            GCG
MAM1_SP134  AGAAAATCTT AAATCTTTGT GCTTGAAGGG GCTTTGAGAG ATACCTATGT
            CCGTTCCTGG TCTTAGTGTG AGTAAGAGCT GATGGGACAA CATGGCCAGA
            GCG
MAST_A088_  AGAAAATCTT AAATCTTTGT GCTTGAAGGG GCTTTGAGAG ATACCTATGT
            CCGTTCCTGG TCTTAGTGTG AGTAAGAGCT GATGGGACAA CATGGCCAGA
            GCG
SAV1_SE210  AGAAAATCTT AAATCTTTGT GCTTGAAGGG GCTTTGAGAG ATACCTATGT
            CCGTTCCTGG TCTTAGTGTG AGTAAGAGCT GATGGGACAA CATGGCCAGA
            GCG

 1 1 1 1 1
```

5 94

```
ASIAN2_Ema   TGACATAGTT TCCAGCATCA TAGTAATACA CAAGGCA-CC AGAGAATGAC
             AGACAGGTGG TGGCATTTGC AGTAAGATGT GTCCATTTC- ACAA
FOR_DS1535   TGACATAGTT TCCAGCATCA TAGTAATACA CAAGGCA-CC AAAGAATGAC
             AGACAGGTGG TGACATTTGC AGTAAGATGT GTCCATTTC- ACAA
MAM1_SP134   TGACATAGTT TCCAGCATCA TAGTAATACA CAAGGCA-CC ACAGAATGAC
             AGACAGGTGG TGGCATTTGC AGTAAGATGT GTCCATTTC- ACAA
MAST_A091_   TGACATAGTT TCCAGCATCA TAGTAATACA CAAGGCACCC ACAGAATGAC
             AGACAGGTGG TGGCATTTGC AGTAAGGTGT GTCCATTTCA ACAA
SAV1_SE210   TGACATAGTT TCCAGCATCA TAGTAATACA CAAGGCA-CC ACAGAATGAC
             AGACAGGTGG TGGCATTTGC AGTAAGATGT GTCCACTTC- ACAA
```

 1 1 1 1 1
5 98

```
ASIAN2_Ema   ATTTAAGCTT TGGAATTCTG CTAATCCAAG GCGACAGGAG ACCTGCCCTT
             CAGTCCAACA TGGTGTGTGT GTGTTTGAGT ATATGTGTGG ATATCAAC
FOR_DS1535   ATTTAAGCTT TGGAATTCTG CTAATCCAAG GCGACAKGAG ACCTGCCCTT
             CAGTCCAACA TGGTGTGTGT GTGTTTGAGT ATATGTGTGG RTATCAAC
MAM1_SP134   ATTTAAGCTT TGGAATTCTG CTAATCCAAG GCGACAGGAG ACCTGCCCTT
             CAGTCCAACA TGGTGTGTGT GTGTTTGAGT ATATGTGTGG ATATCAAC
MAST_A094_   ATTTAAGCTT TGGAATTCTG CTAATCCAAG GCGACAGGAG ACCTGCCCTT
             CAGTCCAACA TGGTGTGTGT GTGTTTGAGT ATATGTGTGG GTATCAAC
SAV1_SE210   ATTTAAGCTT TGGAATTCTG CTAATCCAAG GCGACAGGAG ACCTGCCCTT
             CAGTCCAACA TGGTGTGTGT GTGTTTGAGT ATATGTGTGG ATATCAAC
```

 1 1 1 1 1
5 100

```
ASIAN2_Ema   ACAACAACAT GGACAAAATA CTGAACAACA CGGGAAGCAT CAAAAGAAGA
             TGGAAAGAAT ACACAGAACC ATTGTACCAA AAAGAACTGG TCAATGTACA
FOR_DS1535   ACAACAACAT GGACAAAATM CTGAACAACA CGGGAAGCAT CAAAAGAAGA
             TGGAAAGAAT ACACAGAATC ATTGTACCAA AAAGAACTGG TCAATGTACA
MAM1_SP134   ACAACAACAT GGACAAAATA CTGAACAACA CGGGAAGCAT CAAAAGAAGA
             TGGAAAGAAT ACACAGAATC ATTGTACCAA AAAGAACTGG TCAATGTACA
MAST_A097_   ATAATAATAT GGACAAAATA CTGAACAACA CGGGAAGCAT CAAAAGAAGA
             TGGAAAGAAT ACACAGAATC ATTGTACCAA AAAGAATTGG TCAATGTACA
SAV1_SE210   ACAACAACAT GGACAAAATA CTGAACAACA CGGGAAGCAT CAAAAGAAGA
             TGGAAAGAAT ACACAGAATC ATTGTACCAA AAAGAACTGG TCAATGTACA
```

 1 1 1 1 1
5 101

```
ASIAN2_Ema   TGAGCATCTT CCTTAGGCCA CATTTCATGA GAAGGCTGTG GTCTGGAGCC
             CTGGGTTGGG GGAAGTCAGT TGTTTCATCT TCCAATTACC CACAGTGAGC
             T
FOR_DS1535   TGAGCATCTT CCTTAGGCCA CATTTCATGA GAAGGCTGTG GTCTGGAGCC
             CTGGGTTGGG GGAAGTCAGT TGTTTCATCT TCCAATTACC CACAGTGAGC
             T
MAM1_SP134   TGAGCATCTT CCTTAGGCCA CATTTCATGA GAAGGCTGTG GTCTGGAGCC
             CTAGGTTGGG GGAAGTCAGT YGTTTCATCT TCCAATTACC CACAGTGAGC
             T
MAST_A099_   TGAGCATCTT CCTTAGGCCA CATTTCGTGA GAAGGCTGTG GTCTGGAGCC
             CTGGGTTGGG GGAAGTCAGT CATTTCATCT TCCAATTACC CACAATGAAC
             T
SAV1_SE210   TGAGCATCTT CCTYAGGCCA CATTTCATGA GAAGGCTGTG GTCTGGAGCC
             CTGGGTTGGG GGAAGTCAGT TGTTTCATCT TCCAATTACC CACAGTGAGC
             T
```

 1 1 1 1 1
5 96

```
ASIAN2_Ema   AGAGCAAATG GGCCAGAGGA CGCCCCATCA TCCACTGCCA TCCAGTCGAT
             CCTGCCAGAG TCGCTGAGAA GACAAACCTC CATCCTATTC CGTTTG
FOR_DS1535   AGAGCAAATG GGCCAGAGGA CGCCCCATCA TCCACTGCCA TCCAGTCGAT
             CCTGCCAGAG TCGCTGAGAA GACAAACTTC CATCCTATTC CGTTTG
MAM1_SP134   AGAGCAAATG GGCCAGAGGA CGCCCCATCA TCCACTGCCA TCCAGTCGAT
             CCTGCCAGAG TCGCTGAGAA GACAAACTTC CATCCTATTC CGTTTG
MAST_A101_   AGAGCAAATG GGCCAGAGAA TGCCCCATTA TCCATTGCCA TCCAGTCGAT
             CCTGCCAGAG TCGCTGAGAA GACAAACTTC CATCCTATTC CGTTTG
```

```
SAV1_SE210    AGAGCAAATG GGCCAGAGGA CGCCCCATCA TCCACTGCCA TCCAGTCGAT
              CCTGCCAGAG TCGCTGAGAA GACAAACATC CATCCTATTC CGTTTG

 1 1 1 1 1
5 96

ASIAN2_Ema    ACATTCTAGA TCCTCCTGCC CAGCATTCTG GCCTCTGCTA GACACACCCC
              CAGCAGCATT CCTGGCATTC TAGGCTCCAA ACAGGTTCTA GAGCTT
FOR_DS1535    ACATTCTAGA TCCTCCTGCC CAGCATTCTG GCCTCTGCTA GACACACCCC
              CAGCAGCATT CCTGGCATTC TAGGCTCCAA ACAGGTTCTA GAGCTT
MAM1_SP134    ACATTCTAGA TCCTCCTGCC CAGCATTCTG GCCTCTGCTA GACACACCCC
              CAGCAGCATT CCTGGCATTC TAGGCTCCAA ACAGGTTCTA GAGCTT
MAST_A105_    ACATTCTAGA CCCTCCTGCC CCGCATTCTG GCCTCTGCTA GACACACCCC
              CAGCAGCATT CCTGGCATTC TAGGCTCCAA ACAGGTTCTA GAGCTT
SAV1_SE210    ACATTCTAGA TCCTCCTGCC CAGCATTCTG GCCTCTGCTA GACACACCCC
              CAGCAGCATT CCTGGCATTC TAGCCTCCAA ACAGGTTCTA GAGCTT

 1 1 1 1 1
5 106

ASIAN2_Ema    CCTATCATTC ACCTTCACTA AGAATATTGC TTGACTTGCA GAGGTCATGA
              AGAACTGGAA TCTTGCAGGC TTTAACGAGG AAAAAGAAAA TTTAAAAGTC
              TCTATA
FOR_DS1535    CCTATCATTC ACCTTCACTA AGAATATTGC TTGACTTGCA GAGGTCATGA
              AGAACTGGAA TCTTGCAGGC TTTAACGAGG AAAAAGAAAA TTTAAAAGTC
              TCTATA
MAM1_SP134    CCTATCATTC ACCTTCACTA AGAATATTGC TTGACTTGCA GAGGTCATGA
              AGAACTGGAA TCTTGCAGGC TTTAACGAGG AAAAAGAAAA TTTAAAAGTC
              TCTATA
MAST_A107_    CCTATCATTC ACCTTCACTA AGAATATTGC TTGACTTGCA GAGGTCATGA
              AGAACTGGAA TCTTGCAGGC TTTAACGAAG AAAAAGAAAA TTTAAAAGTC
              TCTGTA
SAV1_SE210    CCTATCATTC ACCTTCACTA AGAATATTGC TTGACTTGCA GAGGTCATGA
              AGAACTGGAA TCTTGCAGGC TTTAACGAGG AAAAAGAAAA TTTAAAAGTC
              TCTATA

 1 1 1 1 1
5 94

ASIAN2_Ema    TAGTGGAAGC CCCTGGTGGC GGCGAGGCTG CGAAGCACCG GGCCCTGGAT
              GGCGGAGGCT CCTCCTCCTT CGTCTGCCGC CGAGGAGACG CCGA
FOR_DS1535    TAGTGGAAGC CCCTGGTGGC GGCGAGGCTG CGAAGCACCG GGCCCTGGAT
              GGCGGAGGCT CCTCCTCCTT CGTCTGCCGC CGAGGAGACG CCGA
MAM1_SP134    TAGTGGAAGC CCCTGGTGGC GGCGAGGCTG CGAAGCACCG GGCCCTGGAT
              GGCGGAGGCT CCTCCTCCTT CGTCTGCCGC CGAGGAGACG CCGA
MAST_A109_    TGGTGGAAGC CTTTGGTGGC GGCGAGGTTG CGAAGCACCG GGCCCTGGAT
              GGCGGAGGCT CCTCCTCCTT CGTCTGCTGC CGAGGAGAGG CCGC
SAV1_SE210    TAGTGGAAGC CCCTGGTGGC GGCGAGGCTG CGAAGCACCG GGCCCTGGAT
              GGCGGAGGCT CCTCCTCCTT CGTCTGCCGC CGAGGAGACG CCGA

 1 1 1 1 1
5 102

ASIAN2_Ema    CTGGTGGATT TGAACTGCTG ACCTTTT-GA TTAGCAGCCA AGCTCTTAAC
              CACTGTGCCA CCAACTTCCA GATGCCTGCT ACCCAGGCAT AAAA-GATTT
              TA
FOR_DS1535    CTGGTGGATT TGAACTGTTG ACCTTTT-GA TTAGCAGCCA AGCTCTTAAC
              CACTGTGCCA CCAACTTCCA GATGCCTGCT ACCCAGGCAT AAAA-GATTT
              TA
MAM1_SP134    CTGGTGGATT TGAACTGCTG ACCTTTT-GA TTAGCAGCCA AGCTCTTAAC
              CACTGTGCCA CCAACTTCCA GATGCCTGCT ACCCAGGCAT AAAA-GATTT
              TA
MAST_A112_    CTGGTGGATT TGAACTGCTG ACCTTTTTGA TTAGCAGCCA AGCTCTTAAC
              CACTGTGCCA CCAACTTCCA GATGCCTGCT ACCCAGGCAT AAAAAGATTT
              TA
SAV1_SE210    CTGGTGGATT TGAACTGCTG ACCTTTT-GA TTAGCAGCCA AGCTCTTAAC
              CACTGTGCCA CCAACTTCCA GATGCCTGCT ACCCAGGCAT AAAA-GATTT
              TA

 1 1 1 1 1
5 102
```

```
ASIAN2_Ema     TCCTGATGGG GTGGGGCCGG CAGAGCAGAG GGGATTTTTT AGCCTGAATT
               ATCAGCGTTG CTCATCTCGA GTGGCCAGTG AACAAGAGAA GTCAAATCTC
               CT
FOR_DS1535     TCCTGATGGG GTGGGGCCGG CAGAGCAGAG GGGATTTTTT AGCCTGAATT
               ATCAGCGTTG CTCATCTCGA GTGGTCAGTG AACAAGAGAA GTCAAATCTC
               CT
MAM1_SP134     TCCTGATGGG GTGGGGCCGG CAGAGCAGAG GGGATTTTTT AGCCTGAATT
               ATCAGCGTTG CTCATCTCGA GTGGCCAGTG AACAAGAGAA GTCAAATCTC
               CT
MAST_A113_     TTCTGATGGG GTGGGGCCGG CAGAGCAGAG GGGATTTTTT AGCCTGAATT
               ATCAGCGTTG CTCATTTCGA GTGGCCAGTG AACAAGAGAA GTCAAATCTC
               CT
SAV1_SE210     TCCTGATGGG GTGGGGCCGG CAGAGCAGAG GGGATTTTTT AGCCTGAATT
               ATCAGCGTTG CTCATCTCGA GTGGTCAGTG AACAAGAGAA GTCAAATCTC
               CT

 1 1 1 1 1
5 98

ASIAN2_Ema     TTCTATCTTC CCAGCCACTC TGAGGAACTG GTATGGGTCA GTAGGTGGGA
               AAATGAGTCT CTGAGAAGCT GGGTGTGTGG CTGGGCCCAA GAGGTAAT
FOR_DS1535     TTCTATCTTC CCAGCCACTC TGAGGAACTG GTATGGGTCA GTAGGTGGGA
               AAATGAGTCT CTGAGAAGCT GGGTGTGTGG CTGGGCCCAA GAGGTAAT
MAM1_SP134     TTCTATCTTC CCAGCCACTC TGAGGAACTG GTATGGGTCA GTAGGTGGGA
               AAATGAGTCT CTGAGAAGCT GGGTGTGTGG CTGGGCCCAA GAGGTAAT
MAST_A117_     TTCTATCTTC CCAGCCACTC TGAGGAACTG GTATGGGTCA GTAGGTGGGA
               AAATGAGTCT CTGAGAAGCT GGGTGTGTGG CTGGGCCCAA GAAGTAAT
SAV1_SE210     TTCTATCTTC CCAGCCACTC TGAGGAACTG GTATGGGTCA GTAGGTGGGA
               AAATGAGTCT CTGAGAAGCT GGGTGTGTGG CTGGGCCCAA GAGGTAAT

 1 1 1 1 1
5 105

ASIAN2_Ema     AACTGTGCTC CATAAGGCTT TCAATGGCTG TGATCTTCAG GAGGTAAGTC
               AGCGGGCCTT TCTTCTGAGG TGCCTCTGGG TGGACTCGAA CTGCCAACCT
               TTCAG
FOR_DS1535     AACTGTGCTC CATAAGGCTT TCAATGGCTG TGATCTTCAG GAAGT----C
               AGCGGGCCTT TCTTCTGAGG TGCCTCTGGG TGGAYTCGAA CTGCCAACCT
               TTCAG
MAM1_SP134     AACTGTGCTC CATAAGGCTT TCAATGGCTG TGATCTTCAG GAAGTAAGTC
               AGCGGGCCTT TCTTCTGAGG TGCCTCTGGG TGGACTCGAA CTGCCAACCT
               TTCAG
MAST_A122_     AACTGTGCTC CATAAGGCTT TCAATGGCTG TGATCTTCAG GAAGTAAGTC
               ACGGGGCCTT TCTTCTGAGG TGCCTCTGGG TGGACTCGAA CTGCCAACCT
               TTCAG
SAV1_SE210     AACTGTGCTC CATAAGGCTT TCAATGGCTG TGATCTTCAG GAAGTAAGTC
               AGCGGGCCTT TCTTCTGAGG TGCCTCTGGG TGGACTCGAA CTGCCAACCT
               TTCAG

 1 1 1 1 1
5 102

ASIAN2_Ema     GGTAAATAAG AACTTTAACT CTTGAAGCCG GAAGGATCTG GGGAATCGAA
               TGCAGCAAAG GCCTAATACA TAATTTAGTC ATGAAAGGCC AAAA-TAGAT
               CA
FOR_DS1535     GGTAAATAAG AACTTTAACT CTTGAAGCCA GAAGGATCTG GGGAATCGAA
               TGCAGCAAAG GCCTAATACA TAATTTAGTC ATGAAAGGCC AAAA-TAGAT
               CA
MAM1_SP134     GGTAAATAAG AACTTTAACT CTTGAAGCCA GAAGGATCTG GGGAATCGAA
               TGCAGCAAAG GCCTAATACA TAATTTAGTC ATGAAAGGCC AAAA-TAGAT
               CA
MAST_A123_     GGTAAATAAG AACTTTAACT CTTGAAGCCA GAAGGATCTG GGGAATCGAA
               TGCAGCAAAG GCCTAATACA TAATTTAGTC ATGAAAGGCC AAAAATAGAT
               CA
SAV1_SE210     GGTAAATAAG AACTTTAACT CTTGAAGCCA GAAGGATCTG GGGAATCGAA
               TGCAGCAAAG GCCTAATACA TAATTTAGTC ATGAAAGGCC AAAA-TAGAT
               CA

 1 1 1 1 1
5 93
```

```
ASIAN2_Ema    AAAGGCCTGG CAATTT--GC TTCTGAAAAA TTATCCATCG AAAACCCAAT
              GGGCACAGCT TTACTCTGAC ACACATGGGG TCGCCATGAG TCG
FOR_DS1535    AAAGGCCTGG CAATTT--AC TTCTGAAAAA TTATCCATCG AAAACCCAAT
              GAGCACAGCT TTACTCTGAC ACACATGGGG TCGCCATGAG TCG
MAM1_SP134    AAAGGCCTGG CAATTT--AC TTCTGAAAAA TTATCCATCG AAAACCCAAT
              GAGCACAGCT TTACTCTGAC ACACATGGGG TCGCCATGAG TCG
MAST_A128_    AAAGGCCTGG CGATTTGTA- TTC--AAAAA TTATCCATCG AAAACCCAAA
              GAGCACAGCT TTACTCTGAC ACACATGGGG TCGCCATGAG TCG
SAV1_SE210    AAAGGCCTGG CAATTT--AC TTCTGAAAAA TTATCCATCG AAAACCCAAT
              GAGCACAGCT TTACTCTGAC ACACATGGGG TCGCCATGAG TCG

 1 1 1 1 1
5 101

ASIAN2_Ema    TCCTGGAAGC ACACAGACCT GTGCCAACAG AACCTCTCCT GGCATGCTGG
              AATTGGCCCT GTTGTCATCC CCACATTTTT CT-CTGGAAC AAGGAGATAA
              A
FOR_DS1535    TCCTGGAAGC ACACAGACCT GTGCCAACAG AACCTCTCCT GGCATGCTGG
              AATTGGCCCT GTTGTCATCC CCACATTTTT CT-CTGGAAC AAGGAGATAA
              A
MAM1_SP134    TCCTGGAAGC ACACAGACCT GTGCCAACAG AACCTCTCCT GGCATGCTGG
              AATTGGCCCT GTTGTCATCC CCACATTTTT CT-CTGGAAC AAGGAGATAA
              A
MAST_A140_    TCCTGGAAGC ACATAGACCT GTGCCAACAG AACCTCTCCT GGCATGCTGG
              AATTGGCCCT GTTGTCATCC CTACATTTTT CTTCTGGAAC AAGGAGATAA
              A
SAV1_SE210    TCCTGGAAGC ACACAGACCT GTGCCAACAG AACCTCTCCT GGCATGCTGG
              AATTGGCCCT GTTGTCATCC CCACATTTTT CT-CTGGAAC AAGGAGATAA
              A

 1 1 1 1 1
5 101

ASIAN2_Ema    ACCCCTGAGA TTCAGGCCAA GCCCTACTTC TCTGCAGAGG TGATGGAGCC
              TGGGAGCTGG GCCAAGTAAA GCCTTTT-CC AGCCTTTCAA AGACTTTTCA
              T
FOR_DS1535    ACCCCTGAGA TTCAGGCCAA GCCCTACTTC TCTGCAGAGG TGATGGAGCC
              TGGGAGCTGG GCCAAGTAAA GCCTTTT-CC AGCCTTTCAA AGACTKTTCA
              T
MAM1_SP134    ACCCCTGCTA TTCAGGCCAA GCCCTACTTC TCTGCAGAGG TGATGGAGCC
              TGGGAGCTGG GCCAAGTAAA GCCTTTT-CC AGCCTTTCAA AGACTTTTCA
              T
MAST_A141_    ACTCTTGAGA TTCAGGCCAA GCCCTACTTC TCTGCAGAGG TGATGGAGCC
              TGGGAGCTGG GCCAAGTAAA GCCTTTTTCC AGCCTTTCAA AGACTTTTCA
              T
SAV1_SE210    ACCCCTGAGA TTCAGGCCAA GCCCTACTTC TCTGCAGAGG TGATGGAGCC
              TGGGAGCTGG GCCAAGTAAA GCCTTTT-CC AGCCTTTCAA AGACTGTTCA
              T

 1 1 1 1 1
5 102

ASIAN2_Ema    TTTAGTGATG ATAGTCCAGG TGGAGGCACA CACCAAGGGC AAAGAAACAT
              GGAGAGCAGC GTTCTCAACC CCGACGCATG TTAGGATCAC CTGGAAAATG
              GC
FOR_DS1535    TTTAGTGATG ATAGTCCAGG TGGAGGCACA CACCAAGGGC AAAGAAACAT
              GGAGAGCAGC RTTCTCAACC CCGACGCATG TTAGGATCAC CTGGAAAATG
              GC
MAM1_SP134    TTTAGTGATG ATAGTCCAGG TGGAGGCACA CACCAAGGGC AAAGAAACAT
              GGAGAGCAGC GTTCTCAACC CCGACGCATG TTAGGATCAC CTGGAAAATG
              GC
MAST_A144_    TTTAGTGATG ATAGTCCAGG TGGAGGCACA CACCAAGGGC AAAGAAACAT
              GGAGAGCAGC GTTCTCAACC CCGACGCATG TTAGGATCAC CTGGAAAATA
              AC
SAV1_SE210    TTTAGTGATG ATAGTCCAGG TGGAGGCACA CACCAAGGGC AAAGAAACAT
              GGAGAGCAGC GTTCTCAACC CCGACGCATG TTAGGATCAC CTGGAAAATG
              GC

 1 1 1 1 1
5 101
```

ASIAN2_Ema TACGTTGAAC ACAGAGCAAC TCCTTCTGTC TGCCTCAGAC TCCATCCTCA
GGCCCTCTCT TCCCCTCTAG GAATTTCACA GGTTACAGCT TCTTCATGGG
G
FOR_DS1535 TACGTTGAAC ACAGAGCAAC TCCTTCTGTC TGCCTCAGAC TCCATCCTCA
GGCCCTCTCT TCCCCTCTAG GAATTTCWCA GGTTACAGCT TCTTCATGGG
G
MAM1_SP134 TACGTTGAAC ACAGAGCAAC TCCTTCTGTC TGCCTCAGAC TCCATCCTCA
GGCCCTCTCT TCCCCTCTAG GAATTTCACA GGTTACAGCT TCTTCATGGG
G
MAST_A145_ TATTTTGAAC ACAGAGCAAC TCCTTCTGTC TGCCTTAGAC TCCATCCTCA
GGCCCTCTCT TCCCCTCTAG GAATTTCACA GGTTACAGCT TCTTCGTGGG
A
SAV1_SE210 TACGTTGAAC ACAGAGCAAC TCCTTCTGTC TGCCTCAGAC TCCATCCTCA
GGCCCTCTCT TCCCCTCTAG GAATTTCACA GGTTACAGCT TCTTCATGGG
G

1 1 1 1 1
5 92

ASIAN2_Ema CAAAGTTCAA CGCTGCAGCC CCTTCTGCTG CTAA-GAAAC AGACTGTTAC
TTCCTGGTCC CCACTGTGTA CTCGTTCATT TCCTGAAAGT TG
FOR_DS1535 CAAAGTTCAA CGCTGCAGCC CCTTCTGCTG CTAA-GAAAC AGACTGTTAC
TTCCTGGTCC CCACTGTGTA CTCGTTCATT TCCTGAAAGT TG
MAM1_SP134 CAAAGTTCAA CACTGCAGCC CCTTCTGCTG CTAA-GAAAC AGACTGTTAC
TTCCTGGTCC CCACTGTGTA CTCGTTCATT TCCTGAAAGT TG
MAST_A146_ AAAAGTTCAA CGCTGCAGCC CCTTCTGCTG CTAAAGAAAC AGACTGTTAC
TTCCTGGTCC CCACTATGTA CTCGTTCATT TCCTGAAAGT TG
SAV1_SE210 CAAAGTTCAA CGCTGCAGCC CCTTCTGCTG CTAA-GAAAC AGACTGTTAC
TTCCTGGTCT CCACTGTGTA CTCGTTCATT TCCTGAAAGT TG

1 1 1 1 1
5 104

ASIAN2_Ema TACATTAGTC CCTAACTAGA GAGTCAGCCT GTCCCTTGAA GCTCTGAAGC
CAGGC-ATGG ACTCCTCTGC AGCTCTGGAA GTCCAGGATG GCGTTTCCCT
CCAG
FOR_DS1535 TACATTAGTC CCTAACTAGA GAGTCAGCCT GTCCCTTGAA GCTCTGAAGC
CAGGC-ATGG ACTCCTCTGC AGCTCTGGAA GTCCAGGATG GCGTTTCCCT
CCAG
MAM1_SP134 TACATTAGTC CCTAACTAGA GAGTCAGCCT GTCCCTTGAA GCTCTGAAGC
CAGGC-ATGG ACTCCTCTGC AGCTCTGGAA GTCCAGGATG GCGTTTCCCT
CCAG
MAST_A148_ TACATTAGTC CCTAACTAGA GAGTCAGCCT GTCCCTTGAA GCTCTGAAGC
CAGGCGATGG ACTCCCCTGC AGCTCTGAAA GTCCCGGATG GCGTTTCCCT
CCAG
SAV1_SE210 TACATTAGTC CCTAACTAGA GAGTCAGCCT GTCCCTTGAA GCTCTGAAGC
CAGGC-ATGG ACTCCTCTGC AGCTCTGGAA GTCCAGGATG GCGTTTCCCT
CCAG

1 1 1 1 1
5 98

ASIAN2_Ema AGCCCTTCCC AGCGTCCCTA CCTGGGCCTC CCTCACCTGG GGTGGATGTC
CACGAGCAGC ACAAGCGTCT GCATGGTGAT CACATCGATG CGTTTGCA
FOR_DS1535 AGCCCTTCCC AGCGTCCCTA CCTGGGCCTC CCTCACCTGG GGTGGATGTC
CACGAGCAGC ACAAGCGTCT GCATGGTGAT CACATCGATG CGTCTGCA
MAM1_SP134 AGCCCTTCCC AGCGTCCCTA CCTGGGCCTC CCTCACCTGG GGTGGATGTC
CACGAGCAGC ACAAGCGTCT GCATGGTGAT CACATCGATG CGTTTGCA
MAST_A155_ AGCCCTTCCC CGAGTCCCTG CCTGGGCCCC CCTCACCTGG GGCGGATGTC
CACGAGCAAC ACAAGCGTCT GCATGGTGAT CACATCGATG CGTTTGCA
SAV1_SE210 AGCCCTTCCC AGCGTCCCTA CCTGGGCCTC CCTCACCTGG GGTGGATGTC
CACGAGCAGC ACAAGCGTCT GCATGGTGAT CACATCGATG CGTCTGCA

1 1 1 1 1
5 107

ASIAN2_Ema TGCACTTGAG AGG-CACTGG TGCAGACCTC CAGGCCCCCA GCCCCACCC-
TGCCAAGTGC TGTGGCCCC- AAGCAACACC AGTGTGGAGC CCTGGACCTC
TGGACCC
FOR_DS1535 TGCACTTGAG AGG-CACTGG TGCAGACCTC CAGGCCCCCA GCCCCACCC-

```
              TGCCACGTGC TGTGGCCCC- AAGCAACACC AGTGTGGAGC CCTGGACCTC
              TGGACCC
MAM1_SP134    TGCACTTGAG AGG-CACTGG TGCAGATGTC CAGGCCCCCA GCCCCACCC-
              TGCCAAGTGC TGTGGCCCC- AAGCAACACC AGTGTGGAGC CCTGGACCTC
              TGGACCC
MAST_A156_    TGCACTTGAG AGG-CACTGG TGCAGACCTC CAGGCCCCCA GCCCCACCCC
              TGCCAAGAGC TGTGGCCCCC AAGCAACACC AGTGTGGACC CCTGGACCTC
              TGGACCC
SAV1_SE210    TGCACTTGAG AGG-CACTGG TGCAGACCTC CAGGCCCCCA GCCCCACCC-
              TGCCAAGTGC TGTGGCCCC- AAGCAACATC AGTGTGGAGC CCTGGACCTC
              TGGACCC

 1 1 1 1 1
5 98

ASIAN2_Ema    TTCTTCAGAA TCAGAGCTGC CCTAGGCATA AAATTCTTAA CCCTCTAGCT
              TAATCCAAAT GCAATGAGTC AGTCAGAAAG TAAA-TGGGA ACCACTGG
FOR_DS1535    TTCTTCAGAA TCAGAGCTGC CCTAGGCATA AAATTCTTAA CCCTCTAGCT
              TAATCCAAAT GCAATGAGTC AGTCAGAAAG TAAA-TGGGA ACCACTGG
MAM1_SP134    TTCTTCAGAA TCAGAGCTGC CCTAGGCATA AAATTCTTAA CCCTCTAGCT
              TAATCCAAAT GCAATGAGTC AGTCAGAAAG TAAA-TGGGA ACCACTGG
MAST_A165_    TTCTTCAGAA TCAGAGCTGC CCTAGGCATA AAATTCTTAA CCCTCTAGCT
              TAATCCAAAT GCAATGAGTC AGTCAGAAAG TAAAATGGGA ATCACTGG
SAV1_SE210    TTCTTCAGAA TCAGAGCTGC CCTAGGCATA AAATTCTTAA CCCTCTAGCT
              TAATCCAAAT GCAATGAGTC AGTCAGAAAG TAAA-TGGGA ACCACTGG

 1 1 1 1 1
5 98

ASIAN2_Ema    ATTCATTCTT TCACTTCTCT TCCCCAACTT GTGCTCAGGG CCCTCATGCT
              GCCTATCTGT CCAGGAATGG CGGAAAATTA GATATCACAT AAAAGTAA
FOR_DS1535    ATTCATTCTT TCACTTCTCT TCCCCAACTT GTGCTCAGGG CCCTCATGCT
              GCCTATCTGT CCAGGAATGG CGGAAAATTA GATATCACAT AAAAGTAA
MAM1_SP134    ATTCATTCTT TCACTTCTCT TCCCCAACTT GTGCTCAGGG CCCTCATGCT
              GCCTATCTGT CCAGGAATGG CGGAAAATTA GATATCACAT AAAAGTAA
MAST_A173_    ATTTATTCTT TCACTTCTCT TCTCCAACTT GTGCTCAGGG CCCTCATGCT
              GCCTATCTGT CCAGGAATGG CAGAAAATTA GATATCACAT AAAAGTAA
SAV1_SE210    ATTCATTCTT TCACTTCTCT TCCCCAACTT GTGCTCAGGG CCCTCATGCT
              GCCTATCTGT CCAGGAATGG CGGAAAATTA GATATCACAT AAAAGTAA

 1 1 1 1 1
5 94

ASIAN2_Ema    GAATGATATC ATACTGCCCC TTCAGAGATM TGCCGCCATC AGGTTATTAG
              GTGGTTTCTG TGCCCCCGTG CATTTAAAAT GGCCTAAAA- TTAA
FOR_DS1535    GAATGATATC ATACTGCCCC TTCTGAGATC TGCCRCCATC AGGTTATTAG
              GTGGTTTCTG TGCCCCCGTG CATTTAAAAT GGCCTAAAA- TTAA
MAM1_SP134    GAATGATATC ATACTGCCCC TTCAGAGATC TGCCGCCATC AGGTTATTAG
              GTGGTTTCTG TGCCCCCGTG CATTTAAAAT GGCCTAAAA- TTAA
MAST_A178_    GAATGATATC ATACTGTTCC TTCTGAGATC TGCCGCCATC AGGTTATTAG
              GTGGTTTCTG TGCCCCCGTG CATTTAAAAT GGCCTAAAAA TTAA
SAV1_SE210    GAATGATATC ATACTGCCCC TTCTGAGATC TGCCACCATC AGGTTATTAG
              GTGGTTTCTG TGCCCCCGTG CATTTAAAAT GGCCTAAAA- TTAA

 1 1 1 1 1
5 97

ASIAN2_Ema    CCGTGATGTC CTTTTTTGGC TTTCGGTCTT TCCTGATGAT GTGTCCAGAG
              TAAGTAAGCT AAGTCTTGCC ATCCTTGCTG CCAAGGAACC TTCAGGA
FOR_DS1535    CCGTGATGTC CTTTTTTGGC TTTCGGTCTT TCCTGATGAT GTGTCCAGAG
              TAAGTAAGCT AAGTCTTGCC ATCCTTGCTG CCAAGGAACC TTCAGGA
MAM1_SP134    CCGTGATGTC CTTTTT-GGC TTTCGGTCTT TCCTGATGAT GTGTCCAGAG
              TAAGTAAGCT AAGTCTTGCC ATCCTTGCTG CCAAGGAACC TTCAGGA
MAST_A179_    TTGTGATGTC CTTTTTTGGC TTTTGGTCTT TCCTGATGAT GTGTCCAGAG
              TAAGTAAGCT AAGTCTTGCC ATCCTTGCTG CCAAGGAACC TTCAGGA
SAV1_SE210    CCGTGATGTC CTTTTTTGGC TTTCGGTCTT TCCTGATGAT GTGTCCAGAG
              TAAGTAAGCT AAGTCTTGCC ATCCTTGCTG CCAAGGAACC TTCAGGA

 1 1 1 1 1
5 99
```

```
ASIAN2_Ema   AGGAATTCTT TCTAGGGAG- ---ACATTTA GAGACAAGTA AATAGCAGAG
             GGCTGCAGAC ACAGCCTCCC AGCACAGAAG AAAGGAGGGC TGGCAACAG
FOR_DS1535   AGGAATTCTT TCTAGGGAG- ---ACATTTA GAGACAAGTA AATAGCAGAG
             GGCTGCAGAC ACAGCCTCCC AGCACAGAAG AAAGGAGGGC TGGCAACAG
MAM1_SP134   AGGAATTCTT TCTAGGGAG- ---ACATTTA GAGACAAGTA AATAGCAGAG
             GGCTGCAGAC ACAGCCTCCC AGCACAGAAG AAAGGAGGGC TGGCAACAG
MAST_A180_   AGGAATTCTT TCTAGGGAGG GAGACATTTA GAGACAAGTA AATAGCAGAG
             GGCTGCAGAC ACAGCCTCCC AGCATAGAAG AAAGGAGGGC TGGCAACAG
SAV1_SE210   AGGAATTCTT TCTAGGGAG- ---ACATTTA GAGACAAGTA AATAGCAGAG
             GGCTGCAGAC ACAGCCTCCC AGCACAGAAG AAAGGAGGGC TGGCAACAG

 1 1 1 1 1
5 105

ASIAN2_Ema   GAGAAATAAC CAGCACAGCC TCTCGCTGCC CCTTGGCTGA CTTCCTTGGA
             GTCCGTTTCA CCGAGGAACT GGCTCGGTGA CTTGAGCCTA AGCTCTGGCT
             CACAT
FOR_DS1535   GAGAAATAAC CAGCACAGCC TCTCGCTGCC CCYTGGCTGA CTTCCTTGGA
             GTCCGTTTCA CCGAGGAACT GGCTCCGTGA CTTGAGCCTA AGCTCCGGCT
             CACAT
MAM1_SP134   GAGAAATAAC CAGCACAGCC TCTCGCTGCC CCTTGGCTGA CTTCCTTGGA
             GTCCGTTTCA CCGAGGAACT GGCTCGGTGA CTTGAGCCTA AGCTCTGGCT
             CACAT
MAST_A186_   GAGAAATAAC TAGCACAGCC CCTCGCTGCC TCCTGGCTGA CTTCCTTGGA
             GTCCGTTTCA CCGAGGGACT GGCTCCGTGA CTTGAGCCTA AGCTCCGGCT
             TACAT
SAV1_SE210   GAGAAATAAC CAGCACAGCC TCTCGCTGCC CCCTGGCTGA CTTCCTTGGA
             GTCCGTTTCA CCGAGGAACT GGCTCCGTGA CTTGAGCCTA AGCTCCGGCT
             CACAT

 1 1 1 1 1
5 93

ASIAN2_Ema   AAATGAAGTC GAGGGCTGGG GGTTCCCCAC CTCCTGAAGG GAAGGCAGCT
             AAYGTTAACT GAATGCTTGC TCTGTTGTTG GAGCCTTCGT GTC
FOR_DS1535   AAATGAAGTC GAGGGCTGGG GGTTCCCCAC CTCCTGAAGG GAAGGCAGCT
             AACGTTAACT GAATGCTTGC TCTGTTGTTG GAGCCTTCGT GTC
MAM1_SP134   AAATGAAGTC GAGGGCTGGG GGTTCCCCAC CTCCTGAAGG GAAGGCAGCT
             AAYGTTAACT GAATGCTTGC TCTGTTGTTG GAGCCTTCGT GTC
MAST_A190_   AAATGAAGTC GAGGGCTGGG GGTTCCC-AC CTCCTAAAGG GAAGGCAGCT
             AACGTTAACT GAATGCTTGC TCTATTGTTG GAGCCTTCGC GTC
SAV1_SE210   AAATGAAGTC GAGGGCTGGG GGTTCCCCAC CTCCTGAAGG GAAGGCAGCT
             AACGTTAACT GAATGCTTGC TCTGTTGTTG GAGCCTTCGT GTC

 1 1 1 1 1
5 103

ASIAN2_Ema   ACTTGGCCCT TTCTGTGTCT TACGCTGTTG CCAGCAGTAA GGGGGTTCTC
             AAAAATTTCC CACTGCAGCT CATTTTAATG TGGGAAGTTG CCAGGGAAGC
             TTC
FOR_DS1535   ACTTGGCCCT TTCTGTGTCT TCCGCTGTTG CCAGCAGTAA GGGGGTTCTC
             AAAAATTCCC CACTGCAGCT CATTTTAATG TGGGAAGTTG CCAGGGAAGC
             TTC
MAM1_SP134   ACTTGGCCCT TTCTGTGTCT TCCGCTGTTG CCAGCAGTAA GGGGGTTCTC
             AAAAATTCCC CACTGCAGCT CATTTTAATG TGGGAAGTTG CCAGGGAAGC
             TTC
MAST_A191_   ACTTGGCCCT TTCTATGTCT TATGCTGTTG CCAGCAGTAA GGGGGTTCTC
             AAAAATTCCC CACTGCAGCT CATTTTAATG TGGGAAGTTG CCAGGGAAGC
             TTC
SAV1_SE210   ACTTGGCCCT TTCTGTGTCT TACGCTGTTG CCAGCAGTAA GGGGGTTCTC
             AAAAATTTCC CACTGCAGCT CATTTTAATG TGGGAAGTTG CCAGGGAAGC
             TTC

 1 1 1 1 1
5 117

ASIAN2_Ema   GAGGGCTGTT GGTGGGGGAA ACGAAGGGGG AGAGGGCACC ACTTTTCCTA
             GCCTTTGGGC CCAGATCTCG GGGTGGTGGT GGTGGAGTGG GCTGAGGTAA
             GACATCCCGG ATTCCCG
FOR_DS1535   TAGGGCTGTT GGTGGGGGAA ACGAAGGGGG AGAGGGCACC ACTTTTCCTA
             GCCTTTGGGC CCAGATCTCG GGGCGGTGGT GGTGGAGTGG GCTGAGGTAA
```

GACATCCCGG ATTCCCG
MAM1_SP134 GAGGGCTGTT GGTGGGGGAA ACGAAGGGGG AGAGGGCACC ACTTTTCCTA
GCCTTTGGGC CCAGATCTCG GGGTGGTGGT GGTGGAGTGG GCTGAGGTAA
GACATCCCGG ATTCCCG
MAST_A195_ GAGGGCTGTT GGCGGGG-AA ACGAAGGGG- AGAGGGCACC ACTTCTCCTA
GTCTTTGGGC CCAGATCTCG GGGTGGTCAT AATGAAGTGG ACTGAGGTAA
GACATCCCGG ATTCCCG
SAV1_SE210 GAGGGCTGTT GGTGGGGGAA ACGAAGGGGG AGAGGGCACC ACTTTTCCTA
GCCTTTGGGC CCAGATCTCG GGGTGGTGGT GGTGGAGTGG GCTGAGGTAA
GACATCCCGG ATTCCCG

1 1 1 1 1
5 91

ASIAN2_Ema ATTTTCATAT GGATCCCCAG GCTCAGGGAA GGGACCTTTC TTAAGTCTTG
CTTCTCCTGG AGGTTGACCA ACCAATAGCC CACTGGCTAT A
FOR_DS1535 ATTTTCATAT GGATCCCCAG GCTCAGGGAA GGGACCTTTC TTAAGTCTTG
CTTCTCCTGG AGGTTGACCA ACCAATGGCC CACTGGCTAT A
MAM1_SP134 ATTTTCATAT GGATCCCCAG GCTCAGGGAA GGGACCTTTC TTAAGTCTTG
CTTCTCCTGG AGGTTGACCA ACCAATAGCC CACTGGCTAT A
MAST_A196_ GTTTTTATAT GGATCCCCAG GCTCAGGGAA GGGACCTTTC TTAAGTCTTG
CTTCTCCTGG AGGTTGACCA ACCAATAGCC CGCTGGCTAT A
SAV1_SE210 ATTTTCATAT GGATCCCCAG GCTCAGAGAA GGGACCTTTC TTAAGTCTTG
CTTCTCCTGG AGGTTGACCA ACCAATGGCC CACTGGCTAT A

1 1 1 1 1
5 108

ASIAN2_Ema AAGTACCCCA TTAATTCTAA ACACAGCTGT CAGAGGGTCT TCAACAACCT
AGAGCTTTCT AAGAGGGGCC GCAGTGTCTC TACTAGGCAG TACCCACCTG
GTATGGAT
FOR_DS1535 AAGTACCCCA TTAATTCTAA ACACAGCTGT CAGAGGGTCT TCAACAACCT
AGAGCTTTCT AAGAGGGGCC GCAGTGTCTC TACTAGGCAG TACCCACCTG
GTATGGAT
MAM1_SP134 AAGTACCCCA TTAATTCTAA ACACAGCTGT CAGAGGGTCT TCAACAACCT
AGAGCTTTCT AAGAGGGGCT GCAGTGTCTC TACTAGGCAG TACCCACCTG
GTATGGAT
MAST_A199_ AAGTACCCCA TTAATTCTAA ACACAGCTGT CAGAGGGTCT TCAACAACCT
AGAGCTTTCT AAGAGGGGCC TCAGTGTCTC TACTAGGCAG TACCCACCTG
GTATGGAT
SAV1_SE210 AAGTACCCCA TTAATTCTAA ACACAGCTGT CAGAGGGTCT TCAACAACCT
AGAGCTTTCT AAGAGGGGCT GCAGTGTCTC TACTAGGCAG TACCCACCTG
GTATGGAT

1 1 1 1 1
5 111

ASIAN2_Ema GAGGCTTCCT CTGGGAGGCC AGCCCCCTTC C-AGGTGGGT GTCCCCCAGG
AGACACAATT CCGTCCTTAG CATGGCCCCA CAAGACACAC GGCCTGCTGC
TTCTGAAAAA A
FOR_DS1535 GAGGCTTCCT CTGGGAGGCC AGCCCCCTTC C-AGGTGGGT GTCCCCCAGG
AGACACAATT CCGTCCTTAG CATGGCCCCA CAAGACACAC GGCCTGCTGC
TTCTGAAAAA A
MAM1_SP134 GAGGCTTCCT CTGGGAGGCC AGCCCCCTTC C-AGGTGGGT GTCCCCCAGG
AGACACAATT CCGTCCTTAG CATGGCCCCA CAAGACACAC GGCCTGCTGC
TTCTGAAAAA A
MAST_A200_ GAGGCTTCCT CTAGGAGGCC AGCCCCCTTC CCAGGTGGGT GTCCCCCAGG
AGACACAATT CCGTCCTTAG CATGGCCCCA CAAGACACAC GGCCTGCTGT
TTCTGAAAAA A
SAV1_SE210 GAGGCTTCCT CTGGGAGGCC AGCCCCCTTC C-AGGTGGGT GTCCCCCAGG
AGACACAATT CCGTCCTTAG CATGGCCCCA CAAGACACAC GGCCTGCTGC
TTCTGAAAAA A

1 1 1 1 1
5 90

ASIAN2_Ema TGTGGGCCCA GGGGATGGGC CTCTGCTGGA GCCCATCATG GATCATGGAT
ACCTACCAGA TCAAGGATGG AGCCCCTCGT GGCATAGTGG
FOR_DS1535 TGTGGGCCCA GGGGATGGGC CTCTGCTGGA GCCCATCATG GATCATGGAT
ACCTACCAGA TCAAGGATGG AGCCCCTCGT GGCATAGTGG
MAM1_SP134 TGTGGGCCCC GGGGATGGGC CTCTGCTGGA GCCCATCATG GATCATGGAT

```
              ACCTACCAGA TCAAGGATGG AGCCCCTCGT GGCATAGTGG
MAST_A201_    TGTGGGCCCA GGGGATGGGC CTCTGCTGGA GCCCATCATG GATCATTGAT
              ACCTACCAGA TCAAGGATGG AGCCCCTCGT GGCACAGTGG
SAV1_SE210    TGTGGGCCCA GGGGATGGGC CTCTGCTGGA GCCCATCATG GATCATGGAT
              ACCTACCAGA TCAAGGATGG AGCCCCTCGT GGCATAGTGG

 1 1 1 1 1
5 100

ASIAN2_Ema    CCAAGAATGC CTTAATAGTC ATTAATTTCA CAAAGCTTCT TCTATATATG
              TCAGCAAAAT ATACTAAAAA TATCCTCCTG TGATAATGAA TGATGTCTTG
FOR_DS1535    CCAAGAATGC CTTAATAGTC ATTAATTTCA CAAAGCTTCT TCTATATATG
              TCAGCAAAAT ATACTAAAAA TATCCTCCTG TGATAATGAA TRATGTATTG
MAM1_SP134    CCAAGAATGC CTTAATAGTC ATTAATTTCA CAAAGCTTCT TCTATATATG
              TCAGCAAAAT ATACTAAAAA TATCCTCCTG TGATAATGAA TGATGTATTG
MAST_A205_    CCAAGAATGC CTTAATAGTC ATTAATTTCA CAAAGCTTCT ---ATATATG
              TCAGCAAAAT ATACTAAAAA TATCCTCCTG CGATAATGAA TGATGTATTG
SAV1_SE210    CCAAGAATGC CTTAATAGTC ATTAATTTCA CAAAGCTTCT TCTATATATG
              TCAGCAAAAT ATACTAAAAA TATCCTCCTG TGATAATGAA TGATGTATTG

 1 1 1 1 1
5 104

ASIAN2_Ema    CCCCCACACC ATCCCTGCTG GCACCTCCTC TGCAGGAAAG GGAGTGAGCT
              GGAATCAGCT CCTGTGCAGA AAGGGTTAAT TCCCTCTT-G GAGGGATTAT
              ACCG
FOR_DS1535    CCCCCACACC ATCCCTGCTG GCACCTCCTC TGCAGGAAAG GGAGTGAGCT
              GGAATCAGCT CCTGTGCAGA AAGGGTTAAT TCCCTCTT-G GAGGGATTAT
              ACTG
MAM1_SP134    CCCCCACACC ATCCCTGCTG GCACCTCCTC TGCAGGAAAG GGAGTGAGCT
              GGAATCAGCT CCTGTGCAGA AAGGGTTAAT TCCCTCTT-G GAGGGATTAT
              ACCG
MAST_A208_    CCTCCACACC ATCCCTGCTG GCACCTCCTC GGCAGGAAAG GGAGTGAGCT
              GGAATCAGCT CCTGTGCAGA AAGGGTTAAT TCCCTCTTAA AAAAGATTAT
              ACCA
SAV1_SE210    CCCCCACACC ATCCCTGCTG GCACCTCCTC TGCAGGAAAG GGAGTGAGCT
              GGAATCAGCT CCTGTGCAGA AAGGGTTAAT TCCCTCTT-G GAGGGATTAT
              ACCC

 1 1 1 1 1
5 98

ASIAN2_Ema    CAGTTATACT TCTTTTTCAA ACCCAGAGAC TTCCAGCATT TGGACCCTTC
              TATTTATCAG ATTTCCACCT ATTACACATT CCCTGTCATC AGTTTCTT
FOR_DS1535    CAGTTATACT TCTTTTTCAA ACCCAGAGAC TTCCAGCATT TGGACCCTTC
              TATTTATCAG ATTTCCACCT ATTACACATT CCCTGTCATC AGTTTCTT
MAM1_SP134    CAGTTATACT TCTTTTTCAA ACCCAGAGAC TTCCAGCATT TGGACCCTTC
              TATTTATCAG ATTTCCACCT ATTACACATT CCCTGTCATC AGTTTCTT
MAST_A212_    TAGTTATATT TTTTTTTCAA ACCCAGAGAC TTCCAGCATT TGGACCCTTC
              TATTTATCAG ATTTCCACCT ATTACACATT CCCTGTCATC AGTTTCTT
SAV1_SE210    CAGTTATACT TCTTTTTCAA ACCCAGAGAC TTCCAGCATT TGGACCCTTC
              TATTTATCAG ATTTCCACCT ATTACACATT CCCTGTCATC AGTTTCTT

 1 1 1 1 1
5 105

ASIAN2_Ema    TGGCCTGTCT GCCACCCTCC ATTT-CTCCC CTTCAATCCA CCAGCCATCT
              TCCACGCTCC CTGGCTGGTC AATACCCACC CACTTTCAAG TTCTAACAGT
              GGCTC
FOR_DS1535    TGGCCTGTCT GCCACCCTCC ATTT-CTTCC CTTCAATCCA CCAGCCATCT
              TCCACGCTCC CTGGCTGGTC AATACCCACC CACTTTCAAG TTCTAACAGT
              GGCTC
MAM1_SP134    TGGCCTGTCT GCCACCCTCC ATTT-CTCCC CTTCAATCCA CCAGCCATCT
              TCCACGCTCC CTGGCTGGTC AATACCCACC CACTTTCAAG TTCTAACAGT
              GGCTC
MAST_A415_    TGGCCTCTCT GCCACCCTCC ATTTTCTTCC CTTCAATCCA CCAGCCATCT
              GCCACGCTCC -TGGCTGGTC ACTACCCACC CACTTTCAAG TTCTAACAGT
              GGCTC
SAV1_SE210    TGGCCTGTCT GCCACCCTCC ATTT-CTCCC CTTCAATCCA CCAGCCATCT
              TCCACGCTCC CTGGCTGGTC AATACCCACC CACTTTCAAG TTCTAACAGT
              GGCTC
```

 1 1 1 1 1
5 97

```
ASIAN2_Ema   CCCAAGGAGC TGGATCCCTC TGCTGTGCTC CCCTTGGATT GTCTTCTTGC
             ATTTGTGTTC TTTGATGCTA ACTGGTGTGG CTACTTGCAC CGGAGAG
FOR_DS1535   CCCAAGGAGC TGGATCCCTC TGCTGTGCTC CCCTTGGATT GTCTTCTTGC
             ATTTGTGTTC TTTGATGCTA ACTGGTGTGG CTACTTGCAC CGGAGAG
MAM1_SP134   CCCAAGGAGC TGGATCCCTC TGCCGTGCTC CCCTTGGATT GTCTTCTTGC
             ATTTGTGTTC TTCGATGCTA ACTGGTGTGG CTACTTGCAC CGGAGAG
MAST_A355_   TCCAAGGAGC TGGATCCCTC TGCTGTGCTC CCCTTGGATT GTCTTCTTGC
             ATTTGTGTTC TTTGATGCTA ACTGGTGTGG CTACTTGCAC CGGAGAG
SAV1_SE210   CCCAAGGAGC TGGATCCCTC TGCTGTGCTC CCCTTGGATT GTCTTCTTGC
             ATTTGTGTTC TTTGATGCTA ACTGGTGTGG CTACTTGCAC CGGAGAG
```

 1 1 1 1 1
5 95

```
ASIAN2_Ema   TAAGAACCTC TGTAACAGTG GGAGGAGAGT ATCATGGCCA AGAAAGCTCT
             GCTCAAGAGA AGGCACAGAG CCTGGGCACA GACGCTGGGC CAGAG
FOR_DS1535   TAAGAACCTC TGTAACAGTG GGAGGAGAGT ATCATGGCCA AGAAAGCTCT
             GGTCAAGAGA AGGCACAGAG CCTGGGCACA GACGCTGGGC CAGAG
MAM1_SP134   TAAGAACCTC TGTAACAGTG GGAGGAGAGT ATCATGGCCA AGAAAGCTCT
             GCTCAAGAGA AGGCACAGAG CCTGGGCACA GACGCTGGGC CAGAG
MAST_A214_   TAAGAACCTC TGTAACAGTG GGAGGAGAGT ATCATGGCCA AGAAAGCTCT
             GATCAAGAGA AGGCACAGAG CCTGGGCACA GACGCCGGGC CAGAA
SAV1_SE210   TAAGAACCTC TGTAACAGTG GGAGGAGAGT ATCATGGCCA AGAAAGCTCT
             GCTCAAGAGA AGGCACAGAG CCTGGGCACA GACGCTGGGC CAGAG
```

 1 1 1 1 1
5 112

```
ASIAN2_Ema   CAGTAAAACA CGTGTTAAAG ATTACAAATT GTTTAACAAG TTTTGGCAAA
             CAAGTCCCCT GGTGGTCTTG AAATTTCTGA GAAGCAGATG GAAGAGGTAC
             AGTTTTATAG AT
FOR_DS1535   CAGTAAAACA CGTGTTAAAG ATTACAAATT GTTTAACAAG TTTTGGCAAA
             CAAGTCCCCT GGTGGTCTTG AAATTTCTGA GAAGCAGATG GAAGAGGTRC
             AGTTTTATAG AT
MAM1_SP134   CAGTAAAACA CGTGTTAAAG ATTACAAATT GTTTAACAAG TTTTGGCAAA
             CAAGTCCCCT GGTGGTCTTG AAATTTCTGA GAAGCAGATG GAAGAGGTAC
             AGTTTTATAG AT
MAST_A215_   TAGTAAAACA CGTGTTAAAG ATTACAAATT GTTTAACAAG TTTTGGCAAA
             CAAGTCCCCT GGTGGTCTTG AAATTTCTGA GAAGCAGATG GAAGAGGTAC
             AGTTTTATAG AT
SAV1_SE210   CAGTAAAACA CGTGTTAAAG ATTACAAATT GTTTAACAAG TTTTGGCAAA
             CAAGTCCCCT GGTGGTCTTG AAATTTCTGA GAAGCAGATG GAAGAGGTAC
             AGTTTTATAG AT
```

 1 1 1 1 1
5 93

```
ASIAN2_Ema   CTCAGAGTGG GGGCCTAACG CCAGATGTAG TGCTCTGGTG AGTCCAGGGT
             GGGGTGTTAC TCAGCCTACA ATCCCYCACC CACACTTCGC CAG
FOR_DS1535   CTCAGAGTGG GGGCCTAATG CCAGATGTAG TGCTCTGGTG AGTCCAGGGT
             GGGGTGTTAC TCAGCCTACA ATCCCCCACC CACACTTCGC CAG
MAM1_SP134   CTCAGAGTGG GGGCCTAACA CCAGATGTAG TGCTCTGGTG AGTCCAGGGT
             GGGGTGTTAC TCAGCCTACA ATCCCCCACC CACACTTCGC CAG
MAST_A216_   CTCAGAGTGG GG-CCTAACA CCAGATGTAG TGCTCTGGTG AGTCCAGGGT
             GGGGTGTTAC TCAGCCCACA GTCCCCCACC CACACTTCCC CAA
SAV1_SE210   CTCAGAGTGG GGGCCTAATG CCAGATGTAG TGCTCTGGTG AGTCCAGGGT
             GGGGTGTTAC TCAGCCTACA ATCCCCCACC CACACTTCGC CAG
```

 1 1 1 1 1
5 100

```
ASIAN2_Ema   GATATGTCCT GGAGAGGGCT CTGGAAGATG CCCTTTCTCA GGGATGTTGG
             AGAAAGGCAG CCACCTGCTA TGGGGCCTGG CATTCTTGCC AGGGGCTGGG
FOR_DS1535   GATATGTCCT GGAGAGGGCT CTGGAAGATG CCCTTTCTCA GGGATGTTGG
             AGAAAGGCAG CCACCTGCTA TGGGGCCTGG CATTCTTGCC AGGGGCTGGG
MAM1_SP134   GATATGTCCT GGAGAGGGCT CTGGAAGATG CCCTTTCTCA GGGATGTTGG
             AGAAAGGCAG CCACCYGCTA TGGGGCCTGG CATTCTTGCC AGGGGCTGGG
```

```
MAST_A217_    GATATGTCCT GGAGAGGGCT CTGGAAGATG CCCTTTCTCA GGGATGTTGG
              AGAAAAGCAG CCACCTGCTA TGGGGCCTGG CATTCTTGCC AGGGGCTGGG
SAV1_SE210    GATATGTCCT GGAGAGGGCT CTGGAAGATG CCCTTTCTCA GGGATGTTGG
              AGAAAGGCAG CCACCTGCTA TGGGGCCTGG CATTCTTGCC AGGGGCTGGG

 1 1 1 1 1
5 99

ASIAN2_Ema    CTGGTCCTCC CTCGTTTCTG GACCTTTGCT CATCCTGCCC TCCCTATAAT
              GGCCGCCTTT TCCCCACTCC TCCTATCCCA ATCTCAGTGC TTTCTGTCT
FOR_DS1535    CTGGTCCTCC CTCGTTTCTG GACCTTTGCT CATCCTGCCC TCCCTATAAT
              GGCCGCCTGT TCCCCACTCC TCCTATCCCA ATCTCAGTGC TTTCTGTCT
MAM1_SP134    CTGGTCCTCC CTCGTTTCTG GACCTTTGCT CATCCTGCCC TCCCTATAAT
              GGCCGCCTTT TCCCCACTCC TCCTATCCCA ATCTCAGTGC TTTCTGTCT
MAST_A219_    CTGGTCCTCC CTCGTTTCTG GACCTTTGCT CATCCTGCCC TCCCTATAAT
              GGCCGCCTGT TCCCCACTCC TCCTATCCCA ATCTCAGTGC TTTCTGTCT
SAV1_SE210    CTGGTCCTCC CTCGTTTCTG GACCTTTGCT CATCCTGCCC TCCCTATAAT
              GGCMGCCTGT TCCCCACTCC TCCTATCCCA ATCTCAGTGC TTTCTGTCT

 1 1 1 1 1
5 103

ASIAN2_Ema    GGGCGACCTT --CTCGCTCT CTGGCTAAGC CCATGGACCT CTTACAACCT
              GTTCACGTGA CGTGGGTTGT ACAAATACTC AGGCCCACCC AATGTTCTCC
              TTC
FOR_DS1535    GGGCGACCTT --CTCGCTCT CTGGCTAAGC CCATGGACCT CTCACAACCT
              GTTCACGTGA CGTGGGTTGT ACAAATACTC AGGCCCACCC AATGTTCTCC
              TTC
MAM1_SP134    GGGCGACCTT --CTCGCTCT CTGGCTAAGC CCATGGACCT CTTACAACCT
              GTTCACGTGA CGTGGGTTGT ACAAATACTC AGGCCCACCC AATGTTCTCC
              TTC
MAST_A220_    CGTCGACCTC GCCTCGCTCT CTGGCTAAGC CCATGGACCT CTCGCAACCT
              GTTCACATAA TGTGGGTTAT ACAAATACTC AGGCCCACCC AATGTTCTCC
              TTC
SAV1_SE210    GGGCGACCTT --CTCGCTCT CTGGCTAAGC CCATGGACCT CTTACAACCT
              GTTCACGTGA CGTGGGTTGT ACAAATACTC AGGCCCACCC AATGTTCTCC
              TTC

 1 1 1 1 1
5 105

ASIAN2_Ema    AGTTTTCTCT TCCAAGGCCC CTGAGTTCTT CTGGCTTTTA GACATCAAAG
              CGCTTATGTG CCTGTTCTTT TCAGAAGCTG CTCTCTCTCC CGAGAGACTT
              TTCTT
FOR_DS1535    AGTTTTCTCT TCCAAGGCCC CTGAGTTCTT CTGGCTTTTA GACATCAAAG
              CGCTTATGTG CCTGTTCTTT TCAGAAGCTG CTCTCTCTCC CGAGAGACTT
              TTCTT
MAM1_SP134    AGTTTTCTCT TCCAAGGCCC CTGAGTTCTT CTGGCTTTTA GACATCAAAG
              CGCTTATGTG CCTGTTCTTT TCAGAAGCTG CTCTCTCTCC CGAGAGACTT
              TTCTT
MAST_A294_    AGTTTTCTCT TCTAAGGCCC CTGAGTTCTT CTGGCTTTTA GACATCAAAG
              CGCTTATGTG CCTGTTCTTT TCAGAAGCTG CTCTCTCTCC CAAGAGACTT
              TTCTT
SAV1_SE210    AGTTTTCTCT TCCAAGGCCC CTGAGTTCTT CTGGCTTTTA GACATCAAAG
              CGCTTATGTG CCTGTTCTTT TCAGAAGCTG CTCTCTCTCC CGAGAGACTT
              TTCTT

 1 1 1 1 1
5 95

ASIAN2_Ema    TGAGCTTCAG GTGCTGCTGG GAGGGGCCAT CAAGGCAATC CCTCCCAGGG
              AGGAGGGCTG GTTCTGTGGG CAACGCGGCC AGAGTGAGTA ACCAG
FOR_DS1535    TGAGCTTCAG GTGCTGCTGG GAGGGGCCAT CAAGGCAATC CCTCCCAGGG
              AGGAGGGCTG GTTCTGTGGG CAACKCGGCC AGAGTGAGTA ACCAG
MAM1_SP134    TGAGCTTCAG GTGCTGCTGG GAGGGGCCAT CAAGGCAATC CCTCCCAGGG
              AGGAGGGCTG GTTCTGTGGG CAACGCGGCC AGAGTGAGTA ACCAG
MAST_A222_    TGAGCTTCAG GTGCTGCTGG GAGGGGCCAT CAAGGCAATC CCTCCCAGGG
              AGGAGGGCTG GTTCTGTGGG CAAAGCTGCC AGAGTGAGTA ACCAG
SAV1_SE210    TGAGCTTCAG GTGCTGCTGG GAGGGGCCAT CAAGGCAATC CCTCCCAGGG
              AGGAGAGCTG GTTCTGTGGG CAACGTGGCC AGAGTGAGTA ACCAG
```

```
 1 1 1 1 1
5 91

ASIAN2_Ema    TCAATATCTC CTCACCTGGA CCTCAGACCC TTGTTTTCCA TTCCACAAAG
              CCACAGGGAC AAGATTCAGC CTCTCCCACC CCAGTGCACA R
FOR_DS1535    TCAATATCTC CYCACCTGGA CMTCAGACCC TTGTTTTCCA TTCCACAAAS
              CCACAGGGAC AAGATTCAGC CTCTCCCACC CCAGTGCACA G
MAM1_SP134    TCAATATCTC CTCACCTGGA CCTCAGACCC TTGTTTTCCA TTCCACAAAG
              CCACAGGGAC AAGATTCAGC CTCTCCCACC CCAGTGCACA G
MAST_A223_    TCAATATCTC CTCACCTGGA CCTCAGACCC TTGTTTTCCA TTCCACAAAG
              CCACAGGGCC AAGATTCAGC CACTCCCACT CCAGTGCACA G
SAV1_SE210    TCAATATCTC CTCACCTGGA CCTCAGACCC TTGTTTTCCA TTCCACAAAG
              CCACAGGGAC AAGATTCAGC CTCTCCCACC CCAGTGCACA G

 1 1 1 1 1
5 134
ASIAN2_Ema    CACTTGTTCT ATATTTTGTA TTACCTGTAG TGGATAAAAA ATAGCAGAGC
              AAAAACTAGG CTTTCATCAG -CTTTTTTGT TTTGTTTTGT TTT-----CA
              GGGACAAAAC ATTTAAACAA ACATGTTTTT CTTT
FOR_DS1535    CACTTGTTCT ATATTTTGTA TTACCTGTAG TGGATAAAAA ATAGCAGAGC
              AAAAACTAGG CTTTCATCAG -CTTTTTTGT TTTGTTTTGT TTTGTTTTCA
              GGGACAAAAC ATTTAAACAA ACATATTTTT CTTT
MAM1_SP134    CACTTGTTCT ATATTTTGTA TTACCTGTAG TGGATAAAAA ATAGCAGAGC
              AAAAACTAGG CTTTCATCAG -CTTTTTTGT TTTGTTTTGT TTTGTTTTCA
              GGGACAAAAC ATTTAAACAA ACATGTTTTT CTTT
MAST_A224_    CACTTGTTCT ATATTTTGTA TTACCTGTAG TGGATA---- ---GCAGAGC
              AAAAACTAGG CTTTCATCAG TCTTTTTTGT TTTGTTTTGT TTTGTTTTCA
              GGGACAAAAC ATTTAAACAA ACATGTTTTT CTTT
SAV1_SE210    CACTTGTTCT ATATTTTGTA TTACCTGTAG TGGATAAAAA ATAGCAGAGC
              AAAAACTAGG CTTTCATCAG -CTTTTTTGT TTTGTTTTGT TTT-----CA
              GGGACAAAAC ATTTAAACAA ACATGTTTTT CTTT

 1 1 1 1 1
5 96

ASIAN2_Ema    GAACTAAGCT GAGTTCCATC AATACACCAG GCTATTTCAG GCCTCCCTAA
              CTTTGCTCAA GCTGCTCCCC AGGGCTGCAA TCCCCTTCTT ATCTCA
FOR_DS1535    GAACTAAGCT GAGTTCCATC AATACACCAG GCTATTTCAG GCCTCCCTAA
              CTTTGCTCAA GCTGCTCCCC AGGGCTGCAA TCCCCTTCTT ATCTCA
MAM1_SP134    GAACTAAGCT GAGTTCCATC AATACACCAG GCTATTTCAG GCCTCCCTAA
              CTTTGCTCAA GCTGCTCCCC AGGGCTGCAA TCCCCTTCTT ATCTCA
MAST_A357_    GAACTAAGCT GAGTTCCATC AATACACCAG GTTATTTCAG GCTTCACTAA
              CTTTGCTCAA GCTGCTCCCC AGGGCTGCAA TCCCCTTCTT ATCTCA
SAV1_SE210    GAACTAAGCT GAGTTCCATC AATACACCAG GCTATTTCAG GCCTCCCTAA
              CTTTGCTCAA GCTGCTCCCC AGGGCTGCAA TCCCCTTCTT ATCTCA

 1 1 1 1 1
5 107

ASIAN2_Ema    ACTGTTGCCG GCACCTGGGG AGGGGGCTCA GCTGGAGGAG TCAGTTCTTG
              CAGCCTGCTT CCCCTCCACC CTACTACGCC TTCCACAGGA ATTGGGGTCG
              CCTGCTG
FOR_DS1535    ACTGTTGCCG GCACCTGGGG AGGGGGCTCA GCTGGAGGAG TCAGTTCTTG
              CAGCCTGCTT CCCCTCCGCC CTACTACGCC TTCCACAGGA ATTGGGGTCG
              CCTGCTG
MAM1_SP134    ACTGTTGCCG GCACCTGGGG AGGGGGCTCA GCTGGAGGAG TCAGTTCTTG
              CAGCCTGCTT CCCCTCCGCC CTACTACGCC TTCCACAGGA ATTGGGGTCG
              CCTGCTG
MAST_A225_    ACTGTTGCCG GCACCTGGGG AGGGGGCTCA GCTGGAGGAG TCAGTTCTTC
              CAGCCTGCTT CCCCTCCGCC CTACTACGCC TTCCACAAGA ATTGGGATCG
              CCTACTA
SAV1_SE210    ACTGTTGCCG GCACCTGGGG AGGGGGCTCA GCTGGAGGAG TCAGTTCTTG
              CAGCCTGCTT CCCCTCCGCC CTACTACGCC TTCCACAGGA ATTGGGGTCG
              CCTGCTG

 1 1 1 1 1
5 101

ASIAN2_Ema    CACTCAGAAC ACGACTGYGG AAGAGCTGCC TCCTTAATGA CGCCCTTACT
              GAGGCAGACG GATTAAAGCC TTCGGGACCT TCATTTGCTG ATGTGCCACG
              A
```

```
FOR_DS1535    CACTCAGAAC ACGACTGCRG AAGAGCTGCC TCCTTAATGA CGCCCTTACT
              GAGGCAGACG GATTAAAGCC TTCGGGACCT TCATTTGCTG ATGTGCCACG
              A
MAM1_SP134    CACTCAGAAC ATGACTGCGG AAGAGCTGCC TCCTTAATGA CGCCCTTACT
              GAGGCAGACG GATTAAAGCC TTCGGGACCT TCATTTAATG ATGTGCCACG
              A
MAST_A418_    TACTCAGAAC ACGACTGGGG AAGAGCTGCC TCCTTAATGA CGCCCTTACT
              GAGGCAGACG GATTAAAGCC TTCGGGACCT TCATTTGCTG ATGTGCCATG
              A
SAV1_SE210    CACTCAGAAC ACGACTGCGG AAGAGCTGCC TCCTTAATGA CGCCCTTACT
              GAGGCAGACG GATTAAAGCC TTCGGGACCT TCATTTGCTG ATGTGCCACG
              A

 1 1 1 1 1
5 96

ASIAN2_Ema    AGTAGAGAGA AACAGGGAAA GACTTTCCAG GTGACAGGAA CCGGAGAAGC
              AAGGTACAAG GGATGAGGCT GAATGCAACA TACCAGCTCT ATCCAG
FOR_DS1535    AGTAGAGAGA AACAGGGAAA GACTTTCCAG GTGACAGGAA CCGGAGAAGC
              AAGGTACAAG GGATGAGGCT GAATGCAACA TACCGGCTCT ATCCAG
MAM1_SP134    AGTAGAGAGA AACAGGGAAA GACTTTCCAG GTGACAGGAA CCGGAGAAGC
              AAGGTACAAG GGATGAGGCT GAATGCAACA TACCAGCTCT ATCCAG
MAST_A227_    AGCAGAGAGA AACAGGGAAA GACTTTCCAG GTGACAGGAA CCGGAGAAGC
              AAGGTACAAG GGATGAGGCT GAATGCAACA TACCGGCTCT ATCCAG
SAV1_SE210    AGTAGAGAGA AACAGGGAAA GACTTTCCAG GTGACAGGAA CCGGAGAAGC
              AAGGTACAAG GGATGAGGCT GAATGCAACA TACCGGCTCT ATCCAG

 1 1 1 1 1
5 99

ASIAN2_Ema    TGTGGTACTG TGCCAAGCCT TCCACATATT ACTACGTAGC TACTCCAATT
              ATCCATATTT GACAGATGGG CAGTCTGAGG CCTTCAGGAT TTAAAAACT
FOR_DS1535    TGTGGTACTG TGCCAAGCCT TCCACATATT ACTACGTAGC TACTCCAATT
              ATCCATATTT GACAGATGGG CAGTCTGAGG CCTTCAGRAT TTAAAAACT
MAM1_SP134    TGTGGTACTG TGCCAAGCCT TCCACATATT ACTACGTAGC TACTCCAATT
              ATCCATATTT GACAGATGGG CAGTCTGAGG CCTTCAGRAT TTAAAAACT
MAST_A229_    TGTGGTACTG TGCCAAGCCT TCCACATATT ACTACATAGC TACTCCAATT
              ATCCATATTT GACAGATGGG CAGTCTGAGG CCTTCAGAAG TTAAAAACT
SAV1_SE210    TGTGGTACTG TGCCAAGCCT TCCACATATT ACTACGTAGC TACTCCAATT
              ATCCATATTT GACAGATGGG CAGTCTGAGG CCTTCAGAAT TTAAAAACT

 1 1 1 1 1
5 104

ASIAN2_Ema    CCCCGACCCC TCCGTTGAAA TTCACCTGTC -AGG-CTGCT GAAGCCCTTG
              CCCTTGGCGT CCTCAACTCT ACTGTCTGAC CGACTGCCTG AGCAGCAGGT
              GCTT
FOR_DS1535    CCCCGACCCC TCCGTTGAAA TTCACCTGTC -AGG-CTGCT GAAGCCCTTG
              CCCTTGGCGT CCTCAACTCT ACTGTCTGAC CGACTGCCTG AGCAGCAGGT
              GCTT
MAM1_SP134    CCCCGACCCC TCCGTTGAAA TTCACCTGTC -AGG-CTGCT GAAGCCCTTG
              CCCTTGGCGT CCTCAACTCT ACTGTCTGAC CGACTGCCTG AGCAGCAGGT
              GCTT
MAST_A299_    CCCCGACCCC TCCGTTGAAA TTCACTTGTT -AGG-CTGCT GAAGCCCTTG
              CCCTTGGCGT CCTCAACTCT ACTGTCTGAC CGACTGCCTG AGCAGCAGGT
              GCTT
SAV1_SE210    CCCCGACCCC TCCGTTGAAA TTCACCTGTC -AGG-CTGCT GAAGCCCTTG
              CCCTTGGCGT CCTCAACTCT ACTGTCTGAC CGACTGCCTG AGCAGCAGGT
              GCTT

 1 1 1 1 1
5 102

ASIAN2_Ema    GTCACTTGCC TCAGAAACAC AGGCGAAGAT TGAACTGGGA CCTTGCCCTG
              CCCCTGACAG CAGAGTGATC ACCCATGGGC AGCCCTCTTA ACCTCCCTGA
              GT
FOR_DS1535    GTCACTTGCC TCAGAAACAC AGGCGAAGAY TGAACTGGGA CCTTGCCCTG
              CCCCTGACAG CAGAGTGATC ACCCATGGGC AGCCCTCTTA ACCTCCCTGA
              GT
MAM1_SP134    GTCACTTGCC TCAGAAACAC AGGCGAAGAT TGAACTGGGA CCTTGCCCTG
              CCCCTGACAG CAGAGTGATC ACCCATGGGC AGCCCTCTTA ACCTCCCTGA
```

```
               GT
MAST_A300_     GTCACTTGCC TTAGAAACAC AGGCGAAGAT TGAAGTGGGA CCTTGCCCTG
               CCCCTGACAA CAGAGTGATC ACCCATAGGC AGCCCTCTTA ACCTCCCTGA
               GT
SAV1_SE210     GTCACTTGCC TCAGAAACAC AGGCGAAGAT TGAACTGGGA CCTTGCCCTG
               CCCCTGACAG CAGAGTGATC ACCCATGGGC AGCCCTCTTA ACCTCCCTGA
               GT

 1 1 1 1 1
5 92

ASIAN2_Ema     TAACTAGGAA ACCTAGAAGA ATAGTTTCTG CTTGTATGAC AGGGATCTTT
               TATTCGTGGA TGACAGAGCC TATTTTGCAG TTTCTATTCA CA
FOR_DS1535     TAACTAGGAA ACCTAGAAGA ATAGTTTCTG CTTGTATGAC AGGGATCTTT
               TATTCGTGGA TGACAGAGCC TATTTTGCAG TTTCTATTCA CA
MAM1_SP134     TAACTAGGAA ACCTAGAAGA ATAGTTTCTG CTTGTATGAC AGGGATCTTT
               TATTCGTGGA TGACAGAGCC TATTTTGCAG TTTCTATTCA CA
MAST_A301_     TAATTAGGAA ATCTAGAAGA ATAGTTTCTG CTTGTATGAC AGGGATCTTT
               TATTCATGAA TGACAGAGCC TATTTTGCAG TTTCTATTCA CA
SAV1_SE210     TAACTAGGAA ACCTAGAAGA ATAGTTTCTG CTTGTATGAC AGGGATCTTT
               TATTCGTGGA TGACAGAGCC TATTTTGCAG TTTCTATTCA CA

 1 1 1 1 1
5 96

ASIAN2_Ema     TATATGCACA TACCGTAGTC TAACCC-AAA AAACCCAGGG CCGCCGAGTT
               GTTTCCTACA GAGTAGAACT GCCCCATAGA GTTTCCAAGG AGTGCC
FOR_DS1535     TATATGCACA TACCGTAGTC TAACCC-AAA AA-CCCAGGG CCGCCGAGTT
               GTTTCCTACA GAGTAGAACT GCCCCATAGA GTTTCCAAGG AGTGCC
MAM1_SP134     TATATGCACA TACCGTAGTC TAACCC-AAA AAACCCAGGG CCGCCGAGTT
               GTTTCCTACA GAGTAGAACT GCCCCATAGA GTTTCCAAGG AGTGCC
MAST_A302_     TATATGCACA TACCGTAGCC TAACCC-AAA AAACCCAGTG CCGCCGAGTT
               GTTTCCTACA GAGTAGAACT GCCTCATAGA ATTTCCAAGG AGTGCC
SAV1_SE210     TATATGCACA TACCGTAGTC TAACCCCAAA AA-CCCAGGG CCGCCGAGTT
               GTTTCCTACA GAGTAGAACT GCCCCATAGA GTTTCCAAGG AGTGCC

 1 1 1 1 1
5 93

ASIAN2_Ema     CRTATCGGTG CAACTTCCTG GGGCTCAGCC CACAGGTGCA GATCCCCTCC
               CATGTACTAT CCTCAGGTAC GTGTGGTCTT GGTCTGGCCT GAT
FOR_DS1535     CGTATCGGTG CAACTTCCTG GGGCTCAGCC CACAGGTGCA GATCCCCTCC
               CATGTACTAT CCTCAGGTAC GTGTGGTCTT GGTMTGGCCT GAT
MAM1_SP134     CGTATCGGTG CAACTTCCTG GGGCTCAGCC CACAGGTGCA GATCCCCTCC
               CATGTACTAT CCTCAGGTAC GTGTGGTCTT GGTCTGGCCT GAT
MAST_A232_     TGTATCGGTG CAACTTCCTG GGGCTCAGCC CACAGGTGCA GATCCCCTCC
               CATGTACTAT CCTCAGGTAC GTGTGGTCTT AGTCTGGCCT GGT
SAV1_SE210     CGTATCGGTG CAACTTCCTG GGGCTCAGCC CACAGGTGCA GATCCCCTCC
               CATGTACTAT CCTCAGGTAC GTGTGGTCTT GGTCTGGCCT GAT

 1 1 1 1 1
5 90

ASIAN2_Ema     GAGTGGTGGG GTTCACAGGT CCCTCTGGCA GCCCAGGTGT GGGGATTGGA
               CTGGAGGGGG -CAGGACTGC AGTCCGGGAG ACCTGTGCTG
FOR_DS1535     GAGTGGTGGG GTTCACAGGT CCCTCTGGCA GCCCAGGTGT GGGGATTGGA
               CTGGAGGGGG -CAGGACTGC AGTCTGGGAG ACCTGTGCTG
MAM1_SP134     GAGTGGTGGG GTTCACAGGT CCCTCTGGCA GCCCAGGTGT GGGGATTGGA
               CTGGAGGGGG -CAGGACTGC AGTCCGGGAG ACCTGTGCTG
MAST_A358_     GAGTGGTGGG GTTCACAGGT CCCTCTGGCA GCCCAGGTG- --GGATTGGA
               CTGGAGGGGG GCAGGACTGC AGTCTGGGAG ACCTGTACTA
SAV1_SE210     GAGTGGTGGG GTTCACAGGT CCCTCTGGCA GCCCAGGTGT GGGGATTGGA
               CTGGAGGAGG -CAGGACTGC AGTCTGGGAG ACCTGTGCTG

 1 1 1 1 1
5 91

ASIAN2_Ema     GCAGCATCCC TGCCGGGCCA AGGGCAGCTC TGCCGAC-AC CACCAAGTTC
               A-TCGCCACG AGCTGCTGTG GTTTAGCACR CTGCAGACAT G
FOR_DS1535     GCAGCATCCC TGCCAGGCCA AGGGCAGCTC TGCCGAC-AC CACCAAGTTC
               A-TCGCCACG AGCTGCTGTG GTTTAGCACG CTGCAGACAT G
```

```
MAM1_SP134   GCAGCATCCC TGCCAGGCCA AGGGCAGCTC TGCCGAC-AC CACCAAGTTC
             A-TCGCCACG AGCTGCTGTG GTTTAGCACG CTGCAGACAT G
MAST_A304_   ACAGCATCCC TGCCAGGCCA AGGGCAGCTC TGCCGACCAC CACCAAGTTC
             AGTCGCCACG AGCTGCTGTG GTTTAGCACG CTGCAAACAC A
SAV1_SE210   GCAGCATCCC TGCCAGGCCA AGGGCAGCTC TGCCGAC-AC CACCAAGTTC
             A-TCGCCACG AGCTGCTGTG GTTTAGCACG CTGCAGACAT G

 1 1 1 1 1
5 93

ASIAN2_Ema   TGATTTTTTA TTTCTCCACC CAAAAACCAA ACTCGTATAC AGGCTCCCCA
             CCCATCCCAA CACTCCGGCA ACCCATTCTT CGGCGTTCCA AGT
FOR_DS1535   TGATTTTTTA TTTCTCCACC CAAAAACCAA ACTCGTATAC AGGCTCCCCA
             CCCATCCCAA CACTCCGGCA ACCCATTCTT CGGCGTTCCA AGT
MAM1_SP134   TGATTTTTTA TTTCTCCACC CAAAAACCAA ACTCGTATAC AGGCTCCCCA
             CCCATCCCAA CACTCCGGCA ACCCATTCTT CGGCGTTCCA AGT
MAST_A305_   TGATTTTTTA TTTCTCCACC CAAAAATCAA ACTCGTATAT AGGCTCCCCA
             CCCATCCCAA CACTCCGGCA ACCCATTCTT CGGCGTTCCA AGT
SAV1_SE210   TGATTTTTTA TTTCTCCACC CAAAAACCAA ACTCGTATAC AGGCTCCCCA
             CCCATCCCAA CACTCCGGCA ACCCATTCTT CGGCGTTCCA CGT

 1 1 1 1 1
5 103

ASIAN2_Ema   GTTAAACACC GGACCAGACC ACAAGTGGG- TGGGAGCCAG ACTGGAACTA
             CACATGTGGA TTGCATAGGG CTCGCTGGTG GTGAGCAACA GAAACTGATT
             AGG
FOR_DS1535   GTTAAACACC GGACCAGACC ACAAGTGGGG TGGGAGCCGG ACTGGAACTA
             CACGTGTGGA TTGCATAGGG CTCGCTGGTG GTGAGCAACA GAAACTGATT
             AGG
MAM1_SP134   GTTAAACACC GGACCAGACC ACAAGTGGG- TGGGAGCCAG ACTGGAACTA
             CATGTGTGGA TTGCATAGGG CTCGCTGGTG GTGAGCAACA GAAACTGATT
             AGG
MAST_A306_   GTTAAACACC GGACCAGACC ATAAGTGGGG TGGGAGCCGG ACTGGAACTA
             CACGTGTGGA TTGCATAGGG CTCACTGGTG GTGAGCAACA GAAACTGATT
             AAA
SAV1_SE210   GTTAAACACC GGACCAGACC ACAAGTGGGG TGGGAGCCAG ACTGGAACTA
             CACGTGTGGA TTGCATAGGG CTCGCTGGTG GTGAGCAACA GAAACTGATT
             AGG

 1 1 1 1 1
5 109

ASIAN2_Ema   CAAATTTGGA TGCCTCTCCA CGGGGATCCT AACTTTTCTT CTATTCGACA
             ATGAGGGATC GGCCAGCAGA GAAGGGTCCT TGTTGAAACC AAGGAAGAAG
             AGCTTCAGT
FOR_DS1535   CAAATTTGGA TGCCTCTCCA CGGGGATCCT AACTTTTCTT GTATTTGACA
             GTGAGGGATC GGCCAGCAGA GAAGGGTCCT TGTTGAAACC AAGGAAGAAG
             AGCTTCAGT
MAM1_SP134   CAAATTTGGA TGCCTCTCCA CGGGGATCCT AACTTTTCTT STATTYGACA
             RTGAGGGATC GGCCAGCAGA GAAGGGTCCT TGTTGAAACC AAGGAAGAAG
             AGCTTCAGT
MAST_A233_   CAAATTTGGA TGCCTCTCCA CGGGGATCCT AACTTTTCTT GTATTCGATG
             GTGAGGGATC GGCCAGCAGA GAAGGGTCCT TGTTGAAACC AAGGAAGAAG
             AGCTTCAGT
SAV1_SE210   CAAATTTGGA TGCCTCTCCA TGGGGATCCT AACTTTTCTT GTATTTGACA
             GTGAGGGATC GGCCAGCAGA GAAGGGTCCT TGTTGAAACC AAGGAAGAAG
             AGCTTCAGT

 1 1 1 1 1
5 94

ASIAN2_Ema   CTGGCAAGTG CTGGTGGGTG TGGGTGTGCC CATCATCCTC CTGCTGATCC
             TGGTGGTGTT CATCCGGCTC CTGCAGTCCC ACTGCCCACG TGTC
FOR_DS1535   CTGGCAAGTG CTGGTGGGTG TGGGTGTGCC CATCATCCTC CTRCTGATCC
             TGGTGGTGTT CATCCGGCTC CTGCAGTCCC ACTGCCCACG TGTC
MAM1_SP134   CTGGCAAGTG CTGGTGGGTG TGGGTGTGCC CATCATCCTC CTGCTGATCC
             TGGTGGTGTT CATCCGGCTC CTGCAGTCCC ACTGCCCACG TGTC
MAST_A234_   CTGGCAAGTG CTGGTGGGTG TGGGTGTGCC CATCATCCTC CTGCTGATCC
             TGGTGGTGTT CATCCGACTC CTGCAGTCCC ACTGCCCACG TATC
SAV1_SE210   CTGGCAAGTG CTGGTGGGTG TGGGTGTGCC CATCATCCTC CTGCTGATCC
```

```
            TGGTGGTGTT CATCCGGCTC CTGCAGTCCC ACTGCCCACG TGTC

 1 1 1 1 1
5 97

ASIAN2_Ema  AAGACAGCTA CCTGGTCAAC CAACTGGAAG AGATCTGTAT TTATGCCTAT
            TCCAAATAAA GGTGATCCAA CAGAATGCAG AAATTATCTA ACAATAT
FOR_DS1535  AAGACAGCTA CCTGGTCAAC CAACTGGAAG AGATCTGTAT TTATGCCTAT
            TCCAAATAAA GGTGATCCAA CAGAATGCAG AAATTATCTA ACAATAT
MAM1_SP134  AAGACAGCTA CCTGGTCAAC CAACTGGAAG AGATCTGTAT TTATGCCTAT
            TCCAAATAAA GGTGATCCAA CAGAATGCAG AAATTATCTA ACAATAT
MAST_A421_  AAGACAGCTA CCTGGTCAAC CAACTGGAAG AGATCTGTAG TTATACCTAT
            TTCAAAGAAA GGTGATCCAG CAGAATGTGG AAATTATCAA ACAATAT
SAV1_SE210  AAGACAGCTA CCTGGTCAAC CAACTGGAAG AGATCTGTAT TTATGCCTAT
            TCCAAATAAA GGTGATCCAA CAGAATGCAG AAATTATCTA ACAATAT

 1 1 1 1 1
5 105

ASIAN2_Ema  TGTCTTTGTG CCACTCTGAA TCCATCTTGA GATCCCCTCT TCTCTCCCTT
            TTAAAGAGAG CTGGCAAACT CACTTCCTGG TAACTCCCTG CTGATTCCAA
            GAATC
FOR_DS1535  TGTCTTTGTG TCACTCTGAA TCCATCTTGA GATCCCCTCT TCTCTCCCTT
            TTAAAAAGAG CTGGCAAACT CACTTCCTGG TAACTCCCTG CTGATTCCAA
            GAATC
MAM1_SP134  TGTCTTTGTG CCACTCTGAA TCCATCTTGA GATCCCCTCT TCTCTCCCTT
            TTAAAGAGAG CTGGCAAACT CACTTCCTGG TAACTCCCTG CTGATTCCAA
            GAATC
MAST_A360_  TGTCTTTGTG CCACTCTGAA TCCATCTTGA GATCCCCTCT TTTCTCCCTT
            TTAAAGAGAG CTGGCAAACT CACTTCCTGG TAACTCCCTG CTGATTCCAA
            GAATC
SAV1_SE210  TGTCTTTGTG CCACTCTGAA TCCATCTTGA GATCCCCTCT TCTCACCCTT
            TTAAAGAGAG CTGGCAAACT CACTTCCTGG TAACTCCCTG CTGATTCCAA
            GAATC

 1 1 1 1 1
5 93

ASIAN2_Ema  TCTTGCCCAG CCCTGCTGGG GACCTGGCTG GGCACAGGGT CCCTGTCGCT
            CAGACCGAGG CTCTCAGCTG ACTTCCTGAA GGCCCAGAAA CTC
FOR_DS1535  TCTTGCCCAG CCCTGCTGGG GACCTGGCTG GGCACAGGGT CCCTGTCGCT
            CAGACCGAGG CTCTCAGCTG ACTTCCTGAA GGCCCAGAAA CTC
MAM1_SP134  TCTTGCCCAG CCCTGCTGGG GACCTGGCTG GGCACAGGGT CCCTGTCGCT
            CAGACCGAGG CTCTCAGCTG ACTTCCTGAA GGCCCAGAAA CTC
MAST_A235_  TCTTGCCCAA CCCTACTGGG GACCTGGTTG AGCACAGGGT CCCTGTCGCT
            CAGACCAAGG CTCTCAGCTG ACTTCCTGAA GGCCCAGAAA CTC
SAV1_SE210  TCTTGCCCAG CTCTGCTGGG GACCTGGCTG GGCACAGGGT CCCTGTCGCT
            CAGACCGAGG CTCTCAGCTG ACTTCCTGAA GGCCCAGAAA CTC

 1 1 1 1 1
5 95

ASIAN2_Ema  TTAGTTGTGT GCCAATTGTA GAACAAACTT TGGAAGCATA ATAGTGGTAT
            TCATAAGACT TTCTGTGCTA TATGTGGGAC CCTCCTGGAA CTGTT
FOR_DS1535  TTAGTTGTGT GCCAATTGTA GAACAAACTT TGGAAGCATA ATAGTGGTAT
            TCATAAGACT TTCTGTGCTA TATGTGGGAC CCTCCTGGAA CTGTT
MAM1_SP134  TTAGTTGTGT GCCAATTGTA GAACAAACTT TGGAAGCATA ATAGTGGTAT
            TCATAAGACT TTCTGTGCTA TATGTGGGAC CCTCCTGGAA CTGTT
MAST_A423_  TTAGTTGTGT GCCAATTTTA GAACAAACTT TGGAAGTGTA ATAGTGGTAT
            TCATAAGACT TTCTGTGCTA TATGTGGGAC CCTCCTGGAA CTGTT
SAV1_SE210  TTAGTTGTGT GCCAATTGTA GAACAAACTT TGGAAGCATA ATAGTGGTAT
            TCATAAGACT TTCTGTGCTA TATGTGGGAC CCTCCTGGAA CTGTT

 1 1 1 1 1
5 93

ASIAN2_Ema  CTGGGTACAC TTGTGTGGAA ATTGCTAATG TTAAGTCTTT AATATTCTGT
            TTCATGTTTT GTTGTTGTTG TTAGGTGCCA TCAAATCAAT TCA
FOR_DS1535  CTGGGTACAC TTGTGTGGAA ATTGCTAATG TTAAGTCTTT AATATTCTGT
            TTCATGTTTT GTTGTTGTTG TTAGGTGCCA TCAAATCAAT TCA
MAM1_SP134  CTGGGTACAC TTGTGTGGAA ATTGCTAAWG TTAAGTCTTT AATATTCTGT
```

TTCATGTTTT GTTGTTGTTG TTAGGTGCCA TCAAATCAAT TCA
MAST_A236_ TTGGGTATAC TTGTGTGGAA ATTGCTAATG TTAAGTCTTT AATATTCTGT
TTCATGTTTT GTTGTTTTTG TTAGGTGCCA TCAAATCAGT TCA
SAV1_SE210 CTGGGTACAC TTGTGTGGAA ATTGCTAATG TTAAGTCTTT AATATTCTGT
TTCATGTTTT GTTGTTGTTG TTAGGTGCCA TCAAATCAAT TCA

1 1 1 1 1
5 108

ASIAN2_Ema ACACACAACC TTAATGATCA CAGAGGACGT CTCACCCCTT TCCTTCACAT
GGGTGCCAGT GAGGGTCACT GGGAAGAGAT ACCCCAGAAA -GGTGTCTTT
TGCGGGTG
FOR_DS1535 ACACACAACC TTAATGATCA CAGAGGACGT CTCACCCCTT TCCTTCACAT
GGGTGCCAGT GAGGGTCACT GGGAAGAGAT ACCCCAGAAA -GGTGTCTTT
TGCGGGTG
MAM1_SP134 ACACACAACC TTAATGATCA CAGAGGACGT YTCACCCCTT TCCTTCACAT
GGGTGCCAGT GAGGGTCACT GGGAAGAGAT ACCCCAGAAA -GGTGTCTTT
TGCGGGTG
MAST_A237_ ACACACAACC TTAATGATCA CAGAGGACGT CTCACCCCTT TCCTTCACAT
GGGTGCCAGT GAGGGTCACT GGGAAGAGAT ATCCCAGAAA AGGTGTCTTT
TGCGGGTA
SAV1_SE210 ACACACAACC TTAATGATCA CAGAGGACGT CTCACCCCTT TCCTTCACAT
GGGTGCCAGT GAGGGTCACT GGGAAGAGAT ACCCCAGAAA -GGTGTCTTT
GGCGGGTG

1 1 1 1 1
5 93

ASIAN2_Ema AATTACTAAT TACAGTTAGG AATGCTAGAA TTAAAAAATT ATGCTACCAA
AAATATTTCC ACATGCTTGC AGTTTGTTTT AATGTGGAGG GTG
FOR_DS1535 AATTACTAAT TACAGTTAGG AATGCTAGAA TTAAAAAATT ATGCTACCAA
AAATATTTCC ACATGCTTGC AGTTTGTTTT AATGTGGAGG GTG
MAM1_SP134 AATTACTAAT TACAGTTAGG AATGCTAGAA TTAAAAAATT ATGCTACCAA
AAATATTTCC ACATGCTTGC AGTTTGTTTT AATGTGGAGG GTG
MAST_A238_ AATTACTAAT TACAGTTAGG AATGCTAGAA TTAAAAAATT ATGCTACCAA
AAATATTTCC ACATGCTTGC AGTTTGTTTT AATGTGGAAG GTA
SAV1_SE210 AATTACTAAT TACAGTTAGG AATGCTAGAA TTAAAAAATT ATGCTACCAA
AAATATTTCC ACATGCTTGC AGTTTGTTTT AATGTGGAGG GTG

1 1 1 1 1
5 111

ASIAN2_Ema TTGAATTGCC CCATAGGGTT TCCAAGGCTG TAATCTTTAT GGAAGCAGAC
TGCCACATCT TTCTCCCTTG GAGTGGTTGG TGGGTTTGAA CTGCCAACTT
TTAGGTTATC A
FOR_DS1535 TTGAATTGCC CCATAGGGTT TCCAAGGCTG TAATCTTTAT GGAAGCAGAC
TTCCACATCT TTCTCCCTTG GAGTGGTTGG TGGGTTTGAA CTGCCAACTT
TTAGGTTATC A
MAM1_SP134 TTGAATTGCC CCATAGGATT TCCAAGGCTG TAATCTTTAT GGAAGCAGAC
TGCCACATCT TTCTCCCTTG GAGTGGTTGG TGGGTTTGAA CTGCCAACTT
TTAGGTTATC A
MAST_A309_ TAGAATTGCC CCCTAGGGTT TCCAAGGCTG TAATCTTTAT GAAAGCAGAT
TGCCACATCT TTCTCCCTTG GAGTGGTTGG TGGATTTGAA CTACCAACCT
TTTGGTTAGC A
SAV1_SE210 TTGAATTGCC CCATAGGGTT TCCAAGGCTG TAATCTTTAT GGAAGCAGAC
TGCCACATCT TTCTCCCTTG GAGTGGTTGG TGGGTTTGAA CTGCCAACTT
TTAGGTTATC A

1 1 1 1 1
5 104

ASIAN2_Ema CTACAAAGAT CTCTTTGTAT GAGACTCCAA CTGGCTGGAA GTTTTTTGGG
AATTTGATGG ATGCAAGCAA ACTGTCCCTT TGTGGGGAGG AAAGCTTCGG
CACT
FOR_DS1535 CTACAAAGAT CTCTTTGTAT GAGACTCCAA CTGGCTGGAA GTTTTTTGGG
AATTTGATGG ATGCAAGCAA ACTGTCCCTT TGTGGGGAGG AAAGCTTCGG
CACT
MAM1_SP134 CTACAAAGAT CTCTTTGTAT GAGACTCCAA CTGGCTGGAA GTTTTTTGGG
AATTTGATGG ATGCAAGCAA ACTGTCCCTT TGTGGGGAGG AAAGCTTCGG
CACT
MAST_A311_ TTACAAAGAT TTCTTTGTAT GAGACTCCAA CTGGCTGGAA GTTTTTTGGG

AATTTGATGG ATGCAAGCAA ACTGTCCCTT TGTGGGGAGG AAAGCTTCGG
CACT
SAV1_SE210 CTACAAAGAT CTCTTTGTAT GAGACTCCAA CTGGCTGGAA GTTTTTTGGG
AATTTGATGG ATGCAAGCAA ACTGTCCCTT TGTGGGGAGG AAAGCTTCGG
CACT

1 1 1 1 1
5 102

ASIAN2_Ema CCATTAAGAG AGACTGAGAA GGAGCAGCCA GTTGGGCAAA ATGAAAACCA
GGTGAAAGTG GTGTCACAGA AGCCAAAGAA ATAAAGGAAA GGTGGTCAAC
TG
FOR_DS1535 CCACTACGAG AGATTGAGAA GGAGCAGCCA GTTGGGCAAA ATGAAAACCA
GGTGAAAGTG GTGTCACAGA AGCCAAAGAA ATAAAGGAAA GGTGGTCAAC
TG
MAM1_SP134 CCATTAAGAG AGACTGAGAA GGAGCAGCCA GTTGGGCAAA ATGAAAACCA
GGTGAAAGTG GTGTCACAGA AGCCAAAGAA ATAAAGGAAA GGTGGTCAAC
TG
MAST_A239_ CCATTAAGTG AGACTGAGAA GGAGCAGCCT GTTGGGCAAA ATGAAAACCA
GGTGAAAGTT GTGTCACAGA AGCCAAAGAA ATAAAGGAAA GGTGATCAAC
TA
SAV1_SE210 CCATTAAGAG AGACTGAGAA GGAGCAGCCA GTTGGGCAAA ATGAAAACCA
GGTGAAAGTG GTGTCACAGA AGCCAAAGAA ATAAAGGAAA GGTGGTCAAC
TG

1 1 1 1 1
5 113

ASIAN2_Ema ACCGGGGCTG ACGAGGTCGC GGGTTGACAG GCCACTGGCT GAGGGGTCGC
CGGCCGAGGG GACCTGGGTT GAGGGTCACG TCCCGAGAAG ATGTCCCAGA
CTGAACAAAC CAG
FOR_DS1535 ACCGGGGCTG ACGAGGTCGY GGGTTGACAG GCCACTGGCT GAGGGGTCGC
CGGCCGAGGG GACCTGGGTT GAGGGTCACG TCCCGAGAAG ATGTCCCAGA
CTGAGCAAAC CAG
MAM1_SP134 ACCGGGGCTG ACGAGGTCGC GGGTTGACAG GCCACTGGCT GAGGGGTCGC
CGGCCGAGGG GACCTGGGTT GAGGGTCACG TCCCGAGAAG ATGTCCCAGA
CTGAGCAAAC CAG
MAST_A312_ ACCGGGGCTG ACGAGGTCGC GGGTTGACAG GCCACTGGCT GAGGGGTCGC
CGGCCGAGGG GACCTGGGTT GAGGGTCACG TCCCGAGAAG ATGTCCCAGA
CTGAGCAAAC CAG
SAV1_SE210 GCCGGGGCTG ACGAGGTCGC GGGTTGACAG GCCACTGGCT GAGGGGTCGC
CGGCCGAGGG GACCTGGGTT GAGGGTCACG TCCCGAGAAG ATGTCCCAGA
CTGAGCAAAC CAG

1 1 1 1 1
5 108

ASIAN2_Ema AAAAGTTCTT CTTAACAGTC AGATCACACA CTATCAGAGC ATTCGTATTT
CTATTAAAAA AAAA-G-CAA TATCTAAAAT GCTGTGCTGG CTAGACATAT
TTTGTAAA
FOR_DS1535 AAAAGTTCTT ---AACAGTC AGATCACACA CTATCAGAGC ATTCGTATTT
CTATTAAAAA AAAAAG-CAA TATCTAAAAT GCTGTGCTGG CTAGACATAT
TTTGTAAA
MAM1_SP134 AAAAGTTCTT CTTAACAGTC AGATCACACA CTATCAGAGC ATTCGTATTT
CTATTAAAAA AAAAAG-CAA TATCTAAAAT GCTGTGCTGG CTAGACATAT
TTTGTAAA
MAST_A241_ AAAAGTTCTT CTTAACAGTC AGATCACACA CTATCAGAGC ATTCGTATTT
CTATTAAAAA AAAAAGACAA CATCTAAAAT GCTGGGCTGG CTAGACTTAT
TTTGTAAA
SAV1_SE210 AAAAGTTCTT CTTAACAGTC AGATCACACA CTATCAGAGC ATTCGTATTT
CTATTAAAAA AAA--G-CAA TATCTAAAAT GCTGTGCTGG CTAGACATAT
TTTGTAAA

1 1 1 1 1
5 102

ASIAN2_Ema CCAAATA-TA AATGCATTTT CCATTGGATT GGCCAAGTAG AACTGGATTA
GCCATCAGCT TCTCAAGGAG CTGAGGGTGA ATCTCATTAA CCAACTTAGA
AA
FOR_DS1535 CCAAATA-TA AATGCATTTT CCATTGGATT GGCCAAGTAG AACTGGATTA
GCCATCAGCT TCTCAAGGAG CTGAGGGTGA ATCTCATTAA CCAACTTAGA

```
              AA
MAM1_SP134    CCAAATAATA AATGCATTTT CCATTGGATT GGCCAAGTAG AACTGGATTA
              GCCATCAGCT TCTCAAGGAG CTGAGGGTGA ATCTCATTAA CCAACTTAGA
              AA
MAST_A313_    CCAAATA-TA AATGCATTTT CCATTGGATT GGCCAAGTAG AATTGGATTA
              GCCATCAGCT TCTCAAGGAG CTGAGGGTGA ATCTCATTAA CCAACTTAGA
              AA
SAV1_SE210    CCAAATA-TA AATGCATTTT CCATTGGATT GGCCAAGTAG AGCTGGATTA
              GCCATCAGCT TCTCAAGGAG CTGAGGGTGA ATCTCATTAA CCAACTTAGA
              AA

 1 1 1 1 1
5 102

ASIAN2_Ema    GCCTGGACAC CTAACAGGGC CCTCAATGCA AGTGCCCCAA CACTCTTGTC
              TTAGAGCATA CTGCTCCTTC CTTCTAACTA CTGCTCCAGA CCTGCTTTCC
              AG
FOR_DS1535    GCCTGGACAC CTAACAGGGC CCTCAATGCA AGTGCCCCAA CACTCTTGTC
              TTAGAGCATA CTGCTCCTTC CTTCTAACTA GTGCTCCAGA CCTGCTTTCC
              AG
MAM1_SP134    GCCTGGACAC CTAACAGGGC CCTCAATGCA AGTGCCCCAA CACTCTTGTC
              TTAGAGCATA CTGCTCCTTC CTTCTAACTA CTGCTCCAGA CCTGCTTTCC
              AG
MAST_A314_    GTCTGGACAC CTAACAGGGC CCTCAATGCA AGTGCCCCAA CACTCTTGTC
              TTAGAGCATA CTGCTCCTTC CCTCTAACTA CTGCTCCAGA CCTGCTTTCC
              AG
SAV1_SE210    GCCTGGACAC CTAACAGGGC CCTCAATGCA AGTGCCCCAA CACTCTTGTC
              TTAGAGCATA CTGCTCCTTC CTTCTAACTA CTGCTCCAGA CCTGCTTTCC
              AG

 1 1 1 1 1
5 107

ASIAN2_Ema    TGTTGACTGG TGTGTAAATT TGGGGTCAAA GCTGAATCAC CTGGGTCAAG
              AGAGGGGCGG CTGAATGTTC TCCTCTACCA CCTCGCCCCA CTTTTCAGCT
              GTGCTTC
FOR_DS1535    TGTTGACTGG TGTGTAAATT TGGGGTCAAA GCTGAATCAC CTGGGTCAAG
              AGAGGGGAGG CTGAATGTTC TCCTCTACCA CCTCGCCCCA CTTTTCAGCT
              GTGCTTC
MAM1_SP134    TGTTGACTGG TGTGTAAATT TGGGGTCAAA GCTGAATCAC CTGGGTCAAG
              AGAGGGGCGG CTGAATGTTC TCCTCTACCA CCTCGCCCCA CTTTTCAGCT
              GTGCTTC
MAST_A243_    TGTTGACTGG TGTGTAAATT TGGGGTCAAA GCTGAATCAC CTGGGTCAAG
              AGAGGGGAGG CTGAATGTTC TCCTCTACCA CCTCGCCCCA CTTTTCAGCT
              GTGCTTC
SAV1_SE210    TGTTGACTGG TGTGTAAATT TGGGGTCAAA GCTGAATCAC CTGGGTCAAG
              AGAGGGGAGG CTGAATGTTC TCCTCTACCA CTTCGCCCCA CTTTTCAGCT
              GTGCTTC

 1 1 1 1 1
5 103

ASIAN2_Ema    CAACAGAAAA TAATTATTTA AACTGTGCAA AGAAGTAATT TGAATTTGAA
              GGCTGTGGAT AGGAAACCAG CCTTGGTAAA GGGAGAGAAG TCAAGAAAAG
              AAC
FOR_DS1535    CAACAGAAAA TAATTATTTA AACTGTGCAA AGAAGTAATT TGAATTTGAA
              GGCTGTGGAT AGGAAACCAG CCTTGGTAAA GGGAGAGAAG TCAAGAAAAG
              AAC
MAM1_SP134    CAACAGAAAA TAATTATTTA AACTGTGCAA AGAAGTAATT TGAATTTGAA
              GGCTGTGGAT AGGAAACCAG CCTTGGTAAA GGGAGAGAAG TCAAGAAAAG
              AAC
MAST_A244_    TAACAGAAAA TAATTATTTA AACTGTGCAA AGAAGTAATT TGAATTTGAA
              GGCTGTGGAT AGGAAACCAG CCTTGGTAAA GGGAGAAAAG TCAAGAAAAG
              AAC
SAV1_SE210    CAACAGAAAA TAATTATTTA AACTGTGCAA AGAAGTAATT TGAATTTGAA
              GGCTGTGGAT AGGAAACCAG CCTTGGTAAA GGGAGAGAAG TCAAGAAAAG
              AAC

 1 1 1 1 1
5 118
```

```
ASIAN2_Ema   GTGAGAAGAG ACCCTGGCCC GGTGGGAGGC GGAGAAGGGA GGGAGAAAGG
             AGCACGGGAG AGGGAGGTGA GAGGAGAGGG AGTTAGTGGA CCAACCTACA
             TGAGCTTTCT TCTTCCTC
FOR_DS1535   GTGAGAAGAG ACCCTGGCCC GGTGGGAGGC GGAGAAGGGA GGGAGAAAGG
             AGCATGGGAG AGGGAGGTGA GAGGAGAGGG AGTTAGTGGA CCAACCTACA
             TGAGCTTTCT TCTTCCTC
MAM1_SP134   GTGAGAAGAG ACCCTGGCCT GGTGGGAGGC GGAGAAGGGA GGGAGAAAGG
             AGCATGGGAG AGGGAGGTGA GAGGAGAGGG AGTTAGTGGA CCAACCTACA
             TGAGCTTTCT TCTTCCTC
MAST_A315_   GTGAGAAGAG ACCCTGGCCC GGTGGGAGGC GGAGGAGGGA GGGAGAAAGG
             AGCATGGGAG AGGGAGGTGA GAGGAGAGGG AGTTAGTGGA CCAACCTACA
             TAAGCTTTCT TCTTCCTT
SAV1_SE210   GTGAGAAGAG ACCCTGGCCC GGTGGGAGGC GGAGAAGGGA GGGAGAAAGG
             AGCATGGGAG AGGGAGGTGA GAGGAGAGGG AGTTAGTGGA CCAACCTACA
             TGAGCTTTCT TCTTCCTC

 1 1 1 1 1
5 90

ASIAN2_Ema   TTGGAAAACA GAGAAGCCAG ATTCGCTCAG CCTCGCCCTG CTCTGTCCTC
             TTACCTCTTC TCTGCTAACA TCCATGTTCC ACACAGCAAT
FOR_DS1535   TTGGAAAACA GAGAAGCCAG ATTCGCTCAG CCTCGCCCTG CTCTGTCCTC
             TTACCTCTTC TCTGCTAACA TCCATGTTCC ACACAGCAAT
MAM1_SP134   TTGGAAAACA GAGAAGCCAG ATTCGCTCAG CCTCGCCCTG CTCTGTCCTC
             TTACCTCTTC TCTGCTAACA TCCATGTTCC ACACAGCAAT
MAST_A317_   TTGGAAAACA GAGGAGCCAG ATTCGCTCAG CCTCGCCCTG CTGTGTGCTC
             TTAC-TCTTC TCTGCTAACA TCCATGTTCC ACACAGCAAT
SAV1_SE210   TTGGAAAACA GAGAAGCCAG ATTCGCTCAG CCTCGCCCTG CTCTGTCCTC
             TTACCTCTTC TCTGCTAACA TCCATGTTCC ACACAGCAAT

 1 1 1 1 1
5 106

ASIAN2_Ema   TACTAGGTGC CAAACACCCT CAGAATTGGG GCCTGTGGAA CTCAACAGAA
             GCATCCTGAA AGGCTGGGGA GTCTGTGGAC TTACACAACC TCAGAGGGCT
             TCCAAA
FOR_DS1535   TACTAGGTGC CAAACACCCT CAGAATTGGG GCCTGTGGAA CTCAACAGAA
             GCATCCTGAA AGGCTGGGGA GTCTGTGGAC TTACACAACC TCAGAGGGCT
             TCCAAA
MAM1_SP134   TACTAGGTGC CAAACACCCT CAGAATTGGG GCCTGTGGAA CTCAACAGAA
             GCATCCTGAA AGGCTGGGGA GTCTGTGGAC TTACACAACC TCAGAGGGCT
             TCCAAA
MAST_A364_   TACTAGGTGC CAAACACCCT TAGAACTGGG GGCTGTGGAA CTCAACAAAA
             GCATCCTGAA AGGCTGGGGA GTCTGTGGAC TTACACAACC TCAGAGGGTT
             TCCAAA
SAV1_SE210   TACTAGGTGC CAAACACCCT CAGAATTGGG GCCTGTGGAA CTCAACAGAA
             GCATCCTGAA AGGCTGGGGA GTCTGTGGAC TTACACAACC TCAGAGGGCT
             TCCAAA

 1 1 1 1 1
5 106

ASIAN2_Ema   CAGAAGGTGA CAGGGGGTTC AAGTCCCACT TCAAAGCTGC TCACGGGAGG
             GGCCCCTGCC CTGGGGGGCC GCCTCTGAGG TGACCAACCT TCCTGGTTTG
             CCAGGC
FOR_DS1535   CAGAAGGTGA CAGTGGGTTC AAGTCCCACT TCAAAGCTGC TCACGGGAGG
             GGCCCCTGCC CTGGGGGACC GCCTCTGAGG TGACCAACCT TCCTGGTTTG
             CCAGGC
MAM1_SP134   CAGAAGGTGA CAGGGGGTTC AAGTCCCACT TCAAAGCTGC TCACGGGAGG
             GGCCCCTGCC CTGGGGGGCC GCCTCTGAGG TGACCAACCT TCCTGGTTTG
             CCAGGC
MAST_A245_   CAGAAGATGA CAGGAGGTTC AAGTCCCACT TCAAAGCTGC TCACGGGAGG
             GGCCCCTGCC CTGGGGTGCC GCCTCTGAGA TGACCAACCT TCCTGGTTTG
             CCAGGA
SAV1_SE210   CAGAAGGTGA CAGTGGGTTC AAGTCCCACT TCAAAGCTGC TCACGGGAGG
             GGCCCCTGCC CTGGGGGACC GCCTCTGAGG TGACCAACCT TCCTGGTTTG
             CCAGGC

 1 1 1 1 1
5 119
```

```
ASIAN2_Ema    CATGTGTGTC AGAGTAAAAC TGTCATAGGG TTTT-CAATG GCTAATTTTT
              T-GGAAGTAG ATTACAARAC GTTTCTTTCA AAACGTCTCT AGGTGGACTC
              AAACTTCCAA ACTTTTGGT
FOR_DS1535    CATGTGTGTC AGAGTAAAAC TGTCATAGGG TTTT-CAATG GCTAATTTTT
              T-GGAAGTAG ATTACAAGAC GTTTCTTTCA AAACGTCTCT AGGTGGACTC
              AAACTTCCAA ACTTTTGGT
MAM1_SP134    CATGTGTGTC AGAGTAAAAC TGTCATAGGG TTTT-CAATG GCTAATTTTT
              T-GGAAGTAG ATTACAAGAC GTTTCTTTCA AAACGTCTCT AGGTGGACTC
              AAACTTCCAA ACTTTTGGT
MAST_A246_    CATGTGTGTC AGAGTAAAAC TGTCATAGGG TTTTTCAACG GCTAATTTTT
              TTGGAAGTAG ATCACAAGAC CTTTCTTTCA AAACGTCTCT AGGTGGACTC
              AAACTTCCAA ACTTTTGGT
SAV1_SE210    CATGTGTGTC AGAGTAAAAC TGTCATAGGG TTTT-CAATG GCTAATTTTT
              T-GGAAGTAG ATTACAAGAC GTTTCTTTCA AAACGTCTCT AGGTGGACTC
              AAACTTCCAA ACTTTTGGT

 1 1 1 1 1
5 93

ASIAN2_Ema    GTCTGGCAGA ACTGTGCATT CCATGCCTGG GTGACCTGGT TCAATGGGGG
              AAACCGACCG CTGTCCCCTG AACGCGTTCC AAAACAAGCA TTC
FOR_DS1535    GTCTGGCAGA ACTGTGCATT CCATGCCTGG GTGACCTGGT TCAATGGGGG
              AAACCGACCG CTGTCCCCTG AACGCGTTCC AAAACAAGCA TTC
MAM1_SP134    GTCTGGCAGA ACTGTGCATT CCATGCCTGG GTGACCTGGT TCAATGGGGG
              AAACCGACCG CTGTCCCCTG AACGCGTTCC AAAACAAGCA TTC
MAST_A247_    -TCTGGCAGA ACTGTGCATT CCATGCCTGG GTGACCTAGT TCAATGGGG-
              AAACCGACCG CTGTCCCCTG AACGCGTTCC AAAACAAGCA TTC
SAV1_SE210    GTCTGGCAGA ACTGTGCATT CCATGCCTGG GTGACCTGGT TCAATGGGGG
              AAACCGACCG CTGTCCCCTG AACGCGTTCC AAAACAAGCA TTC

 1 1 1 1 1
5 117

ASIAN2_Ema    AAAGAACACA AAAGCTTGTT GGGTGTCGTG AAAAGCAAGA AGCCCATTTT
              TT-CGTAGTA CTTGGAGATG TTGGGAAAA- GATGCTTTCT TTTAAAGGCC
              GTCCTGAGAC ATCAGGT
FOR_DS1535    AAAGAACACA AAAGCTTGTT GGGTGTCGTG AAAAGCAASA AGGCCATTTT
              TT-CGTAGTA CTTGGAGATG TTGGGAAAA- GATGCTTTCT TTTAAAGGCC
              GTCCTGAGAC ATCAGGT
MAM1_SP134    AAAGAACACA AAAGCTTGTT GGGTGTCGTG AAAAGCAAGA AGCCCATTTT
              TT-CGTAGTA CTTGGAGATG TTGGGAAAA- GATGCTTTCT TTTAAAGGCC
              GTCCTGAGAC ATCAGGT
MAST_A366_    AAACAACA-A AAAGCTTGTT GGGTGTCGTG AAAAGCAAGA AGCCCTATTT
              TTTCGTAGTA CTTGGAGACG CTGGGAAAAA GATGCGTTGT TTTCAAGGCC
              GTCCTGAGAC ATCAGGT
SAV1_SE210    AAAGAACACA AAAGCTTGTT GGGTGTCGTG AAAAGCAAGA AGCCCATTTT
              TT-CGTAGTA CTTGGAGACG TTGGGAAAA- GATGCTTTCT TTTAAAGGCC
              GTCCTGAGAC ATCAGGT

 1 1 1 1 1
5 95

ASIAN2_Ema    TCGCTTACAT CTGGGGGAGA AATGTTCACC TTCACTCGAG CCTTGCACCA
              GGGAAAGAAT TGGGGCTGGG GGTGGTAGCT GAGCCACAGA GTGTG
FOR_DS1535    TCGCTTACAT CTGGGGGAGA AATGTTCACC TTCACTCGAG CCTTGCACCA
              GGGAAAGAAT TGGAGCTGGG GGTGGTAGCT GAGCCACAGA GTGTG
MAM1_SP134    TCGCTTACAT CTGGGGGAGA AATGTTCACC TTCACTCGAG CCTTGCACCA
              GGGAAAGAAT TGGGGCTGGG GGTGGTAGCT GAGCCACAGA GTGTG
MAST_A320_    TTGCTTACAT CTGGGGGAGA AATGTTCACC TTCACTCGAG CCTTGCACCA
              GGGAAAGAAT TGGGGCTGGG GGTGGTAGCT GAGCCACAGA GTATG
SAV1_SE210    TCGCTTACAT CTGGGGGAGA AATGTTCACC TTCACTCGAG CCTTGCACCA
              GGGAAAGAAT TGGGGCTGGG GGTGGTAGCT GAGCCACAGA GTGTG

 1 1 1 1 1
5 97

ASIAN2_Ema    GGCCTGAAGG CAAGGAGGCG CAGCTGGGAG CGGTCACGGA GCTGCTCAGA
              GAGTTGGCAG AGGTCAGGCC CACTGCTGCC CGCCTCCCAG GAT-GGG
FOR_DS1535    GGCCTGAAGG CAAGGAGGCG CAGCTGGGAG CGGTCACGGA GCTGCTCAGA
              GAGTTGGCAG AGGTCAGGCC CACTGCTGCC CGCCTCCCAG SAT-GGG
MAM1_SP134    GGCCTGAAGG CAAGGAGGCG CAGCTGGGAG CGGTCACGGA GCTGCTCAGA
```

```
             GAGTTGGCAG AGGTCAGGCC CACTGCTGCC CGCCTCCCAG GAT-GGG
MAST_A321_   GGTCTGAAGG CAAGGAGGCG CAGCTGGGAG CGGTCACGGA GCTGCTCAGA
             GAGTTGGCAG AGGTCAGGCC CACTGCTGCC CGCCTCCTGG GATTGGG
SAV1_SE210   GGCCTGAAGG CAAGGAGGCG CAGCTGGGAG AGGTCACGGA GCTGCTCAGA
             GAGTTGGCAG AGGTCAGGCC CACTGCTGCC CGCCTCCCAG GAT-GGG

 1 1 1 1 1
5 107

ASIAN2_Ema   AAGAGTAAAG CTAGAGAATT AATTCTAATA AATACTTGTG TCTCAAAGTA
             AAA-GCCTGA AATGGGCGTC AGGAAACGTT CATGTGCAAG GTGAAGATCA
             ATAACTG
FOR_DS1535   AAGAGTAAAG CTAGAGAATT AATTCTAATA AATACTTGTG TCTCAAAGTA
             AAA-GCCTGA AATGGGCGTC AGGAAACGTT CATGTGCAAG GTGAAGATCA
             ATAACTG
MAM1_SP134   AAGAGTAAAG CTAGAGAATT AATTCTAATA AATACTTGTG TCTCAAAGTA
             AAA-GCCTGA AATGGGCGTC AGGAAACGTT CATGTGCAAG GTGAAGATCA
             ATAACTG
MAST_A249_   AAGAGTAAAG CTAGAGAATT AATTCTAATA AATACTTGTG TCTCAAAGTA
             AAAAGCCTGA AATGGGCGTC AGGAAACGTT CATGTGCGAG GTGAAGATCA
             ATAACTG
SAV1_SE210   AAGAGTAAAG CTAGAGAATT AATTCTAATA AATACTTGTG YCTCAAAGTA
             AAA-GCCTGA AATGGGCGTC AGGAAACGTT CATGTGCAAG GTGAAGATCA
             ATAACTG

 1 1 1 1 1
5 109

ASIAN2_Ema   TTCACTATTC TTTTGGTTTT CCTTTTCTTC TCTCCTGTGT CTCTTCCACA
             GATATTGCTG CCTCTCTTTG GCATCAGTTC GGGTCTTGAA AATGTGGCCA
             CCTGTGAAT
FOR_DS1535   TTCACTATYC TTTTGGTTTT CCTTTTCTTC TCTCCTGTGT CTCTTCCACA
             GATATTGCTG CCTCTCTTTG GCATCAGTTC GGGTCTTGAA AATGTGGCCA
             CCTGTGAAT
MAM1_SP134   TTCACTATTC TTTTGGTTTT CCTTTTCTTC TCTCCTGTGT CTCTTCCACA
             GATATTGCTG CCTCTCTTTG GCATCAGTTC GGGTCTTGAA AATGTGGCCG
             CCTGTGAAT
MAST_A368_   TTCACTATTC TTTTGGTTTT CCTTTTCTTC TCTCCTGTGT CTCTTCCACA
             GATATTGCTG CCTTTCTTTG GCATCAATTC GGGTCTTGAA AATGTGGCCA
             CCTGTAAAT
SAV1_SE210   TTCACTATTC TTTTGGTTTT CCTTTTCTTC TCTCCTGTGT CTCTTCCACA
             GATATTGCTG CCTCTCTTTG GCATCAGTTC GGGTCTTGAA AATGTGGCCA
             CCTGTGAAT

 1 1 1 1 1
5 97

ASIAN2_Ema   CTCAGAGTGG GGTGAGGAAT TCCATTTTGA ATGCTTCCAT TTATCTAGCT
             GTGATTTTCA GCTCTCTGAG TGTGAGGGGA CCCTTGAGAC TACCACA
FOR_DS1535   CTCAGA-TGG GGTGAGGAAT TCCATTTTGA ATGCTTCCAT TTATCTAGCT
             GTGATTTTCA GCGCTCTGAG TGTGAGGGGA CCCTTGAGAC TACCACA
MAM1_SP134   CTCAGAGTGG GGTGAGGAAT TCCATTTTGA ATGCTTCCAT TTATCTAGCT
             GTGATTTTCA GCGCTCTGAG TGTGAGGGGA CCCTTGAGAC TACCACA
MAST_A324_   CTCAGAGTGG GGTGAGGAAT TCCATTTTGA ATGCCTCCAT TTATCTAGCT
             GTGATTTTCA GCGCTCTGAG TGTGAGGGGA CCCTTGAGAC TACCACA
SAV1_SE210   CTCAGAGTGG GGTGAGGAAT TCCATTTTGA ATGCTTCCAT TTATCTAGCT
             GTGATTTTCA GCGCTCTGAG TGTGAGGGGA CCCTTGAGAC TACCACA

 1 1 1 1 1
5 103

ASIAN2_Ema   TTTCAATTCT ATTTTAAAAT ACACATGTGG AAGAGGCTCC WTACTGGGAT
             TCATGGAAGT TGCATGAGTG CGGTGATTTG GGAACCTAGA GGTGATAGGT
             GCA
FOR_DS1535   TTTCAATTCT ATTTTAAAAT ACACATGTGG AAGAGGCTCC TTACTGGGAT
             TCATGGAAGT TGCATGAGTG CGGTGATTTG GGAACCTAGA GGTGATAGGT
             GCA
MAM1_SP134   TTTCAATTCT ATTTTAAAAT ACACATGTGG AAGAGGCTCC TTACTGGGAT
             TCATGGAAGT TGCATGAGTG CGGTGATTTG GGAATCTAGA GGTGATAGGT
             GCA
MAST_A326_   TTTTAATTTT ATTTTAAAAT ACACATGTGG AAGAGGTTCC TTACTGGGAT
```

```
            TCATGGAAAT TGCATGAGTG TGGTGATTTG GGAGCCTAGA GGTGATAGGT
            GCA
SAV1_SE210  TTTCAATTCT ATTTTAAAAT ACACATGTGG AAGAGGCTCC TTACTGGGAT
            TCATGGAAGT TGCATGAGTG CGGTGATTTG GGAACCTAGA GGTGATAGGT
            GCA

 1 1 1 1 1
5 112

ASIAN2_Ema  CAAATTGTGT TTCATCGGGT TTCCAATGAC TGATTTTTCA GAAGTGGGTT
            TTCAGGCCTT TCTTCTGAGG CATCTCTGGG TGGACTCGAA CCACCAGCCT
            TTAGGTGAGC GG
FOR_DS1535  CAAATTGTGT TTCATCGGGT TTTCAATGAC TGATTTTTCA GAAGTGGGTT
            TTCAGGCCTT TCTTCTGAGG CATCTCTGGG TGGACTCGAA CCACCAGCCT
            TTAGGTGAGC GG
MAM1_SP134  CAAATTGTGT TTCATCGGGT TTTCAATGAC TGATTTTTCA GAAGTGGGTT
            TTCAGGCCTT TCTTCTGAGG CATCTCTGGG TGGACTCGAA CCACCAGCCT
            TTAGGTGAGC GG
MAST_A251_  CAAATTGTGT TTCATCGGGT TTTCAATGAC TGATTTTTCA GAAGTGGGTT
            TTCAGGCCTT TCTTCTGAGG CATCTCTGGG TGGACTCGAA CCACCAGCCT
            TTAGGTGAGT GG
SAV1_SE210  CAAATTGTGT TTCATCGGGT TTTCAATGAC TGATTTTTCA GAAGTGGGTT
            TTCAGGCCTT TCTTCTGAGG CATCTCTGGG TGKACTCGAA CCACCAGCCT
            TTAGGTGAGC GG

 1 1 1 1 1
5 105

ASIAN2_Ema  CCGCCAAGAC CTGGATT-AA AAACTCAATC CCTCAGTCAC TATCACCAC-
            TGCCCCTGCC TCTCTGCACC CAGAGCTGGC GGCTGGGCAT TGGCGGCCCC
            AGGCC
FOR_DS1535  CCGCCAAGAC CTGGATT-AA AAACTCAATC CCTCAGTCAC TATCACCAC-
            TGCCCCTGCC TCTCTGCACC CAGAGCTGGC GGCTGGGCAT TGGCGGCCCC
            AGGCC
MAM1_SP134  CCGCCAAGAC CTGGATT-AA AAACTCAATC CCTCAGTCAC TAGCACCAC-
            TGCCCCTGCC TCTCTGCACC CAGAGCTGGC AGCTGGGCAT TGGCGGCCCC
            AGGCC
MAST_A252_  CCGCCAAGAC CTGGATTTAA AAACTCAATC CCTCAGTCAC TACCACCACC
            TGCCCCTGCC TCTCTGCACC CAGAGCTGGC GGCTGGGCAT TGGTGGCCCG
            AGACC
SAV1_SE210  CCGCCAAGAC CTGGATT-AA AAACTCAATC CCTCAGTCAC TATCACCAC-
            TGCCCCTGCC TCTCTGCACC CAGAGCTGGC GGCTGGGCAT TGGCGGCCCC
            AGGCC

 1 1 1 1 1
5 102

ASIAN2_Ema  AAAACGGAGG TTCGGCAGCC CAGGGTCAAC TGTCTCTAGA GCAGGGATGG
            GCCTAGTGGG GTAGAATTGT GTAAGAGCGT TAGACGGGGC CTGGGAGAGG
            AA
FOR_DS1535  AAAACGGAGG TTCGGCAGCC CAGGGTCAAC TGTCTCTAGA GCAGGGATGG
            GCCTAGTGGG GTAGAATTGT GTAAGAGCGT TAGACGGGGC CTGGGAGAGG
            AA
MAM1_SP134  AAAACGGAGG TTCGGCAGCC CAGGGTCAAC TGTCTCTAGA GCAGGGATGG
            GCCTAGTGGG GTAGAATTGT GTAAGAGCGT TAGACGGGGC CTGGGAGAGG
            AA
MAST_A328_  AAAACGGAGG TTCGGCAGCC CAGGGTCAAC CGTCTCTAGA GCAGGGATGG
            GCCTAGTGGG GTAGAATTGC ATAAGAGCAT TAGGCGGGGC CTGGGAGAGG
            AA
SAV1_SE210  AAAACGGAGG TTCGGCAGCC CAGGGTCAAC TGTCTCTAGA GCAGGGATGG
            GCCTAGTGGG GTAGAATTGT GTAAGAGCGT TAGACGGGGC CTGGGAGAGG
            AA

 1 1 1 1 1
5 109

ASIAN2_Ema  TGTT-ATTT- ATGCTGAGCA TTTCTGGGGG CCAGACAATT AACTTTTT-A
            CTGCAGGAAT GAAAGCAAGA ATCCTGCAGG CGGCTCCTTC CTTGAGGGGA
            TCCCTCCTA
FOR_DS1535  TGTT-ATTT- ATGCTGAGCA TTTCTGGGGG CCAGACAATT AACTTTTT-A
            CTGCAGGAAT GAAAGCAAGA ATCCTGCAGG CGGCTCCTTC CTTGAGGGGA
```

```
             TCCCTCCTA
MAM1_SP134   TGTT-ATTT- ATGCTGAGCA TTTCTGGGGG CCAGACAATT AACTTTTT-A
             CTGCAGGAAT GAAAGCAAGA ATCCTGCAGG CGGCTCCTTC CTTGAGGGGA
             TCCCTCCTA
MAST_A254_   TGTTTATTTT ATGCTGAGCA TTTCTGGGGG CCAGACAATT AACTTTTTTA
             CTGCAGGAAT GAAAGCAAGA ATCCTGCAGG CGGCTCCTTC CTTAAAGTAA
             TCCCTCCTA
SAV1_SE210   TGTT-ATTT- ATGCTGAGCA TTTCTGGGGG CCAGACAATT AACTTTTT-A
             CTGCAGGAAT GAAAGCAAGA ATCCTGCAGG CGGCTCCTTC CTTGAGGGGA
             TCCCTCCTA

 1 1 1 1 1
5 91

ASIAN2_Ema   GAAACACACC AAAAGACATC TGAAACACAG ACCAGTGGTG CAAGGCCATA
             GTTCTTGTGT CTTCAGAAAA TCCAGAAGGG CCCAAAAAA- G
FOR_DS1535   GAAACACACC AAAAGACGTC TGAATCACAG ACCAATGGTG CAAGGCCATA
             GTTCTTGTGT CTTCAGAAAA TCCAGAAGGG CCCAAAAAA- G
MAM1_SP134   GAAACACACC AAAAGACGTC TGAATCACAG ACCAGTGGTG CAAGGCCATA
             GTTCTTGTGT CTTCAGAAAA TCCAGAAGGG CCCAAAAAA- G
MAST_A330_   GAAATACACC AAAAGACGTC TGAATCACAG ACCAGTGGTG CAAGGCCATA
             GTTCTTGTGT CTTCAGAAAA TCCAGAAGGG CCCAAAAAAA G
SAV1_SE210   GAAACACACC AAAAGACGTC TGAATCACAG ACCAGTGGTG CAAGGCCATA
             GTTCTTGTGT CTTCAGAAAA TCCAGAAGGG CCCAAAAA-- G

 1 1 1 1 1
5 106

ASIAN2_Ema   ACAAAGAGGT GGGCAGTTTG AACCCATCCA GTGGGTCCAT GGAGAAAGAC
             CTGGCGATTT GCTCCCGTAA AGATTACAGC CTAGAAAACC CTATGGAATA
             GTTGTA
FOR_DS1535   ACAAAGAGGT GGGCAGTTTG AACCCATCCA GTGGGTCCAT GGAGAAAGAC
             CTGGCGATTT GCTCCCGTAA AGATTACAGC CTAGAAAACC CTATGGAACA
             GTTGTA
MAM1_SP134   ACAAAGAGGT GGGCAGTTTG AACCCATCCA GTGGGTCCAT GGAGAAAGAC
             CTGGCGATTT GCTCCCGTAA AGATTACAGC CTAGAAAACC CTATGGAACA
             GTTGTA
MAST_A331_   ACAAAGAGGT GGGCAGTTTG AACCCATCCA GTGGGTCCAT GGAGAAAGAC
             CTGGCGATTT GCTCCCGTAA AGATTACAGC CTAGAAAACC TTATGGAACA
             GTTGTA
SAV1_SE210   ACAAAGAGGT GGGCAGTTTG AACCCATCCA GTGGGTCCAT GGAGAAAGAC
             CTGGCGATTT GCTCCCGTAA AGATTACAGC CTAGAAAACC CTATGGAACA
             GTTGTA

 1 1 1 1 1
5 102

ASIAN2_Ema   CTCCTGAGTC TCCCTTT-GC TCAAGACTTG TGTCTGTCAC ACCAGACAGA
             CCTTGTCATT CAGTCTGTGC TGTCACTCAA GCCCTCCCCA CCATAAGTAA
             AT
FOR_DS1535   CTCCTGAGTC TCCCTTT-GC TCAAGACTTG TGTCTGTCAC ACCAGATAGA
             CCTTGTCATT CAGTCTGTGC TGTCACTCAA GCCCTCCCCA CCATAAGTAA
             AT
MAM1_SP134   CTCCTGAGTC TCCCTTT-GC TCAAGACTTG TGTCTGTCAC ACCAGACAGA
             CCTTGTCATT CAGTCTGTGC TGTCACTCAA GCCCTCCCCA CCATAAGTAA
             AT
MAST_A373_   CTCCTGAGTC TTCCTTTTGT TTAAGACTTG TGTCTGTCAC ACCAGACAGA
             CCTTGTCATT CAGTCTGTGC TGTCACTCAA GCCCTCCCCA CCATAAGTAA
             AT
SAV1_SE210   CTCCTGAGTC TCTCTTT-GC TCAAGACTTG TGTCTGTCAC ACCAGATAGA
             CCTTGTCATT CAGTCTGTGC TGTCACTCAA GCCCTCCCCA CCATAAGTAA
             AT

 1 1 1 1 1
5 103

ASIAN2_Ema   TCCAAGTCAT CAGTGAGTGC CACAGGCGTC ACCCCTAAAC TACTGCATAT
             TCCAAATCCT CTTTGAGGCC TCCATTTCTC CGCCATAAAA TGCTGGGTCT
             GTG
FOR_DS1535   TCCAAGTCAT CAGTGAGTGC CACGGGCGTC ACCCCTAAAC TACTGCATAT
             TCCAAATCCT CTTTGAGGCC TCCATTTCTC CGCCATAAAA TGCTGGGTCT
```

```
                GTG
MAM1_SP134      TCCAAGTCAT CAGTGAGTGC CACAGGCGTC ACCCCTAAAC TACTGCATAT
                TCCAAATCCT CTTTGAGGCC TCCATTTCTC CGCCATAAAA TGCTGGGTCT
                GTG
MAST_A255_      TCCAAGTCAT CAGTGAGTGC CACGGGCGTC ACCCCTAAAC TACTGCATAT
                TCTAAATCCT CTTTGAGGCC TCCATTTCTC CGCCATAAAA TGCTGGGTCT
                GTA
SAV1_SE210      TCCAAGTCAT CAGTGAGTGC CACGGGCGTC ACCCCTAAAC TACTGCATAT
                TCCAAATCCT CTTTGAGGCC TCCATTTCTC CGCCATAAAA TGCTGGGTCT
                GTG

 1 1 1 1 1
5 90

ASIAN2_Ema      AAATCTGAGC TGGCAAAACT CTTCCTGGCT TGCCCTGATG AGCAGACCAT
                CTTTGTTTTC CATTAGTGTG TGTTGTCGTC ACAGTAACTG
FOR_DS1535      AAATCTGAGC TGGCAAAACT CTTCCTGGCT TGCCCTGATG AGCAGACCAT
                CTTTGTTTTC CATTAGTGTG TGTTGTCGTC ACAGTAACTG
MAM1_SP134      AAATCTGAGC TGGCAAAACT CTTCCTGGCT TGCCCTGATG AGCAGACCAT
                CTTTGTTTTC CATTAGTGTG TGTTGTCGTC ACAGTAACTG
MAST_A332_      AAATTTGAGC TGGCAAAACT CTTCCTGGCT TGCCCTGATG AGCAGACCAT
                CTTTGTTTTC CATTAGTGTG TGTTGTCGTC ACAGTAATTG
SAV1_SE210      AAATCTGAGC TGGCAAAACT CTTCCTGGCT TGCCCTGATG AGCAGACCAT
                CTTTGTTTTC CATTAGTGTG TGTTGTCGTC ACAGTAACTG

 1 1 1 1 1
5 103

ASIAN2_Ema      AGCTCCCCTC CTGCTATACC CCACAGAGTT GCTGAGGAAT CCCTCCTCCC
                TGTCACAGGG AGGTTCTCAC TCAAAAGCCC TTTCTGGGAA TGCCACAGCC
                CTT
FOR_DS1535      AGCTCCCCTC CTGCTATACC CCACAGAGTT GCTGAGGAAT CCCTCCTCCC
                TGTCACAGGG AGGTTCTCAC TCAAAAGCCC TTTCTGGGAA TGCCACAGCC
                CTT
MAM1_SP134      AGCTCCCCTC CTGCTATACC CCACAGAGTT GCTGAGGAAT CCCTCCTCCC
                TGTCACAGGG AGGTTCTCAC TCAAAAGCCC TTTCTGGGAA TGCCACAGCC
                CTT
MAST_A257_      AGCTCCCCTC CTGCTATATC CCACAGAGTT GCTGAGGAAT CCCTCCTCCC
                TGTCACAGGG AGGTTCTCAC TCAAAAGCCC TTTCTGGGAA TGCCACAGCC
                CTT
SAV1_SE210      AGCTCCCCTC CTGCTATACC CCACAGAGTT GCTGAGGAAT CCCTCCTCCC
                TGTCACAGGG AGGTTCTCAC TCAAAAGTCC TTTCTGGGAA TGCCACAGCC
                CTT

 1 1 1 1 1
5 101

ASIAN2_Ema      ATTTTAACTC TATCTGGTGC TATGAAAACA GGCAGAAAGC TATTAGGACA
                TGAGGAATTT GGGAAGGGTG AGTGAGAAGG GGTTTGAACT ACAATCTGAA
                A
FOR_DS1535      ATTTTAACTC TATCTGGTGC TATGAAAACA GGCAGAAAGC TATTAGGACA
                TGAGGAATTT GGGAAGGGTG AGTGAGAAGG GGTTTGAACT ACAATCTGAA
                A
MAM1_SP134      ATTTCAACTC TATCTGGTGC TATGAAAACA GGCAGAAAGC TATTAGGACA
                TGAGGAATTT GGGAAGGGTG AGTGAGAAGG GGTTTGAACT ACAATCTGAA
                A
MAST_A333_      ATTTTAACTC TATCTGGTGC TATGAAAACA GGCAGAAAGC TATTAGGACA
                TGAGGAATTT GGGAAGGGTG AATGAGAAGG GGTTTGAACT ACAGTCTGAA
                A
SAV1_SE210      ATTTTAACTC TATCTGGTGC TATGAAAACA GGCAGAAAGC TATTAGGACA
                TGAGGAATTC GGGAAGGGTG AGTGAGAAGG GGTTTGAACT ACAATCTGAA
                A

 1 1 1 1 1
5 116

ASIAN2_Ema      CAGAGGCTTT GGGGTCCTGG GCCAGATGGA CCAGTCTCTT TTTAAGTCCA
                GGGCTCCAGA GACACCCTGG GCTGATTTAC AGCCGCCTGG CCATAAAGGG
                GTGTTGGGAC CACATT
FOR_DS1535      CAGAGGCTTT GGGGTCCTGG GCCAGACGGA CCAGTCTCTT TTTAAGTCCA
                GGGCTCCAGA GACGCCCTGG GCTGATTTAC AGCTGCCCGG CCATAAAGGG
```

```
             GTGTTGGGAC CACATT
MAM1_SP134   CAGAGGCTTT GGGGTCCTGG GCCAGATGGA CCAGTCTCTT TTTAAGTCCA
             GGGCTCCAGA GACGCCCTGG GCTGATTTAC AGCCGCCCGG CCATAAAGGG
             GTGTTGGGAC CACATT
MAST_A258_   CAGAGGCTTT GGGGTCCTGG GCCAGATGGA CCAGTCTCTT TTTAAGTCTA
             GGGTTCCAGA GACGCCCTGG GCTGATTTAC AGCCGCCCGG CCATAAGGGG
             GTGTTGGGAC CACATT
SAV1_SE210   CAGAGGCTTT GGGGTCCTGG GCCAGACGGA CCAGTCTCTT TTTAAGTCCA
             GGGCTCCAGA GACGCCCTGG GCTGATTTAC AGCTGCCCGG CCATAAAGGG
             GTGTTGGGAC CACATT

 1 1 1 1 1
5 110

ASIAN2_Ema   TTGAATATTT GAGAATTTTT -CAGAAATAT TTCATGGTCC TGTATTGTGG
             CCCTGTCTGT GTCTGATCCC AGCGGTACCA CACCTATCTT AAAA-TGGAT
             TGCCACTGGG
FOR_DS1535   TTGAATATTT GAGAATTTTT -CAGAAATAT TTCATGGTCC TGTATTCTGG
             CCCTGTCTGT GTCTGATCCC AGCGGTACCA CACCTATCTT AAAA-TGGAT
             TGCCACTGGG
MAM1_SP134   TTGAATATTT GAGAATTTTT -CAGAAATAT TTCATGGTCC TGTATTCTGG
             CCCTGTCTGT GTCTGATCCC AGCGGTACCA CACCTATCTT AAAA-TGGAT
             TGCCACTGGG
MAST_A259_   TTGAATATTT GAGAATTTTT TCAGAAATAT TTCATGGTCC TGTATTCTGG
             CCCTGTCTGT GTCTGATTCC AGCGGTACCA CACTTATCTT AAAAATGGAT
             TGCCCCTGGG
SAV1_SE210   TTGAATATTT GAGAATTTTT -CAGAAATAT TTCATGGTCC TGTATTCTGG
             CCCTGTCTGT GTCTGATCCC AGCGGTACCA TACCTATCTT AAAA-TGGAT
             TGCCACTGGG

 1 1 1 1 1
5 108

ASIAN2_Ema   CATCATTAAA TTAG-AAACA AA-TGATGTA TTTGCAGAAA TTGGTTTCTT
             AACTGGTCTT GACCTGGGAT GACATCTCAT ATCCGGAATG TTTTCACCCA
             A-GCA-CG
FOR_DS1535   CATCATTAAA TTAG-AAACA AA-TGATGTA TTTGCAGAAA TTGGTTTCTT
             AACTGGTCTT GACCTGGGAT GACATCTCAT ATCCAGAATG TTTTCACCCA
             A-GCA-CG
MAM1_SP134   CATCATTAAA TTAG-AAACA AA-TGATGTA TTTGCAGAAA TTGGTTTCTT
             AACTGGTCTT GACCTGGGAT GACATCTCAT ATCCGGAATG TTTTCACCCA
             A-GCA-CG
MAST_A334_   CATCATTAAA TTAAGAAACA AAATGATGTA TTTGCAGAAA TTGGTTTCTT
             AACTGGTCTT GACCTGGGAT GACATCTCAT ATCCGGAATG TTTTCACCCA
             AAGCAACG
SAV1_SE210   CATCATTAAA TTAG-AAACA AA-TGATGTA TTTGCAGAAA TTGGTTTCTT
             AACTGGTCTT GACCTGGGAT GACATCTCAT ATCCAGAATG TTTTCACCCA
             A-GCA-CG

 1 1 1 1 1
5 115

ASIAN2_Ema   CAGAAAATCA CTAAACAATA TTATTGATGC CATTTTTCTT TCTAAGATCA
             GCTTTGGAGG GATTCCAGCA GCTGGAAAAT CAGTGTCGTT CCCACAAAAA
             AATTTT-CAT GGGTG
FOR_DS1535   CAGAAAATCA CTAAACAATA TTATTGATGC CATTTTTCTT TCTAAGATCA
             GCTTTGGAGG GATTCCAGCA GCTGGAAAAT CAGTGTCGTT CCCACAAAAA
             AATTTT-CAT GGGTG
MAM1_SP134   CAGAAAATCA CTAAACAATA TTATTGATGC CATTTTTCTT TCTAAGATCA
             GCTTTGGAGG GATTCCAGCA GCTGGAAAAT CAGTGTCGTT CCCACAAAAA
             AATTTT-CAT GGGTG
MAST_A262_   TAGAAAATCA CTAAACAATA TTATTGATGC CATTTTTCTT TCTAAGATCA
             GCTTTGGAGG GATTCCAGCA GCTGGAAAAT CAGTGTCGTT CCCACAAAAA
             AATTTTACAC GGGTG
SAV1_SE210   CAGAAAATCA TTAAACAATA TTATTGATGC CATTTTTCTT TCTAAGATCA
             GCTTTGGAGG GATTCCAGCA GCTGGAAAAT CAGTGTCGTT CCCACAAAAA
             A-TTTT-CAT GGGTG

 1 1 1 1 1
5 92
```

```
ASIAN2_Ema   TCGATCACAG ATGAACTTAG CACCCACGGA ATGGTTGATG CCAGTAACTC
             TGTGTGTCTC ATTCCCAGCA GTCTAGTGGC TGCCTGAAAG TT
FOR_DS1535   TCGATCACAG ATGAACTTAG CACCCACGGA ATGGTTGATG CCAGTAACTC
             TGTGTGTCTC ATTCCCAGCA GTCTAGTGGC TGCCTGAAAG TT
MAM1_SP134   TCGATCACAG ATGAACTTAG CACCCACGGA ATGGTTGATG CCAGTAACTC
             TGTGTGTCTC ATTCCCAGCA GTCTAGTGGC TGCCTGAAAG TT
MAST_A335_   TTGATCACAG ATGAACTTAG CACCCACGGA ATGGTTGATG CCGGTAACTC
             TGTGTGTCTC ATTCCCAGCA GTCTAGTGGC TGCCTGAAAG TT
SAV1_SE210   TCGATCACAG ATGAACTTAG CACCCACGGA ATGGTTGATG CCAGTAACTC
             TGTGTGTCTC ATTCCCAGCA GTCTAGTGGC TGCCTGAAAG TT

 1 1 1 1 1
5 96

ASIAN2_Ema   AGAGAGAGAA ATTGGAACTG GGGCCCAGGA ATTAGAATTT GGGATTTAAC
             ATATAAAATG TGCCTTAATA CGTTTGCATC CTGTAGACCA AACCAG
FOR_DS1535   AGAGAGAGAA ATTGGAACTG GGGCCCAGGA ATTAGAATTT GGGATTTAAC
             ATATAAAATG TGCCTTAATA CGTTTGCATC CTGTAGACCA AACCAG
MAM1_SP134   AGAGAGAGAA ATTGGAACTG GGGCCCAGGA ATTAGAATTT GGGATTTAAC
             ATATAAAATG TGCCTTAATA CGTTTGCATC CTGTAGACCA AACCAG
MAST_A264_   AGAGAGAGAA ATTGGAACTG GGGCCCAGGA ATTAGAATTT GGGATTTAAC
             ATATAAAATG TGCCTTAATA TGTTTTCATC CTGTAGACCA AACCAG
SAV1_SE210   AGAGAGAGAA ATTGGAACTG GGGCCCAGGA ATTAGAATTT GGGATTTAAC
             ATATAAAATG TGCCTTAATA CGTTTGCATC CTGTAGACCA AACCAG

 1 1 1 1 1
5 97

ASIAN2_Ema   TTTAAAATCA GGAAAGATGT GTGTCACGGT TGTATTCTTT CACTATACTT
             ATTCAATCTG TATGCTGAAC AAATAATCCA AGAATTGGCA TCAGGAT
FOR_DS1535   TTTAAAATCA GGAAAGATGT GTGTCACGGT TGTATTCTTT CACTATACTT
             ATTCAATCTG TATGCTGAAC AAATAATCCA AGAATTGGCA TCAGGAT
MAM1_SP134   TTTAAAATCA GGAAAGATGT GTGTCACGGT TGTATTCTTT CACTATACTT
             ATTCAATCTG TATGCTGAAC AAATAATCCA AGAATTGGCA TCAGGAT
MAST_A431_   TTTAAAATCA GGAAAGATGT GTGTCACGGT TGTATTCTTT CACTATACTT
             ATTCAATCTG TATGCTGAAC AAATAATCCA AGAACTGGCA TCAGGAC
SAV1_SE210   TTTAAAATCA GGAAAGATGT GTGTCACGGT TGTATTCTTT CACTATACTT
             ATTCAATCTG TATGCTGAAC AAATAATCCA AGAATTGGCA TCAGGAT

 1 1 1 1 1
5 90

ASIAN2_Ema   ACCTGATGGA GAGGTCTTGC TTCATCCACC TCCAGGGACC TCCCTCACCA
             TCAATCCTGG TGGCCTCTGG CCAGGGTGAG ACAAACAGGG
FOR_DS1535   ACCTGATGGA GAGGTCTTGC TTCATCCACC TCCAGGGACC TCCCTCACCA
             TCAATCCTGG TGGCCTCTGG CCAGGGTGAG ACAAACAGGG
MAM1_SP134   ACCTGATGGA GAGGTCTTGC TTCATCCACC TCCAGGGACC TCCCTCACCA
             TCAATCCTGG TGGCCTCTGG CCAGGGTGAG ACAAACAGGG
MAST_A432_   ACCTGATGGA GAGGTCTTGC TTCATCCACC TCCAGGGACC TCCCTCACCA
             TCAATCCTGG TGGCCTCTGG CCAGGGTGAG ACAAACAGAA
SAV1_SE210   ACCTGATGGA GAGGTCTTGC TTCATCCACC TCCAGGGACC TCCCTCACCA
             TCAATCCTGG TGGCCTCTGG CCAGGGTGAG ACAAACAGGG

 1 1 1 1 1
5 94

ASIAN2_Ema   CTTTTATAAG TCACAGGACT AGGGTCAGGG AGGAGCAAGT TTCCAGAAGC
             CATGGCAGTG GAGCCATAAA TCAGCCAGGC TCTCCCCAGC GGCA
FOR_DS1535   CTTTTATAAG TCACAGGACT AGGGTCAGGG AGGAGCAAGT TTCCAGAAGC
             CATGGCAGTG GAGCCATAAA TCAGCCAGGC TCTCCCCAGC GGCA
MAM1_SP134   CTTTTATAAG TCACAGGACT AGGGTCAGGG AGGAGCAAGT TTCCAGAAGC
             CATGGCAGTG GAGCCATAAA TCAGCCAGGC TCTCCCCAGC GGCA
MAST_A337_   CTTTTATAAG TCACAGGACT AGGGTCAGGG AGGAGCAAGT TTCCAGAAGC
             CATGGCAGTG GAGCCATAAA TCAGCCAGAC TCTCCCCAGC GGCA
SAV1_SE210   CTTTTATAAG TCACAGGACT AGGGTCAGGG AGGAGCAAGT TTCCAGAAGC
             CATGGCAGTG GAGCCATAAA TCAGCCAGGC TCTCCCCAGC GGCA

 1 1 1 1 1
5 103

ASIAN2_Ema   CACAGTCTCC TCGTGGCTGG AAAAGAGTGA GATCACCACG GTTGGGCTTC
```

```
             TTGCCTTTCC CCTATTGGCT AAGCAGGATT CTTTTACATT CCAAAATAAG
             AAT
FOR_DS1535   CACAGTCTCC TCGTGGCTGG AAAAGAGTGA GATCACCACG GTTGGGCTTC
             TTGCCTTTCC CCTATTGGCT AAGCAGGATT CTTTTACATT CCAAAATAAG
             AAT
MAM1_SP134   CACAGTCTCC TCGTGGCTGG AAAAGAGTGA GATCACCACG GTTGGGCTTC
             TTGCCTTTCC CCTATTGGCT AAGCAGGATT CTTTTACATT CCAAAATAAG
             AAT
MAST_A338_   TACAGTCTCC TCGTGGCTGG AAAAGAGTGA GATCACCAGA GTTGGGCTTC
             TTGCCTTTCC CCTATTGGCT AAGCAGGATT CTTTTACGTT CCAAAATAAG
             AAT
SAV1_SE210   CACAGTCTCC TCGTGGCTGG AAAAGAGTGA GATCACCACG GTTGGGCTTC
             TTGCCTTTCC CCTATTGGCT AAGCAGGATT CTTTTACATT CCAAAATAAG
             AAT

 1 1 1 1 1
5 101

ASIAN2_Ema   CCTGTGGCAA CTTGGAGGAA CCTCAGGTTA GCTGCATTCC TCAAGTATAG
             AAGTCTTGAG ATAGCCCAGA GCATCAGACT ATTAAAGCTC TTTCAATAGG
             G
FOR_DS1535   CCTGTGGCAA CTTGGAGGAA CCTCAGGTTA GCTGCATTCC TCAAGTATAG
             AAGTCTTGAG ATAGCCCAGA GCATCAGACT ATTAAAGCTC TTTCAATAGG
             G
MAM1_SP134   CCTGTGGCAA CTTGGAGGAA CATCAGGTTA GCTGCATTCC TCAAGTATAG
             AAGTCTTGAG ACAGCCCAGA GCATCAGACT ATTAAAGCTC TTTCAATAGG
             G
MAST_A339_   CCTGTGGCAA CTTGGAGGAA CCTCAGGTTA GCTGCATTCC TCAAGTATAG
             AAGTCTTGAG ATAGCCCAGA GCATCAGACT ATTAAAGCTC TTTCAATAAA
             A
SAV1_SE210   CCTGTGGCAA CTTGGAGGAA CCTCAGGTTA GCTGCATTCC TCAAGTATAG
             AAGTCTTGAG ATAGCCCAGA GCATCAGACT ATTAAAGCTC TTTCAATAGG
             G

 1 1 1 1 1
5 95

ASIAN2_Ema   GTTCCAGCAT GCCCCCA-TA CTGCTGCTGT ACTTTTCTGC TTCTCTCATG
             GACTAATAAT CTTGAGAGAG GGATTAGATG GCTAGTCAGT GGAGA
FOR_DS1535   GTTCCAGCAT GCCCCCA-TA CTGCTGCTCT ACTTTTCTGC TTCTCTCATG
             GACTAATAAT CTTGAGAGAG GGATTAGATG GCTAGTCAGT GGAGA
MAM1_SP134   GTTCCAGCAT GCCCCCA-TA CTGCTGCTGT ACTTTTCTGC TTCTCTCATG
             GACTAATAAT CTTGAGAGAG GGATTAGATG GCTAGTCAGT GGAGA
MAST_A266_   GTTCCAGCAT GCCCCCACTA CTGCTGCTCT ACTTTTCTGC TTCTCTCATG
             GATTAATAAT CTTGAGAGAG GGATTAGATG TCTAGTCAGT GGAGG
SAV1_SE210   GTTCCAGCAT GCCCCCA-TA CTGCTGCTCT ACTTTTCTGC TTCTCTCATG
             GACTAATAAT CTTGAGAGAG GGATTAGATG GCTAGTCAGT GGAGA

 1 1 1 1 1
5 93

ASIAN2_Ema   CTGCAGACAC TCAATGTGTT ACTTTCTATG GCGAGGACAG CCTAGCCCAG
             TTTATGTTCA ACCACACCTG TCATAATTGC ATAATCACGG CAT
FOR_DS1535   CTGCAGACAC TCAATGTGTT ACTTTCTATG GCGAGGACAG CCTAGCCCAG
             TTTATGTTCA ACCACACCTG TCATAATTGC ATAATCACGG CAT
MAM1_SP134   CTGCAGACAC TCAATGTGTT ACTTTCTATG GCGAGGACAG CCTAGCCCAG
             TTTATGTTCA ACCACACCTG TCATAATTGC ATAATCACGG CAT
MAST_A267_   CTGCAGACAC TCAATGTGTT ACTTTCTATG GCGAGGACAG CCTAGCCCAG
             TTTATGTTCA ACCACGCCTG TCATAATTGC ATAAATCACG GCA
SAV1_SE210   CTGCAGACAC TCAACGTGTT ACTTTCTATG GCGAGGACAG CCTGGCCCAG
             TTTATGTTCA ACCACACCTG TCATAATTGC ATAATCACGG CAT

 1 1 1 1 1
5 95

ASIAN2_Ema   TTGATGTTCT CATCAAAGGG GAAATGCCTG AGAACCAAAA TCAGACTCAC
             TTCTTCTATT CTGTATGCCC AGGCTCTTAT TCTGAGTCAG TGGCC
FOR_DS1535   TTGAKGTTCT CATCAAAGGG GAAATRCCTG AGAACCAAAA TCAGACTCAC
             TTCTTCTATT CTGTATGCCC AGGCTCTTAT TCKGAGTCAG TGGCC
MAM1_SP134   TTGATGTTCT CATCAAAGGG GAAATGCCTG AGAACCAAAA TCAGACTCAC
             TTCTTCTGTT CTGTATGCCC AGGCTCTTAT TCTGAGTCAG TGGCC
```

```
MAST_A268_   TTGATGTTCT CATCAAAGGG GAAATGCCTG AGAACCAAAA TCAGACTCAC
             TTCTTCTATT CTGTATGCCC AGGCTCTTAT TCTGAGTCAG TGGCC
SAV1_SE210   TTGAGGTTCT CATCAAAGGG GAAATGCCTG AGAACCAAAA TCAGACTCAC
             TTCTTCTGTT CTGTATGCCC AGGCTCTTAT TCGGAGTCAG TGGCC

 1 1 1 1 1
5 92

ASIAN2_Ema   GCAAAATTGA TTTTT-CTAA GTGCAGAGGC CAGTCTTATG ACAATGCTGC
             CAATATGTCA GGGTGTTATA AGGGCGTGCA ACAGAAAATT TT
FOR_DS1535   GCAAAATTGA TTTTT-CTAA GTGCAGAGGC CAGTCTTATG ACAATGCTGC
             CAATATGTCA GGGTGTTATA RAGGCACGCA ACAGAAAATT TT
MAM1_SP134   GCAAAATTGA TTTTT-CTAA GTGCAGAGGC CAGTCTTATG ACAATGCTGC
             CAATATGTCA GGGTGTTATA AGGGCGTGCA ACAGAAAATT TT
MAST_A435_   GAAAAATTGA TTTTTTCTAA -TGCAGAGGC CAGTCCTATG ACAATGCTGT
             CAATATGTCA GAGTGTAATA AAAGCATGCA ACAGAAAATT TT
SAV1_SE210   GCAAAATTGA TTTTT-CTAA GTGCAGAGGC CAGTCTTATG ACAATGCTGC
             CAATATGTCA GGGTGTTATA AAGGCATGCA ACAGAAAATT TT

 1 1 1 1 1
5 105

ASIAN2_Ema   CTTGGAAACT CTATAGAGCC ATTCTAGTCT GCCCTGTAGG GTTGTTATGA
             GTCAGAATTG ACTCGATGGC AGTGGGTTT- GGTTTTTT-- GTTTTTTTTT
             --AGG
FOR_DS1535   CTTGGAAACC CTATAGAGCC ATTCTAGTCT GCCCTGTAGG GTTGTTATGA
             GTCAGAATTG ACTCGATGGC AGTGGGTTT- GGTTTTTT-- GTTTTTTTTT
             --AGG
MAM1_SP134   CTTGGAAACT CTATAGAGCC ATTCTAGTCT GCCCTGTAGG GTTGTTATGA
             GTCAGAATTG ACTCGATGGC AGTGGGTTT- GGTTTTTTT- GGTTTTTTT-
             --AGG
MAST_A342_   CTTGGAAACT CTATAGAGCC ATTCTAGTCT GCCCTGTAGG GTTGTTATGA
             GTCGGAATTG ACTCGATGGC AGTGGGTTTT GGTTTTTTTT ATTTTTTTTT
             TTAGG
SAV1_SE210   CTTGGAAACT CTATAGAGCC ATTCTAGTCT GCCCTGTAGG GTTGTTATGA
             GTCAGAATTG ACTCGATGGC AGTGGGTTT- GGTTTTTTT- GTTTTTTTTT
             --AGG

 1 1 1 1 1
5 106

ASIAN2_Ema   GAGCAATGGT TCTTCTCTTG AGCACCTCAG TGTCTGGGGG AAGTCAATGG
             CCGGGAGGGG GGTGTAACTC AATGAGATGA GAATAGTTGG GGGGCGTGAG
             AGATCA
FOR_DS1535   GAGCAATGGT TCTTCTCTTG AGCACCTCAG TGTCTGGGGG AAGTCAATGG
             CCGGGAGGGG GGTGTAACTC AATGAGATGA GAATAGTTGG GGGGCGTGAG
             AGATCA
MAM1_SP134   GAGCAATGGT TCTTCTCTTG AGCACCTCAG TGTCTGGGGG AAGTCAATGG
             CCGGGAGGGG GGTGTAACTC AATGAGATGA GAATAGTTGG GGGGCGTGAG
             AGATCA
MAST_A343_   GAGCAATGGT TCTTCTTTTG AGCGCCTCAG TGTCTGGGGG AAGTCAATGG
             CCGGGAGGGG GGTGTAACTC AATGAGATGA GAATAGTTGG GGGCGGTGAG
             AGATCA
SAV1_SE210   GAGCAATGGT TCTTCTCTTG AGCACCTCAG TGTCTGGGGG AAGTCAATGG
             CCGGGAGGGG GGTGTAACTC AATGAGATGA GAATAGTTGG GGGGCGTGAG
             AGATCA

 1 1 1 1 1
5 93

ASIAN2_Ema   TGCAAAAGAG GCTTTGTAGG GAATACAAGA AAATTGGAGA CCCCTGGGAG
             GGCAGATCTC GACTTAATAG GAGCCTGTAA TTACGTGGCA AAG
FOR_DS1535   TGCAAAAGAG GCTTTGTAGG GAATACAAGA AAATTGGAGA CCCCTGGGAG
             GGCAGATCTC GACTTAATAG GAGCCTGTAA TTACGTGGCA AAG
MAM1_SP134   TGCAAAAGAG GCTTTGTAGG GAATACAAGA AAATTGGAGA CCCCTGGGAG
             GGCAGATCTC GACTTAATAG GAGCCTGTAA TTACGTGGCA AAG
MAST_A380_   TGCAAAAGAG GCTTTGTAGG GAATACAAGA AAATTGGAGA CCCCTGGGAG
             GGCAGATCTC GACTTAATAG GAGCCTGTAA TTACATGGCA AAG
SAV1_SE210   TGCAAAAGAG GCTTTGTAGG GAATACAAGA AAATTGGAGA CCCCTGGGAG
             GGCAGATCTC GACTTAATAG GAGCCTGTAA TTACGTGGCA AAG
```

```
 1 1 1 1 1
5 92

ASIAN2_Ema   GGAAGAGGAC CTGGAGGATG AGAGAGGAGC CATTGAGGCC AGGCTGGGGT
             GGGCAAGAGT CAGGGAGACC TCGCCAGTGT CCAGCACAGG TT
FOR_DS1535   GGAAGAGGAC CTGGAGGATG AGAGAGGAGC CATTGAGGCC AGGCTGGGGT
             GGGCAAGAGT CAGGGAGACC TCACCAGTGT CCAGCACAGG TT
MAM1_SP134   GGAAGAGGAC CTGGAGGATG AGAGAGGAGC CATTGAGGCC AGGCTGGGGT
             GGGCAAGAGT CAGGGAGACC TCGCCAGTGT CCAGCACAGG TT
MAST_A344_   GGAAGAGGAC CTGGAGGATG AGAGAGGAGC CACTGAGGCC AGGCTGGGGT
             GGGCAAGAGT CAGGGAGACC TCGCCAGTGT CCAGCACAGG TT
SAV1_SE210   GGAAGAGGAC CTGGAGGATG AGAGAGGAGC CATTGAGGCC AGGCTGGGGT
             GGGCAAGAGT CAGGGAGACC TCGCCAGTGT CCAGCACAGG TT

 1 1 1 1 1
5 102

ASIAN2_Ema   AGGTTTAACT ACATAGGGAC CATCTTATTG AGGAACGTCA TCACCCTTTG
             TCTCTTCTTC CTCCCGAGTG CAAGCTTTCC CCTTCCCCCT TACAGTTGTA
             AT
FOR_DS1535   AGGTTTAACT ACATAGGGAC CATCTTATTG AGGAACGTCA TCACYCTTTG
             TCTCTTCTTC CTCCCGAGTG CAAGCTTTCC CCTTCCCCCT TACAGTTGTA
             AT
MAM1_SP134   AGGTTTAACT ACATAGGGAC CATCTTATTG AGGAACGTCA TCACCCTTTG
             TCTCTTCTTC CTCCCGAGTG CAAGCTTTCC CCTTCCCCCT TACAGTTGTA
             AT
MAST_A438_   AGGTTTTACT ATATAGGGAT CATCTTATTG AGGAATGTCA CCACCCTTTG
             TCTCTTCTTC CTCCCGGGTG CAAGCTTTCC CCTTCCCCCT TACAGTTGTA
             AT
SAV1_SE210   AGGTTTAACT ACATAGGGAC CATCTTATTG AGGAACGTCA TCACCCTTTG
             TCTCTTCTTC CTCCCGAGTG CAAGCTTTCC CCTTCCCCCT TACAGTTGTA
             AT

 1 1 1 1 1
5 95

ASIAN2_Ema   CAATCCATAG CTAAGTGGGA CGAGGAAGGA GCAGTGGACA GTGCCATGGA
             TTCCTTGGAC TTTATTATCT TGTTTACCTA GTCTTTTTGA TGTAG
FOR_DS1535   CAATCCATAG CTAAGTGGGA CGAGGAAGGA GCAGTGGACA GTGCCATGGA
             TTCCTTGGAC TTTATTATCT TGTTTACCTA GTCTTTTTGA TGTAG
MAM1_SP134   CAATCCATAG CTAAGTGGGA CGAGGAAGGA GCAGTGGACA GTGCCATGGA
             TTCCTTGGAC TTTATTATCT TGTTTACCTA GTCTTTTTGA TGTAG
MAST_A269_   CAATCCATAG CTAAGTGGGA CGAGGAAGGA GCAGTGGACA GTGCCATGGA
             TTCCTTGGAC TTTATTATCT TGTTTACCTA GTCTTTTTGA TGTAA
SAV1_SE210   CAATCCATAG CTAAGTGGGA CGAGGAAGGA GCAGTGGACA GTGCCATGGA
             TTCCTTGGAC TTTATTATCT TGTTTACCTA GTCTTTTTGA TGTAG

 1 1 1 1 1
5 102

ASIAN2_Ema   CCAATCCCTG GAAAAGGACA TCGTGCTTGG TAAAGCAGAA GGTCAGCGAC
             ACAATGGCTG CAACAA-TGG GCTCAAACAC AGGGACAATT GTGAAGATGG
             CA
FOR_DS1535   CCAATCCCTG GAAAAGGACA TCGTGCTTGG TAAAGCAGAA GGTCAGCGAC
             ACAATGGCTG CAACAA-TGG GCTCAAACAC AGGGACAACT GTGAAGATGG
             CA
MAM1_SP134   CCAATCCCTG GAAAAGGACA TCGTGCTTGG TAAAGCAGAA GGTCAGCGAC
             ACAATGGCTG CAACAA-TGG GCTCAAACAC AGGGACAATT GTGAAGATGG
             CA
MAST_A381_   CCAATTTCTG GAAAAGGACA TCGTGCTTGG TAAAGCAGAA GGTCAGCGAC
             ACAATGGCTG CAACAAACGG GCTCAAACAC AGGGACAATT GTGAAGACGG
             CA
SAV1_A381_   CCAATCCCTG GAAAAGGACA TCGTGCTTGG TAAAGCAGAA GGTCAGCGAC
             ACAATGGCTG CAACAA-TGG GCTCAAACAC AGGGACAACT GTGAAGATGG
             CA

 1 1 1 1 1
5 91

ASIAN2_Ema   ATCTTTTTGC TCTAACTTTC ATACAATGTA AGTGAGATTC CAAGTATGCA
             AATGGTGGGA GGAAAGAAAT GCATTTTCAC TACCCCAATT A
```

```
FOR_DS1535   ATCTTTTTGC TCTAACTTTC ATACAATGTA AGTGAGATTC CAAGTATGCA
             AATGGTGGGA GGAAAGAAAT GCATTTTCAC TACCCCAATT A
MAM1_SP134   ATCTTTTTGC TCTAACTTTC ATACAATGTA AGTGAGATTC CAAGTATGCA
             AATGGTGGGA GGAAAGAAAT GCATTTTCAC TACCCCAATT A
MAST_A439_   TTTTTTTTGC TCTAACTTTC ATACAATGTA AGTGAGATTC CAAGTATGCA
             AATGGTGGGA GGAAAGAAAT GCATTTTCAC TACCCCAATT A
SAV1_SE210   ATCTTTTTGC TCTAACTTTC ATACAATGTA AGTGAGATTC CAAGTATGCA
             AATGGTGGGA GGAAAGAAAT GCATTTTCAC TACCCCAATT A

 1 1 1 1 1
5 94

ASIAN2_Ema   ACAGGGTGGC AGTCTGGGTC ATCAGAGCCC TCGTCACTCC CGGAAGAGCT
             AGCCTTGCCC CTTCTGGCCC AGCCTCGCTC CCTGACTCCT ACAA
FOR_DS1535   ACAGGGTGGC AGTCTGGGTC ATCAGAGCCC TCGTCACTCC CGGAAGAGCT
             AGCCTTGCCC CTTCTGGCCC AGCCTCGCTC CCTGACTCCT ACAA
MAM1_SP134   ACAGGGTGGC AGTCTGGGTC ATCAGAGCCC TCGTCACTCC CGGAAGAGCT
             AGTCTTGCCC CTTCTGGCCC AGCCTCGCTC CCTGACTCCT ACAA
MAST_A270_   -CAGGGTGGC AGTCTGGGTC ATCAGAGCCC TCGTCACTCC CGGAAGAGCT
             AGCCTTGCCC CTTCTGGCCC AGCCTCACTC CCTGACTCCT ACAA
SAV1_SE210   ACAGGGTGGC AGTCTGGGTC ATCAGAGCCC TCGTCACTCC CGGAAGAGCT
             AGTCTTGCCC CTTCTGGCCC AGCCTCGCTC CCTGACTCCT ACAA

 1 1 1 1 1
5 96

ASIAN2_Ema   TGCTCCAG-- -CTTCTTGAT GGTAGTGAGG TCCCCCTCAG CCTCTGTAGG
             ACTGGCCCTT ATATTCAATT TCAAGGCTTG GCCCTCC-AG CCAGAC
FOR_DS1535   TGCTCCAG-- -CTTCTTGAT GGTAGTGAGG TCCCCCTCAG CCTCTGTAGG
             ACTGGCCCTT ATATTCAATT TCAAGGCATG GCCCTCC-AG CCAGAC
MAM1_SP134   TGCTCCAG-- -CTTCTTGAT GGTAGTGAGG TCCCCCTCAG CCTCTGTAGG
             ACTGGCCCTT ATATTCAATT TCAAGGCTTG GCCCTCC-AG CCAGAC
MAST_A271_   CATTCCAGGC TCTTCTTGAT GGTGGTGAGG TCCCCCTCAG CCTCTGTAGG
             ATTGGCCCTT ATATTCAATT TCATGGCATG GCCCTCCCAG CCAGAC
SAV1_SE210   TGCTCCAG-- -CTTCTTGAT GGTAGTGAGG TCCCCCTCAG CCTCTGTAGG
             ACTGGCCCTT ATATTCAATT TCAAGGCATG GCCCTCC-AG CCAGAC

 1 1 1 1 1
5 94

ASIAN2_Ema   TGATACGGCC ACAGCTCTGC CGAGAATGGA GTCCCTGGAT GGTGCAAATG
             GCTAAGACGC TGGGCTCCTA ACCAAAAGGT TAGAGGTTCA TCTC
FOR_DS1535   TGATATGGCC ACAGCTCTGC CGAGAATGGA GTCCCTGGAT GGTGCAAATG
             GCTAAGACGC TGGGCTCCTA ACCAAAAGGT TAGAGGTTCA TCTC
MAM1_SP134   TGATACGGCC ACAGCTCTGC CGAGAATGGA GTCCCTGGAT GGTGCAAATG
             GCTAAGACGC TGGGCTCCTA ACCAAAAGGT TAGAGGTTCA TCTC
MAST_A272_   TGATACAGCC ACAGCTCTGC CGAGAATGGA GTCCCTGGAT GGTGCAAATG
             GCTAAGACGC TGGGCTCCTA ACCAAAAGGT TAGAGGTTCA TATC
SAV1_SE210   TGATACGGCC ACAGCTCTGC CGAGAATGGA GTCCCTGGAT GGTGCAAATG
             GCTAAGACGC TGGGCTCCTA ACCAAAAGGT TAGAGGTTCA TGTC

 1 1 1 1 1
5 91

ASIAN2_Ema   ATCTGTTGAT GTCAATTTCT CCTTTCCTGC AGCCTGGGCT GCATCTGTGT
             CCTCCCTGGC ATCGGTGTGC ATGCCAGTAG CTTCTTTTTC T
FOR_DS1535   ATCTGTTGAT GTCAATTTCT CCTTTCCTGC AGCCTGGGCT GCATCTATGT
             CCTCCCTGGC ATCGGTGTGC ATGCCAGTAG CTTCTTTTTC T
MAM1_SP134   ATCTGTTGAT GTCAATTTCT CCTTTCCTGC AGCCTGGGCT GCATCTGTGT
             CCTCCCTGGC ATCGGTGTGC ATGCCAGTAG CTTCTTTTTC T
MAST_A382_   ATCTGTTGAT GTCAATTTCT CCTTTCCTGC AGCCTGGGCT GCGTCTATGT
             CCTCCCTGGC TTCGGTGTGC ATGCCAGTAA CTTCTTTTTC T
SAV1_SE210   ATCTGTTGAT GTCAATTTCT CCTTTCCTGC AGCCTGGGCT GCATCTGTGT
             CCTCCCTGGC ATTGGTGTGC RTGCCAGTAG CTTCTTTTTC T

 1 1 1 1 1
5 104

ASIAN2_Ema   GGAGACTGGC CAAAAACTGC TACGGTGGGC TTACCAGCCC ATGGAGACAG
             GCAGCTGAAG CGGGCTTGGC AGCCCACGGA GCAAGAGAGC TGAGTGCCTT
             TGGG
```

FOR_DS1535 GGAGACTGGC CAAAAACTGC TACGGTGGGC TTACCAGCCC ATGGAGACAG
GCAGCTGAAG CGGGCTTGGC AGCCCACGGA GCAAGAGAGC TGAGTGCCTT
TGGG
MAM1_SP134 GGAGACTGGC CAAAAACTGC TACGGTGGGC TTACCAGCCC ATGGAGACAG
GCAGCTGAAG CGGGCTTGGC AGCCCACGGA GCAAGAGAGC TGAGTGCCTT
TGGG
MAST_A273_ GGAGACTGGC CAAAAACTGC TACGGTGGGC TTACCAGCCC ATGGAGACAG
GCGGTTGAAG CGGGCTTGCC AGCCCACGGA GCAAGAGAGC TGAGTGCCCT
TGGA
SAV1_SE210 GGAGACTGGC CAAAAACTGC TACGGTGGGC TTACCAGCCC ATGGAGACAG
GCAGCTGAAG CGGGCTTGGC AGCCCACGGA GCAAGAGAGC TGAGTGCCTT
TGGG

1 1 1 1 1
5 103

ASIAN2_Ema GCAGCCAGGA CTCAGACATG CCCAGAAGGG GATGTGGATA GGGAGACCTG
GGAAGGGG-C AGTTTTGAGA GAGGGATAAG TTGGGCAGGG GGG-CAGGAG
AGG
FOR_DS1535 GCAGCCAGGA CTCAGACATG CCCAGAAGGG GATGTGGATA GGGAGACCTG
GGAAGGGG-C AGTTTTGAGA GAGGGATAAG GTGGGCAGGG GGG-CAGGAG
AGG
MAM1_SP134 GCAGCCAGGA CTCAGACATG CCCAGAAGGG GATGTGGATA GGGAGACCTG
GGAAGGGG-C AGTTTTGAGA GAGGGATAAR GTGGGCAGGG GGG-CAGGAG
AGG
MAST_A277_ GCAGCCAGGA CTCAGACATG CCCAGAAGGG GATGTGGATA GGGAGATCTG
GGAAGGGGGC AGTTTTGAAA GAGGGATAAG GTGGGCAGGG GGGGCAGGAG
AGA
SAV1_SE210 GCAGCCAGGA CTCAGACATG CCCAGAAGGG GATGTGGATA GGGAGACCTG
GGAAGGGG-C AGTTTTGAGA GAGGGATAAG GTGGGCAGGG GGG-CAGGAG
AGG

1 1 1 1 1
5 104

ASIAN2_Ema CTGCCAAGCT CCTGGTTCTG GTCATGAATG TTATTTGTTT TTCTAATTCC
TCTCTAAAAT TGCCCTGCTT ATGAATGCCA GAGTCCCAGC AAGCCTTATC
TCCC
FOR_DS1535 CTGCCAAGCT CCTGGTTCTG GTCATGAATG TTATTTGTTT TTCTAATTCC
TCTCTAAAAT TGCCCTGCTT ATGAATGCCA GAGTCCCAGC AAGMCTTATC
TCCC
MAM1_SP134 CTGCCAAGCT CCTGGTTCTG GTCATGAATG TTATTTGTTT TTCTAATTCC
TCTCTAAAAT TGCCCTGCTT ATGAATGCCA GAGTCCCAGC AAGCCTTATC
TCCC
MAST_A442_ TTGTCAAGCT TCTGGTTTTG GTCATGAATG TTATTTGTTT TTCTAATTCT
TCTTTAAAAT TTCTCTGCTT ATGAATGCCA GAGTCCCAGC AAGCCTTATC
TCCC
SAV1_SE210 CTGCCAAGCT CCTGGTTCTG GTCATGAATG TTATTTGTTT TTCTAATTCC
TCTCTAAAAT TGCCCTGCTT ATGAATGCCA GAGTCCCAGC AAGCCTTATC
TCCC

1 1 1 1 1
5 107

ASIAN2_Ema TTGTTTTATT TTATATTTAC CTTCAAAACA CATTGGCAAG TGTTTTCCCA
TAAGAACGTG TTTGAATGAC GGGTGTATAA AAGCAGTAGC TTGAAATTTC
TTTTTAA
FOR_DS1535 TTGTTTTATT TTATATTTAC CTTCAAAACA CATTGGCAAG TGTTTTCCCA
TAAGAACGTG TTTGAATGAC GGGTGTATAA WAGCAGTAGC TTGAAATTTC
TTTTTAA
MAM1_SP134 TTGTTTTATT TTATATTTAC CTTCAAAACA CATTGGCAAG TGTTTTCCCA
TAAGAACGTG TTTGAATGAC GGGTGTATAA AAGCAGTAGC TTGAAATTTC
TTTTTAA
MAST_A278_ TTGTTTTATT TTATATTTAC CTTCAAA-CA TTTTGGCAAG TGTTTTCCCA
TACGAACATG TTTGAATGAC GGGTGTATAA AAGCAGTAGC TTGAAATTTC
TTTTTAA
SAV1_SE210 TTGTTTTATT TTATATTTAC CTTCAAAACA CATTGGCAAG TGTTTTCCCA
TAAGAACGTG TTTGAATGAC GGGTGTATAA TAGCAGTAGC TTGAAATTTC
TTTTTAA

1 1 1 1 1

5 91

```
ASIAN2_Ema   CTGTGGCTTC AGTTTAAGAT GGGAAGGTGT GGCCTGCACA GTCATACCAG
             TGAGTGGATG CCTGTGGGAG GAGGCAGCAG TTGGAAAGGA C
FOR_DS1535   CTGTGGCTTC AGTTTAAGAT GGGAAGGTGT GGCCTGCACA GTCATACCAG
             TGAGTGGATG CCTGTGGGAG GAGGCAGCAG TTGGAAAGGA C
MAM1_SP134   CTGTGGCTTC AGTTTAAGAT GGGAAGGTGT GGCCTGCACA GTCATACCAG
             TGAGTGGACG CCTGTGGGAG GAGGCAGCAG TTGGAAAGGA C
MAST_A385_   CTGTGGCTTC AGTTTAAGAT GGGAAGGTGT AGACTGCACA GTCATACCAG
             TGAGTGGATG CCTGTGGGAG GAGACAGCAT TTGGAAAGGA C
SAV1_SE210   CTGTGGCTTC AGTTTAAGAT GGGAAGGTGT GGCCTGCACA GTCATACCAG
             TGAGTGGATG CCTGTGGGAG GAGGCAGCAG TTGGAAAGGA C
```

 1 1 1 1 1
5 99

```
ASIAN2_Ema   CCTGGAATTC AGTGTCTTTC TCAGCTGTTT TGGCCTGACC TCCCAGCTGG
             CTGGGGTCAG CGCCCCCCGC TGCTTCAGGG ACCCCATCCT GGAGCCTCT
FOR_DS1535   CCTGGAATTC AGTGTCTTTC TCAGCTGTTT TGGCCTGACC TCCCAGCTGG
             CTGGGGTCAG CGCCCCCCGC TGCTTCAGGG ACCCCATCCT GGAGCCTCT
MAM1_SP134   CCTGGAATTC AGTGTCTTTC TCAGCTGTTT TGGCCTGACC TCCCAGCTGG
             CTGGGGTCAG CGCCCCCCGC TGCTTCAGGG ACCCCATCCT GGAGCCTCT
MAST_A279_   TCTGGAATTC AGTGTCTTTC TCAGCTGTTT TGGCCTGACT TCCCAGCTGG
             CTGGGGTCAG CGCCCCCCGC TGCTTCAGGG ATCCCATCCT GGAGCCTCT
SAV1_SE210   CCTGGAATTC AGTGTCTTTC TCAGCTGTTT TGGCCTGACC TCCCAGCTGG
             CTGGGGTCAG CGCCCCCCGC TGCTTCAGGG ACCCCCTCCT GGAGCCTCT
```

 1 1 1 1 1
5 112

```
ASIAN2_Ema   CTTACTTCTG GGTTCTTTCC AAAAGAGACT GTTGAGATAA AACACCACTG
             AAATTCACAG CAGCCAGAAC GTCAACTGTT TTCTTTGAGC TTTAGGAGCT
             TTCCATTCTC CA
FOR_DS1535   CTTACTTCTG GGTTCTTTCC AAAAGAGACT GTTGAGATAA AACACTATTG
             AAATTCACAG CAGCCAGAAC GTCAACTGTT TTCTTTGAGC TTTAGGAGCT
             TTCCATTCTC CA
MAM1_SP134   CTTACTTCTG GGTTCTTTCC AAAAGAGACT GTTGAGATAA AACACCATTG
             AAATTCACAG CAGCCAGAAC GTCAACTGTT TTCTTTGAGC TTTAGGAGCT
             TTCCATTCTC CA
MAST_A386_   TTTACTTCTG GGTTCTTTCC AAAAGAGACT GTTGAGATAA AACACCATTG
             AAATTCACAG CAGCCAGAAC GTCAACTGTT TTCTTTGAGC TTTAGGAGCT
             TTCCATTCTC CA
SAV1_SE210   CTTACTTCTG GGTTCTTTCC AAAAGAGACT GTTGAGATAA AACACCATTG
             AAATTCACAG CAGCCAGAAC GTCAACTGTT TTCTTTGAGC TTTAGGAGCT
             TTCCATTCTC CA
```

 1 1 1 1 1
5 99

```
ASIAN2_Ema   AAGACATGAG TCAATATGGA AT-AGGACTG AGGTCAGGTT GAAAAAAAAA
             --GTGAGAGA GTTCTGGATT TCTTTATGGA TGGGGCACAA GTGTCCGTA
FOR_DS1535   AAGACATGAG TCAATATGGA ATTAGGACTG AGGTCAGGTT GAAAAAAAA-
             --GTGAGAGA GTTCTGGATT TCTTTATGGA TGGGGCACAA GTGTCCGTA
MAM1_SP134   AAGACATGAG TCAATATGGA ATTAGGACTG AGGTCAGGTT GAAAAAAAAA
             --GTGAGAGA GTTCTGGATT TCTTTATGGA TGGGGCACAA GTGTCCGTA
MAST_A387_   AAGACATGAG TCAATATGGA ATTAGGACTG AGGTCAAGTT GAAAAAAAAA
             AAGTGAGAGA GTTCTGGATT TCTTTATGGA TGGGGCACAA GTGTCCCTA
SAV1_SE210   AAGACATGAG TCAATATGGA ATTAGGACTG AGGTCAGGTT GAAAAAAAAA
             ---------- GTTCTGGATT TCTTTATGGA TGGGGCACAA GTGTCCGTA
```

 1 1 1 1 1
5 91

```
ASIAN2_Ema   TGGAGACTGA ATGCCATTTG AGGTCAAGAC CCAAATTAGA AACTCTACCC
             AGGAATATTA AGCTAGAGCT GGGGTATGGG GATAAGGGCA T
FOR_DS1535   TGGAGACTGA ATGCCATTTG AGGTCAAGAC CCAAATTAGA AACTCTACCC
             AGGAATATTA AGCTAGAGCT GGGGTATGGR GATAAGGGCA T
MAM1_SP134   TGGAGACTGA ATGCCATTTG AGGTCAAGAC CCAAATTAGA AACTCTACCC
             AGGAATATTA AGCTAGAGCT GGGGTATGGG GATAAGGGCA T
MAST_A388_   TGGAGACTGA ATGCCATTTG AGGTCAAGAC CCAAATTAGA AACTCTACCC
             AAGAATATTA AGCTAGAGCT GGGGTATGAA GATAAGGGCA C
```

```
SAV1_SE210   TGGAGACTGA ATGCCATTTG AGGTCAAGAC CCAAATTAGA AACTCTACCC
             AGGAATATTA AGCTAGAGCT GGGGTATGGG GATAAGGGCA T

 1 1 1 1 1
5 98

ASIAN2_Ema   TGCATGTCGT ATGATTTTTT GAGAGCCCAT AAGCCCCAAA GAAAGGGTCA
             GAGATGCCAG TGAATGTTAG AGCTGGTGAC CTGGAGCAGG TGCTTACA
FOR_DS1535   TGCATGTCGT ATGATTTTTT GAGAGCCCAT AAGCCCCAAA GAAAGGGTCA
             GAGATGCCAG TGAATGTTAG AGCTGGTGAC CTGGAGCAGG TGCTTACT
MAM1_SP134   TGCATGTCGT ATGATTTTTT GAGAGCCCAT AAGCCCCAAA GAAAGGGTCA
             GAGATGCCAG TGAATGTTAG AGCTGGKGAC CTGGAGCAGG TGCTTACA
MAST_A443_   TGCATGTTGT ATGATTTTTT GAGAGCCCAT AAGCCCCAAA GAAAGGGTCA
             GAGATGCCAG CGAACGTTAG AGCTGGTGAC CTGGAGCAGG AGCTTACA
SAV1_SE210   TGCATGTCGT ATGATTTTTT GAGAGCCCAT AAGCCCCAAA GAAAGGGTCA
             GAGATGCCAG TGAATGTTAG AGCTGGTGAC CTGGAGCAGG TGCTTACA

 1 1 1 1 1
5 107

ASIAN2_Ema   CCAAACGCAT T-GCC-GTTG AGTCAATT-C CGACTCAGTG ACCCTATAAA
             AACCGAGGGG AACTGCCCCA TAGGGTTTCT GAGGAAC-AG CTGTTGGATT
             CCAACTG
FOR_DS1535   CCAAACGCAT T-GCC-GTTG AGTCAATT-C CGACTCAGTG ACCCTATAAA
             AACAGAGGGG AACTGCCCCA TAGGGTTTCT GAGGAAC-AG CTGTTGGATT
             CCAACTG
MAM1_SP134   CCAAACGCAT T-GCC-GTTG AGTCAATT-C CGACTCAGTG ACCCTATAAA
             AACAGAGGGG AACTGCCCCA TAGGGTTTCT GAGGAAC-AG CTGTTGGATT
             CCAACTG
MAST_A347_   CCAAACGCAT TTGTCCGTTG AGTCAATTTC CGACTCAGTG ACCCTATAAA
             AACAGAGGGG AACTGCCCGA TAGGGTTTCT GAGGAACTAG CTGTTGGATT
             CCAACTA
SAV1_SE210   CCAAACGCAT T-GTC-GTTG AGTCAATT-C CGACTCAGTG ACCCTATAAA
             AACAGAGGGG AACTGCCCCA TAGGGTTTCT GAGGAAC-AG CTGTTGGATT
             CCAACTG

 1 1 1 1 1
5 107

ASIAN2_Ema   CGCAAGGGCG CACCCAGCAT GGAGCAGCTC ACCAGGAAGA TGGCTTCGAT
             GTGGCCGATA CCCCGACGCA GGGAGGAACC CAGGCGGTCC CCGACCCCAG
             CCAACAG
FOR_DS1535   CGCAAGGGCG CACCCAGCAT GGAGCAGCTC ACCAGGAAGA TGGCTTCGAT
             GTGGCCGATA CCCCGACGCA GGGAGGAACC CAGGCGGTCC CCGACCCCAG
             CCAACAG
MAM1_SP134   CGCAAGGGCG CACCCAGCAT GGAGCAGCTC ACCAGGAAGA TGGCTTCGAT
             GTGGCCGATA CCCCGACGCA GGGAGGAACC CAGGCGGTCC CCGACCCCAG
             CCAACAG
MAST_A390_   CGCAAGGGCG CACCCAGCAT GGAGCAACTC ACCAGGAAGA TGGCTTCGAT
             GTGGCCGATA CCCCGACGCA GGGAGGAACC CAGGCGGTCC CCGACCCCAG
             CCAACAG
SAV1_SE210   CGCAAGGGCG CACCCAGCAT GGAGCAGCTC ACCAGGAAGA TGGCTTCGAT
             GTGGCCGATA CCCCGACGCA GGGAGGAACC CAGGCGGTCC CCGACCCCAG
             CCAACAG

 1 1 1 1 1
5 108

ASIAN2_Ema   CTCAAACTAA AATGAAATAA TGTTCT-GGA AACATCAGAA CTCATGTTCC
             CATGAGTCCC CATAAAAATC ACCACACCCG CTGAAATCGT AACCATGTGC
             TGTTTGCA
FOR_DS1535   CTCAAACTAA AATGAAATAA TGTTCT-GGA AACATCAGAA CTCATGTTCC
             CATGAGTCTC CATAAAAATC ACCACACCCG CTGAAATCGT AACCGTGTGC
             TGTTTGCA
MAM1_SP134   CTCAAACTAA AATGAAATAA TGTTCT-GGA AACATCAGAA CTCATGTTCC
             CATGAGTCCC CATAAAAATC ACCACACCCG CTGAAATCGT AACCGTGTGC
             TGTTTGCA
MAST_A282_   -TCAAACTAA AATGAAATAA TGTTTTTGGA AACATCAGAA CTCGTGTTCC
             CATGAGTCCC CATAAAAATC ACCACACCCG CTGAAATCGT AACCGTGTGC
             TGTTTACA
SAV1_SE210   CTCAAACTAA AATGAAATAA TGTTCT-GGA AACATCAGAA CTCATGTTCC
```

CATGAGTCCC CATAAAAATC ACCACACCCG CTGAAATCGT AACCGTGTGC
TGTTTGCA

1 1 1 1 1
5 93

ASIAN2_Ema AACTTAAAAC GTCCTTCTAA CAGCTTCTTC TTTTGAAATG GAGATGTTGT
TGCTAGGTTC CTCTGAGTCA GTTCCCACTC ATAGTGACCA TGA
FOR_DS1535 AAYTTAARAC GTCCTTCTAA CAGCTTCTTC TTTTGAAATG GAGATGTTGT
TGCTAGGTCC CTCTGAGTCA GTTCCCACTC ATAGTGACCA TGA
MAM1_SP134 AACTTAAAAC GTCCTTCTAA CAGCTTCTTC TTTTGAAATG GAGATGTTGT
TGCTAGGTTC CTCTGGGTCA GTTCCCACTC ATAGTGACCA TGA
MAST_A393_ AACTTAAAAC GTTTTTTTAA TAGCTTCTTC TTTTGAAATG GAGATGTTGT
TGCTAGGTTC CTCTGAGTCA GTTCCTACTC ATAGTGACCA TGA
SAV1_SE210 AACTTAAAAC GTCCTTCTAA CAGCTTCTTC TTTTGAAATG GAGATGTTGT
TGCTAGGTTC CTCTGGGTCA GTTCCCACTC ATAGTGACCA TGA

1 1 1 1 1
5 93

ASIAN2_Ema CTGGGAACTC TGGGAGTCTT TCCTCAGAGG TGGTGGCAGT GAAAACAGAG
CAGGAGGGCG GAGTCACCTG CTCCCCACAT TCAGCTCACA GGG
FOR_DS1535 CTGGGAACTC TGGGAGTCTT TCCTCAGAGG TGGTGGCAGT GAAAACAGAG
CAGGAGGGCG GAGTCACCTG CTCCYCACAT TCAGCTCACA GGG
MAM1_SP134 CTGGGAACTC TGGGAGTCTT TCCTCAGAGG TGGTGGCAGT GAAAACAGAG
CAGGAGGGCG GAGTCACCTG CTCCCCACAT TCAGCTCACA GGG
MAST_A283_ TTGGGAACTC TGGGAGTCTT TCCTCAGAGG TGGTGGCAGT GAAAACAGAG
CAGGAGGGCG GAGTCACCTG CTCCCCATAT TCAGCTCACA GGG
SAV1_SE210 CTGGGAACTC TGGGAGTCTT TCCTCAGAGG TGGTGGCAGT GAAAACAGAG
CAGGAGGGCG GAGTCACCTG CTCCCCACAT TCAGCTCACA GGG

1 1 1 1 1
5 97

ASIAN2_Ema ATGAAATTTT TGATGCATAT GGATTTTTCT ATTTGCATCC TTGGG-TGAT
GATATAACTT CTGCTAACCA ACTACCTGAG ACAGCTCTGG ACACAGG
FOR_DS1535 ATGAAATTTT TGATGCATAT GGATTTTTCT ATTTGCATCC TTGGG-TGAT
GATATAACTT CTGCTAACCA ACTACCTGAG ACAGCTCTGG ACACAGG
MAM1_SP134 ATGAAATTTT TGATGCATAT GGATTTTTCT ATTTGCATCC TTGGG-TGAT
GATATAACTT CTGCTAACCA ACTACCTGAG ACAGCTCTGG ACACAGG
MAST_A285_ ATGAAATTTT TGATGCATAT GGATTTTTCT ATTTGCATCC TTGGGGTGAT
GATATAACTT CTGCTAACCA ACTACCTGAG ACAGCTCTGG ACACAGG
SAV1_SE210 ATGAAATTTT TGATGCATAT GGATTTTTCT ATTTGCATCC TTGGG-TGAT
GATATAACTT CTGCTAACCA ACTACCTGAG ACAGCTCTGG ACACAGG

1 1 1 1 1
5 100
ASIAN2_Ema CTAGAGTCTG TAGTCTTTAT AAATAAAATC CAAATAACAC CTTAGCTGAA
TATAATTTTC CAGCTTATTG CTGTTTTGAC ACAAATGAAA CCGAGATATA
FOR_DS1535 CTAGAGTCTG TAGTCTTTAT AAATAAAATC CAAATAACAC CTTAGCTGAA
TATAATTTTC CAGCTTAAGG CTGTTTTGAC ACAAATGAAA CCGAGATATA
MAM1_SP134 CTAGAGTCTG TAGTCTTTAT AAATAAAATC CAAATAACAC CTTAGCTGAA
TATAATTTTC CAGCTTATTG CTGTTTTGAC ACAAATGAAA CCGAGATATA
MAST_A445_ CTAGAGTCTG TAGCCTTTAT AAATAAAAAC CAAATAACAC CTTAGCTGAA
TATAATTTTC CAGCTTATTG CTGTTTTGAC ACAAATGAAA CCGAGATATA
SAV1_SE210 CTAGAGTCTG TAGTCTTTAT AAATAAAATC CAAATAACAC CTTAGCTGAA
TATAATTTTC CAGCTTATTG CTGTTTTGAC ACAAATGAAA CCGAGATATA

1 1 1 1 1
5 93

ASIAN2_Ema ACTTT-GTGA AAATTCTTCC TCTCTCTGCC CACAGCCACC GAGTTCACTC
CGCAGCAGGG TTAATGGCTG TTTCCATATC CCTCCAGAGA CAC
FOR_DS1535 ACTTT-GTGA AAATTCTTCC TCTCTCTGCC CACAGCCACC GAGTTCACTC
CRCAGCAGGG TTAATGGCTG TTTCCATATC CCTCCAGAGA CAC
MAM1_SP134 ACTTT-GTGA AAATTCTTCC TCTCTCTGCC CACAGCCACC GAGTTCACTC
CGCAGCAGGG TTAATGGCTG TTTCCATATC CCTCCAGAGA CAC
MAST_A396_ ACTTTTGTGA AAATTCTTCC TCTCTCTGCC CACAGCCACC GAGTTCACTC
CGCAGCAGGG TTAATGGCTG TTTCCACATC CCTCCAAAGA CAC
SAV1_SE210 ACTTT-GTGA AAATTCTTCC TCTCTCTGCC CACAGCCACC GAGTTCACTC
CGCAGCAGGG TAAATGGCTG TTTCCATATC CCTCCAGAGA CAC

1 1 1 1 1
5 122

```
ASIAN2_Ema   CCCACAGTCA CGCGTGGGGA GAAATAAGTC GAGAAGGCGG CGGTGTCAGG
             GGAAGTGGGA AGTCGCCCGC CAGCGTGGGC CGTTCTCTGA AAGCCCAGCA
             CTCTCCCCCC CGCCCAGAAG CA
FOR_DS1535   CCCACAGTCA CGCGTGGGGA GAAATAAGTC GAGAAGGCGG CGGTGTCAGG
             GGAAGTGGGA AGTCGCCCGC CAGCGTGGGC CGTTCTCTGA AAGCCCAGCA
             CTCTCCCCCC CGCCCAGAAG CA
MAM1_SP134   CCCACAGTCA CGCGTGGGGA GAAATAAGTC GAGAAGGCGG CGGTGTCAGG
             GGAAGTGGGA AGTCGCCCGC CAGCGTGGGC CGTTCTCTGA AAGCCCAGCA
             CTCTCCCCCC -GCCCAGAAG CA
MAST_A398_   CCCACAGTCA CGCGTGGGGA GAAAGAAGTC GAGAAGGCGG CGGTGTCAGG
             GGAAGT---- ---CGCCCGC CAGCGTGGGC CGTTCTCTGA AAGCCCAGCA
             CTCTCCCCCC CGCCCAGAAG CA
SAV1_SE210   CCCACAGTCA CGCGTGGGGA GAAATAAGTC GAGAAGGCGG CGGTGTCAGG
             GGAAGTGGGA AGTCGCCCGC CAGCGTGGGC CGTTCTCTGA AAGCCCAGCA
             CTCTCCCCCC CGCCCAGAAG CA
```

1 1 1 1 1
5 99

```
ASIAN2_Ema   GGGCAAGGAC GAGAAGGCAG TCAGGGACAG GAAAGCTGGA AATGGGAAAC
             CCGAGGTCAA AAA-GGGGAA AGTGTTAACA ATCAGAACAT GGAATGTGT
FOR_DS1535   GGGCAAGGAC GAGAAGGCAG TCAGGGACAG GAAAGCTGGA AATGGGGAAC
             CCGAGGTCAA AAA-GGGGAA AGTGTTAACA ATCAGAACAT GGAATGTGT
MAM1_SP134   GGGCAAGGAC GAGAAGGCAG TCAGGGACAG GAAAGCTGGA AATGGGGAAC
             CCAAGGTCAA AAA-GGGGAA AGTGTTAACA ATCAGAACAT GGAATGTGT
MAST_A399_   GGGCAAGGAC GAGAAGGCAG TCAGGGGCAG GAAAGCTGGA AATGCGGAAC
             CCGAGGTCAA AAAAGGGGAA AGTGTTAACA ATCAGAACAT GGAATGTGT
SAV1_SE210   GGGCAAGGAC GAGAAGGCAG TCAGGGACAG GAAAGCTGGA AATGGGGAAC
             CCGAGGTCAA AAA-GGGGAA AGTGTTAACA ATCAGAACAT GGAATGTGT
```

1 1 1 1 1
5 105

```
ASIAN2_Ema   ACAGATTAGC AGGAGTGCTG GACCTCATTC AGAGATTGCC RTGTGGTAAG
             AGGAAGACTG TGTTCCCCCA -GGCGTGTTG TCGGTTTGGG GC-TGGGGTC
             ACACC
FOR_DS1535   ACAGATTAGC AGGAGTGCTG GACCTCATTC AGAGATTGCC GTGTGGTAAG
             AGGAAGACTG TGTTCCCCCA -GGCGTGTTG TCGGTTTGGG GC-TGGGGTC
             ACACC
MAM1_SP134   ACAGATTAGC AGGAGTGCTG AACCTCATTC AGAGATTGCC GTGTGGTAAG
             AGGAAGACTG TGTTCCCCCA -GGCGTGTTG TCGGTTTGGG GC-TGGGGTC
             ACACC
MAST_A401_   ACAGATTAGC AGAAGTGCTG GACCTCATTC AGAGATTGCC ATGTGGTAAG
             AGGAAGACTG TGTTCCCCCA CGGCGTGTTG TCGGTTTGGG GCGTGGGGTC
             ACACC
SAV1_SE210   ACAGATTAGC AGGAGTGCTG GACCTCATTC AGAGATTGCC GTGTGGTAAG
             AGGAAGACTC TGTTCCCCCA -GGCGTGTTG TCGGTTTGGG GC-TGGGGTC
             ACACC
```

1 1 1 1 1
5 109

```
ASIAN2_Ema   GCTGAAATGC AGCAAAGGAA TAAATTCTTA CTTGTGGGAA GAAATTTGTT
             TGTTGAAGTG ATGGTATTGA GGCGGTATAT CATT--TTCT TGTGGGA-AT
             TGTTCTTTT
FOR_DS1535   GCTGAAATGC AGCAAAGGAA TAAATTCTTA CTTGTGGGAA GAAATTTGTT
             TGTTGAAGTG ATGGTATTGA GGCGGTATAT CATT--TTCT TGTGGGA-AT
             TGTTCTTTT
MAM1_SP134   GCTGAAATGC AGCAAAGGAA TAAATTYTTA CTTGTGGGAA GAAATTTGTT
             TGTTGAAGTG ATGGTATTGA GGCGGTATAT CATT--TTCT TGTGGGA-AT
             TGTTCTTTT
MAST_A286_   GTTGAAATGC AGCAAAGGAA TAAATTCTTA CTTGTGGGAA GAAATTTGTT
             TGTTGAAGTG ATGGTATTGA GGCGGTATAT CATTCATATT TAAACGACAT
             TTTCCCGTT
SAV1_SE210   GCTGAAATGC AGCAAAGGAA TAAATTCTTA CTTGTGGGAA GAAATTTGTT
             TGTTGAAGTG ATGGTATTGA GGCGGTATAT CATT--TTCT TGTGGGA-AT
             TGTTCTTTT
```

 1 1 1 1 1
5 90

ASIAN2_Ema CTTCGCATGG GCATGACTGT GCACATGGAA TGTGCTGAAT CTGGTTTGAA
CTACCAAGAC CTGTACAAGT AACCTCAGCT TCTGGAGTGT
FOR_DS1535 CTTCGCATGG GCATGACTGT GCACATGGAA TGTGCTGAAT CTGGTTTGAA
CTACCAAGAC CTGTACAAGT AACCTCAGCT TCTGGAGTGT
MAM1_SP134 CTTCACATGG GCATGACTGT GCACATGGAA TGTGCTGAAT CTGGTTTGAA
CTACCAAGAC CTGTACAAGT AACCTCAGCT TCTGGAGTGT
MAST_A450_ CTTCGCATGG GCATGACTGT GCACATGGAA TGTGCTGAAT CTGGTTTGAA
CTACCAAGAC CTGTGCAAGT AACCTCAGCT TCTGGAGTGT
SAV1_SE210 CTTCGCATGG GCATGACTGT GCACATGGAA TGTGCTGAAT CTGGTTTGAA
CTACCAAGAC CTGTACAAGT AACCTCAGCT TCTGGAGTGT

 1 1 1 1 1
5 102

ASIAN2_Ema ACCAAAAGAC CTTTCCCAGA AGGCCAGAGG GGAGCCACCT ATGGGGGCAG
GGCAGATGGA ACCCTGCCCT GTGCAGTTGG AACAGTTTTT -GGATTTCCC
CA
FOR_DS1535 ACCAAAAGAC TTTTCC-AGA AGGCCAGAGG GGAGCCACCT ATGGGGGCAG
GGCAGATGGA ACCCTGCCCT GTGCAGTTGG AACAGTTTTT -GGATTTCCC
CA
MAM1_SP134 ACCAAAAGAC CTTTCCCAGA AGGCCAGAGG GGAGCCACCT ATGGGGGCAG
GGCAGATGGA ACCCTGCCCT GTGCAGTTGG AACAGTTTTT -GTATTTCCC
CA
MAST_A404_ ACCAAAAGAC TTTTCCCAGA AGGCCAGAGG GGAGCCACCT ATGGGGGCAG
GGCAGATGGA ACCCTGCCCT GTGCAGTTGG AACAGTTTTT -GGATTTCCC
CA
SAV1_SE210 ACCAAAAGAC CTTTCCCAGA AGGCCAGAGG GGAGCCACCT ATGGGGGCAG
GGCAGATGGA ACCCTGCCCT GTGCAGTTGG AACAGTTTTT TGGATTTCCC
CA

 1 1 1 1 1
5 109

ASIAN2_Ema CTTTCCCTTT -GCATTCACA ACTTGGCTAA CTGTTGGACG CAAGAGGCCT
AGTTTTTGGC CTGTCTTGGC TTTCGACATG CCTTCCTCAC TAAGCTTAAT
CATTTCTAG
FOR_DS1535 CTTTCCCTTT -GCATTCACA ACTTGGCTAA CTGTTGGACG CAAGAGGCCT
AGTTTTTGGC CTGTCTTGGC TTTCAACATG CCTTCCTCAC TAAGCTTAAT
CATTTCTAG
MAM1_SP134 CTTTCCCTTT -GCATTCACA ACTTGGCTAA CTGTTGGACG CAAGAGGCCT
AGTTTTTGGC CTGTCTTGGC TTTCGACATG CCTTCCTCAC TAAGCTTAAT
CATTTCTAG
MAST_A451_ CTTTTCTTTT TGCGTTCATA ACTTGGCTAA CTGTTGGGTG CAAGAGGCCT
AGCTTTTGGC CTGTCTTGGC TTTCAACTTG CCTCCCTCAC TAAGCTTAAT
CATTTCTAG
SAV1_SE210 CTTTCCCTTT -GCATTCACA ACTTGGCTAA CTGTTGGACG CAAGAGGCCT
AGTTTTTGGC CTGTCTTGGC TTTCAACATG CCTTCCTCAC TAAGCTTAAT
CATTTCTAG

 1 1 1 1 1
5 106

ASIAN2_Ema TTTCTCCTGA AGCCGATGCC CTGAATTTGG ATTTGCAACC TACTAGACTG
TGAGATAAAA A-TTTCTCTT TGTTAAAGCC ATCCAATTGT GGTATTTCTG
TTATGG
FOR_DS1535 TTTCTCCTGA AGSCGATGCC CTGAATTTGG ATTTGCAACC TACTAGACTG
TGAGATAAAA AATTTCTCTT TGTTAAAGCC ATCCAATTGT GGTATTTCTG
TTATGG
MAM1_SP134 TTTCTCCTGA AGCCGATGCC CTGAATTTGG ATTTGCAACC TACTAGACTG
TGAGATAAAA AATTTCTCTT TGTTAAAGCC ATCCAATTGT GGTATTTCTG
TTATGG
MAST_A405_ TTTCTCCTGA AGCCGATGCC CTGAATTTGG ATTTGCAACC TACTAGACTG
TGAGATAAAA AATTTCTCTA TGTTAAAGCC ATCCAATTGT GGTATTTCTG
TTATGG
SAV1_SE210 TTTCTCCTGA AGGCGATGCC CTGAATTTGG ATTTGCAACC TACTAGACTG
TGAGATAAAA AATTTCTCTT TGTTAAAGCC ATCCAATTGT GGTATTTCTG
TTATGG

```
 1 1 1 1 1
5 109

ASIAN2_Ema   AATAAGGGCT TGAAGCCACA TTATTGAATA TCCCAACCCA CCATAAAGGT
             TGGGCGCTTC TCAAATTGCA GGCCTCTCCT CTTGCAAGTC GCAAAATAAA
             CTGGCACAT
FOR_DS1535   AATAAGGGCT TGAAGCCACA TTATTGAATA TCCCAACCCA CCATAAAGGT
             TGGGCGCTTC TCAAATTGCA GGCCTCTCCT CTTGCAAGTC GCAAAATAAA
             CTGGCACAT
MAM1_SP134   AATAAGGGCT TGAAGCCACA TTATTGAATA TCCCAACCCA CCATAAAGGT
             TGGGCGCTTC TCAAATTGCA GGCCTCTCCT CTTGCAAGTC GCAAAATAAA
             CTGGCACAT
MAST_A454_   AATAAGGGCT TGAAGCCACA TTATTGAATA TCCCAACCCA CCATAAAGTT
             TGGGCGCTTC TCAAATTGCA GGCCTCTCCT CTTGTAAGTC ACAAAATAAA
             CTGCGACAA
SAV1_SE210   AATAAGGGCT TGAAGCCACA TTATTGAATA TCCCAACCCA CCATAAAGGT
             TGGGCGCTTC TCAAATTGCA GGCCTCTCCT CTTGCAAGTC ACAAAATAAA
             CTGGCACAT

 1 1 1 1 1
5 93

ASIAN2_Ema   CCATGCCCCT GGGCCCCTTG CAGCTACTAC ATCTGCTGCG GAAGTTTACA
             GACCTCTGCT GCTGCCTCCC GGGGCTGCAG CTGCCCGATG CAC
FOR_DS1535   CCATGCCCCT GGGCCCCTTG CAGCTACTAC ATCTGCTGCG GAAGTTTACA
             GACCTCTGCT GCTGCCTCCC GGGGCTGCAG CTGCCCGATG CAC
MAM1_SP134   CCATGCCCCT GGGCCCCTTG CAGCTACTAC ATCTGCTGCG GAAGTTTACA
             GACCTCTGCT GCTGCCTCCC GGGGCTGCAG CTGCCTGATG CAT
MAST_A407_   CCAAATCCCA GGCCCC-TTA CAGCCACTAC ATCTGCTGCA GAAGTTTACA
             GACCTCTGCT GCTGCCTCCC GGGGCTGCAG CTGCCCGACG CGC
SAV1_SE210   CCATGCCCCT GGGCCCCTTG CAGCTACTAC ATCTGCTGCG GAAGTTTACA
             GACCTCTGCT GCTGCCTCCC GGGGCTGCAG CTGCCCGATG CGC

 1 1 1 1 1
5 93

ASIAN2_Ema   CCTTCAGGAG TGAAACCTCA GAGTTGTTTT TGACCCCTCG CTGCCCCAGA
             CTCCAGGCCC CCGCGTCTCT GAGTCCTGGT TGTGCTGCAT CTT
FOR_DS1535   CCTTCAGGAG TGAAACCTCA GAGTTGTTTT TGACCCCTCG CTGCCCCAGA
             CTCCAGGCCC CCGCGTCTCT GAGTCCTGGT TGTGCTGCAT CTT
MAM1_SP134   CCTTCAGGAG TGAAACCTCA GAGTTGTTTT TGACCCCTCG CTGCCCCAGA
             CTCCAGGCCC CCGCGTCTCT GAGTCCTGCT TGTGCTGCAT CTT
MAST_A409_   CCTTCAGGAG TGAAACCTCA GAGTTGTTTT TGACCCCTCG CTGCCCCAGA
             CTCCAGGCCC CCGCGTCTCT GAGTCCTGAT TGTGCTGCCT CTT
SAV1_SE210   CCTTCAGGAG TGAAACCTCA GAGTTGTTTT TGACCCCTCG CTGCCCCAGA
             CTCCAGGCCC CCGCGTCTCT GAGTCCTGGT TGTGCTGCAT CTT

 1 1 1 1 1
5 91

ASIAN2_Ema   GCTAGTCAGT TGATTGGCTA TGTGAATGTT GGGGCAGCTG GTCAGTAAGG
             TGGCTATGTG AGCATGTCTT CAAGTGCAGA TTGAGAAAAT A
FOR_DS1535   GCTAGTCAGT TGATTGGCTA TGTGAATGTT GGGGCAGCTG GTCAGTAAGG
             TGGCTATGTG AGCATGTCTT CAAGTGCAGA TTGAGAAAAT A
MAM1_SP134   GCTAGTCAGT TGATTGGCTA TGTGAATGTT GGGGCAGCTG GTCAGTAAGG
             TGGCTATGTG AGCATGTCTT CAAGTGCAGA TTGAGAAAAT A
MAST_A456_   GCTAGTTAGT TGATTGGCTA TGTGAATGTT GGGGCAGCTG GTCAGTAAGG
             TGGCTGTGTG AGCATGTCTT CAAGTGCAGA TTGAGAAAAT A
SAV1_SE210   GCTAGTCAGT TGATTGGCTA TGTGAATGTG GGGGCAGCTG GTCAGTAAGG
             TGGCTATGTG AGTATGTCTT CAAGTGCAGA TTGAGAAAAT A

 1 1 1 1 1
5 95

ASIAN2_Ema   CTGAATCTAC ATGCCTCTTG TTTTATTTCT CTTTAAACTT GAAGAACAGT
             CAATCTGCAG ATACTGTGAG GTACAGATTA TACTGTCGGC TACTG
FOR_DS1535   CTGAATCTAC ATGCCTCTTG TTTTATTTCT CTTTAAACTT GAAGAACAGT
             CAATCTGCAG ATACTGTGAG GTACAGATTA TACTGTCGGC TACTG
MAM1_SP134   CTGAATCTAC ATGCCTCTTG TTTTATTTCT CTTTAAACTT GAAGAACAGT
             CAATCTGCAG ATACTGTGAG GTACAGATTA TACTGTCGGC TACTG
```

```
MAST_A289_    TTGAATCTAC ATGCCTCTTG TTTTATTTCT CTTTAAACTT GAAGAACAGT
              CAATCTGCAG ATACTGTGAG GTACAGATTA TACTGTCGGC TACTG
SAV1_SE210    CTGAATCTAC ATGCCTCTTG TTTTATTTCT CTTTAAACTT GAAGAACAGT
              CAATCTGCAG ATACTGTGAG GTACAGATTA TACTGTCGGC TACTG

 1 1 1 1 1
5 92

ASIAN2_Ema    GAGGTTTCTT CCTGCTGCCT CAGTGTGTGG AATGGGGCTA GAGGCTGTAG
              CTAGGCCACC CACCCACAAT GCTCTGACCA CGTTGCCTTC TT
FOR_DS1535    GAGGTTTCTT CCTGCTGCCT CAGTGTGTGG AACGGGGCTA GAGGCTGTAG
              CTAGGCCACC CACCCACAAT GCTCTGACCA CGTTGCCTTC TT
MAM1_SP134    GAGGTTTCTT CCTGCTGCCT CAGTGTGTGG AATGGGGCTA GAGGCTGTAG
              CTAGGCCACC CACCCACAAT GCTCTGACCA CGTTGCCTTC TT
MAST_A410_    GAGGTTTCTT CCTGCTGCCT CAGTGTGTGG AATGGGGCTA GAGGCTGTAG
              CTAGGCCACC CACCCACAAT GCTCTGACCA CGTTGCCTTC TT
SAV1_SE210    GAGGTTTCTT CCTGCTGCCT CAGTGTGTGG AACAGGGCTA GAGGCTGTAG
              CTAGGCCACC CACCCACAAT GCTCTGACCA CGTTGCCTTC TT

 1 1 1 1 1
5 107

ASIAN2_Ema    AGCTTTGTGG AAGGAAGACC AGTCAGAAGT AGGCCTACCC TGGCAGAGAG
              GAACCTGGAG GGGAGTGGGG ACTAGGTGAA CACCAAAAAA GGTTCAGCTC
              TCAGTCA
FOR_DS1535    AGCTTTGTGG AAGGAAGACC AGTCAGAAGT AGGCCTACCC TGGCAGAGAG
              GAACCTGGAG GGGAGTGGGG ACTAGGTGAA CACCAAAAAA GGTTCAGCTC
              TCGGTCA
MAM1_SP134    AGCTTTGTGG AAGGAAGACC AGTCAGAAGT AGGCCTACCC TGGCAGAGAG
              GAACCTGGAG GGGAGTGGGG ACTAGGTGAA CACCAAAAAA GGTTCAGCTC
              TCAGTCA
MAST_A412_    AGCTTTGTGG AAGGAAGACC AGTCAGAAGT AGGCCTACCC TGGCAGAGAG
              GAACCTGGAG GG-AGTGGG- ACTAGGTGAA CACCAAAAAA GGTTCAGCTC
              TCAGTCA
SAV1_SE210    AGCTTTGTGG AAGGAAGACC AGTCAGAAGT AGGCCTACCC TGGCAGAGAG
              GAACCTGGAG GGGAGTGGGG ACTAGGTGAA CACCAAAAAA GGTTCAGCTC
              TCGGTCA

 1 1 1 1 1
5 101

ASIAN2_Ema    AATTTTATTT GCCAGATAAT AGTAACACGG GTCTTAAAAC TTAAACAGTT
              TTGAGCGACG ATGTCTTAGC CACATCACCA CTCCAATATT TAGCTGTTTT
              T
FOR_DS1535    AATTTTATTT GCCAGATAAT AGTAACACGG GTCTTAAAAC TTAAACAGTT
              TTGAGCGACA ATGTCTTAGC CACATCATCA CTCCAATATT TAGCTGTTTT
              T
MAM1_SP134    AATTTTATTT GCCAGATAAT AGTAACACGG GTCTTAAAAC TTAAACAGTT
              TTGAGCGACG ATGTCTTAGC CACATCACCA CTCCAATATT TAGCTGTTTT
              T
MAST_A414_    AATTTTATTT GCCAGATAAT AGTAACACAG GTCTTAAAAC TTAAACAGTT
              TTGAGCGACG ATGTCTTACC CACATCACCT CTCCAATATT TAGCTATTTT
              T
SAV1_SE210    AATTTTATTT GCCAGATAAT AGTAACACGG GTCTTAAAAC TTAAACAGTT
              TTGAGCGACG ATGTCTTAGC CACATCACCA CTCCAATATT TAGCTGTTTT
              T

 1 1 1 1 1
5 114

ASIAN2_Ema    AAAAGAGGTG TGGGCTACCC TTCCAGAAAA -GAGTGAACA GAAATGCAAA
              CAATTTCCAG AGTGTCTGGG CATGTGGCCA TGCTTGGCAC TCCACAGTTT
              ACAGTTAGTT CCCT
FOR_DS1535    AAAAGAGGTG TGGGCTACCC TTCCAGAAAA -GAGTGAACA GAAATGCAAA
              CAATTTCCAG AGTGTCTGGG CATGTGGCCA TGCTTGGCAC TCCACAGTTT
              ACAGTTAGTT CCCT
MAM1_SP134    AAAAGAGGTG TGGGCTACCC TTCCAGAAAA -GAGTGAACA GAAATGCAAA
              CAATTTCCAG AGTGTCTGGG CATGTGGCCA TGCTTGGCAC TCCACAGTTT
              ACAGTTAGTT CCCT
MAST_A291_    AAAAGAGGTG TGGGCTACCC TTCCAAAAAA AGAGTGAACA GAAATGCAAA
              CAATTTCCAG AGTGTCTGGG CATGTGGCCA TGCTTGGCAC TCCAC-GTTT
```

```
             ACAGTTAGTT CCCC
SAV1_SE210   AAAAGAGGTG TGGGCTACCC TTCCAGAAAA -GAGTGAACA GAAATGCAAA
             CAATTTCCAG AGTGTCTGGG CATGTGGCCA TGCTTGGCAC TCCACAGTTT
             ACAGTTAGTT CCCT

 1 1 1 1 1
5 162

ASIAN2_Ema   GGGAA-GTCT GGTGGGAGAA AAGTGGGGCA GGGGTGGGCA GGAGGAACAC
             CACATTCTCA GGCCTAACA- CACATAACA- CAAATCCAAA AA--TC-AAA
             CACGTTGCTG TCAAGTCAGC CCT-CAA--- ---------C TTATGGC-GA
             CCCCAGGCAC TA
FOR_DS1535   GGGAA-GTCT GGTGGGAGAA AAGTGGGGCA GGGGTGGGCA GGAGGAACAC
             CACATTCTCA GGCCTAACA- CACATAACA- CAAATCCAAA AA--TC-AAA
             CACGTTGCTG TCAAGTCAGC CCT-CAA--- ---------C TTATGGC-GA
             CCCCAGGCAC TA
MAM1_SP134   GGGGA-GTCT GGTGGGAGAA AAGTGGGGCA GGGGTGGGCA GGAGGAACAC
             CACATTCTCA GGCCTAACA- CACATAACA- CAAATCCAAA AA--TC-AAA
             CACGTTGCTG TCAAGTCAGC CCT-CAA--- ---------C TTATGGC-GA
             CCCCAGGCAC TA
MAST_B003_   GGGAAGGTCT GGTGGGAGAA AAGTGGGGCA GGGGTGGGCA GGAGGAACAC
             CACATTCTCA GGCCTAACAC CACATAACAC CAGATCCAAA AAACTCAAAA
             CACATTGCTG TCAAGTCA-C CTTAGAAGAG TGTTCCTGTC TTTTCACTGA
             CTCTCTACCT TA
SAV1_SE210   GGGAA-GTCT GGTGGGAGAA AAGTGGGGCA GGGGTGGGCA GGAGGAACAT
             CACATTCTCA GGCCTAACA- CACATAACA- CAAATCCAAA AA--TC-AAA
             CACGTTGCTG TCAAGTCAGC CCT-CAA--- ---------C TTATGGC-GA
             CCCCAGGCAC TA

 1 1 1 1 1
5 148

ASIAN2_Ema   AGGCCCATTT GCCTCCTTAC TGAGGAYGGC AGTGGTCCAG TGGCAGAATT
             CTCGTCCTCT AGCAGGAGAC CCAGGTTGTA TTCCTGGCTA ACGCACCTCC
             AGGGCAGCTG CTACCCGCTT GTCAGTGGTA GCTTGCGTGT TGCTATGA
FOR_DS1535   AGGCCCATTT GCCTCCTTAC TGAGGACGGC AGTGGTCCAG TGGCAGAATT
             CTCGTCCTCT AGCAGGAGAC CCAGGTTGTA TTCCTGGCTA ACGCACCTCC
             AGGGCAGCTG CTACCCGCTT GTCAGTGGAA GCTTGCGTGT TGCTATGA
MAM1_SP134   AGGCCCATTT GCCTCCTTAC TGAGGACGGC AGTGGTCCAG TGGCAGAATT
             CTCGTCCTCT AGCAGGAGAC CCAGGTTGTA TTCCTGGCTA ACGCACCTCC
             AGGGCAGCTG CTACCCGCTT GTCGGTGGTA GCTTGCGTGT TGCTATGA
MAST_B008_   AGGCCCATTT GCCTCCTTAC TGAGGACGGC AGTGGTCCAG TGGCAGAATT
             CTTGTCCTCT AGCAGGAGAC CCAGGTTGTA TTCCTGGCTA ACGCACCTCC
             AGTGCAGCTG CTACCCGCTT GTCAGTGGAA GCTTGCGTGT TGCTATGA
SAV1_SE210   AGGCCCATTT GCCTCCTTAC TGAGGACGGC AGTGGTCCAG TGGCAGAATT
             CTCGTCCTCT AGCAGGAGAC CCAGGTTGTA TTCCTGGCTA ACGCACCTCC
             AGGGCAGCTG CTACCCGCTT GTCAGTGGAA GCTTGCGTGT TGCTATGA

 1 1 1 1 1
5 145

ASIAN2_Ema   ACTGGGGACA TTTATGTTTG CTGGTTACTA CGGAAATTAC TATGGATTAC
             TGCCTCTAGG AAGGAAGTCT AGTGGAGGGG GCTTAGGGAG GAGGTTTACC
             GAATTTCATT AGG-TCTCTC TTCCTCCTCA TATCTCTTTT TGTCT
FOR_DS1535   ACTGGGGACA TTTATGTTTG CTGGTTACTA CGGAAATTAC TATGGATTAC
             TGCCTCTAGG AAGGAAGTCT AGTGGAGGGG GCTTAGGGAG GAGGTTTACC
             GAATTTCATT AGG-TCTCTC TTCCTCCTCA TATCTCTTTT TGTCT
MAM1_SP134   ACTGGGGACA TTTATGTTTG CTGGTTACTA CGGAAATTAC TATGGATTAC
             TGCCTCTAGG AAGGAAGTCT AGTGGAGGGG GCTTAGGGAG GAGGTTTACC
             GRATTTCATT AGG-TCTCTC TTCCTCCTCA TATCTCTTTT TGTCT
MAST_B011_   ATTGGGGATA TTTATGTTTG CTGGTTACTA TGGAAATTAC TATGGATTAC
             TGCCTCTAGG AAGGAAGTCT AGTGGAGGGG GCTTGGGGAG GAGGTT-ACC
             GAATTTCATT AGGGTCTCTC TTCCTCCTCA TATCTCTTTT TGTCT
SAV1_SE210   ACTGGGGACA TTTATGTTTG CTGGTTACTA CGGAAATTAC TATGGATTAC
             TGCCTCTAGG AAGGAAGTCT AGTGGAGGGG GCTTAGGGAG GAGGTTTACC
             GAATTTCATT AGG-TCTCTC TTCCTCCTCA TATCTCTTTT TGTCT

 1 1 1 1 1
5 143

ASIAN2_Ema   CGTTTCTCCG GGTGCACATC CAAGTGAAAT AAGGTGGAAT GCCTCAGCAT
```

```
            CCACTGGCCA CACCCTTCCC ACTTGCCCCA GGTGGGTGCC AAGGGAGGCA
            ACCCCAGAGA GCACACACAG GACTATCCAC GCCAGCACCG CTG
FOR_DS1535  CGTTTCTCCG GGTGCACATC CAAGTGAAAT AAGGTGGAAT GCCTCAGCAC
            CCACTGACCA CACCCTTCCC ACTTGCCCCA GGTGGGTGCC AAGGGAGGCA
            ACCCCAGAGA GCACACACAG GACTATCCAC GCCAGCACCG CTG
MAM1_SP134  CGTTTCTCCG GGTGCACATC CAAGTGAAAT AAGGTGGAAT GCCTCAGCAC
            CCACTGGCCA CACCCTTCCC ACTTGCCCCA GGTGGGTGCC AAGGGAGGCA
            ACCCCAGAGA GCACACACAG GACTATCCAC GCCAGCACCG CTG
MAST_B014_  TGTTTCTTCG GGTGCACATC CAAGTGAAAT AAGGTGGAAT GCCTCAGCAC
            CCACTGGCCA CACCCTTTCC ACTTGCCCCA AGTGGGTGCC AAGGGAGGCA
            ACCCCAGAGA GCACACACAG GACTATCCAC GCCAGCACCG CTG
SAV1_SE210  CGTTTCTCCG GGTGCACATC CAAGTGAAAT AAGGTGGAAT GCCTCAGCAC
            CCACTGGCCA CACCCTTCCC ACTTGCCCCA GGTGGGTGCC AAGGGAGGCA
            ACCCCAGAGA GCACACACAG GACTATCCAC GCCAGCACCG CTG

 1 1 1 1 1
5 144

ASIAN2_Ema  GGTACCATGA CCTCTGAGGG TCCTCCAGCC TCAATCAGAC CATTAAGTCT
            GGTCTTTTTA C-CAGAATTT GAGGTCTACC ACCCACTGTT CTCCTGCTCC
            ATCAAGGATT CTCTGTAGTG TTCCCAGTCA TTGGTGGTAG CTGG
FOR_DS1535  GGTACCATGA CCTCTGAGGG TCCTCCAGCC TCAATCAGAC CATTAAGTCT
            GGTCTTTTTA CCTAGAATTT GAGGTCTACC ACCCACTGTT CTCCTGCTCC
            ATCAAGGATT CTCTGTAGAG TTCCCAGTCA TTGGTGGTAG CTGG
MAM1_SP134  GGTACCATGA CCTCTGAGGG TCCTCCAGCC TCAATCAGAC CATTAAGTCT
            GGTCTTTTTA C-CAGAATTT GAGGTCTACC ACCCACTGTT CTCCTGCTCC
            ATCAAGGATT CTCTGTAGTG TTCCCAGTCA TTGGTGGTAG CTGG
MAST_B017_  GGTACCATGA ACTCTGAGGG TCCTCCAGCC TCAATCAGAC CATTAAGTCT
            GGTCTTTT-A C-TAGAATTT GAGGTTTACC ACCCACTGTT CTCCTGCTTC
            ATCAAGGATT CTCTGTAGTG TTCCCAGTCA TTGGTGGTAA CTGA
SAV1_SE210  GGTACCATGA CCTCTGAGGG TCCTCCAGCC TCAATCAGAC CATTAAGTCT
            GGTCTTTTTA C-TAGAATTT GAGGTCTACC ACCCACTGTT CTCCTGCTCC
            ATCAAGGATT CTCTGTAGTG TTCCCAGTCA TTGGTGGTAG CTGG

 1 1 1 1 1
5 139

ASIAN2_Ema  TCCTGTTGTG CAGTTAAGAA A-CGGATTTT -CATGAAAAA AA-TAATTCC
            TCTTATGTTG GGTTGG-AAA -TGGTTGACA GTGGACTCCA TCTCAGTGCA
            TCCACGGAGC -AGCACCTAG TAGGAATGTT GGAGAACAC
FOR_DS1535  TCCTGTTGTG AAGTTAAGAA A-CGGATTTT -CATGAAAAA AA-TAATTCC
            TCTTATGTTG GGTTGG-AAA -TGGTTGACA GTGGACTCCA TCTCAGTGCA
            TCCAYGGAGC -AGCACCTAG TAGGAATGTT GGAGAACAC
MAM1_SP134  TCCTGTTGTG CAGTTAAGAA A-CGGATTTT -CATGAAAAA AA-TAATTCC
            TCTTATGTTG GGTTGG-AAA -TGGTTGACA GTGGACTCCA TCTCAGTGCA
            TCCACGGAGC -AGCACCTAG TAGGAATGTT GGAGAACAC
MAST_B022_  TCCTGTTGTG AAGTTAAGAA AATGGATTTT TCATGAAAAA AAATAATTCC
            TCTTATGTTG GGTTGGGAAA ATGGTTGACA GTGGACTCCA TCTCAGTGCA
            TCCACAGAGC GAGCACCTAG TAGGAATGTT GGAGAACAC
SAV1_SE210  TCCTGTTGTG AAGTTAAGAA A-CGGATTTT -CATGAAAAA AA-TAATTCC
            TCTTATGTTG GGTTGG-AAA -TGGTTGACA GTGGACTCCA TCTCAGTGCA
            TCCACGGAGC -AGCACCTAG TAGGAATGTT GGAGAACAC

 1 1 1 1 1
5 139

ASIAN2_Ema  TCCTGTTGGG TTATT-CTGC TGACAGCTAC CTTTTGGTRT GGGGACCCAG
            CCCATTTAGG GCTCTGGAGA TTGTGTTGCA GAAGGGGTAT AAACTTTCCT
            GGACCCTGC- ACCC-ACC-T G-TGTTTGTG GTGGCCAGT
FOR_DS1535  TCCTGTTGGG TTATT-CTGC TGACAGCTAC CTTTTGGTGT GGGGACCCAG
            CCCATTTAGG GCTCTGGAGA TTGTGTTGCA GAAGGRGTAT AAACTTTCCT
            GGACCCTGC- ACCC-ACC-T G-TGTTTGTG GTGGCCAGT
MAM1_SP134  TCCTGTTGGG TTATT-CTGC TGACAGCTAC CTTTTGGTGT GGGGACCCAG
            CCCATTTAGG GCTCTGGAGA TTGTGTTGCA GAAGGGGTAT AAACTTTCCT
            GGACCCTGC- ACCC-ACC-T G-TGTTTGTG GTGGCCAGT
MAST_B023_  TTTTGTTGGG TTATTTCTGC TAACAACTAC CTTTTAGTGT GGGGACCCAG
            CCCATTTAGG GCTCTANAAA TTGTGTTGCA GAAGGTGTAT AAACTNCCCT
            GGACCCTGCC ACCCCACCCT GGTGTTTGTG GTGGCCAGT
SAV1_SE210  TCCTGTTGGG TTATT-CTGC TGACAGCTAC CTTTTGGTGT GGGGACCCAG
            CCCATTTAGG GCTCTGGAGA TTGTGTTGCA GAAGGGGTAT AAACTTTCCT
            GGACCCTGC- ACCC-ACC-T G-TGTTTGTG GTGGCCAGT
```

```
 1 1 1 1 1
5 136

ASIAN2_Ema    CCTGCGCTGG CGTCCAGGCT CTCCCGTTTA TCAGCTTCTA AAGCCTGGGA
              AGTGTTTAAC CTCTCTGTGC CTCGGTTTCC TCACTGA-TA AACA-TGGGA
              ACTGATAACT GTGTCATTGT GAGCATTTGG AACTGT
FOR_DS1535    CCTGCGCTGG CATCCAGGCT CTCCCATTTA TCAGCTTCTA AAGCCTGGGA
              AGYGCTTAAC CTCTCTGTGC CTCGGTTTCC TCACTGA-TA AACA-TGGGA
              ACTGATAACT GTGTCATTGT GAGCAYTTGG AACCGT
MAM1_SP134    CCTGCGCTGG CGTCCAGGCT CTCCCATTTA TCAGCTTCTA AAGCCTGGGA
              AGTGCTTAAC CTCTCTGTGC CTCGGTTTCC TCACTGA-TA AACA-TGGGA
              ACTGATAACT GTGTCATTGT GAGCATTTGG AACCGT
MAST_B025_    CCTGCCCTGG CGTCCAGGCT CTCCCATTTA TCAGCTTCTA AAGCCTGGGA
              AGTGCTTAAC CTCTCTGTGC CTCGGTTTCC TCACTGAATA AACAGTGGGA
              ACTGATAACT GTGTCATTGT GAGCATTTGG AACAAT
SAV1_SE210    CCTGTGCTGG CGTCCAGGCT CTCCCATTTA TCAGCTTCTA AAGCCTGGGA
              AGTGCTTAAC CTCTCTGTGC CTCGGTTTCC TCACTGA-TA AACA-TGGGA
              ACTGATAACT GTGTCATTGT GAGCATTTGG AACCGT

 1 1 1 1 1
5 135

ASIAN2_Ema    TCCCTGCAGG GGCAGGAGAG TGGCAGAGTT TGGCTCACCT CCCTCCTGGC
              CCGGCTCCCT CTAGTCACCA GAGACTAATA GGAACCATGC CAGCCCCAGC
              CGGTCTCGGC TTTAGCTTCT GTGATGTTGC CTGTG
FOR_DS1535    TCCCTGCAGG GGCAGGAGAG TGGCAGAGTT TGGCTCACCT CCCTCCTGGC
              CCGGCTCCCT CTAGTCACCA GAGACTAATA GGAACCATGC CAGCCCCAGC
              CGGTCTCAGC TTTAGCTTCT GTGATGTTGC CTGTG
MAM1_SP134    TCCCTGCAGG GGCAGGAGAG TGGCAGAGTT TGGCTCACCT CCCTCCTGGC
              CCGGCTCCCT CTAGTCACCA GCGACTAATA GGAACCATGC CAGCCCCAGC
              CAGTCTCAGC TTTAGCTTCT GTGATGTTGC CTGTG
MAST_B029_    TCCCTGCAGG GGCAGGAGAG TGGCAGAATT TGGTTCACCT CCCTCCTGGC
              TCGGCTCCCT CTAGTCACCA GAGATTAATA GGAACCATGT CAGCCCCAGC
              CGGTCTCAGC TTTAGCTTCT GTGATGTTGC CTGTA
SAV1_SE210    TCCCTGCAGG GGCAGGAGAG TGGCAGAGTT TGGCTCACCT CCCTCCTGGC
              CCGGCTCCCT CTAGTCACCA GAGACTAATA GGAACCATGC CAGCCCCAGC
              CGGTCTCAGC TTTAGCTTCT GTGATGTTGC CTGTG

 1 1 1 1 1
5 136

ASIAN2_Ema    CTTAATCTTC TTTAGGCCTG GGCACAACAC AGTCAGTGCG CAGGGAGGGA
              TTTGAGCTGG GGCTGGAGTT CAGACTGAGG TGGGGAGTTG TGGAACTTGC
              CCCATTTTGG GGTCCCC-TT CAAGGGCCCT CAGGAC
FOR_DS1535    CTTAATCTTC TTTAGGCCTG GGCACAACAC AGTCAGTGCG CAGGGAGGGA
              TTTGAGCTGG GGCTGGAGTT CAGACTGAGG TGGGGAGTTG TGGAACTTGC
              CCCATTTTGG GGTCCCCCTT CAAGGGCCCT CAGGAC
MAM1_SP134    CTTAATCTTC TTTAGGCCTG GGCACAACAC AGTCAGTGCG CAGGGAGGGA
              TTTGAGCTGG GGCTGGAGTT CAGACTGAGG TGGGGAGTTG TGGAACTTGC
              CCCATTTTGG GGTCCCC-TT CAAGGGCCCT CAGGAC
MAST_B082_    CTTAATCTTC TTTAGGCCTG GGCACAACAC AGTCAGTGCG CAGGGAGGGA
              TTTGAGCTGG GGCTGGAGTT CAGACTGAGG TGGGGAGTTG TGGAACTTGC
              CCCATTTTGG G-TCCCC-TT CAAGGGTCCT CAG-AT
SAV1_SE210    CTTAATCTTC TTTAGGCCTG GGCACAACAC AGTCAGTGCG CAGGGAGGGA
              TTTGAGCTGG GGCTGGAGTT CAGACTGAGG TGGGGAGTTG TGGAACTTGC
              CCCATTTTGG GGTCCCCCTT CAAGGGCCCT CAGGAC

 1 1 1 1 1
5 132

ASIAN2_Ema    CCCATGAGCC CCCAGCCATC TCTTGAGATG CTTCCTCCCA GACCATTGGG
              GAAAA-CCAG TCTGAATTCA GCATTTATGA GAACAAAAGA GAAGCAGTTG
              TGACCCAGAG ACTACAAATG TTTTTCTTAA GC
FOR_DS1535    CCCATGAGCC CCCAGCCATC TCTTGAGATG CTTCCTCCCA GACCATTGGG
              GAAAAGCCAG TCTGAATTCA GCATTTATGA GAACAAAAGA GAAGCAGTTG
              TGACCCAGAG ACTACAAATG TTTTTCTTAA GC
MAM1_SP134    CCCATGAGCC CCCAGCCATC TCTTGAGATG CTTCCTCCCA GACCATTGGG
              GAAAAGCCAG TCTGAATTCA GCATTTATGA GAACAAAAGA GAAGCAGTTG
              TGACCCAGAG ACTACAAATG TTTT-CTTAA GC
MAST_B033_    CCCATGAGCC CCCAGCTACC TCTTGAGATG CTTCCTCCCA GACCATTGGG
```

```
            GAAAAGCCAG TCTGAATTCA GCATTTATGA GAACAAAAGA GAAGCAGTTG
            TGACCCAAAG ACTACAAATG TTTTTCTTAA AC
SAV1_SE210  CCCATGAGCC CCCAGCCATC TCTTGAGATG CTTCCTCCCA GACCATTGGG
            GAAAAGCCAG TCTGAATTCA GCATTTATGA GAACAAAAGA GAAGCAGTTG
            TGACCCAGAG ACTACAAATG TTTTTCTTAA GC

 1 1 1 1 1
5 131

ASIAN2_Ema  TTGA--CCTG CCTGGGGGAT CTGCCCCAC- ACCCTTCCCC TGAGTCTTTG
            GAACTTGCAT CACCTATGTC AATGTGCCAG ACAGCCAGGA GGTCCAAGGC
            CTCTGGGGGT GCCTTTTACA AGTGTAGGAG C
FOR_DS1535  TTGA--CCTG CCTGGGGGAT CTGCCCCAC- ACCCTTCCCC TGAGTCTTTG
            GAACTTGCAT CACCTATGTC AATGTGCCAG ACAGCCAGGA GGTCCAAGGC
            CTCTGGGGGT GCCTTTTACA AGTGTAGGAG C
MAM1_SP134  TTGA--CCTG CCTGGGGGAT CTGCCCCAC- ACCCTTCCCC TGAGTCTTTG
            GAACTTGCAT CACCTATGTC AATGTGCCAG ACAGCCAGGA GGTCCAAGGC
            CTCTGGGGGT GCCTTTTACA AGTGTAGGAG C
MAST_B035_  TTCAAAACTG CCTGGGGGAT CTGCCCCACC ACCCTTCACC TGGGTCTTTG
            GAACTTGCAT CACCTATGTC AATGTGCCAG ACAGCCAGGA GGTCCAAGGC
            CTCTAGGAAT GCCTTTTACG AGTGTAGGAG C
SAV1_SE210  TTGA--CCTG CCTGGGGGAT CTGCCCCAC- ACCCTTCCCC TGAGTCTTTG
            GAACTTGCAT CACCTATGTC AATGTGCCAG ACAGCCAGGA GGTCCAAGGC
            CTCTGGGGGT GCCTTTTACA AGTGTAGGAG C

 1 1 1 1 1
5 132

ASIAN2_Ema  TGGTTAAGTG GCTTGTCTGA GGATACTCAG CTAGGTGAAG CCAAGAAGGT
            CAAAGGCCAG GGCTATGTGA GTCCACGCCC AACCCCAACA TCACCCCCAA
            ACACCGTTTA GCCCCAGCCA AGCTTTCTCT CT
FOR_DS1535  TGGTTAAGTG GCTTGTCTGA GGATACTCAG CTAGGTGAAG CCAAGAAGGT
            CAAAGGCCAG GGCTATGTGA GTCCACGCCC AACCCCAACA TCACCCCCAA
            ACACCGTTTA GCCCCAGCCA AGCTTTCTCT CT
MAM1_SP134  TGGTTAAGTG GCTTGTCTGA GGATACTCAG CTAGGTGAAG CCAAGAAGGT
            CAAAGGCCAG GGCTATGTGA GTCCACGCCC AACCCCAACA TCACCCCCAA
            ACACCGTTTA GCCCCAGCCA AGCTTTCTCT CT
MAST_B036_  TGGTTAAGTG GCTTGTCTGA GGATACTCAG CTAGGTGAAG CCAAGAAGGT
            CAAAGGCCAG GGCTATGTGA GTCCATGTCC AACTCCAACA TCACCCC-AA
            ACACCGTTTA GCCCCAGCCA AGCTTTCTCT CT
SAV1_SE210  TGGTTAAGTG GCTTGTCTGA GGATACTCAG CTAGGTGAAG CCAAGAAGGT
            CAAAGGCCAG GGCTATGTGA GTCCACGCCC AACCCCAACA TCAGCCCCAA
            ACACCGTTTA GCCCCAGCCA AGCTTTCTCT CT

 1 1 1 1 1
5 132

ASIAN2_Ema  TTGGAAAGAG TATCTTAAGC ACAAAGGGTT CTTGATAAAC ATTGAATAAT
            GGCAGAGACA GAGAGAAAGA GAGAGGGAGA GAAGAAAGAA AGGAAGGAGG
            AAAGGAGGGA GGGAGTGGAA AAGGGAAAAA GG
FOR_DS1535  TTGGAAAGAG TATCTTAAGC ACAAAGGGTT CTTGATAAAC ATTGAATAAT
            GGCAGAGACA GAGAGAAAGA GAGAGGGAGA GAAGAAAGAA AGGAAGGAGG
            AAAGGAGGGA GGGAGTGGAA AAGGGAAAAA GG
MAM1_SP134  TTGGAAAGAG TATCTTAAGC ACAAAGGGTT CTTGATAAAC ATTGAATAAT
            GGCAGAGACA GAGAGAAAGA GAGAGGGAGA GAAGAAAGAA AGGAAGGAGG
            AAAGGAGGGA GGGAGTGGAA AAGGGAAAAA GG
MAST_B083_  TTGGAAAGAG TATCTTAAGC ACAAAGGGTT -TTGATAAAC ATTGAATAAT
            GGCAGAGACA NAGAGAAAGA GAGAGGGAGA GAAGAAAGAA AGGAAGGAGG
            AAAAGAGGGA GGGAGTGGAA AAGGGAAAAA GG
SAV1_SE210  TTGGAAAGAG TATCTTAAGC ACAAAGGGTT CTTGATAAAC ATTGAATAAT
            GGCAGAGACA GAGAGAAAGA GAGAGGGAGA GAAGAAAGAA AGGAAGGAGG
            AAAGGAGGGA GGGAGTGGAA AAGGGAAAAA GG

 1 1 1 1 1
5 130

ASIAN2_Ema  GAGCCGCCTG CTCGAGGAGA A-GGAA-TCT -C-TGATT-A GCCAGC-TGA
            GCCGTGGGAA GACCTCTGCC -ACCC-AGAG CCTAGAGGAG CTAC-GG-AG
            G-C-AGCTGG AGGAGGA-GA GC-AAGGTGG
FOR_DS1535  GAGCCGCCTG CTCGAGGAGA A-GGAA-TCT -C-TGATT-A GCCAGC-TGA
            GCCGTGGGAA GACCTCTGCC -ACCC-AGAG CCTAGAGGAG CTAC-GG-AG
```

```
              G-C-AGCTGG AGGAGGA-GA GC-AAGGTGG
MAM1_SP134    GAGCCGCCTG CTCGAGGAGA A-GGAA-TCT -C-TGATT-A GCCAGC-TGA
              GCCGTGGGAA GACCTCTGCC -ACCC-AGAG CCTAGAGGAG CTAC-GG-AG
              G-C-AGCTGG AGGAGGA-GA GC-AAGGTGG
MAST_B084_    GAGCCGCCTG CTCGAGGAGA AAGGAACTCT TCCTGATTTA GCCAGCCTGA
              GCCATGGGAA GACCTCTGCC CACCCCAGAG CCTAGAGGAG CTACCGGGAG
              GGCGAGCTGG AGGAGGAAGA GCCAAGGTGA
SAV1_SE210    GAGCCGCCTG CTCGAGGAGA A-GGAA-TCT -C-TGATT-A GCCAGC-TGA
              GCCGTGGGAA GACCTCTGCC -ACCC-AGAG CCTAGAGGAG CTAC-GG-AG
              G-C-AGCTGG AGGAGGA-GA GC-AAGGTGG

 1 1 1 1 1
5 132

ASIAN2_Ema    GAGTGTTGGG GAAAGGGATT ACCAGGGAGC ATCGGCATGC TCACTGGGAG
              GCAGATGGGA CTAGGTTGTT GCCTGGGCAA TACCACACAG CAGCCTGCAT
              GCCATTTCAG GCTCTTCTCC GAAGATGGCA AT
FOR_DS1535    GAGTGTTGGG GAAAGGGATT ACCAGGGAGC ATCGGCATGC TCACTGGGAG
              GCAGATGGGA CTAGGTTGTT GCCTGGGCAA TACCACACAG CAGCCTGCAT
              GCCATTTCAG GCTCTTCTCC GAAGATGGCA AC
MAM1_SP134    GAGTGTTGRG GAAAGGGATT ACCAGGGAGC ATCGGCATGC TCACTGGGAG
              GCAGATGGGA CTAGGTTGTT GCCTGGGCAA TACCACACAG CAGCCTGCAT
              GCCATTTCAG GCTCTTCTCC GAAGATGGCA AT
MAST_B037_    GAGTGTTGGG -AAAGGGATT ACCAGG-AGC ATCGGCATGC TCACTGGGAG
              GCAGATGGGA CTAGGTTGTT GCCTGGGCAA TACCACACAG CAGCCTGCAT
              GCCATTTCAG GCTCTTCTCC GAAGATGGCA AC
SAV1_SE210    GAGTGTTGGG GAAAGGGATT ACCAGGGAGC ATCGGCATGC TCACTGGGAG
              GCAGATGGGA CTAGGTTGTT GCCTGGGCAA TACCACACAG CAGCCTGCAT
              GCCATTTCAG GCTCTTCTCC GAAGATGGCA AC

 1 1 1 1 1
5 130

ASIAN2_Ema    CCCGCCAGAG TCCAGCTCTG CAGAGCTCCC TAGTAACACC TGGGCTCCCA
              GCCTGGCAGG GCACTTGGCG AACCTGCTGC ATCTCCACCG TGAATACCAC
              AGGCCCAGGC TGGGGGAGGG GACTCTGGAG
FOR_DS1535    CCCGCCAGAG TCCAGCTCTG CAGAGCTCCC TAGTAACACC TGGGCTCCCA
              GCCTGGCAGG GCACTTGGCG AACCTGCTGC GTCTCCACCG TGAATACCAC
              AGGCCCAGGC TGGGGGAGGG GACTCTGGAG
MAM1_SP134    CCCGCCAGAG TCCAGCTCTG CAGAGCTCCC TAGTAACACC TGGGCTCCCA
              GCCTGGCAGG GCACTTGGCG AACCTGCTGC ATCTCCACCG TGAATACCAC
              AGGCCCAGGC TGGGGGAGGG GACTCTGGAG
MAST_B038_    TCCGCCAGAG TCCAGCTCTG TAGAGCTCCC TAGTAACACT TGGGCTCCCA
              GCCTGGCAGG GCACTTGGCG AACCTGCTGC GTCTCCACCG TGAATACCAC
              AGGCCCAGGC TGGGGGAGGG GACTCTGAAA
SAV1_SE210    CCCGCCAGAG TCCAGCTCTG CAGAGCTCCC TAGTAACACC TGGGCTCCCA
              GCCTGGCAGG GCACTTGGCG AACCTGCTGC GTCTCCACCG TGAATACCAC
              AGGCCCAGGC TGGGGGAGGG GACTCTGGAG

 1 1 1 1 1
5 129

ASIAN2_Ema    CCAAAGTCAG AACTCAAAAA GGGCAGCCCA CCGCTCACAG CAGCCTGCCA
              GGCTAGTCTT TCAGCTGCCG CCACCTTCCA ACCATCTCCA GCCATTGCCC
              TGTTGCCTGA GGCCGTCAAT GAATTCTTA
FOR_DS1535    CCAAAGTCAG AACTCAAAAA GGGCAGCCCA CCGCTCACAG CAGCCTGCCA
              GGCTAGTCTT TCAGCTGCTG CCACCTTCCA ACCATCTCCA GCCATTGCCC
              TGTTGCCTGA GGCCGTCAAT GAATTTTTA
MAM1_SP134    CCAAAGTCAG AACTCAAAAA GGGCAGCCCA CCGCTCACAG CAGCCTGCCA
              GGCTAGTCTT TCAGCTGCCG CCACCTTCCA ACCATCTCCA GCCATTGCCC
              TGTTGCCTGA GGCCGTCAAT GAATTCTTA
MAST_B040_    TCAAAGTCAG AACTCAAAAA GGGCAGCCCA CTGCTCACAG CAGCCTGCCA
              GGCTAGTCTT TCAGCTGCTG CCACCTTCCA ACCATCTCCA GCCATTGCCC
              TGTTGCCTGA GGCCGTCAAT GAATTCTTA
SAV1_SE210    CCAAAGTCAG AACTCAAAAA GGGCAGCCCA CCGCTCACAG CAGCCTGCCA
              GGCTAGTCTT TCAGCTGCTG CCACCTTCCA ACCATCTCCA GCCATTGCCC
              TGTTGCCTGA GGCCGTCAAT GAATTTTTA

 1 1 1 1 1
5 130
```

```
ASIAN2_Ema    TCTCCCGAAC GCCTTGGTTC CAGGAACCAA GGAAATCCTG TCACGAAGTG
              AATGAGCACT ACAAAAGAAT CAGAGCTGCT TGCAGGATGA GCTCTGCCTC
              CTGGAGACTT CCTCTAGATT AGACAACCTG
FOR_DS1535    TCTCCCGAAC GCCTTGGTTC CAGGAACCAA GGAAATCCTG TCACGAAGTG
              AATGAGCACT ACAAAAGAAT CAGAGCTGCT TGCAGGATGA GCTCTGCCTC
              CTGGAGACTT CCTCCAGATT AGACAACCTG
MAM1_SP134    TCTCCCGAAC GCCTTGGTTC CAGGAACCAA GGAAATCCTG TCACGAAGTG
              AATGAGCACT ACAAAAGAAT CAGAGCTGCT TGCAGGATGA GCTCTGCCTC
              CTGGAGACTT CCTCCAGATT AGACAACCTG
MAST_B041_    TCTCCGGAAC ACCTTGGTTC CAGGAACCAA GGAAATCCTG TCACGAAGTG
              AATGAGCACT ACAAA-GAAT CAGAGCTGCT TGCAGGATGA GCTCTGCCTC
              CTGGAGACTT CCTCCAGATT AGACAACCTG
SAV1_SE210    TCTCCCGAAC GCCTTGGTTC CAGGAACCAA GGAAATCCTG TCACGAAGTG
              AATGAGCACT ACGAAAGAAT CAGAGCTGCT TGCAGGATGA GCTCTGCCTC
              CTGGAGACTT CCTCCAGATT AGACAACCTG

 1 1 1 1 1
5 128

ASIAN2_Ema    CCATGAAGTT AC-CTGCAGA GCTTTGGCTG TCCAGAGATT TCAGTCTTCC
              ATCAATGTCA TCCTCTTAAA CAACAACAAA AAAGCAGTGG CCCCAATGAG
              GCTTCCTGGC CTTGCTCTTT ACTTCCGC
FOR_DS1535    CCATGAAGTT AC-CTGCAGA GCTTTGGCTG TCCAGAGATT TCAGTCTTCC
              ATCAATGTCA TCCTCTTAAA CAACAACAAA AAAGCAGTGR CCCCAATGAG
              GCTTCCTGGC CTTGCTCTTT ACTTCCGC
MAM1_SP134    CCATGAAGTT AC-CTGCAGA GCTTTGGCTG TCCAGAGATT TCAGTCTTCC
              ATCAATGTCA TCCTCTTAAA CAACAACAAA AAAGCAGTGG CCCCAATGAG
              GCTTCCTGGC CTTGCTCTTT ACTTCCGC
MAST_B086_    TCATGAAGTT ATTCTGCAGA GCTTTGGCTA TCCAGAGATT TCAGTCTTCC
              ATCAATGTCA TCCTCTTAAA CAACAACAAA AAAGCAGTGG CCC-AATGAG
              GCTTCCTGGC CTTGCTCTTT ACTTCTGC
SAV1_SE210    CCATGAAGTT AC-CTGCAGA GCTTTGGCTG TCCAGAGATT TCAGTCTTCC
              ATCAATGTCA TCCTCTCAAA CAACAACAAA AAAGCAGTGG CCCCAATGAG
              GCTTCCTGGC CTTGCTCTTT ACTTCCGC

 1 1 1 1 1
5 127

ASIAN2_Ema    ATTTAGTCTG CCTGTTAC-A CAGAAGATTG TTATGAGTCG GAGATTCGAT
              GGCAATTAAC AACAATGGTT TACTTAAAAA AATATAGCAA ATTCTCAGTC
              ATAATA-TAA AATTTAACCC AATTATT
FOR_DS1535    ATTTAGTCTG CCTGTTAC-A CAGAAGATTG TTATGAGTCG GAGATTCGAT
              GGCAATTAAC AACAAYGGTT TACTTAAAAA AATATAGCAA ATTCTCAGTC
              ATAATA-TAA AATTTAACCC AATTATT
MAM1_SP134    ATTTAGTCTG CCTGTTAC-A CAGAAGATTG TTATGAGTCG GAGATTCGAT
              GGCAATTAAC AACAATGGTT TACTTAAAAA AATATAGCAA ATTCTCAGTC
              ATAATA-TAA AATTTAACCC AATTATT
MAST_B048_    ATTTAGTCTG CCTGTTACCA CAGAAGATTG TTATGAGTCG GAGATTCGAT
              GGCAATTAAC AACAATGGTT TACTTAAAAA A-TATAGCAA ATTCTCAGTC
              ATAATAATAA AATTTAACCC AATTATT
SAV1_SE210    ATTTAGTCTG CCTGTTAC-A CAGAAGATTG TTATGAGTCG GAGATTCGAT
              GGCAATTAAC AACAACGGTT TACTTAAAAA AATATAGCAA ATTCTCAGTC
              ATAATA-TAA AATTTAACCC AATTATT

 1 1 1 1 1
5 126

ASIAN2_Ema    CATTAGATTC ATTCTTTAGC AAAAATCATC AAGGGAATCA GACCAGCCTT
              CACTGTAATG TGGAGTGAGA GCTTATCCTA ATTTGTTTGG CACCTCTTAA
              CTCTCCCATT CTTTGTGTTG TTGGAA
FOR_DS1535    CATTAGATTC ATTCTTTAGC AAAAATCATC AAGGGAATCA GACCAGCCTT
              CACTGTAATG TGGAGAGAGA GCTTATCCTA ATTTGTTTGG CACCTCTTAA
              CTCTCCCATT CTTTGTGTTG TTGGAA
MAM1_SP134    CATTAGATTC ATTCTTTAGC AAAAATCATC AAGGGAATCA GACCAGCCTT
              CACTGTAATG TGGAGAGAGA GCTTATCCTA ATTTGTTTGG CACCTCTTAA
              CTCTCCCATT CTTTGTGTTG TTGGAA
MAST_B088_    TATTAGATTC ATTTTTTAGC AAAAATCATC AAGGGAATCA GACCAGCCTT
              CACTGTAATG TGGAGAGAGA GCTTATCCTA ATTTGTTTGG CACCTCTTAA
              CTCTTCCATT ATTTGTGTTG TTGGAA
SAV1_SE210    CATTAGATTC ATTCTTTAGC AAAAATCATC AAGGGAATCA GACCAGCCTT
              CACTGTAATG TGGAGAGAGA GCTTATCCTA ATTTGTTTGG TACCTCTTAA
```

```
            CTCTCCCATT CTTTGTGTTG TTGGAA

 1 1 1 1 1
5 125

ASIAN2_Ema  AGCGCTGTGT TCTCAACAGC TCATAAATCA CGCATCTCCT TCAGAGGGGA
            GTTTAAAGAC TGCCCATCTG TTGGCTGGCG CCCTCCTCTC CCTTCCGGCG
            GACAGGCGAG GCCTGGGAGC CTTGC
FOR_DS1535  AGCGCTGTGT TCTCAACAGC TCATAAATCA CGCATCTCCT TCAGACGGGA
            GTTTAAAGAC TGCCCATCTG TTGGCTGGCG CCCTCCTCTC CCCTCCGGCG
            GACAGGCGAG GCCTGGGAGC CTTGC
MAM1_SP134  AGCGCTGTGT TCTCAACAGC TCATAAATCA CGCATCTCCT TCAGAGGGGA
            GTTTAAAGAC TGCCCATCTG TTGGCTGGCG CCCTCCTCTC CCTTCCGGCG
            GACAGGCGAG GCCTGGGAGC CTTGC
MAST_B050_  AGTGTTGTGT TTTCAACAGC TCATAAATCA CGCATTTCCT TCAGAGGGGA
            GTTTAAAGAC TGCCCGTCTG CTGGCTGGCA CCCTCCTCTC CCCTCCGGCG
            GACAGGCGAG GCCTGGGAGC CTTTA
SAV1_SE210  AGCGCTGTGT TCTCAACAGC TCATAAATCA CGCATCTCCT TCAGAGGGGA
            GTTTAAAGAC TGCCCATCTG TTGGCTGGCG CCCTCCTCTC CCTTCCGGCG
            GACAGGCGAG GCCTGGGAGC CTTGC

 1 1 1 1 1
5 124

ASIAN2_Ema  GCTAATGAGC TTGAAGTAAA AA-GAAA-TT TA-TCTCTGC ATCTGCCACT
            GGTGCCTTGG CCAAGTGCCT GCAGCCATGC CAGGCAGAAG CCAGGTTCTA
            CAGAAGTGGG TCTCCGAATG GTGC
FOR_DS1535  GCTAATGAGC TTGAAGTAAA AA-GACA-TT TA-TCTCTGC AGCTGCCACT
            GGTGCCTTGG CCAAGTGCCT GCAGCCATGC CAGGCAGAAG CCAGGTTCTA
            CAGAAGTGGG TCTCCGAATG GTGC
MAM1_SP134  GCTAATGAGC TTGAAGTAAA AA-GAAA-TT TA-TCTCTGC ATCTGCCACT
            GGTGCCTTGG CCAAGTGCCT GCAGCCATGC CAGGCAGAAG CCAGGTTCTA
            CAGAAGTGGG TCTCCGAATG GTGC
MAST_B054_  TTAAATGAGC TTGAAGTAAA AAAGAAAATT TAATCTCTGC AACTGCCACT
            GGTGCCTTGG CCAAGTGCCT GCAGCCATGC CAGGCAGAAG CCAGGTTCTA
            CAGAAGTGGG TCTCCGAATA ATGC
SAV1_SE210  GCTAATGAGC TTGAAGTAAA AA-GACA-TT TA-TCTCTGC AGCTGCCACT
            GGTGCCTTGG CCAAGTGCCT ACAGTCATGC CAGGCAGAAG CCAGGTTCTA
            CAGAAGTGGG TCTCCAAATG GTGC

 1 1 1 1 1
5 124

ASIAN2_Ema  TTACCCTTAC AAGTCTGTGA ATTAGACATG TTTATTCCGT TTTACAGAGG
            CCGAATCAAA GAGGTAAAAT GCCATCCTCA AGGTCACACA TCTATTAAGT
            CGAAGAGCCA GGATTCAATC CTAA
FOR_DS1535  TTACCCTTAC AAGTCTGTGA ATTAGACATG TTTATTCCCT TTTACAGAGG
            CCGAATCAAA GAGGTAAAAT GCCATCCTCA AGGTCACACA TCTATTAAGT
            CGAAGAGCCA GGATTCAATC CTAA
MAM1_SP134  TTACCCTTAC AAGTCTGTGA ATTAGACATG TTTATTCCCT TTTACAGAGG
            CCGGATCAAA GAGGTAAAAT GCCATCCTCA AGGTCACACA TCTATTAAGT
            CGAAGAGCCA GGATTCAATC CTAA
MAST_B056_  TTACCGTTAC AAGTCTGTGA ATTAGACATT TTTATTCCCT TTTACAGAGG
            CCAAATCAAA GAGGTAAAAT GCCATCCTCA AGGTTACACA TCTATTAAGT
            CAAAGAGCCA GGATTCAATC CTAA
SAV1_SE210  TTACCCTTAC AAGTCTGTGA ATTAGACATG TTTATTCCCT TTTACAGAGG
            CCGAATCAAA GAGGTAAAAT GCCATCCTCA AGGTCACACA TCTATTAAGT
            CGAAGAGCCA GGATTCAATC CTAA

 1 1 1 1 1
5 124

ASIAN2_Ema  TGAAGACACC AGTGGCCCCT CGACAGAAAA GACCAGGTTA TCTGCTCCGG
            TAAAGATTAC AGTCTAGGAA ACTCTATGGG GCAGTTCTAC TCTGTCCTAT
            AGGATTGCTA A-GAGTCAGA ATCA
FOR_DS1535  TGAAGACACC AGTGGCCCCT CGACAGAAAA GACCAGGTTA TCTGCTCCGG
            TAAAGATTAC AGTCTAGGAA ACTCTATGGG GCAGTTCTAC TCTGTCCTAT
            AGAATTGCTA A-GAGTCAGA ATCA
MAM1_SP134  TGAAGACACC AGTGGCCCCT CGACAGAAAA GACCAGGTTA TCTGCTCCGG
            TAAAGATTAC AGTCTAGGAA ACTCTATGGG GCAGTTCTAC TCTGTCCTAT
            AGGATTGCTA A-GAGTCAGA ATCA
```

```
MAST_B057_   TGAAGACACC AGTGGCCCCT CGACAGAAAA GACCAGGTGA TCCGCTCCGG
             TAAAGATTAT AGTCTAGGAA ACCCTATGGG GCAGTTCTAC TCCATCATAT
             TGGGTT-CTA ATGAGTCAGA ATTG
SAV1_SE210   TGAAGACACC AGTGGCCCYT CGACAGAAAA GACCAGGTTA TCTGCTCCGG
             TAAAGATTAC AGTCTAGGAA ACTCTATGGG GCAGTTCTAC TCTGTCCTAT
             AGGATTGCTA A-GAGTCAGA ATCA

 1 1 1 1 1
5 125

ASIAN2_Ema   CAGAGCTAAA AGAGAGAGAA TG-TTTCCCC TAGAGGTGGC ACTCTGAA-T
             TCAGACTGCT AGCCTCCTAA ACTGTGAGAA AATAAATTTC TGCTTGTTAA
             AGCCACCCAC TTGTGGTATT TCTGT
FOR_DS1535   CAGAGCTAAA AGAGASAGAA YR-TTTCCCC TAGAGGTGGC ACTCTGAA-T
             TCAGACTTCT AGCCTCCTAA ACTGTGAGAA AATAAATTTC TGCTTGTTAA
             AGCCACCCAC TTGTGGTATT TCTGT
MAM1_SP134   CAGAGCTAAA AGAGAGAGAA TG-TTTCCCC TAGAGGTGGC ACTCTGAA-T
             TCAGACTTCT AGCCTCCTAA ACTGTGAGAA AATAAACTTC TGCTTGTTAA
             AGCCACCCAC TTGTGGTATT TCTGT
MAST_B060_   TAGAGCTGAC AGAGAG--AA GGCTTTCCCC TAGAGTTGGC AC-CTGAAAT
             TCAGACTTTT AGCCTCCTAA TCTGTGAGAA AATAAATTTT TGCTTGTTAA
             AGCCACCCAT TTGTGGTATT TCTAT
SAV1_SE210   CAGAGCTAAA AGAGAGAGAA TG-TTTCCCC TAGAGGTGGC ACTCTGAA-T
             TCAGACTTCT AGCCTCCTAA ACTGTGAGAA AATAAATTTC TGCTTGTTAA
             AGCCACCCAC TTGTGGTATT TCTGT

 1 1 1 1 1
5 133

ASIAN2_Ema   AGGAATATTT TCTAGCCCAA ACTACCAGGA GTTTTCCAAG CAAATTTTAG
             ACACTGTGCC ATTCAGGATA TCACGCAGCT CCGGTTCCAA GGGGAATAAA
             TTCCCAACCT CCTCGAAAGG AACATTTTTT CCA
FOR_DS1535   AGGAATATTT TCTAGCCCAA ACTACCAGGA GTTTTCCAAG CAAATTTTAG
             ACACTGTGCC ATTCAGGATA TCACGCAGCT CCGGTTCCAA GGGGAATAAA
             TTCCCAACCT CCTCGAAAGG AACATTTTTT CCA
MAM1_SP134   AGGAATATTT TCTAGCCCAA ACTACCAGGA GTTTTCCAAG CAAATTTTAG
             ACACTGTGCC ATTCAGGATA TCATGCAGCT CCGGTTCCAA GGGGAATAAA
             TTCCCAACCT CCTCGAAAGG AACATTTTTT CCA
MAST_B094_   AGGAATATTT TCTAGCGCAA ACTACCAGGA GTTTTCCAAG CAATTTTTAG
             ATATTGTGCT ATTTAGGATA TCAGGCAGCT CTGGTTCCAA GGGGAATA--
             TTACGA---- --TGGTAAG- ---ATTTTTT GCT
SAV1_SE210   AGGAATATTT TCTAGCCCAA ACTACCAGRA GTTTTCCAAG CAAATTTTAG
             ACACTGTGCC ATTCAGGATA TCACGCAGCT CCGGTTCCAA GGGGAATAAA
             TTCCCAACCT CCTCGAAAGG AACATTTTTT CCA

 1 1 1 1 1
5 119

ASIAN2_Ema   TATCCACCTA GGGGAGCCTG TCTCATAACC GATGAACCCC GATACACCTT
             ACCGTCACTT GCTAATTCAG TCCATATACC ACCATCTTCA GCAAACCCCT
             ATAGGGCACA AAA-GTGAG
FOR_DS1535   TATCCACCTA GGGGAGCCTG TCTCGTAACC GATGAACCCC GATACACCTT
             ACCGTCACTT GCTAATTCAG TCCATATACC ACCATCTTCA GCAAACCCCT
             ATAGGGCACA AAA-GTGAG
MAM1_SP134   TATCCACCTA GGGGAGCCTG TCTCGTAACC GATGAACCCC GATACACCTT
             ACCGTCACTT GCTAATTCAG TCCATATACC ACCATCTTCA GCAAACCCCT
             ATAGGGCACA AAA-GTGAG
MAST_B099_   TATTCACCTA GGGGAGCCTG TCTCGTAACC GATGAACCCC GGTATACCTT
             ACCGTCACTT GCTAATTCA- TCCATATACC ACCATTTTCA GTAAACCCTT
             NTAGGGCACA AAAAGTAAA
SAV1_SE210   TATCCACCTA GGGGAGCCTG TCTCGTAACC GATGAACCCC GATACACCTT
             ACCGTCACTT GCTAATTCAG TCCATATACC ACCATCTTCA GCAAACCCCT
             ATAGGGCACA AAA-GTGAG

 1 1 1 1 1
5 118

ASIAN2_Ema   TGGTGATTAG AGCCCCTGCA TCGGAATCTC TGGGAATATC TGTTAATACT
             GCAGATTCCT GGGCCTCCAA GGGTGGGGCC TGGGATTCTG CATTCTCAAC
             AAGCAGCCAA GTGTTCCT
FOR_DS1535   TGGTGATTAG AGCYCCTGCA TCGGAATCTC TGGGAATATC TGTTAATACT
```

```
             GCAGATTCCT GGGCCTCCAA GGGTGGGGCC TGGGATTCTG CATTCTCAAC
             AAGCAGCCAA GTGTTCCT
MAM1_SP134   TGGTGATTAG AGCCCCTGCA TCGGAATCTC TGGGAATATC TGTTAATACT
             GCAGATTCCT GGGCCTCCAA GGGTGGGGCC TGGGATTCTG CATTCTCAAC
             AAGCAGCCAA GTGTTCCT
MAST_B100_   TGGTGATTAG AGCCCCTGCA TCGGAATCTC TGGGAATATC TGTTAATACT
             GCAGATTCCT GGGCCTCCAA GGGTGGGGCC TGGGATTCTG CATTCTCAAC
             AAGCAGCCAA ATATTCCT
SAV1_SE210   TGGTGATTAG AGCCCCTGCA TCGGAATCTC TGGGAATATC TGTTAATACT
             GCAGATTCCT GGGCCTCCAA GGGTGGGGCC TGGGATTCTG CATTCTCAAC
             AAGCAGCCAA GTGTTCCT

 1 1 1 1 1
5 116

ASIAN2_Ema   CTGGAAGCAC TTACTCTTAA CAGTAGGTTA AAGTCACCGC ATTTCTGATT
             GCTTCATCTG GCAAAGGCAG CTTCACTCAA GGCCATATCA CAGAATTCCA
             GGTGTAGCAG TTCAAT
FOR_DS1535   CTGGAAGCAC TTACTCTTAA CAGTAGGTTA AAGTCACCGC ATTTCTGATT
             GCTTCATCTG GCAAAGGCAG CTTYGCTCAA GGCCATATCA CAGAATTCCA
             GGTGTAGCAG TTCAAT
MAM1_SP134   CTGGAAGCAC TTACTCTTAA CAGTAGGTTA AAGTCACCGC ATTTCTGATT
             GCTTCATCTG GCAAAGGCAG CTTCACTCAA GGCCATATCA CAGAATTCCA
             GGTGTAGCAG TTCAAT
MAST_B101_   CTGGAAGTAC TTACTCTTAA CAGTAGGTTA AAGTCACCGC ACTTCTGATT
             GCTTCATCTG GCAAAGGCAG CTTCGCTCAA GGCCATATCA CAGAATTCCA
             GGTGTAGCAG TTCAAT
SAV1_SE210   CTGGAAGCAC TTACTCTTAA CAGTAGGTTA AAGTCACCGC ATTTCTGATT
             GCTTCATCTG GCAGAGGCAG CTTTGCTCAA GGCCATATCA CAGAATTCCA
             GGTGTAGCAG TTCAAT

 1 1 1 1 1
5 116

ASIAN2_Ema   AGATGAAATG AAATGGGGCT AATAGGCATT TTTAAAGAGA AGGCAAAGTA
             ATTCAGAGCA GGAGGTATGA AGCTCAGGAC CATGGAGATT T-CTTGCTTT
             -GGTTTTAGA ATCTTA
FOR_DS1535   AGATGAAATG AAATGGTGCT AATAGGCATT TTTAAAGAGA AGGCAAAGTA
             ATTCGGAGCA GGAGGTATGA AGCTCAGGAC CGTGGAGATT T-CTTGCTTT
             -GGTTTTAGA ATCTTA
MAM1_SP134   AGATGAAATG AAATGGTGCT AATAGGCATT TTTAAAGAGA AGGCAAAGTA
             ATTCGGAGCA GGAGGTATGA AGCTCAGGAC CGTGGAGATT T-CTTGCTTT
             -GGTTTTAGA ATCTTA
MAST_B102_   AGATGAAATG AAATGGTGCT AATAGGCATT TTTAAAGAGA AGGCAAAGTA
             ATTCGGAGCG GGAGATATGA AGCTCAGGAC CGTGGAGATT TTCTTGCTTT
             TGGTTTTGGA ATCTTA
SAV1_SE210   AGATGAAATG AAATGGTGCT AATAGGCATT TTTAAAGAGA AGGCAAAGTA
             ATTCGGAGCA GGAGGTATGA AGCTCAGGAC CGTGGAGATT T-CTTGCTTT
             -GGTTTTAGA ATCTTA

 1 1 1 1 1
5 115

ASIAN2_Ema   AGCCCTACCC CTATTTTCCC AAGAAGGATC CAATTCCTTC CGAGCTGTGT
             GCTTTCTCAT GCTGACCTGA TATCTGAAAC TTTGCTGAAA A-GTTCTTGT
             CTTTCTAACT AAACC
FOR_DS1535   AGCCCTACCC CTATTTTCCC AAGAAGGATC CAATTCCTTC CGAGCTGTGT
             GCTTTCTCAT GCTGACCTGA TATCTGAAAC TTTGCTGAAA A-GTTCTTGT
             CTTTCTAACT AAACC
MAM1_SP134   AGCCCTACCC CTATTTTCCC AAGAAGGATC CAATTCCTTC CGAGCTGTGT
             GCTTTCTCAT GCTGACCTGA TATCTGAAAC TTTGCTGAAA A-GTTCTTGT
             CTTTCTAACT AAACC
MAST_B152_   AGTTCTACCC CTATTTTCCC AAGAAGGATC CAATTCCTTC TGAGCTGTGT
             GCTTTCTTAT GCTGACCTGA TATCTGAAAC TTTGCTGAAA AAATTCTTAT
             CTTTCTAACT AAACC
SAV1_SE210   AGCCCTACCC CTATTTTCCC AAGAAGGATC CAATTCCTTC CGAGCTGTGT
             GCTTTCTCAT GCTGACCTGA TATCTGAAAC TTTGCTGAAA A-GTTCTTGT
             CTTTCTAACT AAACC

 1 1 1 1 1
5 116
```

ASIAN2_Ema CTTCCGTAAA GATTATAGCC TGTGGAAACC CTACCCTGTT TTATAGGGCT
GCTATGAGTT GGAACCAACT CAATGGCAAT AGGTTTC-AG GGTTTTTTT-
GTTTTTTT-G GTATCT
FOR_DS1535 CTTCCGTAAA GATTATAGCC TGTGGAAACC CTACCCTGTT TTATAGGGCT
GCTATGAGTT GGAACCAACT CAATGGCAAT AGGTTTC-AG GGTTTTTTT-
GTTTTTTT-G GTATCT
MAM1_SP134 CTTCCGTAAA GATTATAGCC TGTGGAAACC CTACCCTGTT TTATAGGGCT
GCTATGAGTT GGAACCAACT CAATGGCAAT AGGTTTC-AG GGTTTTTTT-
GTTTTTTT-G GTATCT
MAST_B105_ TTTTCGTAAA GATTATAGCC TGTGGAAACC CTACCCTGTT TTATAGGGCT
GCTATGAGTT GGAACCAACT CAATGGCAAT AGGTTTCTAG GGTTTTTTTT
ATTTTTTT-G GTATCT
SAV1_SE210 CTTCCGTAAA GATTATAGCC TGTGGAAACC CTACCCTGTT TTATAGGGCT
GCTATGAGTT GGAACCAACT CAATGGCAAT AGGTTTC-AG GGTTTTTTT-
GTTTTTTT-G GTATCT

1 1 1 1 1
5 115

ASIAN2_Ema TCTGCTGTGA TTTGTGAGTG TGGCTGAGAG CTGGGACCTG TTCTCCCTCC
GTAGAGCTCA CCCTT----C AGGAGGCGGG TTCAAGGCCA TGAGAGCCAA
GCCAGAAGAT GTTCA
FOR_DS1535 TCTGCTGTGA TTTGCGAGTG TGGCTGAGAG CTGGGACCTG TTCTCCCTCC
GTAGAGCTCA CCCTTACTYC AGGAGGCGGG TTCAAGGCCA TGAGAGCCAA
GCCAGAAGAT GTTCA
MAM1_SP134 TCTGCTGTGA TTTGCGAGTG TGGCTGAGAG CTGGGACCTG TTCTCCCTCC
GTAGAGCTCA CCCTTACTTC AGGAGGCGGG TTCAAGGCCA TGAGAGCCAA
GCCAGAAGAT GTTCA
MAST_B106_ TCTGCTGTGA TTTGCGAGTG TGGCTGAGAG CTGGGACCTG TTCTCCCTCC
GTAGAGCTCA CCCTTACTTC AGGAGACAAA TTCAAGGCCA TGAAAGCCAA
GCCAGAAAAT ATTCA
SAV1_SE210 TCTGCTGTGA TTTGCGAGTG TGGCTGAGAG CTGGGACCTG TTCTCCCTCC
GTAGAGCTCA CCCTTACTTC AGGAGGCGGG TTCAAGGCCA TGAGAGCCAA
GCCAGAAGAT GTTCA

1 1 1 1 1
5 115

ASIAN2_Ema GAAGCCGACT GCTGAGCCC- TGCAGCGTGG TCACTCATCA ACGATGACCA
TACTCTTGGC TCCAATTCTC TGTGAAGGGC TTGCACTCTC TGT------G
AATT--ATCA -AGAA
FOR_DS1535 GAAGCCGACT GCTGAGCCC- TGCAGCGTGG TCACTCATCA ACRATGACCA
TACTCTTGGC TCCAATTCTC TGTGAAGGGC TTGCACTCTC TGT------G
AATT--MTCA -AGAA
MAM1_SP134 GAAGCCGACT GCTGAGCCC- TGCAGCGTGG TCACTCATCA ACGATGACCA
TACTCTTGGC TCCAATTCTC TGTGAAGGGC TTGCACTCTC TGT------G
AATT--ATCA -AGAA
MAST_B107_ GAAGTTGATT GCTGAACCCC TGCAGCGTGG TCACTCATCA ACGATGACCA
TACTCTTGGC TTTAATTCTC TGTGAAGGGC TTGCACTCTC TGTTCAGGCG
TATGCTATCC TACAA
SAV1_SE210 GAAGCCGACT GCTGAGCCC- TGCAGCGTGG TCACTCATCA ACGATGACCA
TACTCTTGGC TCCAATTCTC TGTGAAGGGC TTGCACTCTC TGT------G
AATT--ATCA -AGAA

1 1 1 1 1
5 118

ASIAN2_Ema TCAGTGATTC TAAATATGAA ACCWTCTCCC AGCTCTGCTC TGGAGCTGTC
AGTTTGCTGT TCATAGCATC GGCAACACCT ACCACATCCA CGAATGTCGC
CGAGGAGGAG GCATATCG
FOR_DS1535 TCAGTGATTC TAAATATGAA ACCATCTCCC AGCTCTGCTC TGGAGCTGTC
AGTTTGCTGT TCATAGCATC GGCAACACCT ACCACATCCA CGAATGTCGC
CGAGGAGGAG GCATATCG
MAM1_SP134 TCAGTGATTC TAAATATGAA ACCATCTCCC AGCTCTGCTC TGGAGCTGTC
AGTTTGCTGT TCATAGCATC GGCAACACCT ACCACATCCA CGAATGTCGC
CGAGGAGGAG GCATATCG
MAST_B154_ TTAGTGATTC TAAATATGAA ACCATCTCCC AGCTCTGCTC TGGAGCTGTC
AGTTTGCTGT TCATAGCATC GGCAACACCT AC-ACATC-A CGAATGTCGC
-GAAGAGGAG GCATATCA
SAV1_SE210 TCAGTGATTC TAAATATGAA ACCATCTCCC AGCTCTGCTC TGGAGCTGTC

AGTTTGCTGT TCATAGCATC GGCAACACCT ACCACATCCA CGAATGTCGC
CGAGGAGGAG GCATATCG

 1 1 1 1 1
5 114

ASIAN2_Ema CCTTCGTCTC ATGCATTATG AGGAGTTCTG GCCACCTTGG CTGCCATGCT
GAGTGCCACC TGCCCACAGT ACACCCCGAG GCCKAGATCT GCTGCCTTTG
CTTTTCAAAG GGTG
FOR_DS1535 CCTTCGTCTC ATGCATTATG AGGAGTTCTG GCCACCTTGG CTGCCATGCT
GAGTGCCACC TGCCCACAGT ACACCCCGAG GCCRAGATCT GCTGCCTTTG
CTTTTCAAAG GGTG
MAM1_SP134 CCTTCGTCTC ATGCATTATG AGGAGTTCTG GCCACCTTGG CTGCCATGCT
GAGTGCCACC TGCCCACAGT ACACCCCGAG GCCGAGATCT GCTGCCTTTG
CTTTTCAAAG GGTG
MAST_B156_ CCTTCGTCTC ATGCATTATA AGGAGTTCTG GCCACCTTGG CTGCCATGCT
GGGTGCCACC TGCCCACAGT ACACCCCGAG GCCGAGATCT GCTGCCTTTG
CTTTTCAAAG GGTA
SAV1_SE210 CCTTCGTCTC ATGCATTATG AGGAGTTCTG GCCACCTTGG CTGCCATGCT
GAGTGCCACC TGCCCACAGT ACACCCCGAG GCCGAGATCT GCTGCCTTTG
CTTTTCAAAG GGTG

 1 1 1 1 1
5 116

ASIAN2_Ema TAAAGTGGCC TTGGTGCAAT GGCAAAAATA TGAAGCCGAC AACGATTCTG
AAGCAGTGAA GTTCCTGGAG ATCACATGGC AGGAGCCGTC -CTG------
-------AAA TACATC
FOR_DS1535 TAAAGTGGCC TTGGTGCAAT GGCAAAAATA TGAAGCCGAC AACGATTCTG
AAGCAGTGAA GTTCCTGGGG ATCACATGGC AGGAGCCGTC -CTG------
-------AAA TACATC
MAM1_SP134 TAAAGTGGCC TTGGTGCAAT GGCAAAAATA TGAAGCCGAC AACGATTCTG
AAGCAGTGAA GTTCCTGGGG ATCACATGGC AGGAGCCGTC -CTG------
-------AAA TACATC
MAST_B109_ TAAAGTGGCC TTGGTGCAAT GGCAAAAATA TGAAGCCGAC AACGATTCTG
AAGCAGTGAA GTTCTTGGGG GTGG-AGGG- ATGTTTCTTC TCTGCAGAAA
GGGCAAAAAA TACCTT
SAV1_SE210 TAAAGTGGCC TTGGTGCAAT GGCAAAAATA TGAAGCCGAC AACGATTCTG
AAGCAGTGAA GTTCCTGGGG ATCACATGGC AGGAGCCGTC -CTG------
-------AAA TACATC

 1 1 1 1 1
5 114

ASIAN2_Ema TTTATTAGGT GAGTGGCTTG CCGACCCCAG AGCTGACATG GCTGCTCAAT
GGCCAACCTG TGCTTCCAGA CGCCTCACAC AAGATGCTGG TCAGGGAAAC
TGGAGTCCAC TCTC
FOR_DS1535 TTTATTAGGT GAGTGGCTTG CCGACCCCAG AGCTGACATG GCTGCTCAAT
GGCCAACCTG TGCTTCCAGA CGCCTCACAC AAGATGCTGG TCAGGGAAAC
TGGAGTCCAC TCTC
MAM1_SP134 TTTATTAGGT GAGTGGCTTG CCGACCCCAG AGCTGACATG GCTGCTCAAT
GGCCAACCTG TGCTTCCAGA CGCCTCACAC AAGATGCTGG TCAGGGAAAC
TGGAGTCCAC TCTC
MAST_B110_ TTTATTAGGT GAGTGGCTTG CCGACCCCAG AGCTGACATG GCTGCTCAAT
GGCCAACCTG TGCTTCCAGA CGCCTCACAC AAGATGCTGG TCAGGGAAAC
TGGAGTCCAC TCTC
SAV1_SE210 TTTATTAGGT GAGTGGCTTG CCGACCCCAG AGCTGACATG GCTGCTCAAT
GGCCAACCTG TGCTTCCAGA CGCCTCACAC AAGATGCTGG TCAGGGAAAC
TGGAGTCCAC TCTC

 1 1 1 1 1
5 114

ASIAN2_Ema TTTSTATTTC ATTGCAAGAT TCTGCCTTTC ATACATATGA CCCACAATTT
CCCAYTTGAC CACAGCRAAA GGACAAACCT CATCACAGCC ATTAGGGTAC
TTCCAGTTTG TCTT
FOR_DS1535 TTTCTATTTC ATTGCAAGAT TCTGCCTTTC ATACATATGA CCCACAATTT
CCCATTTGAC CACAGCGAAA GGACAAACCT CATCACAGCC ATTAGGGTAC
TTCCAGTTTG TCTT
MAM1_SP134 TTTGTGTTTC ATTGCAAGAT TCTGCCTTTC ATACATATGA CCCACAATTT
CCCATTTGAC CACAGCAAAA GGACAAACCT CATCACAGCC ATTAGGGTAC

```
            TTCCAGTTTG TCTT
MAST_B111_  TTTGTATTTC ATTGCAAGAT TCTGCCTTTC ATACATATGA CCCACAATTT
            CCCATTTGAC CACAGCAAAA GGACAAACCT CATCACAGCC ATTAGGGTAC
            TTCCAGTTTG TCTT
SAV1_SE210  TTTGTATTTC ATTGCAAGAT TCTGCCTTTC ATACATATGA CCCACAATTT
            CCCATTTGAC CACAGCGAAA RGACAAACCT CATCACAGCC ATTAGGGTAC
            TTCCGGTTTG TCTT

 1 1 1 1 1
5 114

ASIAN2_Ema  CTGCAGACAG GCCTAGAACG AAGGCTGCAT GTCCCTGCCT GGGCTGCTTG
            CTGTCAGGAA ACAGGACAGG GGGCCGGCGT CCTCCAAGGA CTGTCACGTG
            GAGAGCAAAT AGGA
FOR_DS1535  CTGCAGACAG GCCTAGAATG AAGGCTGCAT GTCCCTGCCT GGGCTGCTTG
            CTGTCAGGAA ACCGGACAGG GGGCCGGCGT CCTCCAAGGA CTGTCATGTG
            GAGAGCAAAT AGGA
MAM1_SP134  CTGCAGACAG GCCTAGAACG AAGGCTGCAT GTCCCTGCCT GGGCTGCTTG
            CTGTCAGGAA ACAGGACAGG GGGCCGGCGT CCTCCAAGGA CTGTCACGTG
            GAGAGCAAAT AGGA
MAST_B112_  CTGCAGACAG GCCTAGAATG AAGGCTGCAT GTCCCAGCCT GGGCTGCTTG
            CTGTCAGGAA ACTGGACAGG GAGCCGGCGT CCTCCAAGGA CTGTCACGTA
            GAAAACAAAT AGAA
SAV1_SE210  CTGCAGACAG GCCTAGAACG AAGGCTGCAT GTCCCTGCCT GGGCTGCTTG
            CTGTCGGGAA ACCGGACAGG GGGCCGGCGT CCTCCAAGGA CTGTCATGTG
            GAGAGCAAAT AGGA

 1 1 1 1 1
5 114

ASIAN2_Ema  GTGCAACCAT TACCGTGTTC CCAGCCTGTG AAGGAGAGCT GGATAGCCAA
            TGCTGTTTTA CAACCTGCAA TTCCATTAAT ACTGAAAAAG AAGGTACTGA
            CCTTGACAAC AATG
FOR_DS1535  GTGCAACCAT TACCGTGTTC CCAGCCTGTG AAGGAGAGCT GGATAGCCAA
            TGCCGTTTTA CAACCTGCAA TTCCATTAAT ACTGAAAAAG AAGGTACTGA
            CCTTGACAAC AATG
MAM1_SP134  GTGCAACCAT TACCGTGTTC CCAGCCTGTG AAGGAGAGCT GGATAGCCAA
            TGCCGTTTTA CAACCTGCAA TTCCATTAAT ACTGAAAAAG AAGGTACTGA
            CCTTGACAAC AATG
MAST_B157_  GTGCAACCAT TACCGTGTTC CCGGCTTGTG AAGGAGAGCT GGATAGCCAA
            TGCCGTTTTA CAACCTGCAA TTCCATTAAT ACTGAAAAAG AAGGTACTGA
            CCTTGACAAC AATG
SAV1_SE210  GTGCAACCAT TACCGTGTTC CCAGCCTGTG AAGGAGAGCT GGATAGCCAA
            TGCCGTTTTA CAACCTGCAA TTCCATTAAT ACTGAAAAAG AAGGTACTGA
            CCTTGACAAC AATG

 1 1 1 1 1
5 114

ASIAN2_Ema  TTTCCATGTA CTGAAGGCTT G-CTCTCAGA AGCTGGGGAG CTGGGAGAAC
            GGGGAGCCCA TGGCACGTTA GTTGTCTGGA AAAGCCCATG AGGCAGCCAA
            -CAGCTGCTC ATCC
FOR_DS1535  CTTCCATGTA CTGAAGGCTT G-CTCTCAGA AGCTGGGGAG CTGGGAGAAC
            GGGGAGCCCA TGGCACGTTA GTTGTCTGGA AAAGCCCATG AGGCAGCCAA
            -CGGCTGCTC ATCC
MAM1_SP134  TTTCCATGTA CTGAAGGCTT G-CTCTCAGA AGCTGGGGAG CTGGGAGAAC
            AGGGAGCCCA TGGCACGTTA GTTGTCTGGA AAAGCCCATG AGGCAGCCAA
            -CAGCTGCTC ATCC
MAST_B158_  TTTTCATGTA CTGAAGGCTT GTCTCTCAGA AGCTGGGG-G CTGGGAGAAC
            GGGGAGCCCA TGGCACATTA GTTGTCTGGA AAAGCCCATG AGGCAGCTAA
            ACAGCTGCTC ATCC
SAV1_SE210  CTTCCATGTA CTGAAGGCTT G-CTCTCAGA AGCTGGGGAG CTGGGAGAAC
            GGGGAGCCCA TGGCACGTTA GTTGTCTGGA AAAGCCCATG AGGCAGCCAA
            -CGGCTGCTC ATCC

 1 1 1 1 1
5 116

ASIAN2_Ema  CACCACGTAT AAGAATTTTA CACCTAGAAC CATAAAACTT AGGATACAGA
            ACCCACGGTT CTGCTGAGAC TGTGAGGATT GAGAAAGTGA TGTGGCTTTT
            AAGCTGAAAC ACCAAT
```

```
FOR_DS1535    CACCACGTAT AAGAATTTTA CACCTAGAAC CATAAAACTT AGGATACAGA
              ACCCACGGTT CTGCTGAGAC TGTGAGGATT GAGAAAGTGA TGTGGCTTTT
              AAGCTGAAAC ACCAAT
MAM1_SP134    CACCACGTAT AAGAATTTTA CACCTAGAAC CATAAAACTT AGGATACAGA
              ACCCACGGTT CTGCTGAGAC TGTGAGGATT GAGAAAGTGA TGTGGCTTTT
              AAGCTGAAAC ACCAAT
MAST_B159_    CACCAGGTAT AAGAATTTTA CACCTAGAAC -ATGAA-CTT AG-ATACAGA
              ACCTACGGTT CTGCTGAGAC TGTGAGGATT GAGAAAGTGA TGTGGCTTTT
              AAGCTGAAAC ACCAAT
SAV1_SE210    CACCACGTAT AAGAATTTTA CACCTAGAAC CATAAAACTT AGGATACAGA
              ACCCACGGTT CTGCTGAGAC TGTGAGGATT GAGAAAGTGA TGTGGCTTTT
              AAGCTGAAAC ACCAAT

 1 1 1 1 1
5 113

ASIAN2_Ema    TCGATTCTGA CTCATAGCGA CCCTATAGGA CAGAGTAGAA CTGCCCCAC-
              AGAGTTTCCA AGGAGCACCT GGCAGATTTG AAGTGCCGAC CCTTTGATTA
              GCAGCCGTAG CAC
FOR_DS1535    TCGATTCTGA CTCATAGCGA CCCTATAGGA CAGAGTAGAA CTGCCCCAC-
              AGAGTTTCCA AGGAGCACCT GGCAGATTTG AACTGCCGAC CCTTTGATTA
              GCAGCYGTAG CAC
MAM1_SP134    TCGATTCTGA CTCATAGCGA CCCTATAGGA CAGAGTAGAA CTGCCCCAC-
              AGAGTTTCCA AGGAGCACCT GGCAGATTTG AAGTGCCGAC CCTTTGATTA
              GCAGCCGTAG CAC
MAST_B114_    TCGATTCTGA CACGTAGCGA CCCTACAGGA CAGAGCAGAA CTGCCCCACC
              AGAGTTTCTA AGGAGCTCTT GACAGATTTG AACTGCCGAC CCTTTAATTA
              GCATCTGTAG CAC
SAV1_SE210    TCGATTCTGA CTCATAGCGA CCCTATAGGA CAGAGTAGAA CTGCCCCAC-
              AGAGTTTCCA AGGAGCACCT GGCAGATTTG AACTGCCGAC CCTTTGATTA
              GCAGCTGTAG CAC

  1 1 1 1 1
5 143

ASIAN2_Ema    CCTGCCATGC TCAGCATTTG TGAACACGTA CCAAGAGCCG GCACTAGAAA
              AAAAAAAAAW CCAAGAGCCT ACTGTGGACA GAGTATTAGC CACCCAGTAA
              GCACTCAGTA AATGTCAGTT GTTTAGTTGC TTTGTTGTTG CCA
FOR_DS1535    CCTGCCATGC TCAGCATTTG TGAACACGTA CCAAGAGCC- ----------
              ---------- ---------T ACTGTGGACA GAGTATTAGC CACCCRGTAA
              GCACTCAGTA AATGTCAGTT GTTTAGTTGC TTTGTTGTTG CCA
MAM1_SP134    CCTGCCATGC TCAGCATTTG TGAACACGTA CCAAGAGCC- ----------
              ---------- ---------T ACTGTGGACA GAGTATTAGC CACCCAGTAA
              GCACTCAGTA AATGTCAGTT GTTTAGTTGC TTTGTTGTTG CCA
MAST_B116_    TTTGTTATGC TCAGCATTTG TGAACACGTA CCAAGAGCC- ----------
              ---------- ---------T ACTGTGGACA GAGTATTAGC CACCCAGTAA
              GCACTCAGTA AATGTCAGTT GTTTAGTTGC ATTGTTGTTG CCA
SAV1_SE210    CCTGCCATGC TCAGCATTTG TGAACACGTA CCAAGAGCC- ----------
              ---------- ---------T ACTGTGGACA GAGTATTAGC CACCCAGTAA
              GCACTCAGTA AATGTCAGTT GTTTAGTTGC TTTGTTGTTG CCA

 1 1 1 1 1
5 113

ASIAN2_Ema    GATCTGGAGG GATTTCT-CT T-ACTTT-TT -CAGCGAGGA AAGACACTGG
              GTGAGCCCCT TTTTGGGTGG CGATGAGGGA GATGGGTCTT CATCAGATGG
              ATGTTTTGAG ATG
FOR_DS1535    GATCTGGAGG GATTTCT-CT T-ACTTT-CT -CAGYGAGGA AAGACACTGG
              GTGAGCCCCT TTTTGGGTGG CGATGAGGGA GATGGGTCTT CATCAGATGG
              ATGTTTTGAG ATG
MAM1_SP134    GATCTGGAGG GATTTCT-CT T-ACTTT-CT -CAGCGAGGA AAGACACTGG
              GTGAGCCCCT TTTTGGGTGG CGATGAGGGA GATGGGTCTT CATCAGATGG
              ATGTTTTGAG ATG
MAST_B163_    ATTTTGGAGG GATTTCTTCT TTACTTTTCT TCAGCGAGGA AAGACACTGG
              GTGAGCCCCT TTTTGGGTGG CGATGAGG-A GATGGGTCTT CATCAGATGG
              ATGTTTTGAG ATG
SAV1_SE210    GATCTGGAGG GATTTCT-CT T-ACTTT-CT -CAGCGAGGA AAGACACTGG
              GTGAGCCCCT TTTTGGGTGG CGATGAGGGA GATGGGTCTT CATCAGATGG
              ATGTTTTGAG ATG

 1 1 1 1 1
```

5 133

```
ASIAN2_Ema   GAGTC-TGAT AAGATTAGGA CAGAAAAAGC CACCTTT-GT TTGAAGGTAC
             ATAGGCCTCA CCACCTCAGG AGAAACGGCT GCTCCGCATA GGTGGTCACG
             CCCAGCCGGC CAAAGTTCTT TGGGCTGGGA ATT
FOR_DS1535   GAGTC-TGAT AAGATTAGGA CAGAAAAAGC CACCTTT-GT TTGAAGGTAC
             ATAGGCCTCA CCACCTCAGG AGAAACGGCT GCTCCGCATA GGTGGTCACG
             CCCAGCCGGC CAAAGTTCTT TGAGCTGGGA ATT
MAM1_SP134   GAGTC-TGAT AAGATTAGGA CAGAAAAAGC CACCTTT-GT TTGAAGGTAC
             ATAGGCCTCA CCACCTCAGG AGAAACGGCT GCTCCGCATA GGTGGTCACG
             CCCAGCCGGC CAAAGTTCTT TGAGCTGGGA ATT
MAST_B120_   GAGTCCTGAT AAGATTAGGA CAGAAAAAGG CACCTTTTGT TTGAAGGTAC
             ATAGGCCTCA CCACCTCA-- ---------- --------TA GGTGGTCAGG
             CCCAGCCAGC CAAA-TTCTT TGAGCTAGGA ATT
SAV1_SE210   GAGTC-TGAT AAGATTAGGA CAGAAAAAGC CACCTTT-GT TTGAAGGTAC
             ATAGGCCTCA CCACCTCAGG AGAAACGGCT GCTCCGCATA GGTGGTCACG
             CCCAGCCGGC CAAAGTTCTT TGAGCTGGGA ATT
```

1 1 1 1 1
5 112

```
ASIAN2_Ema   AAACTCCTGG ACTGGGGCCT GGCTGGAGCT TTTGGGAACC ACTCCCTGCC
             CATCCCAGGC TCCTGGCTCT ATTCCAGGGG TTGGATGAGG CTGCTCGCCT
             ACTCACAGAC AC
FOR_DS1535   AAACTCCTGG ACTGGGGCCT GGCTGGAGCT TTTGGGAACC ACTCCCTGCC
             CATCCCAGGC TCCTGGCTCT ATTCCAGGGG TTGGATGAGG CTGCTCGCCT
             ACTCACAGAC AC
MAM1_SP134   AAACTCCTGG ACTGGGGCCT GGCTGGAGCT TTTGGGAACC ACTCCCTGCC
             CATCCCAGGC TCCTGGCTCT ATTCCAGGGG TTGGATGAGG CTGCTCGCCT
             ACTCACAGAC AC
MAST_B166_   AAACTCCTGG ACTGGGGCTT GGCTGGAGCT TTTGGAAACC ACTCCCTGCC
             CATCCCAGGC TCCTGGCTCT ATTCCAGGGG TTGGATGAGG CTGCTCGCCT
             ACTCACAGAC AC
SAV1_SE210   AAACTCCTGG ACTGGGGCCT GGCTGGAGCT TTTGGGAACC ACTCCCTGCC
             CATCCCAGGC TCCTGGCTCT ATTCCAGGGG TTGGATGAGG CTGCTCGCCT
             ACTCACAGAC AC
```

1 1 1 1 1
5 114

```
ASIAN2_Ema   TGGAGAAGGT GGGGACAGGG T-GACCACAG AACCTACGAG CAAACCTGAA
             ATGCCCTGTA GATTTTGAGA ATCAAGTGAG GCAGATCTAC GAAAAATAAG
             AAAAAATACT TCTC
FOR_DS1535   TGGAGAAGGT GGGGACAGGG T-GACCACAG AACCTACGAG CAAACCTGAA
             ATGCCCTGTA GATTTTGAGA ATCAAGTGAG GCAGATCTAC GAAAAATAAG
             AAAAAATACT TCTC
MAM1_SP134   TGGAGAAGGT GGGGACAGGG T-GACCACAG AACCTACGAG CAAACCTGAA
             ATGCCCTGTA GATTTTGAGA ATCAAGTGAG GCAGATCTAC GAAAAATAAG
             AAAAAATACT TCTC
MAST_B167_   TGGAGAAGGT GGGGACAGGG T-GACCACAG AACCTACGAG GAAACCTGAA
             ATGCATAGTA GATTTTGAGA ATCAAGTGAG GCAGATCTAC GAAAAATAAG
             AAAAA-TACT TCTC
SAV1_SE210   TGGAGAAGGT GGGGACAGGG T-GACCACAG AACCTACGAG CAAACCTGAA
             ATGCCCTGTA GATTTTGAGA ATCAAGTGAG GCAGATCTAC GAAAAATAAG
             AAAAAATACT TCTC
```

1 1 1 1 1
5 111

```
ASIAN2_Ema   CACTCACCTC CTCCTAACCT CATAGCTGGA GTCCCTGGAT GCCTCTGAAC
             CTGAAGAGAA AGCTGAGGAC TSCTGGGAGC CACAGATTAG CCCAGAGCTG
             CTGGCCCATG G
FOR_DS1535   CACTCACCTC CTCCTAACCT CATAGCTGGA GTCCCTGGAT GCCTCTGAAC
             CTGAGGAGAA AGCTGAGGAC TGCTGGGAGC CACAGATTAG CCCAGAGCTG
             CTGGCCCATG G
MAM1_SP134   CACTCACCTC CTCCTAACCT CATAGCTGGA GTCCCTGGAT GCCTCTGAAC
             CTGAGGAGAA AGCTGAGGAC TGCTGGGAGC CACAGATTAG CCCAGAGCTG
             CTGGCCCATG G
MAST_B121_   CACTCACGTC CTCCTAACCT CATAGCTGGA GTCCCTAGAT GCCTCTGAAC
             CTGAGGAGAA AGCTGAGGAC TGCTGGGAGC CACAGATTAG CCCAGAGCTG
             CTGGCCCATA A
```

```
SAV1_SE210   CACTCACCTC CTCCTAACCT CATAGCTGGA GTCCCTGGAT GCCTCTGAAC
             CTGAGGAGAA AGCTGAGGAC TGCTGGGAGC CACAGATTAG CCCAGAGCTG
             CTGGCCCATG G

 1 1 1 1 1
5 114

ASIAN2_Ema   CCCCAGGAGG AAGGGGA-CA CACTCCTCTT TGGGCCCTGT -CTCTCCCAG
             CTCTGCCCTC TGTAGTAGTT TCCAAGCCCA GGACCGAGTC AGGGTGTGTT
             CTAGATACCA ACCT
FOR_DS1535   CCCCAGGAGG AAGGGGA-CA CACTCCTCTT TGGGCCCTGT -CTCTCCCAG
             CTCTGCCCTC TGTAGTAGTT TCCAAGCCCA GGACCGAGTC AGGGTGTGTT
             CTAGATACCA ACCT
MAM1_SP134   CCCCAGGAGG AAGGGGA-CA CACTCCTCTT TGGGCCCTGT -CTCTCCCAG
             CTCTGCCCTC TGTAGTAGTT TCCAAGCCCA GGACCGAGTC AGGGTGTGTT
             CTAGATACCA ACCT
MAST_B122_   TCCCAGGAGG AAGGGGACCA CACTCCTCCT TTGGCCCTGT CCTCT-CCAG
             CTCTG-CCTC TGTAGTAGTT TCCAAGCCCA GGACCGAGTC AGGGTGTGTT
             -TAGATACCA ACCT
SAV1_SE210   CCCCAGGAGG AAGGGGA-CA CACTCCTCTT TGGGCCCTGT -CTCTCCCAG
             CTCTGCCCTC TGTAGTAGTT TCCAAGCCCA GGACCGAGTC AGGGTGTGTT
             CYAGATACCA ACCT

 1 1 1 1 1
5 111

ASIAN2_Ema   CAAGCTTCTG AGAAAGGTGC TCTGGGCTCA TTTTACAGGT GTCAGA-GCC
             GAAGGTCAGT GTGGCTACTC ACCTTAACCA AGTAGTGAGG GATGAGGCCC
             AAAGGAAGGC T
FOR_DS1535   CAAGCTTCTG AGAAAGGTGC TCTGGGCTCA TTTTACAGGT GTCAGA-GCC
             GAAGGTCAGT GTGGCTACTC ACCTTAACCA AGTAGTGAGG GATGAGGCCC
             AAAGGAAGGC T
MAM1_SP134   CAAGCTTCTG AGAAAGGTGC TCTGGGCTCA TTTTACAGGT GTCAGA-GCC
             GAAGGTCAGT GTGGCTACTC ACCTTAACCA AGTAGTGAGG GATGAGGCCC
             AAAGGAAGGC T
MAST_B123_   TAAGCTTCTG AGAAAGGTGC TCTGGGCTCA TTTTACAAAT ATCAAAAGCC
             GAAGATCAAT ATAGCTACTC ACCTTAACCA AGTAGTGAGG AATGAGGCCC
             AAAGGAAGGC T
SAV1_SE210   CAAGCTTCTG AGAAAGGTGC TCTGGGCTCA TTTTACAGGT GTCAGA-GCC
             GAAGGTCAGT GTGGCTACTC ACCTTAACCA AGTAGTGAGG GATGAGGCCC
             AAAGGAAGGC T

 1 1 1 1 1
5 111

ASIAN2_Ema   AGACCTGAGC TCACATCCTG GTTCTGCTAG CTCTAAGACA TAGGCAGCCT
             GAGGCCTCAG TTTCCACATC TGTAAAATGC AGGCAGTGAC CCCATCTTCA
             CAGGTCTAGT T
FOR_DS1535   AGACCTGAGC TCACATCCTG GTTCTGCTAG CTCTAAGACA TAGGCAGCCT
             GAGGCCTCAG TTTCCACATC TGTAAAATGC AGGCAGTGAC CYCATCTTCA
             CAGGTCTAGT T
MAM1_SP134   AGACCTGAGC TCACATCCTG GTTCTGCTAG CTCTAAGACA TAGGCAGCCT
             GAGGCCTCAG TTTCCACATC TGTAAAATGC AGGCAGTGAC CCCATCTTCA
             CAGGTCTAGT T
MAST_B169_   AGATCTGAGC TCACATCCTG GTTCTGCTAG CTCTAAGACA TAGGCAGCAT
             GAGGCCTCAG TTTCCACATC TGTAAAATGC AGGCAGTGAC CCCATCTTCA
             CAAGTCTAGT T
SAV1_SE210   AGACCTGAGC TCACATCCTG GTTCTGCTAG CTCTAAGACA TAGGCAGCCT
             GAGGCCTCAG TTTCCACATC TGTAAAATGC AGGCAGTGAC CCCATCTTCA
             CAGGTCTAGT T

 1 1 1 1 1
5 111

ASIAN2_Ema   GCAA-GAGTC ATAGTCCTTG TAAGAGGAGA AACAGGTAAT ATGACTTGTC
             GG-AGAAA-C AAA-GAAC-A AAA-TTGTCC ATTGGGATGG AATAGAAGAT
             CCTATTCCTA A
FOR_DS1535   GCAA-GAGTC ATAGTCCTTG TAAGAGGAGA AACAGGTAAT ATTACTTGTT
             GG-AGAAA-C AAA-GAAC-A AAA-TTGTCC ATTGRGATGG AATAGAAGAT
             CCTATTCCTA A
MAM1_SP134   GCAA-GAGTC ATAGTCCTTG TAAGAGGAGA AACAGGTAAT ATTACTTGTT
```

GG-AGAAA-C AAA-GAAC-A AAA-TTGTCC ATTGGGATGG AATAGAAGAT
CCTATTCCTA A
MAST_B170_ GCAAAGAGTC ATAGTCCTTG TAAGAGGAGA AACAGGTAAT ATTACTTGTC
GGGAGAAAAC AAAAGAACCA AAAGTTGTCC ATTGGGATGG AGTAGAAAAT
CCTATTCCTA A
SAV1_SE210 GCAA-GAGTC ATAGTCCTTG TAAGAGGAGA AACAGGTAAT ATGACTTGTC
GG-AGAAA-C AAA-GAAC-A AAA-TTGTCC ATTGGAATGG AATAGAAGAT
SCTATTCCTA A

1 1 1 1 1
5 111

ASIAN2_Ema TGTTCCTAAG CTAT-GCTTC TGGGGTCCTT CAAACCTCTG TG-CATGATT
CTGCCTYCCT AGATTTTTC- ACTCTGGATG GAGGCAGAAT GTGAGGTGGG
GGTCTTCTCA G
FOR_DS1535 TGTTCCTAAG CTAT-GCTTC TGGGGTCCCT CAAACCTCTG TG-CATGATT
CTGCCTCCCT AGATTTTTC- ACTCTGGATG GAGGCAGAAT GTGAGGTGGG
GGTCTTCTCA G
MAM1_SP134 TGTTCCTAAG CTAT-GCTTC KGGGGTCCCT CAAACCTCTG TG-CATGATT
CTGCCTCCCT AGATTTTTC- ACTCTGGATG GAGGCAGAAT GTGAGGTGGG
GGTCTTCTCA G
MAST_B171_ TGTTCCTAAG CTATCGCTTC TGGGGTCCCT CAAACCTCTG TGTCATGATT
CTGCTTCCCT AGATTTTTCC ACTCTGGATG GAGGCAGAAT GTGAGGTGGA
GGTCTTCTCA G
SAV1_SE210 TGTTCCTAAG CTAT-GCTTC TGGGGTCCCT CAAACCTCTG TG-CATGATT
CTGCCTCCCT AGATTTTTC- ACTCTGGATG GAGGCAGAAT GTGAGGTGGG
GGTCTTCTCA G

1 1 1 1 1
5 111

ASIAN2_Ema TGTCTTAGAT AACACTTCTG TTTCTGAGAA AGTGACCCAT GACTGTTAGC
AAGTGAACCA AGGAGGGGCA GCTTGCCTGC AGGGAAAACA CTTAGTATAT
TTTAGACAGA T
FOR_DS1535 TGTCTTAGAT AACACTTCTG TTTCTGAGAA AGTGACCCAT GACTGTTAAC
AAGTGAACCA AGGAGGGGCA GCTTGCCTGC AGGGAAAACA CTTASTATAT
TTTAGACAGA T
MAM1_SP134 TGTCTTAGAT AACACTTCTG TTTCTGAGAA AGTGACCCAT GACTGTTAAC
AAGTGAACCA AGGAGGGGCA GCTTGCCTGC AGGGAAAACA CTTAGTATAT
TTTAGACAGA T
MAST_B172_ TGTCTTAGAT AACACTTCTG TTTCTAAGAA AGTGACCCAT GACTGTTAAC
AAGTGAACCA AGGAGGGGCA GCTTGCCTGC AGAGAAAACA CTTAATATAT
TTTAGACAGA T
SAV1_SE210 TGTCTTAGAT AACACTTCTG TTTCTGAGAA AGTGACCCAT GACTGTTAAC
AAGTGAACCA AGGAGGGGCA GCTTGCCTGC AGGGAAAACA CTTACTATAT
TTTAGACAGA T

1 1 1 1 1
5 111

ASIAN2_Ema CCAAAGACGC CTGTTCCAGC TGCAAGGAGG TCAGCATTCC AAGGGGAGAG
CTGGAGGAAA CACTTGGCCA CTCAAAGGTA GACCAGGATG AAGGA-GAAA
AAAA-GCCCA G
FOR_DS1535 CCAAAGACGC CTGTTCCAGC TGCAAGGAGG TCAGCATTCC AAGGGGAGAG
CTGGAGGAAA CACTTGGCCA CTCAAAGGTA GACCGGGATG AAGGA-GAAA
AAAA-GCCCA G
MAM1_SP134 CCAAAGACGC CTGTTCCAGC TGCAAGGAGG TCAGCATTCC AAGGGGAGAG
CTGGAGGAAA CACTTGGCCA CTCAAAGGTA GACCAGGATG AAGGA-GAAA
AAAA-GCCCA G
MAST_B126_ CTAAAGATGC CTGTTCCAGC TGCAAGGAGG TCAGCATTCC AAGGGGAGAG
CTGGAGAAAA CACTTGGCCA CTCAAAGGTA GACCAGGATG AAGGAAGAAA
AAAAAGCCCA A
SAV1_SE210 CCAAAGACGC CTGTTCCAGC TGCAAGGAGG TCAGCATTCC AAGGGGAGAG
CTGGAGGAAA CACTTGGCCA CTCAAAGGTA GACTGGGATG AAGGA-GAAA
AAAA-GCCCA G

1 1 1 1 1
5 110

ASIAN2_Ema GTCCTTGAGC ATAGAGAATG TGATTCAGCT GGAGATGGGC TCACAAGGAC
TCACAAGGAC ACATCAACTG GGCCCTGAGA TATAAAGACA ATACCTAGGA

```
          CAACCTTGGA
FOR_DS1535   GTCCTTGAGC ATAGAGAATG TGATCCAGCT GGAGATGGGC TCACAAGGAC
          TCACAAGGAC ACATCAACTG GGCCCTGAGA TATAAAGACA ATACCTAGGA
          CAACCTTGGA
MAM1_SP134   GTCCTTGAGC ATAGAGAATG TGATTCAGCT GGAGATGGGC TCACAAGGAC
          TCACAAGGAC ACATCAACTG GGCCCTGAGA TATAAAGACA ATACCTAGGA
          CAACCTTGGA
MAST_B174_   GTCCTTGAGC ATAGAGAATG TGATCCAGCT GGAGATGGGC TCACAAGGAC
          TCACAAGGAC ACATCAACTG GGCCCTGAGA TATAAAGACA ATACCTAGGA
          CAACCTTGGA
SAV1_SE210   GTCCTTGAGC ATAGAGAATG TGATCCAGCT GGAGATGGGC TCACAAGGAC
          TCACAAGGAC ACATCAACTG GGCCCTGAGA TATAAAGACA ATACCTAGGA
          CAACCTTGGA

 1 1 1 1 1
5 110

ASIAN2_Ema   TTCATYCTCT TCTTATTAAC CCCTTCCTCC TTCCATGCCT TC-ATAGARG
          ATACAGACAA ACACTTCTAA TAGGAAGATA CATGTCCTAC TCATTATAAA
          CAAGGCAGGC
FOR_DS1535   TTCATCCTCT TCTTATTAAC CCCTTCCTCC TTCCATGCCT TC-ATAGAGG
          ATACAGACAA ACACTTCTAA TAGTAAGATA CATGTCCTAC TCATTATAAA
          CAAGGCAGGC
MAM1_SP134   TTCATTCTCT TCTTATTAAC CCCTTCCTCC TTCCATGCCT TC-ATAGAGG
          ATACAGACAA ACACTTCTAA TAGGAAGATA CATGTCCTAC TCATTATAAA
          CAAGGCAGGC
MAST_B175_   TTCATCCTCT TCTTATTAAC CCCTTCCTCC TTCCATGCCT TCCATAGAGG
          ATACAGACAA ACACTTCTAA TAGGAAGATA CATGTCCTAC TCATTATAAA
          CAAGGCAGGC
SAV1_SE210   TTCATCCTCT TCTTATTAAC CCCTTCCTCC TTCCATGCCT TC-ATAGAGG
          ATACAGACAA ACACTTCTAA TAGTAAGATA CATGTCCTAC TCATTATAAA
          CAAGGCAGGC

 1 1 1 1 1
5 110

ASIAN2_Ema   TGCTTTGCAT ACAGAGTATA TTTCCCAATA GTTGAAGAGA AAAGGCAGCA
          GTTAGCTAAG CTCCTGCCTC TCCCCTTTAT GGTCTCACAC AGAAGTTACC
          TGTCTTGGGG
FOR_DS1535   TGCTTTGCAT ACAGAGTATA TTTCCCAATA GTTGAAGAGA AAAGGCAGCA
          GTTAGCTAAG CTCCTGCCTC TCCCCTTTAT GGTCTCACAC AGAAGTTACC
          TGTCTTRGGG
MAM1_SP134   TGCTTTGCAT ACAGAGTATA TTTCCCAATA GTTGAAGAGA AAAGGCAGCA
          GTTAGCTAAG CTCCTGCCTC TCCCCTTTAT GATCTCACAC AGAAGTTACC
          TGTMTTGGGG
MAST_B176_   TGTTTTTCAT ACAGAGTATA TTTCCCAATA GTTGAAGAGA AAAGGCAGCA
          GTTAGCTAAG CCCCTGCCTC TCCCCTTTAT GGTCTCACAC AGAAGTTACC
          TGTCTTGGGA
SAV1_SE210   TGCTTTGCAT ACAGAGTATA TTTCCCAATA GTTGAAGAGA AAAGGCAGCA
          GTTAGCTAAG CTCCTGCCTC TCCCCTTTAT GGTCTCACAC AGAAGTTACC
          TGTCTTGGGG

 1 1 1 1 1
5 115

ASIAN2_Ema   TT-------- --CAT-GTTT GCAGCATTTC TTCTCATCTT TC-ATCCCCT
          ACCTGCTGGA AAACAGTTTG ATTCCATCTT TACTATCTTC CTGAGTCCAT
          CTCTAAAAGA ATGGG
FOR_DS1535   TT-------- --CAT-GTTT GCAGCATTTC TTCTCATCTT TC-ATCCCCT
          ACCTGCTGGA AAACAGTTTG ATTCCATCTT TACTATCTTC CTGAGTCCAT
          CTCTAAAAGA ATGGG
MAM1_SP134   TT-------- --CAT-GTTT GCAGCATTTC TTCTCATCTT TC-ATCCCCT
          ACCTGCTGGA AAACAGTTTG ATTCCATCTT TACTATCTTC CTGAGTCCAT
          CTCTAAAAGA ATGGG
MAST_B130_   TTAGAAAAAG AGTATACTTT GCAGCA-TTC TTCTCATCTT TCTATCCCCT
          ACCTGCTGGA AAACAGTTTG ATT-CA-CCT TACTAT-TTC CTGAGT-CAT
          CTCTAAAAGA ATGGT
SAV1_SE210   TT-------- --CAT-GTTT GCAGCATTTC TTCTCATCTT TC-ATCCCCT
          ACCTGCTGGA AAACAGTTTG ATTCCATCTT TACTATCTTC CTGAGTCCAT
          CTCTAAAAGA ATGGG
```

```
 1 1 1 1 1
5 110

ASIAN2_Ema   TGTGCACACT GGGGACAAAG CCAGTGATCT TCCCCCAGAG CACTTTTT-G
             TT-CACTTGT CACGCACTTT GGTTTACGTA CATCGGGAGC GAGGACAGAA
             AAGACCAAGT
FOR_DS1535   TGTGCACACT GGGGACAAAG CCAGTGATCT TCCCCCAGAG CACTTTTT-G
             TT-CACTTGG CACGCACTTT GGTTTACGTA CATCGGGAGC GAGGACAGAA
             AAGACCAAGT
MAM1_SP134   TGTGCACACT GGGGACAAAG CCAGTGATCT TCCCCCAGAG CACTTTTT-G
             TT-CACTTGT CACGCACTTT GGTTTACGTA CATCGGGAGC GAGGACAGAA
             AAGACCAAGT
MAST_B178_   GTGCACACTG GGGACAAAAG CCAGTGATCT TCCCCCAGAG CACTTTTTTG
             TTTCACTTAT CACGCACTTT GGTTTACGTA CATCGGGAGC GAGGACAGAA
             AAGACCAAGT
SAV1_SE210   TGTGCACACT GGGGACAAAG CCAGTGATCT TCCCCCAGAG CACTTTTT-G
             TT-CACTTGG CACGCACTTT GGTTTACGTA CATCGGGAGC GAGGACAGAA
             AAGACCAAGT

 1 1 1 1 1
5 110

ASIAN2_Ema   TGTAGGCTCT ATGAAGGGAG CGAGGAGGCT GGGCACGGTG AGCTGTTGGG
             GGAGCTCGGT GTTGGGGTGC CAATTAGGCC ATCGGCACCC ACTTCCGATG
             GACTCACCTG
FOR_DS1535   TGTAGGCTCT ATGAAGGGAG CGAGGAGGCT GGGCACGGTG AGCTGTTGGG
             GGAGCTYGGT GTTGGGGTGC CAATTAGGCC ATCGGCACCC ACTTCCGATG
             GACTCACCTG
MAM1_SP134   TGTAGGCTCT ATGAAGGGAG CGAGGAGGCT GGGCACGGTG AGCTGTTGGG
             GGAGCTCGGT GTTGGGGTGC CAATTAGGCC ATCGGCACCC ACTTCCGATG
             GACTCACCTG
MAST_B179_   TGTAGGCTCT ATGAAGGGAG GGAGGAGGCT GGGCACGGTG AGCTGTTGGG
             GGAGCTCGGT GTTGGGGTGC CAATTAGGCC ATCGGCACCC ACTTCTGATG
             GACTCACCTG
SAV1_SE210   TGTAGGCTCT ATGAAGGGAG CGAGGAGGCT GGGCACGGTG AGCTGTTGGG
             GGAGCTCGGT GTTGGGGTGC CAATTAGGCC ATCGGCACCC ACTTCCGATG
             GACTCACCTG

 1 1 1 1 1
5 110

ASIAN2_Ema   AAAGTGAAA- TGAATGTGTG AGGCTATTCC TACACTCATT CACCAAA-TG
             TTTGCTGAGC ACCCCTTACG CAGTAGACCT GTGTGGGGTG CTGGAGACAC
             AACAGAACGG
FOR_DS1535   AAAGTGAAA- TGAATGTGTG AGGCTATTCC TACACTCATT CACCAAA-TG
             TTTGCTGAGC GCCCCTTACG CAGTAGACCT GTGTGGGGTG CTGGAGACAC
             AACAGAACGG
MAM1_SP134   AAAGTGAAA- TGAATGTGTG AGGCTATTCC TACACTCATT CACCAAA-TG
             TTTGCTGAGC GCCCCTTACG CAGTAGACCT GTGTGGGGTG CTGGAGACAC
             AACAGAACGG
MAST_B180_   AAAGTGAAAA TGAATGTGTG AGGCTATTCC TACACTCATT CACCAAAATG
             TTTGCTGAGC GCCCTTTACG CAGTAGACCT GTGTGGGGTG CTGGAGACAC
             AACAGAACGA
SAV1_SE210   AAAGTGAAA- TGAATGTGTG AGGCTATTCC TACACTCCTT CACCAAA-TG
             TTTGCTGAGC GCCCCTTACG CAGTAGACCT GTGTGGGGTG CTGGAGACAC
             AACAGAACGG

 1 1 1 1 1
5 109

ASIAN2_Ema   GGAAAATGCA AATGYTTCCC TGAAACAGAG GTGCTTAGGG GGAAGGAGAG
             GAGCTGGGCT CATCCAAGAA CCCAAATACA GAGGCAAGGT GGACAGAAAA
             GTCCAGAAA
FOR_DS1535   GGAAAATGCA AATGTTTCCC TGAAACAGAG GTGCTTAGGG GGAAGGAGAG
             GAGCTGGGCT CATCCAAGAA CCCAAATACA GAGGCAAGGT GGACAGAAAA
             GTCCAGAAA
MAM1_SP134   GGAAAATGCA AATGTTTCCC TGAAACAGAG GTGCTTAGGG GGAAGGAGAG
             GAGCTGGGCT CATCCAAGAA CCCAAATACA GAGGCAAGGT GGACAGAAAA
             GTCCAGAAA
MAST_B135_   GGAAAATGCA AATGTTTCCT TGAAATAGAG GTGCTTAGGG GGAAGGAGAG
             AAGCTGGGCT CATCCAAGAA CCCAAATACA GAGGCAAGGT GGACAGAAAA
```

```
             ATCCAAAAA
SAV1_SE210   GGAAAATGCA AATGTTTCCC TGAAACAGAG GTGCTTAGGG GGAAGGAGAG
             GAGCTGGGCT CATCCAAGAA CCCAAATACA GAGGCAAGGT GGACAGAAAA
             GTCCAGAAA

 1 1 1 1 1
5 109

ASIAN2_Ema   GAGAAACCAA AA-CC--AAA CCC-GTTGCC CTCAAGTCAA TTCTGACTCA
             TATCAACCCT ATCGGACAGA TTMGAACTGT CCGTAGGGTT TCCAAGGAGC
             ACCTGGTAG
FOR_DS1535   GAGAAACCAA AA-CC--AAA CCC-GTTGCC CTCAAGTCAA TTCTGACTCA
             TATCAACCCT ATCGGACAGA TTAGAACTGT CCRTAGGGTT TCCAAGGAGC
             ACCTGGTAG
MAM1_SP134   GAGAAACCAA AA-CC--AAA CCC-GTTGCC CTCAAGTCAA TTCTGACTCA
             TATCAACCCT ATCGGACAGA TTAGAACTGT CCGTAGGGTT TCCAAGGAGC
             ACCTGGTAG
MAST_B181_   AGAAAACCAA AAACCCGAAA CCCCTTTGCC CTCAAGTCGA TTCTGACTCA
             TATCGACCTT ATCGGACAGA TTAGAACTGT CCGTAGGGTT TCCAAGGAGC
             ACCTGATAA
SAV1_SE210   GAGAAACCAA AA-CC--AAA CCC-GTTGCC CTCAAGTCAA TTCTGACTCA
             TATCAACCCT ATCGGACAGA TTAGAACTGT CCATAGGGTT TCCAAGGAGC
             ACCTGGTAG

 1 1 1 1 1
5 111

ASIAN2_Ema   CAAAAAAGGA CCTGTGGGTT TCCAGGTGGA GGTATGGCAG AAAGGCTTTC
             TGGGCATGTA GAAAAGTATG AACACACAAG AGGGTGGTGG TTTTGGGGAA
             TGGCTGCAGT C
FOR_DS1535   CAAAAAAGGA CCTGTGGGTT TCCAGGTGGA GGTATGGCAG AAAGGCTTTC
             TGGGCATGTA GAAAAGTATG AACACACAAG AGGGTGGTGG TTTTGGGGAA
             TGGCTGCAGT C
MAM1_SP134   CAAAAAAGGA CCTGTGGGTT TCCAGGTGGA GGTATGGCAG AAAGGCTTTC
             TGGGCATGTA GAAAAGTATG AACACACAAG AGGGTGGTGG TTTTGGGGAA
             TGGCTGCAGT C
MAST_B184_   CAAAAAATGA TTTGTGGGTT TTTAGGTGGA GGTATGGCAG AAAGGCTTTC
             TGGGCATGTA GAAAAGTATG AACACACAAG AGGGTGGTGG TTTTGGG-AA
             TGGTT-CAGT C
SAV1_SE210   CAAAAAAGGA CCTGTGGGTT TCCAGGTGGA GGTATGGCAG AAAGGCTTTC
             TGGGCATGTA GAAAAGTATG AACACRCAAG AGGGTGGTGG TTTTGGGGAA
             TGGCTGCAGT C

 1 1 1 1 1
5 113

ASIAN2_Ema   -TTTTTTTTT TTTAGCATAG TTACCAGGAA CATCGGTGGT GACT----CA
             GCTCTAGACC TAAAGAATCA ATTCCCCCAT TCCCACTCCT GCCCACTGTC
             TTCCCAGATC GTA
FOR_DS1535   --TTTTTTTT TTTAGCATAG TTACCAGGAA CATCGGTGGT GACT----CA
             GCTCTAGACC TAAAGAATCA ATTCCCCCAT TCCCACTCCT RCCCACTGTC
             TTCCCAGATC GTA
MAM1_SP134   TTTTTTTTTT TTTAGCATAG TTACCAGGAA CATCGGTGGT GACTGACTCA
             GCTCTAGACC TAAAGAATCA ATTCCCCCAT TCCCACTCCT GCCCACTGTC
             TTCCCAGATC ATA
MAST_B137_   TTTTTTTTTT TTTAGCATAG TTACCAGGAA CATCGGTGGT GACT----CA
             GCTCTAGACC TAAAGAATCA ATTCCCCCAT TCCCACTCCT GCCCACTGTC
             TTCCCAAATC GTA
SAV1_SE210   -TTTTTTTTT TTTAGCATAG TTACCAGGAA CATCGGTGGT GACT----CA
             GCTCTAGACC TAAAGAATCA ATTCCCCCAT TCCCACTCCT GCCCACTGTC
             TTCCCAGATC GTA

 1 1 1 1 1
5 110

ASIAN2_Ema   GGGAGATACC TCACCACTTG GGGGCTGGTG AAGAGAGGGT CTGAGAAGAG
             GCTTGGGGCT TTAGCACAGA TGTGGGGCCC TCTAGGTCGG GGACCACTTA
             GCTTTCCAGA
FOR_DS1535   GGGAGATACC TCACCACTTG GGGGCTGGTG AAGAGAGGGT CTGAGAAGAG
             GCTTGGGGCT TTAGCACAGA TGTGGGGCCC TCTAGGTCGG GGACCACTTA
             GCTTTCCAGA
```

```
MAM1_SP134    GGGAGATACC TCACCACTTG GGGGCTGGTG AAGAGAGGGT CTGAGAAGAG
              GCTTGGGGCT TTAGCACAGA TGTGGGGCCC TCTAGGTCGG GGACCACTTA
              GCTTTCCAGA
MAST_B139_    GGGAGATACC TCACCACTTG GGG-CTGGTG AAGAGAGGGT CTGAGAAGAG
              GCTTGGGGCT TTAGCACAGA TGTGGGGCCC TCTAGGTCGG GGACCACTTA
              GCTTTCCAGA
SAV1_SE210    GGGAGATACC TCACCACTTG GGGGCTGGTG AAGAGAGGGT CTGAGAAGAG
              GCTTGGGGCT TTAGCACAGA TGTGGGGCCC TCTAGGTCGG GGACCACTTA
              GCTTTCCAGA

 1 1 1 1 1
5 108

ASIAN2_Ema    GTTTCAACCT TTAGTTAGAA AAGATGTGCT AGCTTAGAAG AAAACATTCC
              AGCTCYGTGG GATGGAGACC TGGAGTCTTG CCTGATGTCA AACCCTTTGA
              GGCCTAAT
FOR_DS1535    GTTTCAACCT TTAGTTAGAA AAGATGTGCT AGCTTAGAAG AAAACATTCC
              AGCTCCGTGG GATGGAGACC TGGAGTCTTG CCTGATGTCA AACCCTTTGA
              GGCCTAAT
MAM1_SP134    GTTTCAACCT TTAGTTAGAA AAGATGTGCT AGCTTAGAAG AAAACATTCC
              AGCTCCGTGG GATGGAGACC TGGAGTCTTG CCTGATGTCA AACCCTTTGA
              GGCCTAAT
MAST_B140_    GTTTCAACCT TTAGTCAGAA AAGATGTGCT AGCTTAGAAG AAAACATTCC
              AGCTCCGTGG GATGGAGACC TGGAGTCTTG CCTGATGTCA AACCCTTTGA
              GGCCTAAT
SAV1_SE210    GTTTCAACCT TTAGTTAGAA AAGATGTGCT AGCTTAGAAG AAAACATTCC
              AGCTCCGTGG GATGGAGACC TGGAGTCTTG CCTGATGTCA AACCCTTTGA
              GGCCTAAT

 1 1 1 1 1
5 108

ASIAN2_Ema    ACATGAAAGA AGATTTACAA CCCAGATTTA AATGCAGGA- TTCTGTA-TA
              TATGCAATTA ATGA-TC-TT CCCTATATGA TACTTCA-TT TTGGCTTCTT
              TACCTTAA
FOR_DS1535    ACATGAAAGA AGATTTACAA CCCAGATTTA AATGCAGGA- TTCTGTA-TA
              TATGCAATTA ATGA-TC-TT CCCTATATGA TACTTCA-TT TTGGCTTCTT
              TACCTTAA
MAM1_SP134    ACATGAAAGA AGATTTACAA CCCAGATTTA AATGCAGGA- TTCTGTA-TA
              TATGCAATTA ATGA-TC-TT CCCTATATGA TACTTCA-TT TTGGCTTCTT
              TACCTTAA
MAST_B188_    ACAAGAAAGA AGATTTACAA CCCAGATTTA AATGCAGGAG TTCTGTACTA
              TATGCAATTA ATGAATCGTT CCCTATATGA TACTTCAGTT TTGGCTTCTT
              TACCTTAA
SAV1_SE210    ACATGAAAGA AGATTTACAA CCCAGATTTA AATGCAGGA- TTCTGTA-TA
              TATGCAATTA ATGA-TC-TT CCCTATATGA TACTTCA-TT TTGGCTTCTT
              TACCTTAA

 1 1 1 1 1
5 108

ASIAN2_Ema    TGCCCTGTTT GGGCCCTGGT CATCCATTAT GGGTCTCCCT GTGTTGGAGG
              CCAGTTTATC CTTTCCTGGA GTCTCACCTT GCCTCTAGCC CTGGAAC-TG
              GTCCTGTC
FOR_DS1535    TGCCCTGTTT GGGCCCTGGT CATCCATTAT GGGTCTCCCT GTGTTGGAGG
              CCASTTTATC CTTTCCTGGA GTCTCACCTT GCCTCTAGCC CTGGAAC-TG
              GTCCTGTC
MAM1_SP134    TGCCCTGTTT GGGCCCTGGT CATCCATTAT GGGTCTCCCT GTGTTGGAGG
              CCAGTTTATC CTTTCCTGGA GTCTCACCTT GCCTCTAGCC CTGGAAC-TG
              GTCCTGTC
MAST_B141_    TGCCCTGTTT GGGCCCTGGT CAACCACTAT GGGTCTCCCT GTGTTGGAGG
              CCACTTTATC CTTTTCTGGA GTCTCACCTT GCCTCTAGCC CTGGAACGTG
              GTCCTATC
SAV1_SE210    TGCCCTGTTT GGGCCCTGGT CATCCATTAT GGGTCTCCCT GTGTTGGAGG
              CCASTTTATC CTTTCCTGGA GTCTCACCTT GCCTCTAGCC CTGGAAC-TG
              GTCCTGTC

 1 1 1 1 1
5 108

ASIAN2_Ema    TGCAAAATTC TATCATTTGA TCTCTGGCAT TGTTTCTATC ACCAAGGCCA
```

```
              TATTTTCCAA CTACCCATCC TTCTTCTTTG TTTCCAACTT TTGCATTCCA
              ATCACCAG
FOR_DS1535    TGCAAAATTC TATCATTTGA TCTCTGGCAT TGTTTCTATC ACCAAGGCCA
              TATTTTCCAA CTACCCATCC TTCTTCTTTG TTTCCAACTT TTGCATTCCA
              ATCACCAG
MAM1_SP134    TGCAAAATTC TATCATTTGA TCTCTGGCAT TGTTTCTATC ACCAAGGCCA
              TATTTTCCAA CTACCCATCC TTCTTCTTTG TTTCCAACTT TTGCATTCCA
              ATCACCAG
MAST_B143_    TGCAAAATTC TATCCTGTGA TCTCTGGCAT TGTTTCTATC ACCAAGGCCA
              TATTTTCCAG CTACCAATCC TTCTTCTTTG TTTCCAACTT TTGCATTCCA
              ATCACCAA
SAV1_SE210    TGCAAAATTC TATCATTTGA TCTCTGGCAT TGTTTCTATC ACCAAGGCCA
              TATTTTCCAA CTACCCATCC TTCTTCTTTG TTTCCAACTT TTGCATTCCA
              ATCACCAG

 1 1 1 1 1
5 112

ASIAN2_Ema    CAAGATAAAG AATTACCCGT TAAACAGTA- TTTGAGGAAT GTT-GGTCAT
              TGTGACACTG GGGTCGTTTT TAAAATCACT CACAAGGCGT CAGTCTTCAT
              AATAATTGGC TC
FOR_DS1535    CAAGATAAAG AATTACCCGT TAAACAGTA- TTTGAGGAAT GTT-GGTCAT
              TGTGACACTG GGGTCGTTTT TAAAATCACT CACAAGGCGT CAGTCTTCAT
              AATAATTGGC TC
MAM1_SP134    CAAAATAAAG AATTACCCAT TAAACAGTA- TTTGAGGAAT GTT-GGTCAT
              TGTGACACTG GGGTAGTTTT TAAAATCACT CACAAGGCGT CAGTCTTCAT
              AATAATTGGC TC
MAST_B189_    TAAGATAAAG AATTACCCGT TAAACAGTAC TTTGAGGAAT GTTTGGTCAT
              TGTGACACTG GGGTCGTTTT TAAAATCAC- ---AAGGCGT CAGTCTTCAT
              AATAATTGGC TC
SAV1_SE210    CAAGATAAAG AATTACCCGT TAAACAGTA- TTTGAGGAAT GTT-GGTCAT
              TGTGACACTG GGGTCGTTTT TAAAATCACT CACAAGGCGT CAGTCTTCAT
              AATAATTGGC TC

 1 1 1 1 1
5 108

ASIAN2_Ema    CAGCAGTAGG TTCAGCCTTA GTGSTCTTTA ACTTGTGGGT CCYGAGCACA
              GCTGGGCCTT TTGGGGTACT CCCAGATACG GTCACATGTG GAGACTTTCT
              GGCTGGAG
FOR_DS1535    CAGCAGTAGG TTCAGCCTTA GTGGTCTTTA ACTTGTGGGT CCCGAGCACA
              GCTGGGCCTT TTGGGGTACT CCCAGAAACG GTCACATGTG GAGACTTTCT
              GGCTGGAG
MAM1_SP134    CAGCAGTAGG TTCAGCCTTA GTGGTCTTTA ACTTGTGGGT CCCGAGCACA
              GCTGGGCCTT TTGGGGTACT CCCAGATACG GTCACATGTG GAGACTTTCT
              GGCTGGAG
MAST_B191_    TAGCAGCAGG TTCAGCCTTA GTGGTCTTTA ACTTGTGGGT CCCGAGCACA
              GCTGGGCCTT TTGGGGTACT CCCAGATACG GTCACATGTG GAGACTTTCT
              GGCTGGAA
SAV1_SE210    CAGCAGTAGG TTCAGCCTTA GTGGTCTTTA ACTTGTGGGT CCCGAGCACA
              GCTGGGCCTT TTGGGGTACT CCCAGAAACG GTCACATGTG GAGACTTTCT
              GGCTGGAG

 1 1 1 1 1
5 107

ASIAN2_Ema    ATAATCATTG TTGAGTTGTT GGAGSCCACT GTGAAGACTA TTTTCGACAT
              GACRACTTAG AGCCATTTAT CCCTTCATTC TCTAGCTTCT CCCTCCAGC-
              AGAGAAC
FOR_DS1535    ATAATCATTG TTGAGTTGTT GGAGCCCACT GTGAAGACTA TTTTCGACAT
              GACGACTTAG AGCCATTTAT CCCTTCATTC TCTAGCTTCT CCCTCCAGC-
              AGAGAAC
MAM1_SP134    ATAATCATTG TTGAGTTGTT GGAGCCCACT GTGAAGACTA TTTTCGACAT
              GATGACTTAG AGCCATTTAT CCCTTCATTC TCTAGCTTCT CCCTCCAGC-
              AGAGAAC
MAST_B192_    ATAATCATTG TTGAGTTGTT GGAGCCCACT GTGAAGACTA TTTTCGACAT
              GACGACTTAG AGCCATTTAT CCCTTCATTC TCTAGCTTCT CCCTCCAGCG
              AGAGAAC
SAV1_SE210    ATAATCATTG TTGAGTTGTT GGAGCCCACT GTGAAGACTA TTTTCGACAT
              GACGACTTAG AGCCATTTAT CCCTTCATTC TCTAGCTTCT CCCTCCAGC-
              AGAGAAC
```

1 1 1 1 1
5 107

```
ASIAN2_Ema    GTACAGGAAC AGATGTGTGG GACAACAAGA TCATTTGT-A CCTAGTTCAG
              AGGGTATGTT CTACAATCTT ATTAAAAAGC AAACCAAAAC AAAACTCAAT
              TAAAAAA
FOR_DS1535    GTACAGGAAC AGATGTGTGG GACAACAAGA TCATTTGT-A CCTAGTTCAG
              AGGGTATGTT CTACAATCTT ATTAAAAAGC AAACCAAAAC AAAACTCAAT
              TAAAAAA
MAM1_SP134    GTACAGGAAC AGATGTGTGG GACAACAAGA TCATTTGT-A CCTAGTTCAG
              AGGGTATGTT CTACAATCTT ATTAAAAAGC AAACCAAAAC AAAACTCAAT
              TAAAAAA
MAST_B193_    GTGTAGGAAC AGATATGTGG GACAACAAGA TCATTTGTTA CTTAGTTGAG
              AGGGTATGTT CCACAATCTT ATTAAAAAGC AAACCAAAAC AAAACTCAAT
              TAAAAAA
SAV1_SE210    GTACAGGAAC AGATGTGTGG GACAACAAGA TCATTTGT-A CCTAGTTCAG
              AGGGTATGTT CTACAATCTT ATTAAAAAGC AAACCAAAAC AAAACTCAAT
              TAAAAAA
```

1 1 1 1 1
5 107

```
ASIAN2_Ema    GATAACGAGC TGAACGCAGG ATCCAGAGCA GAGCAAAAGC CAGCGAGCTT
              TGCCAGAAAG TCCACATATA TTGGATGCAG GCCACACCCC CAAGGAAACT
              CCCTTTC
FOR_DS1535    GATAACGAGC TGAATGCAGG ATCCAGAGCA GAGCAAAAGC CAGCGAGCTT
              TGCCAGAAAG TCCACATATA YTGGATGCAG GCCACACCCC CAAGGAAACT
              CCCTTTC
MAM1_SP134    GATAACGAGC TGAATGCAGG ATCCAGAGCA GAGCAAAAGC CAGCGAGCTT
              TGCCAGAAAG TCCACATATA TTGGATGCAG GCCACACCCC CAAGGAAACT
              CCCTTTC
MAST_B194_    GTCAATGAGC TGAATGCAAG ATCCAGAGCA AAGCAGAAGC CTGCGAGCTT
              TGCCAGAAAG TCCACATATA TTGGATGCAG GCCACACACC CGAGGAAACT
              CCGTTTC
SAV1_SE210    GATAACGAGC TGAATGCAGG ATCCAGAGCA GAGCAAAAGC CAGCGAGCTT
              TGCCAGAAAG TCCACATATA TTGGATGCAG GCCACACCCC CAAGGAAACT
              CCCTTTC
```

1 1 1 1 1
5 107

```
ASIAN2_Ema    TCTTTACAAG ATCAGATATT TTACAATCAG GAACATTCCT AGAGAACCAA
              TACCAAAATC AAGGCAGACA AGAGATAAAG CCTCAGCTGC ACCAGCCAAG
              AGCAGCA
FOR_DS1535    TCTTTACAAG ATCAGATATT TTACAATCAG GAACATTCCT AGAGAACCAA
              TACCAAAATC AAGGCAGACA AGMRATAAAG CCTCAGCTGC ACCAGCCAAG
              AGMAGCA
MAM1_SP134    TCTTTACAAG ATCAGATATT TTACAATCAG GAACATTCCT AGAGAACCAA
              TACCAAAATC AAGGCAGACA AGAGATAAAG CCTCAGCTGC ACCAGCCAAG
              AGCAGCA
MAST_B217_    TCTTTACAAG ATCAGATATT TTACAATCAG GAACATTCTT AGAGAACCAA
              TACCAAAATC AAGGCAGACA AGAGATAAAG CCTCAGCTGC ACCAGCCAAG
              AGCAGCA
SAV1_SE210    TCTTTACAAG ATCAGATATT TTACAATCAG GAACATTCCT AGAGAACCAA
              TACCAAAATC AAGGCAGACA AGAGATAAAG CCTCAGCTGC ACCAGCCAAG
              AGCAGCA
```

1 1 1 1 1
5 108

```
ASIAN2_Ema    TGAGCATTGG GACTGGCTCC TGTGTAGGCG TCTGGGGAGA CTGCGGCCCT
              CTGGCCCTCG GCTGGTTCCT CATCCTCCCC TCATCTCCAG ATCTGGACAC
              TCCCAAAC
FOR_DS1535    TGAGCATTGG GACTGGCTCC TGTGTAGGCG TCTGGGGAGA CTGCGGCCCT
              CTGGCCCTCG GCTGGTTCCT CATCCTCCCC TCATCTCCAG ATCTGGACAC
              TCCCAAAC
MAM1_SP134    TGAGCATTGG GACTGGCTCC TGTGTAGGCG TCTGGGGAGA CTGCGGCCCT
              CTGGCCCTCG GCTGGTTCCT CATCCTCCCC TCATCTCCAG ATCTGGACAC
              TCCCAAAC
MAST_B219_    TGAGCATTGG GACTGGCTCC TGTGTAGGCG TCTGGGGAGA CTGCGGCC-T
```

CTGGCCCTCG GCTGGTTCCT CATCCTCCCC TCATCTCCAG ATCTGGACAC
TCCCAAAC
SAV1_SE210 TGAGCATTGG GACTGGCTCC TGTGTAGGCG TCTGGGGAGA CTGCGGCCCT
CTGGCCCTCG GCTGGTTCCT CATCCTCCCC TCATCTCCAG ATCTGGACAC
TCCCAAAC

1 1 1 1 1
5 107

ASIAN2_Ema TAGATGACTG GGTGGA-TCC ACCAAGTTTA TGAAAGAAGA TAGGAATAT-
CTGGATTTGG TTTCAGGTGT TGGAGATCAT GGTTGAAATT TAGGGACAGA
GTGAAAG
FOR_DS1535 TAGATGACTG GGTGGA-TCC ACCAAGTTTA TGAAAGAAGA TAGGAATAT-
CTGGATTTGG TTTCAGGTGT TGGAGATCAT GGTTGAAWTT TAGGGACAGA
GTGAAAG
MAM1_SP134 TAGATGACTG GGTGGA-TCC ACCAAGTTTA TGAAAGAAGA TAGGAATAT-
CTGGATTTGG TTTCAGGTGT TGGAGATCAT GGTTGAAATT TAGGGACAGA
GTGAAAG
MAST_B220_ TAGGGAAAAA AATAAAGAAT ACCAAGTTTA TGAGAGAAGA TGGGAATATT
CTGGATTTGG TTTCAGGTGT TGGAGATCAT GGTTGAAATT TAGGGACAGA
GTAAAAG
SAV1_SE210 TAGATGACTG GGTGGA-TCC ACCAAGTTTA TGAAAGAAGA TAGGAATAT-
CTGGATTTGG TTTCAGGTGT TGGAGATCCT GGTTGAAATT TAGGGACAGA
GTGAAAG

1 1 1 1 1
5 107

ASIAN2_Ema CCTCAGGGCC CCACCACGAT GCACGTGCAG CTGATCATGG AGCGGCTGCT
GAGAAGGATC AACCGCACTG TGATTGGGAT GAGCCGACAG TCTCCCCACA
TCGTGAG
FOR_DS1535 CCTCAGGGCC CCACCACGAT GCACGTGCAG CTGATCATGG AGCGGCTGCT
GAGAAGGATC AACCGCACTG TGATTGGGAT GAGCCGACAG TCTCCCCACA
TCGTGAG
MAM1_SP134 CCTCAGGGCC CCACCACGAT GCACGTGCAG CTGATCATGG AGCGGCTGCT
GAGAAGGATC AACCGCACTG TGATTGGGAT GAGCCGACAG TCTCCCCACA
TCGTGAG
MAST_B221_ TCTCAGGGCC CCACCACGAT GCACGTGCAG CTGATCATGG AGCGGTTGTT
GAGAAGGATC AACCGCACTG TGATTGAGAT GAGCCGACAG TCTCCCCACA
TCGTAAA
SAV1_SE210 CCTCAGGGCC CCACCACGAT GCACGTGCAG CTGATCATGG AGCGGCTGCT
GAGAAGGATC AACCGCACTG TGATTGGGAT GAGCCGACAG TCTCCCCACA
TCGTGAG

1 1 1 1 1
5 107

ASIAN2_Ema TTGGACACAT CTGCCAAACG GACCCGTGTG AGTGAGGCAA GGCCAGGTCT
CAATGTGTTA GTAAGGAGGG TCCCCCGAAG GGATGAAGAG CTGAGCGAGC
TTCAGGC
FOR_DS1535 TTGGACACAT CTGCCAAACG GACCCGTGTG AGTGAGGCAA GGCCAGGTCT
CAATGCGTTA GTAAGGAGGG TCCYCCGAAG GGATGAAGAG CTGAGCGAGC
TTCAGGC
MAM1_SP134 TTGGACACAT CTGCCAAACG GACCCGTGTG AGTGAGGCAA GGCCAGGTCT
CAATGTGTTA GTAAGGAGGG TCCCCCGAAG GGATGAAGAG CTGAGCGAGC
TTCAGGC
MAST_B222_ TTGGACATAT TTGCCAAACG GACCCGTGTG AGTGAGGCAA GGCCAGGTCT
CAATGCGTTA GCAAGGAGGG TCCCCCAAAG GGATGAAGAG CTGAGCAAGC
TTCAGGC
SAV1_SE210 TTGGACACAT CTGCCAAACG GACCCGTGTG AGTGAGGCAA GGCCAGGTCT
CAATGCGTTA GTAAGGAGGG TCCCCCGAAG GGATGAAGAG CTGAGCGAGC
TTCAGGC

1 1 1 1 1
5 107

ASIAN2_Ema CCCTCCTGGC TCTGAGGAAC ACATGACCCT CCTCTAGTTA ATAACCAATC
CCTCCACTTG GGCTGTACAT CCARTGAACT CCAGTTCATT CAGGAGCCTT
ATTATAC
FOR_DS1535 CCCTCCTGGC TCTGAGGAAC ACATGACCCT CCTCTAGTTA ATAACCAATC
CCTCCACTTG GGCTGTACAT CCAGTGAACT CCAGTTCATT CAGGAGCCTT

ATTATAC

```
MAM1_SP134   CCCTCCYGGC TCTGAGGAAC ACATGACCCT CCTCTAGTTA ATAACCAATC
             CCTCCACTTG GGCTGTACAT CCAGTGAACT CCAGTTCATT CAGGAGCCTT
             ATTATAC
MAST_B195_   CCCTCCTGGC TCTGAGGAAC ACATGACCCT CCTCTAGTTA ATAACCAATC
             CCTCCACTTG GGCTGTACAT CCAGTGAACT CCAGTTCATT CAGGAGCCTT
             ATTATAC
SAV1_SE210   CCCTCCTGGC TCTGAGGAAC ACATGACCCT CCTCTAGTTA ATAACCAATC
             CCTCCACTTG GGCTGTACAT CCAGTGAACT CCAGTTCATT CAGGAGCCTT
             ATTATAC
```

1 1 1 1 1
5 110

```
ASIAN2_Ema   CCCACCTTGA GGTCACTTGG GGCTGGGGTT GCATAAGGAA CTCAAAGTGA
             GGGCAAGGTC CAGACAGAGA GAGCAAAGGC AGGTACTGAG GGAAGGAGAA
             GTGAAACTGG
FOR_DS1535   CCCACCTTGA GGTCACTTGA GGCTGGGGTT GCATAAGGAA CTCAAAGTGA
             GGGCAAGGTC CAGACAGAGA GAGCAAAGGC AGGTACTGAG GGAAGGAGAA
             GTGAAACTGG
MAM1_SP134   CCCACCTTGA GGTCACTTGG GGCTGGGGTT GCATAAGGAA CTCAAAGTGA
             GGGCAAGGTC CAGACAGAGA GAGCAAAGGC AGGTACTGAG GGAAGGAGAA
             GTGAAACTGG
MAST_B196_   TTTATCTTGA GGTCACTTGG G-CTGGG-TT GCATAAGGAA CTCAAAGTGA
             GGGCAAGGTC CAGACAGAGA GAGCAAAGGC AGGTACTGAG G-AAGGAGAA
             GTGAAACTGG
SAV1_SE210   CCCACCTTGA GGTCACTTGG GGCTGGGGTT GCATAAGGAA CTCAAAGTGA
             GGGCAAGATC CAGACAGAGA GAGCAAAGGC AGGTACTGAG GGAAGGAGAA
             GTGAAACTGG
```

1 1 1 1 1
5 108

```
ASIAN2_Ema   TAAAATTATA CGTTATTTTC TTCTTTGCTG GTGCGAGGGG AGGAAGGTGA
             TGGGAAGTGG AGAGAAGAAC AGCTATCAGC TCAGGAAGAT TGAGGCATGA
             GCTAGTGA
FOR_DS1535   TAAAATTATA CGTTATTTTC TTCTTTGCTG GTGCGAGGGG AGGAAGGTGA
             TGGGAAGTGG AGAGAAGAAC AGCTATCAGC TCAGGAAGAT TGAGGCATGA
             GCTAGTGA
MAM1_SP134   TAAAATTATA CGTTATTTTC TTCTTTGCTG GTGCGAGGGG AGGAAGGTGA
             TGGGAAGTGG AGAGAAGAAC AGCTATCAGC TCAGGAAGAT TGAGGCATGA
             GCTAGTGA
MAST_B223_   TAAAATTATA CGTTATTTTC TTCTTTGCTG GTGCGAGGGG AGGAAGGTGA
             -GGGAAGTGG AGAGAAGAAC AGCTATGAGC TCAGGAAGAT TGAGGCATGA
             GCTAGTGA
SAV1_SE210   TAAAATTATA CGTTATTTTC TTCTTTGCTG GTGCGAGGGG AGGAAGGTGA
             TGGGAAGTGG AGAGAAGAAC AGCTATCAGC TCAGGAAGAT TGAGGCATGA
             GCTAGTGA
```

1 1 1 1 1
5 106

```
ASIAN2_Ema   CAAGAAATTT TAAAGACAGT TACCTGGCCA ACTGACTGGA ATAGATTCAT
             GTTTGTGCCC ATTCCAAAGA AAGGTGGTCC AACAGAATGC AGAAATTATT
             GAACAA
FOR_DS1535   CAAGAAATTT TAAAGACAGT TACCTGGCCA ACTGACTGGA ATAGATCCAT
             GTTTGTGCCC ATTCCAAAGA AAGGTGGTCC AACAGAATGC AGAAATTATT
             GAACAA
MAM1_SP134   CAAGAAATTT TAAAGACAGT TACCTGGCCA ACTGACTGGA ATAGATCCAT
             GTTTGTGCCC ATTCCAAAGA AAGGTGGTCC AACAGAATGC AGAAATTATT
             GAACAA
MAST_B226_   TAAGAAATTT TAAAGACAGT TACCTGGCCA ACTGACTGGA ATAGATCCAT
             GTTTGTGCCC ATTCCAAAGA AAGGTGGTCC AACAGAATGC AGAAATTATT
             GAACAA
SAV1_SE210   CAAGAAATTT TAAAGACAGT TACCTGGCCA ACTGACTGGA ATAGATCCAT
             GTTTGTGCCC ATTCCAAAGA AAGGTGGTCC AACAGAATGC AGAAATTATT
             GAACAA
```

1 1 1 1 1
5 106

```
ASIAN2_Ema    TTGCCAGGAA TTTCTGGACC TCCTTGGGAC CAAGGGCTGA TCTGGGTGCT
              AGGGTACATC TGTGCACCCA GAGTTCCTCC CCTTGGGGGC AGTCAAGCAA
              GGGGGG
FOR_DS1535    TTGCCAGGAA TTTCTGGACC TCCTTGGGAC CAAGGGCTGA TCTGGGTGCT
              AGGGTACATC TGTGCACCCA GAGTTCCTCC CCTTGGGGGY AGTCAAGCAA
              GGGGGG
MAM1_SP134    TTGCCAGGAA TTTCTGGACC TCCTTGGGAC CAAGGGCTGA TCTGGGTGCT
              AGGGTACATC TGTGCACCCA GAGTTCCTCC CCTTGGGGGC AGTCAAGCAA
              GGGGGG
MAST_B197_    TTGCCAGGAA TTTCTGGACC TCTTTGGGAC CAAGGGCTGA TCTGGGTGCT
              AAAGTACATC TGTGCACCCA GAGTTCCTCC CCTTGGGGGC AGTCAAGCAA
              GGAGGA
SAV1_SE210    TTGCCAGGAA TTTCTGGACC TCCTTGGGAC CAAGGGCTGA TCTGGGTGCT
              AGGGTACATC TGTGCACCCA GAGTTCCTCC CCTTGGGGGC AGTCAAGCAA
              GGGGGG

 1 1 1 1 1
5 110

ASIAN2_Ema    GAAACATCAG GCCTAAGCAT CCAAAGAAAT GGAGACCC-A GAGAGGTTAG
              CTACCTTACC TAGAGTCACA GACAGATCAT C-AAG-TCCT TT-CTCTTCA
              CTCTTTT-CT
FOR_DS1535    GAAACATCAG GTCTAMGCAT CCAAAGAAAT GGAGACCC-A GAGAGGTTAG
              CTACCTTACC TAGAGTCACA GACAGATCAT C-AAG-TCCT TT-CTCTTCA
              CTCTTTT-CT
MAM1_SP134    GAAACATCAG GCCTAAGCAT CCAAAGAAAT GGAGACCCCA GAGAGGTTAG
              CTACCTTACC TAGAGTCACA GACAGATCAT C-AAG-TCCT TT-CTCTTCA
              CTCTTTT-CT
MAST_B198_    GAA-CATCAG GTCTAAGCAT CCAA-GAA-T GGAGACCC-A GAGAGGTTAG
              CTACCTTACC TAGAGTCACA GACAGATCAT CGAAGGTCCT TTTCTCTTCA
              CTCTTTTTCT
SAV1_SE210    GAARCATCAG GTCTAAGCAT CCAAAGAAAT GGAGACCC-A GAGAGGTTAG
              CTACCTTACC TAGAGTCACA GACAGATCAT C-AAG-TCCT TT-CTCTTCA
              CTCTTTT-CT

 1 1 1 1 1
5 106

ASIAN2_Ema    CAAAGAGGCA AAAG------ --TAGGACAT GGACAAGCAT GCTGCCTGAG
              CCCTATCTGC TCGAATCCAA GGAGTATCAC AGAATAGGCA AAAGTCAGAA
              AGTGGA
FOR_DS1535    CAAAGAGGCA AAAG------ --TAGGACAT GGACAAGCAT GCTGTCTGAG
              CCCTATCTGC TCGAATCCAA GGAGTATCAC AGAATAGGCA AAAGTCAGAA
              AGTGGA
MAM1_SP134    CAAAGAGGCA AAAG------ --TAGGACAT GGACAAGCAT GCTGCCTGAG
              CCCTATCTGC TCGAATCCAA GGAGTATCAC AGAATAGGCA AAAGTCAGAA
              AGTGGA
MAST_B229_    CAAAAAAGAG AAAGTCCCTT GATAGGACAC AGACAAGCAT GCTGCCCGAG
              CCCTATCTGC TCGAATCCAA GGAGTATCAC AGAATAGGCA AAAGTCAGAA
              AGTGGA
SAV1_SE210    CAAAGAGGCA AAAG------ --TAGGACAT GGACAAGCAT GCTGCCTGAG
              CCCTATCTGC TCGAATCCAA GGAGTATCAC AGAATAGGCA AAAGTCAGAA
              AGTGGA

 1 1 1 1 1
5 107

ASIAN2_Ema    AGAACAGACG AGTGTGTGCA GTGTCA---- ---------- ----------
              ---------- ---G-CTGTG CC-AGGGCTC TG-TGGAAAA GCTGCTGGGA
              GAACAGA
FOR_DS1535    AGAACAGACG AGTGTGTGCA GTGTCAGAAG GTGCCCCCG- CCTCACACGC
              GCTCCCTGGG ACAG-CTGTG CC-AGGGCTC TG-TGGAAAA GCTGCTGGGA
              GAACAGA
MAM1_SP134    AGAACAGACG AGTGTGTGCA GTGTCAGAGG GTGTCCCCG- CCTCACACGC
              GCTCCCTGGG ACAG-CTGTG CC-AGGGCTC TG-TGGAAAA GCTGCTGGGA
              GAACAGA
MAST_B230_    AGAACAGACG AGTGTATGCA GCGTCAGA-G GTGCCCCCGG CCTCACACGT
              GCTCCCTGGG ACAGGCTGTG CCGAGGGCTC TGGTGGAAAA GCTGCTGGGA
              GAACAGA
SAV1_SE210    AGAACAGACG AGTGTGTGCA GTGTCAGAAG GTGCCCCCG- CCTCACACGC
              ACTCCCTGGG ACAG-CTGTG CC-AGGGCTC TG-TGGAAAA GCTGCTGGGA
```

```
              GAACAGA

 1 1 1 1 1
5 106

ASIAN2_Ema    TCTAAGTGAA GATGAGACGT CTCAAGGAAT CAAGGCACTG CAGTGGGCAG
              GAGGGGCTTC AGGAAATGAG TGAAGAAGGC ACAGCCTCAG CCTCCTGGTG
              AGCCCA
FOR_DS1535    TCTAAGTGAA GATGAGACGT CTCAAGGAAT CAAGGCACTG CAGTGGGCAG
              GAGGGGCTTC AGGAAATGAG TRAAGAAGGC ACAGCCTCAG CCTCCTGGTG
              AGCCCA
MAM1_SP134    TCTAAGTGAA GATGAGACGT CTCAAGGAAT CAAGGCACTG CAGTGGGCAG
              GAGGGGCTTC AGGAAATGAG TGAAGAAGGC ACAGCCTCAG CCTCCTGGTG
              AGCCCT
MAST_B233_    TCTAAGTGAA GATGAGCCGT CTCAAGGAAT CAAGGCACTG CAGTGGGCAG
              GAGGGGCTTC AGGAAATGAG TGAAGAAGGC ACAACCTCAG CCTCCTGGTG
              AGCCCA
SAV1_SE210    TCTAAGTGAA GATGAGCCGT CTCAAGGAAT CAAGGCACTG CAGTGGGCAG
              GAGGGGCTTC AGGAAATGAG TGAAGAAGGC ACAGCCTCAG CCTCCTGGTG
              AGCCCA

 1 1 1 1 1
5 52

ASIAN2_Ema    TCTCAGGCCT C-AGTCTTCA CATCCGCCC- AC-AGGGGCT TGTCTGTTTT
              GA
FOR_DS1535    TCTCAGGCCT C-AGTCTTCA CATCCGCCC- AC-AGGGGCT TGTCTGTTTT
              GA
MAM1_SP134    TCTCAGGCCT C-AGTCTTCA CATCCGCCC- AC-AGGGGCT TGTCTGTTTT
              GA
MAST_B234_    CTTCAG-CCT CGAGTCTCCA CATCCGCCCT ACGAGGGGCT TGTCTGTTTT
              AA
SAV1_SE210    TCTCAGGCCT C-AGTCTTCA CATCCGCCC- AC-AGGGGCT TGTCTGTTTT
              GA

 1 1 1 1 1
5 106

ASIAN2_Ema    GGGCCTCTCA GTCTCTCCAG AGTCTTTGGC CAGAGATAAC TGGGCTCCAC
              TCAGCCCGGG TGCTCTGTGT CTCCAGGTAA GAGTCTACCA CATGCCCAGC
              CTGCCC
FOR_DS1535    GGGCCTCTCA GTCTCTCCAG AGTCTTTGGC CAGAGATAAC TGGGCTCCAC
              TCAGCCCGGG TGCTCTGTGT CTCCAGGTAA GAGTCTACCA CATGCCCAGC
              CTGCCC
MAM1_SP134    GGGCCTCTCA GTCTCTCCAG AGTCTTTGGC CAGAGATAAC TGGGCTCCAC
              TCAGCCCGGG YGCTCTGTGT CTCCAGGTAA GAGTCTACCA CATGCCCAGC
              CTGCCC
MAST_B200_    GGGCCTCTCA GTCTCTCCAG AGTCTTTGGC CAGAGATAAC TGGGCTGCAC
              TCAGCCCGGG TGCTATATGT CTCCAGGTAA GAGTCTACCA CATGCCCAGC
              CTGCCC
SAV1_SE210    GGGCCTCTCA GTCTCTCCAG AGTCTTTGGC CAGAGATAAC TGGGCTCCAC
              TCAGCCCGGG TGCTCTGTGT CTCCAGGTAA GAGTCTACCA CATGCCCAGC
              CTGCCC

 1 1 1 1 1
5 106

ASIAN2_Ema    CGATAAGAGA TTGAAA-TC- TGAGTGCAAG AACGTC-TGA AACATTTTTC
              TAGGAATCCA TGTAGCAATT TCA-TAAACA AATGCTGTGG -AAAGGA-GC
              ATTCTA
FOR_DS1535    CGATAAGAGA TTGAAA-TC- TGAGTGCAAG AACGTC-TGA AACATTTTTC
              TAGGAATCCA TGTAGCAATT TCA-TAAACA AATGCTGTGG -AAAGGA-GC
              ATTCTA
MAM1_SP134    CGATAAGAGA TTGAAA-TC- TGAGTGCAAG AACGTC-TGA AACATTTTTC
              TAGGAATCCA TGTAGCAATT TCA-TAAACA AATGCTGTGG -AAAGGA-GC
              ATTCTA
MAST_B236_    TGATAAGAGA TTGAAAATCC TGAGTGCAAG AATGTCCTGA AACATTTTTT
              TAGGAATCCA TGTAGCAATT TCAGTAAGCA AATGCTGTGG TAAAGGACGC
              ATTCTA
SAV1_SE210    CGATAAGAGA TTGAAA-TC- TGAGTGCAAG AACGTC-TGA AACATTTTTC
              TAGGAATCCA TGTAGCAATT TCA-TAAACA AATGCTGTGG -AAAGGA-GC
```

```
            ATTCTA

 1 1 1 1 1
5 106

ASIAN2_Ema  AGTCCACAAA -GTTAGCCAT GTGAGCATGA ACCCTCAAGG GAGAAGGGCA
            AGCTGCGGTA GGCTCCTGAG GATAAGGACG CATACTCTGG TTTTAAACTC
            TCTTGG
FOR_DS1535  AGTCCACAAA -GTTAGCCAT GTGAGCATGA ACCCTCAAGG GAGAAGGGCA
            AGCTGCGGTA GGCTCCTGAG GATAAGGACG CATACTCTGG TTTTAAACTC
            TCTTGG
MAM1_SP134  AGTCCACAAA -GTTAGCCAT GTGAGCATGA ACCCTCAAGG GAGAAGGGCA
            AGCTGCGGTA GGCTCCTGAG GATAAGGACG CATACTCTGG TTTTAAACTC
            TCTTGG
MAST_B237_  AGTTCACAAA AGTTAGCCAT GTGAGCATGA ACCCTCAAGG GAGAAGGGCA
            AGCTGTGGTA GGCTCCTGAG GATAAGGATG CCTACTCTGG TTTTAAACTC
            TCTTAA
SAV1_SE210  AGTCCACAAA -GTTAGCCAT GTGAGCATGA ACCCTCAAGG GAGAAGGGCA
            AGCTGCGGTA GGCTCCTGAG GATAAGGACG CATACTCTGG TTTTAAACTC
            TCTTGG

 1 1 1 1 1
5 105

ASIAN2_Ema  GCATTATTAA TTTCTCTTAC CTGTCTCCTA GTTGTAATCT CAGATACTAC
            ATCCCTCAGC ATCTG-CAAA TGCCAGTAAC GAGTTTC-TC ATACTAAATA
            GAGAG
FOR_DS1535  GCATTATTAA TTTCTCTTAC CTGTCTCCTA GTTGTAATCT CAGATACTAC
            ATCCCTCAGC ATCTG-CAAA TGCCAGTAAC GAGTTTC-TC ATACTAAATA
            GAGAG
MAM1_SP134  GCATTATTAA TTTCTCTTAC CTGTCTCCTA GTTGTAATCT CAGATACTAC
            ATCCCTCAGC ATCTG-CAAA TGCCAGTAAC GAGTTTC-TC ATACTAAATA
            GAGAG
MAST_B240_  GCATTATTAA TTTCTCTTAC CTGTCTCCTA GTTGTAATCT CAGATACTAC
            ATCCCTCAGC ATCTGGCAAA TGCCAGTAAC GAGTTTCGTC ATACTAAATG
            GAGAT
SAV1_SE210  GCATTATTAA TTTCTCTTAC CTGTCTCCTA GTTGTAATCT CAGATACTAC
            ATCCCTCAGC ATCTG-CAAA TGCCAGTAAC GAGTTTC-TC ATACTAAATA
            GAGAG

 1 1 1 1 1
5 105

ASIAN2_Ema  TGGCCTACCC CAGCCCCTGT CATTTGAGGA TGACCCAAAG CGTTCACCCC
            C-AGGCCCC- TCC-TGCCTG CACAAGTGAA GGGCAGCTCT CAGGGGTACA
            GTCCT
FOR_DS1535  TGGCCTACCC CAGCCCCTGT CATTTGAGGA TGACCCAAAG SGTTCACCCC
            C-AGGCCCC- TCC-TGCCTG CACAAGTGAA GGGCAGCTCT CAGGGGTACA
            GTCCT
MAM1_SP134  TGGCCTACCC CAGCCCCTGT CATTTGAGGA TGACCCAAAG CGTTCACCCC
            C-AGGCCCC- TCC-TGCCTG CACAAGTGAA GGGCAGCTCT CAGGGGTACC
            GTCCT
MAST_B241_  GTTTTATTTC TAGTTCCTGT NATTTGAGGA TGACCCAAAG GGTTCACCCC
            CCAGGCCCCC TCCCTGCCTG CACAAGTGAA GGGCAGCTCT CAGGGGTACA
            GTCCT
SAV1_SE210  TGGCCTACCC CAGCCCCTGT CATTTGAGGA TGACCCAAAG GGTTCACCCC
            C-AGGCCCC- TCC-TGCCTG CACAAGTGAA GGGCAGCTCT CAGGGGTACA
            GTCCT

 1 1 1 1 1
5 105

ASIAN2_Ema  TAACCCACCC AGTGCTGTCG AGTTGATTAC TGACTCATAG CGACCCTAAA
            GGACAGAGTA GAACTGCCTC ATAGAGTTTC CAAGGAGTGC CTGGCGGATT
            CAAAC
FOR_DS1535  TAACCCACCC AGTGCTGTCG AGTTGATTAC TGACTCATAG CGACCCTAAA
            GGACAGAGTA GAACTGCCTC ATAGAGTTTC CAAGGAGTGC CTGGCGGATT
            CAAAC
MAM1_SP134  TAACCCACCC AGTGCTGTCG AGTTGATTAC TGACTCATAG CGACCCTAAA
            GGACAGAGTA GAACTGCCTC ATAGAGTTTC CAAGGAGTGC CTGGCGGATT
            CAAAC
```

```
MAST_B242_    TAACCCACCC AGTGCTGTCG AGTTGATTAC TGACTCATAG CGACCCTGTA
              GGACAGAGTA GAATTGCCTT ATAGAGTTTC CAAGGAGCGT CTGGCAGATT
              CAAAC
SAV1_SE210    TAACCCACCC AGTGCTGTCG AGTTGATTAC TGACTCATAG CGACCCTAAA
              GGACAGAGTA GAACTGCCTC ATAGAGTTTC CAAGGAGTGC CTGGCGGATT
              CAAAC

 1 1 1 1 1
5 105

ASIAN2_Ema    GTGCCCGACC TCAGTCAACT GCTGAGAGTG TTCTTAAGTC CTGTGTGTAT
              GTCTGTATAC ATCTGACCAC ACGTCACTCC TTTCTCTCTC TTCTTAGTAC
              AGAAG
FOR_DS1535    GTGCCCGACC TCAGTCAACT GCTGAGAGTG TTCTTAAGTC CTGTGTGTAT
              GTCCGTATAC ATCTGACCAC ACGTCACTCC TTTCTCTCTC TTCTTAGTAC
              AGAAG
MAM1_SP134    GTGCCCGACC TCAGTCAACT GCTGAGAGTG TTCTTAAGTC CTGTGTGTAT
              GTCCATATAC ATCTGACCAC ACGTCACTCC TTTCTCTCTC TTCTTAGTAC
              AGAAG
MAST_B243_    GTGCCCGACC TCAGTCAATT GCTGAGAGTG TTCTTAAGTC CTGTGTGTAT
              GTCTGTATAC ATCTGACCAC ACGTCACTCC TTTCTCTCTC TTCTTAGTAC
              AAAAA
SAV1_SE210    GTGCCCGACC TCAGTCAACT GCTGAGAGTG TTCTTAAGTC CTGTGTGTAT
              GTCCGTATAC ATCTGACCAC ACGTCACTCC TTTCTCTCTC TTCTTAGTAC
              AGAAG

 1 1 1 1 1
5 106

ASIAN2_Ema    TTCTTAACTG CAGCCTGGAG AAAAGTGGTG TCAGAAAACA AATATTTGGG
              AAACAGTAGC AGAGAATCAA TAGACATATC ACACCGACAG AGTGATGTCC
              ATGGGG
FOR_DS1535    TTCTTAACTG CAGCCTGGAG AAAAGTGGTG TCAGAAAAGA AATATTTGGG
              AAAGAGTAGC AGAGAATCAA TAGACATATC ACAGCAACAG AGTGATGTCC
              ATGGGG
MAM1_SP134    TTCTTAACTG CAGCCTGGAG AAAAGTGGTG TCAGAAAAGA AATATTTGGG
              AAAGAGTAGC AGAGAATCAA TAGACATATC ACAGCGACAG AGTGATGTCC
              ATGGGG
MAST_B244_    TTCTTAACTG CAGCCTGGAG AAAAGTGGTG TCAGAAAAGA AATATTTGG-
              AAAGAGGAGC AGAGAATCAC TAGACATATC ACAGCGACAG AGTGATGTCC
              ATGGAA
SAV1_SE210    TTCTTAACTG CAGCCTGGAG AAAAGTGGTG TCAGAAAAGA AATATTTGGG
              AAAGAGTAGC AGAGAATCAA TAGACATATC ACAGCGACAG AGTGATGTCC
              ATGGGG

 1 1 1 1 1
5 106

ASIAN2_Ema    AGGTGACAGC TGGGTCTGCA AGTCTGTCCT TTCTGATGGC CACCAGCGCA
              CCGTGCGGAA GGTAGCCTGG TCTCCCTGTG GGAATTACCT GGCGTCTGCC
              AGCTTC
FOR_DS1535    AGGTGACAGC TGGGTCTGCA AGTCTGTCCT TTCTGATGGC CACCAGCGCA
              CCGTGCGGAA GGTAGCCTGG TCTCCCTGTG GGAATTACCT GGCGTCTGCC
              AGCTTC
MAM1_SP134    AGGTGACAGC TGGGTCTGCA AGTCTGTCCT TTCTGATGGC CACCAGCGCA
              CCGTGCGGAA GGTAGCCTGG TCTCCCTGTG GGAATTACCT GGCGTCTGCC
              AGCTTC
MAST_B245_    AGGTGAGAGC TGGGTCTGCA AGTCTGTCCT TTCTGATGGC CACCAGCGCA
              CTGTGCGGAA GGTAACCTGG TCTCCCTATG G-AATTACCT GGCGTCTGCC
              AGCTTC
SAV1_SE210    AGGTGACAGC TGGGTCTGCA AGTCTGTCCT TTCTGATGGC CACCAGCGCA
              CCGTGCGGAA GGTAGCCTGG TCTCCCTGTG GGAATTACCT GGCGTCTGCC
              AGCTTC

 1 1 1 1 1
5 105

ASIAN2_Ema    GCCCCGCC-T CCTGGCAGGT CGATCACCTT GACTGGTCGA GGCACCCGCA
              CTAGGCCTGT GCTCTGGAGG GCCTCCAGGC TGGCCATCTC CCCCTCGAAC
              ATCTG
FOR_DS1535    GCCCCGCC-T CCTGGCAGGT CGATCACCTT GACTGGTCGA GGCACCCGCA
```

```
               CTAGGCCTGT GCTCTGGAGG GCCTCCAGGC TGGCCATCTC CCCCTCGAAC
               ATCTG
MAM1_SP134     GCCCCGCC-T CCTGGCAGGT CGATCACCTT GACTGGTCGA GGCACCCGCA
               CTAGGCCTGT ACTCTGGAGG GCCTCCAGGC TGGCCATCTC CCCCTCGAAC
               ATCTG
MAST_B246_     GCCCCGCCCT CCTGGCAGGT CGATCACCTT GACTGGTCGA GGCACTCGCA
               CTAGGCCTGT GCTCTGGAGG GCCTCCAGGC TGGCCATCTC CCCCTCGAAC
               ATCTG
SAV1_SE210     GCCCCGCC-T CCTGGCAGGT CGATCACCTT GACTGGTCGA GGCACCCGCA
               CTAGGCCTGT GCTCTGGAGG GCCTCCAGGC TGGCCATCTC CCCCTCGAAC
               ATCTG

 1 1 1 1 1
5 105

ASIAN2_Ema     AACGAAGGTG TTGTAATCAC CTGTCAGACA GGTGATTAGA CCAGGCCATC
               TTCATCTCAC CTCAGGGAAT CTGTGACCTT TGCCGTTGCA CACGTGTCTG
               TCTGA
FOR_DS1535     AACGAAGGTG TTGTAATCAC CTGTCAGACA GGTGATTAGA CCAGGCCATC
               TTCATCTCAC CTCAGGGAAT CTGTGACCTT TGCCGTTGCA CACGTGTCTG
               TCTGA
MAM1_SP134     AACGAAGGTG TTGTAATCAC CTGTCAGACA GGTGATTAGA CCAGGCCATC
               TTCATCTCAC CTCAGGGAAT CTGTGACCTT TGCCGTTGCA CACGTGTCTG
               TCTGA
MAST_B203_     AACAAAGGTG TTGTAATCAC CTGTCAGACA GGTGATTAGA CCAGGCCAGC
               TTCATCTCAC CTCAGGGAAT CTGTGACCTT TGCCGTTGCA CACGTGTCTG
               TCTGA
SAV1_SE210     AACGAAGGTG TTGTAATCAC CTGTCAGACA GGTGATTAGA CCAGGCCATC
               TTCATCTCAC CTCAGGGAAT CTGTGACCTT TGCCGTTGCA CACGTGTCTG
               TCTGA

 1 1 1 1 1
5 106

ASIAN2_Ema     GTTAGGAAAC TTCCTCAGGG AGAAGGTGGC CAACAACGTG GCTTTTACCA
               GAAGCACTTG GAGCTTCTGG TCTTCCTGAC ACTTTATGTC TTCAATCTCA
               TCTCTC
FOR_DS1535     GTTAGGAAAC TTCCTCAGGG AGAAGGTGGC CAACAACGTG GCTTTTACCA
               GAAGCACTTG GAGCTTCTGG TCTTCCCGAC ACTTTAKGTC TTCAATCTCA
               TCTCTC
MAM1_SP134     GTTAGGAAAC TTCCTCAGGG AGAAGGTGGC CAACAACGTG GCTTTTACCA
               GAAGCACTTG GAGCTTCTGG TCTTCCCGAC ACTTTAKGTC TTCAATCTCA
               TCTCTC
MAST_B247_     GTTAGGAAAC TTC-TCAGGG AGAAGGTGGC CAACAACGTG GCTTTTACCA
               GAAGCACTTG GAGCTTCTGG TCTTCCCGAC ACTTTATGTC TTCAATCTCA
               TCTCTC
SAV1_SE210     GTTAGGAAAC TTCCTCAGGG AGAAGGTGGC CAACAACGTG GCTTTTACCA
               GAAGCACTTG GAGCTTCTGG TCTTCCTGAC ACTTTATGTC TTCAATCTCA
               TCTCTC

 1 1 1 1 1
5 105

ASIAN2_Ema     TCTGTCTTCC TGAGTCCTAG CATCCCTCAG CTGCTAATGT CTGAATTCTA
               ACTACCTTTC ATGACCCATC ATACAACATT GTTCCATTTT CTATGTAAGC
               ATCAT
FOR_DS1535     TCTGTCTTCC TGAGTCCTAG CATCCTTCAG CTGCTAATGT CTAAATTCTA
               ACTACCTTTC ATGACCCATC AAACAACATT GTTCCATTTT CTATGTAAGC
               ATCTT
MAM1_SP134     TCTGTCTTCC TGAGTCCTAG CATCCTTCAG CTGCTAACGT CTAAATTCTA
               ACTACCTTTC ATGACCCATC ATACAACATT GTTCCATTTT CTATGTAAGC
               ATCAT
MAST_B248_     TTTGTCTTCC TGAGTCCTAG CATCCTTCAG CTGCTAATGT CTAAATTCTA
               ACTACCTTTC ATGACCCATC ATACAACATT GTTCCATTTT CTATGTAAGC
               ATCAT
SAV1_SE210     TCTGTCTTCC TGAGTCCTAG CATCCTTCAG CTGCTAATGT CTAAATTCTA
               ACTACCTTTC ATGACCCATC ATACAACATT GTTCCATTTT CTATGTAAGC
               ATCAT

 1 1 1 1 1
5 105
```

ASIAN2_Ema TATGCCATCA TAAGCCTTGG GGACCAAGTC AGTTATGCTG AAAAATTCTG
CCCTGTCTTG CACATCCATA TTATTTCTAA CATGATTTTG TATCTTTGCT
AATAA
FOR_DS1535 TATGCCATCA TAAGCCTTGG GGACCAAGTC AGTTATGCTG AAAAATTCTG
CCCTGTCTTG CACATCCATA TTATTTCTAA CATGATTTTG TATCTTTGCT
ARTAA
MAM1_SP134 TATGCCATCA TAAGCCTTGG GGACCAAGTC AGTTATGCTG AAAAATTCTG
CCCTGTCTTG CACATCCATA TTATTTCTAA CATGATTTTG TATCTTTGCT
AATAA
MAST_B250_ TATGCCTTCA TAAGCCTTGG GGACCAAGTC AGTTATGCTG AAAAATTCTG
CCCTGTCTTG CACATCCATA TTATTTCTAA CATGATTTTG TATCTTTGCT
AATAA
SAV1_SE210 TATGCCATCA TAAGCCTTGG GGACCAAGTC AGTTATGCTG AAAAATTCTG
CCCTGTCTTG CACATCCATA TTATTTCTAA CATGATTTTG TATATTTGCT
AATAA

1 1 1 1 1
5 104

ASIAN2_Ema CCCATGGCAC ATTTCAAAGT CTCAGCATTT GTGCTCACTG GCTGATAGGA
CCTCCTTTTC TGGTATAGTT TTGGACCATT GAGAGCCAGG AGACAAAGRG
CCAT
FOR_DS1535 CCCATGGCAC ATTTCAAAGT CTCAGCATTT GTGCTCACTG GCTGATAGGA
CCTCCTTTTC TGGTATAGTT TTGGACCATT GAGAGCCAGG AGACAAAGAG
CCAT
MAM1_SP134 CCCATGGCAC ATTTCAAAGT CTCAGCATTT GTGCTCACTG GCTGATAGGR
CCTCCTTTTC TGGTATAGTT TTGGACCATT GAGAGCCAGG AGACAAAGAG
CCAT
MAST_B251_ CCCATGGCAC ATTTCAAAGT CTCAGCATTT GTGCTCACTG GCTGATAGGA
CCTCCTTTTC TGGTATAGTT ATGGACCATT GAGAGCCAGG AGACAAAAAG
CCAT
SAV1_SE210 CCCATGGCAC ATTTCAAAGT CTCAGCATTT GTGCTCACTG GCTGATAGGA
CCTCCTTTTC TGGTATAGTT ATGGACCATT GAGAGCCAGG AGAAAAACAG
CCAT

1 1 1 1 1
5 104

ASIAN2_Ema TCCCTTGGAG GTGAAAAGTG CTGCTGACTC AAAGCCTCCA GACTAAAAGA
GACTTGGAAG AAAGGACTGG TGATCTACAT CTGAAAATTC ACCAATGAAA
ACAC
FOR_DS1535 TCCCTCGGAG GTGAAAAGTG CTGCTGACTC AAAGCCTCCA GACTAAAAGA
GACTTGGAAG AAAGGCCTGG TGATCTACAT CTGAAAATTC ACCAATGAAA
ACAC
MAM1_SP134 TCCCTCGGAG GTGAAAAGTG CTGCTGACTC AAAGCCTCCA GACTAAAAGA
GACTTGGAAG AAAGGCCTGG TGATCTACAT CTGAAAATTC ACCAATGAAA
ACAC
MAST_B205_ TCCCTCAGAG GTGAAAAGTG CTGCTGACTC AAAGCCTCCA GACTAAAAGA
GACTTGGAAG AAAGGCCTGG TGATCTATAT CTGAAAATTC ACCAATGAAA
ACAC
SAV1_SE210 TCCCTCGGAG GTGAAAAGTG CTGCTGACTC AAAGCCTCCA GACTAAAAGA
GACTTGGAAG AAAGGCCTGG TGATCTACAT CTGAAAATTC ACCAATGAAA
ACAC

1 1 1 1 1
5 104

ASIAN2_Ema CTCAACTTGT ACTGGCTTCA GAGACAACCA CATATATATT CGTATACTTC
TTGGCCGATA CTGTGAACAG TGCTATCCAC TGGTACTGCA AAATTAGGCT
AAGG
FOR_DS1535 CTCAACTTGT ACTGGCTTCA GAGACAACCA CATATATATT CGTATACTTC
TTGGCCGATA CTGTGAACAG TGCTATCCAC TGGTACTGCA AAATTAGGCT
AAGG
MAM1_SP134 CTCAACTTGT ACTGGCTTCA GAGACAACCA CATATATATT CGTATACTTC
TTGGCCGATA CTGTGAACAG TGCTATCCAC TGGTACTGCA AAATTAGGCT
AAGG
MAST_B253_ CTTAACTTGT ACTGGCTTCA GAGACAACCA CATATATATT CGTATACTTC
TTGGCCAATA CTGTGAACAG TGCTATCCAC TGGTACTGCA AAATTAGGCT
AAGA
SAV1_SE210 CTCAACTTGT ACTGGCTTCA GAGACAACCA CATATRTATT CGTATACTTC

TTGGCCGATA CTGTGAACAG TGCTATCCAC TGGTACTGCA AAATTAGGCT
AAGG

1 1 1 1 1
5 104

ASIAN2_Ema CCAGAGAAAC TCACCTTTGA GTGGCCAAAT GGGGATGAAC AGGTGATAAG
ACAGTTCAGG GTAGAGGTGT GTGCACAGAC ATCAA-TAAG AGACTCCTTT
CCAT
FOR_DS1535 CCAGAGAAAC TCACCTTTGA GTGGCCAAAT GGGGATGAAC AGGTGATAAG
ACAGTTCAGG GTAGAGGTGT GTGCACAGAC ATCAA-TAAG AGACTCCTTT
CCAT
MAM1_SP134 CCAGAGAAAC TCACCTTTGA GTGGCCAAAT GGGGATGAAC AGGTGATAAG
ACAGTTCAGG GTAGAGGTGT GTGCACAGAC ATCAA-TAAG AGACTCCTTT
CCAT
MAST_B256_ CCAGAAAAAC TCACCTTTGA GTGGCCAAAT GGGGATGAAC AGGTGATAAG
ACAGTTCAGG GTAGAGATGT GTGCACAGAC ATCAAATAAA AGACTCCTTT
CCAT
SAV1_SE210 CCAGAGAAAC TCACCTTTGA GTGGCCAAAT GGGGATGAAC AGGTGATAAG
ACAGTTCAGG GTAGAGGTGT GTGCACAGAC ATCAA-TAAG AGACTCCTTT
CCAT

1 1 1 1 1
5 104

ASIAN2_Ema TATAAGTCCC ATCTAGCTGA CACACCCTCG GTGGCCCAGC AGGGCTCCAA
CAACCCTTGT GAGCACCTCC CGTGTGCCGG ACACCGTGCT AAGAGCTAGG
AATG
FOR_DS1535 TATAAGTCCC ATCTGGCTGA CACACCCTCG GTGGCCCAGC AGGGCTCCAA
CAACCCTTGT GAGCACATCC -GTGTGCCGG ACACCATGCT AAGAGCTAGG
AATG
MAM1_SP134 TATAAGTCCC ATCTGGCTGA CACACCCTCG GTGGCCCAGC AGGGCTCCAA
CAACCCTTGT GAGCACCTCC CGTGTGCCGG ACACCGTGCT AAGAGCTAGG
AATG
MAST_B207_ TATAAGTCCT ATCTGGCTGA CACACCCTCG GTGGCCCAGC AGGGCTCCAG
TAACCCTTGT GAGCACCTCC CGTGTGCCGG ATACCGTGTT AAGAGCTAGG
AATA
SAV1_SE210 TATAAGTCCC ATCTGGCTGA CACACCCTCG GTGGCCCAGC AGGGCTCCAA
CAACCCTTGT GAGCACATCC -GTGTGCCGG ACACCATGCT AAGAGCTAGG
AATG

1 1 1 1 1
5 104

ASIAN2_Ema TTTCTGGAGC CCTGGTGGTG C-AGTGGTTA AGAGCTATGG CTGCTAACCA
AAAGGTCCAC AGTTCAAACC CACCAGCCAT TCCTTGGAAA CTCTATAGGG
GCAG
FOR_DS1535 TTTCTGGAGC CCTGGTGGTG C-AGTGGTTA AGAGCTATGG CTGCTAACCA
AAAGGTCCAC AGTTCAAACC CACCAGCCAT TCCTTGGAAA CTCTATAGGG
GCAG
MAM1_SP134 TTTCTGGAGC CCTGGTGGTG C-AGTGGTTA AGAGCTATGG CTGCTAACCA
AAAGGTCCAC AGTTCAAACC CACCAGCCAT TCCTTGGAAA CTCTAYAGGG
GCAG
MAST_B259_ TCTTTGGAGC CCTGGTGGTG CCAGTGGTTA AGAGCTATGG CTACAAACCA
AAAGGTCCAC AGTTCAAATC CACCAGCCAT TCCTTGGAAA TCCGCGTGGG
TAAT
SAV1_SE210 TTTCTGGAGC CCTGGTGGTG C-AGTGGTTA AGAGCTATGG CTGCTAACCA
AAAGGTCCAC AGTTCAAACC CACCAACCAT TCCTTGGAAA CTCTATAGGG
GCAG

1 1 1 1 1
5 104

ASIAN2_Ema TGGCTCCCTC CGAAGAAAGG GTGGAAGAAG GGCGGGTCCA GCAACCACAC
TGCTGTGTCC CCTTGGGGGT GGGGAGGCTG GAGGGCACAG GAGGAGTTCC
AGAG
FOR_DS1535 TGGCTCCCTC CGAAGAAAGG GTGGAAGAAG GGCGGGTCCA GCAACCACAC
TGCTGGGTCC CCTTGGGGGT GGGGAGGCTG GAGGGCACAG GAGGAGTTCC
AGAG
MAM1_SP134 TGGCTCCCTC CGAAGAAAGG GTGGAAGAAG GGCGGGTCCA GCAACCACAC
TGCTGTGTCC CCTTGGGGGT GGGGAGGCTG GAGGGCACAG GAGGAGTTCC

```
              AGAG
MAST_B260_    TGGTTCCCTC CGAAGAAAGG GTGGAAGAAG GGCGGGTCCA GCAACCACAC
              TGCTGTGTCC CCTTGGGGGT GGGAAGGCTG AAAGACACAA GAAAAATTCC
              AAAA
SAV1_SE210    TGGCTCCCTC CAAAGAAAGG GTGGAAGAAG GGCGGGTCCA GCAAYCACAC
              TGCTGGGTCC CCTTGGGGGT GGGGAGGCTG GAGGGCACAG GAGGAGTTCC
              AGAG

 1 1 1 1 1
5 104

ASIAN2_Ema    CAAATCCAGA GGAAGGCTAG AGGAAGTCAA TTCTAGAAGC TGTCTCAGAA
              CAGTGAGGAA GAACTTT-CC AA-GAAGCCA CCAAAAAACC AAACTGGGTC
              ATAG
FOR_DS1535    CAAATCCAGA GGAAGGCTAG AGGAAGTCAA TTCTAGAAGC TGTCTCAGAA
              CAGTGAGGAA GAACTTT-CC AA-GAAGCCA CCAAAAAACC AAACTGGGTC
              ATAG
MAM1_SP134    CAAATCCAGA GGAAGGCTAG AGGAAGTCAA TTCTAGAAGC TGTCTCAGAA
              CAGTGAGGAA GAACTTT-CC AA-GAAGCCA CCAAAAAACC AAACTGGGTC
              ATAG
MAST_B261_    TAAATCCAGA GGAAGGCTAG AGGAAGTCAA TTCTAGAAGC TGTCTCAGAA
              CAGTGAGGAA GAACTTTTCC AAAGAAGCCA CCAAAAAACC AAACTGGGTC
              ATAA
SAV1_SE210    CAAATCCAGA GGAAGGCTAG AGGAAGTCAA TTCTAGAAGC TGTCTCAGAA
              CAGTGAGGAA GAACTTT-CC AA-GAAGCCA CCAAAAAACC AAACTGGGTC
              ATAG

 1 1 1 1 1
5 104

ASIAN2_Ema    CCYGGCCTGG AGCAAGTAAG GCTCTCAACT CACGAACTGC ACACTTTTAA
              ACTCACTGTC ACTTTGGGCT CAGATCCTTA GACTTCGGGA GCCGGTTTCT
              TATG
FOR_DS1535    CCCGGCCTGG AGCAAGTAAG GCTCTCAACT CACGAACTGC ACACTTTTAA
              ACTCACTGTC ACTTTGGGCT CAGATCCTTA GACTTCGGGA GCCGGTTTCT
              TATG
MAM1_SP134    CCCGGCCTGG AGCAAGTAAG GCTCTCAACT CACGAACTGC ACACTTTTAA
              ACTCACTGTC ACTTTGGGCT CAGATCCTTA GACTTCGGGA GCCGGTTTCT
              TATG
MAST_B262_    TCCGGCTTGG AGCAAGTAAG GCTCTCACCT CACGAACTGC ACACTTATAA
              ACTCACTGTC ACTTTGGGCT CAGATCCTTA GACTTCGGGA GCCAGTTTCT
              TATA
SAV1_SE210    CCCGGCCTGG AGCAAGTAAG GCTCTCAACT CAGGAACTGC ACACTTTTAA
              ACTCACTGTC ACTTTGGGCT CAGATCCTTA GACTTCGGGA GCCGGTYTCT
              TATG

 1 1 1 1 1
5 50

ASIAN2_Ema    TAAGGTGGGG TGCCCCAGCG CACACCTCTG ATGAAAACAG GCTACGCACG
FOR_DS1535    TAAGGTGGGG TGCCCCAGCG CACACCTCTG ATGAAAACAG GCTACGCACG
MAM1_SP134    TAAGGTGGGG TGCCCCAGCG CACACCTCTG ATGAAAACAG GCTACGCACG
MAST_B264_    TAAGGTGGGG TGCCCCAGCG CACACCTCTG ATGAAAACAG GCTACGCACG
SAV1_SE210    TAAGGTGGGG TGCCCCAGCG CACACCTCTG ATGAAAACAG GCTACGCACG

 1 1 1 1 1
5 104

ASIAN2_Ema    CAGAAAGCTC C-GGAAGCCT TGCCAACAGC CCTGGAGGCT GAGTCCAGAA
              ATAGAAGACC CCCAGAGAGA AAGGCTGCTG ATGGCCAGGC TGCCCTCTGC
              CAGC
FOR_DS1535    CAGAAAGCTC C-GGAAGCCT TGCCAACAGC CCTGGAGGCT GAGTCCAGAA
              ATAGAAGACC CCCAGAGAGA AAGGCTGCTG ATGGCCAGGC TGCCCTCTGC
              CAGC
MAM1_SP134    CAGAAAGCTC C-GGAAGCCT TGCCAACAGC CCTGGAGGCT GAGTCCAGAA
              ATAGAAGACC CCCAGAGAGA AAGGCTGCTG ATGGCCAGGC TGCCCTCTGC
              CAGC
MAST_B209_    CAGAAAGTTT TTAGAAGTTT TGCCAACAGC CCTGGAGGCT GAGTCCAGAA
              ATAGAAGACC CCCAGAGAGA AAGGCTGCTG ATGGCCAGGC TGCCCTCTGC
              CAGC
SAV1_SE210    CAGAAAGCTC C-GGAAGCCT TGCCAACAGC CCTGGAGGCT GAGTCCAGAA
```

ATAGAAGACC CCCAGAGAGA AAGGCTGCTG ATGGCCAGGC TGCCCTCTGC
CAGC

1 1 1 1 1
5 104

ASIAN2_Ema GCTTATTCTT CTTTCCTGTT GAAAGAAAAA -GCTTT-AAT TTCAAGCTCT
GTGGGATAGC CTCTTCTCTC TCCTTCCTCA TCTCCATTTT AATCAATTAA
TGAT
FOR_DS1535 GCTTATTCTT CTTTCCTGTT GAAAGAAAAA -GCTTT-AAT TTCAAGCTCT
GTGGGATAGC CTCTTCTCTC TCCTTCCTCA TCTCCATTTT AATCAATTAA
TRAT
MAM1_SP134 GCTTATTCTT CTTTCCTGTT GAAAGAAAAA -GCTTT-AAT TTCAAGCTCT
GTGGGATAGC CTCTTCTCTC TCCTTCCTCA TCTCCATTTT AATCAATTAA
TGAT
MAST_B210_ GCTTATTCTT CTTTCCTGTT GAAAGAAAAA AGCTTTTAAT TTCAAGCTCT
GTGGGATAGC CTCTTCTCTT TCCTTCCTCA TCTCCATTTT AATCAATTAA
TGAT
SAV1_SE210 GCTTATTCTT CTTTCCTGTT GAAAGAAAAA -GCTTT-AAT TTCAAGCTCT
GTGGGATAGC CTCTTCTCTC TCCTTCCTCA TCTCCATTTT AATCAATTAA
TGAT

1 1 1 1 1
5 104

ASIAN2_Ema CTCAGGGTCA GGAGATGGAT GTGAGTCTAA ACGTTGGCCT TCGTAACTGT
GACCATTTTA AGTTCTTTAA TCACCCTGAT CTCAATTCCT TATCCTTATT
TTAC
FOR_DS1535 CTCAGGGTCA GGAGATGGAT GTGAGTCTAA ACGTTGGCCT TCGTAACTGT
GACCATTTTA ARTTCTTTAA TCACCCTGAG CTCAATTCCT TATCCTTATT
TTAC
MAM1_SP134 CTCAGGGTCA GGAGATGGAT GTGAGTCTAA ACGTTGGCCT TCGTAACTGT
GACCATTTTA AGTTCTTTAA TCACCCTGAT CTCAATTCCT TATCCTTATT
TTAC
MAST_B265_ CTCAGGGTCA GGAGATGGAT CTGAGTCTAA ACGTTGGCCT TCGTAACTGT
GACCATTTTA AGTTCTTTAA TCACCCTGAG CTCAATTCCT TATCCTTATT
TTAC
SAV1_SE210 CTCAGGGTCA GGAGATGGAT GTGAGTCTAA ACGTTGGCCT TCGTAACTGT
GACCATTTTA AGTTCTTTAA TCACCCTGAG CTCAATTCCT TATCCTTATG
TTAC

1 1 1 1 1
5 103

ASIAN2_Ema TCAGGAGCTC TCTGGTCCTG CAGCTCTGTC CTCGGCCAAG CACAGCCCAG
TCTGCTAGAT ATCTGCAGAC AAAGTTCCCG TGCACCTCCA ATGGCCTCAT
CTC
FOR_DS1535 TCAGGAGCTC TCTGCTCCTG CAGCTCTGTC CTCGGCCAAG GACAGCCCAG
TCTGCTAGAC ATCTGCAGAC AAAGTTCCCG TGCACCTCCA ATGGCCTCAT
CTC
MAM1_SP134 TCAGGAGCTC TCTGGTCCTG CAGCTCTGTC CTCGGCCAAG GACAGCCCAG
TCTGCTAGAT ATCTGCAGAC AAAGTTCCCG TGCACCTCCA ATGGCCTCAT
CTC
MAST_B267_ TCAGGAGCTC TCTGCTCCTG CAGCTCTGTC CTCGGCCAAG GACAGCCCAG
TCTGCTAGAC ATTTGCAGAC AGACTTTCCG TGCACCTCCC ATGGCCTCAT
CTC
SAV1_SE210 TCAGGAGCTC TCTGCTCCTG CAGCTCTGTC CTCGGCCAAG GACAGCCCAG
TCTGCTAGAC ATCTGCAGAC AAAGTTCCCG TGCACCTCCA ATGGCCTCAT
CTC

1 1 1 1 1
5 103

ASIAN2_Ema TGTGGTTATT TAATAAATGT CACTGTCCCC ACTAGACTGC AAGCTGCATG
AGGTCARGAC CATATCTGTG TTGGTCACCA GGGTATTCCT AGTGCCTAGC
ACC
FOR_DS1535 TGTGGTTATT TAATAAATGT CACTGTCCCC ACTAGACTGC AAGCTGCATG
AGGTCAGGAC CATATCTGTG TTGGTCACCA GGGTATTCCT AGTGCCTAGC
ACC
MAM1_SP134 TGTGGTTATT TAATAAATGT CACTGTCCCC ACTAGACTGC AAGCTGCATG
AGGTCAGGAC CATATCTGTG TTGGTCACCA GGGTATTCCT AGTGCCTAGC

ACC
MAST_B269_ TGTGGTTATT TAATAAATGT CACTGTCCCC ACTAGACTGC AAGCTGTATG
AGGTCAGGAC CTTATCTGTG TTGGTCACCA GGGTATTCCC AGTGCCTAGC
ACC
SAV1_SE210 TGTGGTTATT TAATAAATGT CACTGTCCCC ACTAGACTGC AAGCTGCATG
AGGTCAGGAC CATATCTGTG TTGGTCACCA GGGTATTCCT AGTGCCTAGC
ACC

1 1 1 1 1
5 106

ASIAN2_Ema AAGTGTTTAT CCATATAGTG TGACTCACAG ATGTCCCCCC ACACACACAC
ATACACACAC TCATGCACAC ACATTGCCCC TAGTCCCAGC TGAGCTCACA
GTGGCT
FOR_DS1535 AAGTGTTTAT CCATATAGTG TGACTCACAG ATGTCCCCCC ACACACACAC
ATACACACAC TCATGCACAC ACATTGCCCC TAGTCCCAGC TGATCTCACA
GTGGCT
MAM1_SP134 AAGTGTTTAT CCATATAGTG TGACTCACAG ATGTCCCCCC ACACACACAC
ATACACACAC TCATGCACAC ACATTGCCCC TAGTCCCAGC TGAGCTCACA
GTGGCT
MAST_B211_ AAGTGTTTAT CCATATAGTG TGACTCACAG ATGTCCCCCC C-ACACACAC
ATACACACAC TCATGCACAC ACATTGCCC- TAGTCC-AGC TGATCTCACA
GTGGCT
SAV1_SE210 AAGTGTTTAT CCATATAGTG TGACTCACAG ATGTCCCCCC CCACACACAC
ATACACACAC TCATGCACAC ACATTGCCCC TAGTCCCAGC TGAGCTCACA
GTGGCT

1 1 1 1 1
5 103

ASIAN2_Ema AACATTTGAG CCAAGATCTC CCTGTCTAGT TTGAAGCTGA GACAGTTGAA
CAGAAGGTCT TGTTCACATC TGGGCAGCCG CTCTTCTTTT TATCTTTATT
TCA
FOR_DS1535 AACATTTGAG CCAAGATCTC CCTGTCTAGT TTGAAGCTGA GACAGTTGAA
CAGAAGGTCT TGTTCACATC TGGGCAGCCG CTCTTCTTTT TATCTTTATT
TCA
MAM1_SP134 AACATTTGAG CCAAGATCTC CCTGTCTAGT TTGAAGCTGA GACAGTTGAA
CAGAAGGTCT TGTTCACATC TGGGCAGCCG CTCTTCTTTT TATCTTTATT
TCA
MAST_B212_ AACATTTGAG CCAAGATCTC TTTGTCTAGT TTGAAGCTGA GACAGTTGAA
CAGAAGGTCT TGTTCACATC TGGGCAGCTG CTCTTCTTTT TATCTTTATT
TCA
SAV1_SE210 AACATTTGAG CCAAGATCTC CCTGTCTAGT TTGAAGCTGA GACAGTTGAA
CAGAAGGTCT TGTTCACATC TGGGCAGCCG CTCTCCTTTT TATCTTTATT
TCA

1 1 1 1 1
5 104

ASIAN2_Ema TGACCCCTCC AGACA-TTTG AGAGACACCA AGCCTTTGAG AAAGAGAAAG
TAGACCTT-A TTGCAAGCTG GTCAAGCAAG GAGCTCAGAG ACTAAGTCTC
AAAT
FOR_DS1535 TGACCCCTCC AGACA-TTTG AGAGACACCA GGCCTTTGAG AAAGAGAAAG
TAGACCTT-A TTGCAAGCTG GTCAAGCAAG GAGCTCAGAG ACTAAGTCTC
AAAT
MAM1_SP134 TGACCCCTCC AGACA-TTTG AGAGACACCA AGCCTTTGAG AAAGAGAAAG
TAGACCTT-A TTGCAAGCTG GTCAAGCAAG GAGCTCAGAG ACTAAGTCTC
AAAT
MAST_B271_ TTCGCCCTCC AGTCAATTAG AGAAACACTA GGCCTTTGAG GAGGAGAAAG
T-GACCTTTA CCGTAAGCTG GTCAAGCAAG GAGCTCAGAG ACTAAGTCTC
AAAC
SAV1_SE210 TGACCCCTCC AGACA-TTTG AGAGACACCA GGCCTTTGAG AAAGAGAAAG
TAGACCTT-A TTGCAAGCTG GTCAAGCAAG GAGCTCAGAG ACTAAGTCTC
AAAT

1 1 1 1 1
5 103

ASIAN2_Ema TCCATTAAAR CCATGATGTA CAGGCCTTGA GTCAGAAAGA GCTGGGGAAG
AGCAATAGAT CATCAAACTG GCTGCACAGT TTCAGCAAA- GAAAGCCAGT
CAG

FOR_DS1535 TCCATTAAAG CCATGATGCA CAGGCCTTGA GTCAGAAAGA GCTGGGGAAG
AGCAATAGAT CATCAAACTG GCTGCACAGT TTCAGCAAA- GAAAGCCAGT
CAG
MAM1_SP134 TCCATTAAAG CCATGATGTA CAGGCCTTGA GTCAGAAAGA GCTGGGGAAG
AGCAATAGAT CATCAAACTG GCTGCACAGT TTCAGCAAA- GAAAGCCAGT
CAG
MAST_B273_ TCCATTAAAG CCATGATGCA CAGGCCCTGA GTCAGAAAGA GCTGGGGAAG
AGCAACAGAT CATCAAATTG GCTACACAAT TTCAGCAAAA GAAAACCAGT
CAA
SAV1_SE210 TCCATTAAAG CCATGATGCA CAGGCCTTGA GTCAGAAAGA GCTGGGGAAG
AGCAATAGAT CATCAAACTG GCTGCACAGT TTCAGCAAA- GAAAGCCAGT
CAG

1 1 1 1 1
5 103

ASIAN2_Ema CTCACTCCCC AGTCAGGGTG CAGCCAGGTA GAAATGGAAG TCACCGTCCA
GGTCCCCAAG GTCCCTGGGC CATGATGCCC CCCAGAACGA ACCTGTTGTT
GGT
FOR_DS1535 CTCACTCCCC AGTCAGGGTG CAGCCAGGTA GAAATGGAAG TCACCGTCCA
GGTCCCCAAG GTCCCTGGGC CATGATGCCC CCCAGAACGA ACCTGTTGTT
GGT
MAM1_SP134 CTCACTCCCC AGTCAGGGCG CAGCCAGGTA GAAATGGAAG TCACCGTCCA
GGTCCCCAAG GTCCCTGGGC CATGATGCCC CCCAGAACGA ACCTGTTGTT
GGT
MAST_B214_ TTCACTCCCC AGTCAGGGTG CAGCCAGGTA GAAATGGAAG TCACCGTCCA
GGTCCCCAAG GTCCCTGGGC CATGATCGCC CCCAGAACGA ACCTGTTGTT
GGT
SAV1_SE210 CTCACTCCCC AGTCMGGGTG CAGCCAGGTA GAAATGGAAG TCACCGTCCA
GGTCCCCAAG GTCCCTGGGC CATGATGCCC CCCAGAACGA ACCTGTTGTT
GGT

1 1 1 1 1
5 103

ASIAN2_Ema AGGTCTCTTT CTTCATGTCG CCTTGGCCCA AACATCTAGG CTTACCACTT
AGGCTTTTCC TAACTTCCCT TCCCAGTGGG ATTCTTAATT AGGTAGGTTT
AGC
FOR_DS1535 AGGTCTCTTT CTTCATGTCG CCTTGGCCCA AACATCTAGG CTTACCACTT
AGGCTTTTCC TAACTTCCCT TCCCAGTGGG ATTCTTAATT AGGTAGGTTT
AGC
MAM1_SP134 AGGTCTCTTT CTTCATGTCG CCTTGGCCCA AACATCTAGG CTTACCACTT
AGGCTTTTCC TAACTTCCCT TCCCAGTGGG ATTCTTAATT AGGTAGGTTT
AGC
MAST_B277_ AGGTCTCTTT CTTTATGTCG CCTTGGCCCA AACATCTAGG CTTACCACTT
AGGCTTTTCC TAACTTCCCT TCCCAATGAA ATTCTTAATT AAATAAATTT
AAC
SAV1_SE210 AGGTCTCTTT CTTCATGTCG CCTTGGCCCA AACATCTAGG CTTACCACTT
AGGCTTTTCC TAACTTCCCT TCCCAGTGGG ATTCTTAATT AGGTAGGTTT
AGC

1 1 1 1 1
5 103

ASIAN2_Ema CTGCTAGGCA GTTTTCAAAG CATCTTTGAG TATATTGGGG CCCGCCCAGG
GTGGGARCTG CTCAGAGAAA TGATACTGTA TGTGGAGGGG TGCGCTAGCC
AGC
FOR_DS1535 CTGCTAGGCA GTTTTCAAAG CATCTTTGAG TATATTGGGG CCCGCCCAGG
GTGGGAGCTG CTCAGAGAAA TGATACTGTA TGTGGAGGGG TGCGCTGGCC
AGC
MAM1_SP134 CTGCTAGGCA GTTTTCAAAG CATCTTTGAG TATATTGGGG CCCGCCCAGG
GTGGGAGCTG CTCAGAGAAA TGATACTGTA TGTGGAGGGG TGCGCTAGCC
AGC
MAST_B278_ CTGCTAGGCA GTTTTCAAAG CATTTTTGAC TATATTGGGG CCCGCCCAGG
GTGGGAGCTG CTCAAAGAAA TGATACTGTA TGTGGTGGGG TGCACTAGCC
AGC
SAV1_SE210 CTGCTAGGCA GTTTTCAAAG CATCTTTGAG TATATTGGGG CCCGCCCAGG
GTGGGAGCTG CTCAGAGAAA TGATACTGTA TGTGGAGGGG TGCGCTGGCC
AGC

1 1 1 1 1

5 104

```
ASIAN2_Ema   GGGATGGAAG ATTCTCCAAG GACYGGGGAA GAGGACAAAC TGGAGAACAG
             TGGTGGTTGT TAGCTGTTCT GGAGTCGCTC TGACTCAGGG CAACCCCATG
             CACA
FOR_DS1535   GGGATGGAAG ATTCTCCAAG GACTGGGGAA GAGGACAAAC TGGAGAACAG
             TGGTGGTTGT TAGCTGTTCT GGAGTCGCTC TGACTCAGGG CAACCCCATG
             CACA
MAM1_SP134   GGGATGGAAG ATTCTCCAAG GACTGGGGAA GAGGACAAAC TGGAGAACAG
             TGGTGGTTGT TAGCTGTTCT GGAGTCACTC TGACTCAGGG CAACCCCATG
             CACA
MAST_B279_   GGGATGGAAG ATTCTCCAAG GACTGGG-AA GAGGACAAAC TGGAGAACAG
             TGGCGGTTTT TAGCTGTTCT GGAGTCGCTC TGACTCAGGG CAACCCCATG
             CACA
SAV1_SE210   GGGATGGAAG ATTCTCCAAG GACTGGGGAA GAGGACAAAC TGGAGAACAG
             TGGTGGTTGT TAGCTGTTCT GGAGTCGCTC TGACTCAGGG CAACCCCATG
             CACA
```

 1 1 1 1 1
5 104

```
ASIAN2_Ema   GTGAGGTGCC TCTGGGCACT CATCAGAAGA GCAAAAAGAG CTTTGTAACA
             CTTGCCAGAG CAGGGCAGAG GCCACAGGCC AGAGAGAGGC ATACTTGTGA
             GCAC
FOR_DS1535   GTGAGGTGCC TCTGGGCACT CATCAGAAGA GCAAAAAGAG CTTTGTAACA
             CTTGCCAGAG CAGGGCAGAG GCCACAGGCC AGAGAGAGGC ATACTTGTGA
             GCAC
MAM1_SP134   GTGAGGTGCC TCTGGGCACT CATCAGAAGA GCAAAAAGAG CTTTGTAACA
             CTTGCCAGAG CAGGGCAGAG GCCACAGGCC AGAGAGAGGC ATACTTGTGA
             GCAC
MAST_B281_   GTGAGGTGCC TCTGGGCACT CATCAGAAGA GCAAAA-GAG CTTGGTAACA
             CTTGCTAGAG CAGG-CAGAG GCCACAGGCC AGAGAGAGGC ATACTTGTGA
             GCAC
SAV1_SE210   GTGAGGTGCC TCTGGGCACT CATCAGAAGA GCAAAAAGAG CTTTGTAACA
             CTTGCCAGAG CAGGGCAGAG GCCACAGGCC AGAGAGAGGC ATACTTGTGA
             GCAC
```

 1 1 1 1 1
5 103

```
ASIAN2_Ema   TGTATTATCT CATTTAATCC TTT-CAGCAG CCCT-GCCAG TTGGCCCATA
             GCATTTACAG AGGCAACATT TGTGATTTTG ACTCTTTGAA AACAACTCCA
             AAG
FOR_DS1535   TGTATTATCT CATTTAATCC TTT-CAGCAG CCCT-GCCAG TTGGCCCATA
             GCATTTACAG AGGCAACATT TGTGATTTTG ACTCTTTGAA AACAACTCCA
             AAG
MAM1_SP134   TGTATTATCT CATTTAATCC TTT-CAGCAG CCCT-GCCAG YTGGCCCATA
             GCATTTACAG AGGCAACATT TGTGATTTTG ACTCTTTGAA AACAACTCCA
             AAG
MAST_B215_   TGTATTATCT CATTTAATTC TTTTCAGCAG CCCTCGCCAG TTGGCCCATA
             GCATTTACAG AGACAACATT TGTGATTTTG ACTCTT-GAA AACAACTCCA
             AAA
SAV1_SE210   TGTATTATCT CATTTAATCC TTT-CAGCAG CCCT-GCCAG TTGGTCCATA
             GCATTTACAG AGGCAACATT TGTGATTTTG ACTCTTTGAA AACAACTCCA
             AAG
```

 1 1 1 1 1
5 102

```
ASIAN2_Ema   GGCCCAATTG GTTCTCTGAG ATTCCGCAAG CCACTGTTGC TGCCTGGACC
             CTGAGACATG GGAAGAAATG GCCTAGGGCC CATGCTATAC TCTTGTTGAG
             GG
FOR_DS1535   GGCCCAATTG GTTCTCTGAG ATTCCGCAAG CCACTGTTGC TGCCTGGACC
             CTGAGACATG GGAAGAAATG GCCTAGGGCC CATGCTATAC TCTTGTTGAG
             GG
MAM1_SP134   GGCCCAATTG GTTCTCTGAG ATTCCGCAAG CCACTGTTGC TGCCTGGACC
             CTGAGACATG GGAAGAAATG GCCTAGGGCC CATGCTATAC TCTTGTTGAG
             GG
MAST_B283_   GGCCCAATTG GTTCTCTGAG ATTCTGTAAG CCACTGTTGC TGCCTGGACC
             CTGAGATATG GGAAGAAATG GCCTAGGGCC CATGCTATAC TCTTGTTGAA
             GA
```

SAV1_SE210    GGCCCAATTG GTTCTCTGAG ATTCCGCAAG CCACTGTTGC TGCCTGGACC
CTGAGACATG GGAAGAAATG GCCTAGGGCC CATGCTATAC TCTTGTTGAG
GG

1 1 1 1 1
5 102

ASIAN2_Ema    AAAGACGGAG ACACTGTGTT CCTGAGGCGA TTTACCTCTG ATCTCTGTCT
CTGTCACCTC TGGACTGGTG CTCAGGGCCT TGCTGAGGAG CTGAGGGGG-
CA
FOR_DS1535    AAAGACGGAG ACACTGTGTT CCTGAGGCGA TTTACCTCTG ATCTCTGTCT
CTGTCACCTC TGGACTGGTG CTCAGGGCCT TGCTGAGGAG CTGAGGGGG-
CA
MAM1_SP134    AAAGACGGAG ACACTGTGTT CCTGAGGCGA TTTACCTCTG ATCTCTGTCT
CTGTCACCTC TGGACTGGTG CTCAGGGCCT TGCTGAGGAG CTGAGGGGG-
CA
MAST_B284_    AAAGACGGAG ACACTGTGTT CCTGAGGCGA TTTACCTCTG ATCTCTGTCT
CTGTCACCTC TGGACTGGTG CTCAGGGCCT TGCTGAGGAG CTAAAGAAAA
CA
SAV1_SE210    AAAGACGGAG ACACTGTGTT CCTGAGGCGA TTTACCTCTG ATCTCTGTCT
CTGTCACCTC TGGACTGGTG CTCAGGGCCT TGCTGAGGAG CTGAGGGGG-
CA

1 1 1 1 1
5 104

ASIAN2_Ema    TTTTGATTGA TGTAAAATCC AGTGACCCAA GGGAGGAAGC CCTAATGTTA
GACTTCTCAC CAACTGTGGG TGGGATTTGG GACCAGATTT AAA-TAAATT
TGGA
FOR_DS1535    TTTTGATTGA TGTAAAATCC AGTGACCCAA GGGAGGAAGC CCTAATGTTA
GACTTCTCAC CAACTGTGGG TGGGATTTGG GACCAGATTT AAA-TAAATT
TGGA
MAM1_SP134    TTTTGATTGA TGTAAAATCC AGTGACCCAA GGGAGGAAGC CCTAATGTTA
GACTTCTCAC CAACTGTGGG TGGGATTTGG GACCAGATTT AAA-TAAATT
TGGA
MAST_B285_    TTTTGATTGA TGTAAA-TCC AGTGACCCAA AGGAGGAAGC CCTAATGTTA
GACTTCTCAC CAACTGTGGG TGGGATT-GG GACCAAATTT AAAATAAAGT
TTAA
SAV1_SE210    TTTTGATTGA TGTAAAATCC AGTGACCCAA GGGAGGAAGC CCTAATGTTA
GACTTCTCAC CAACTGTGGG TGGGATTTGG GACCAGATTT AAA-TAAATT
TGGA

1 1 1 1 1
5 102

ASIAN2_Ema    AATTATTAGA GCAAATTTGC TCATACATTT TGCTGAATAT TCAAAATAAT
TCATGTTCAC TCCCCCTCTT TAACATAAGA GCTTTGGGTA GGCTTTCCCT
TG
FOR_DS1535    AATTATTAGA GCAAATTTGC TCATACATTT TCCTGAATAT TCAAAATAAT
TCATGTTCAC TCCCCCTCTT TAACATAAGA GCTTTGGGTA GGCTTTCCCT
TG
MAM1_SP134    AATTATTAGA GCAAATTTGC TCATACATTT TGCTGAATAT TCAAAATAAT
TCATGTTCAC TCCCCCTCTT TAACATAAGA GCTTTGGGTA GGCTTTCCCT
TG
MAST_B286_    AATTATTAGA GCAAATTTGC TTATACATTT TGCTGAATAT TCAAAATAAT
TCATGTTCAC TCCCCCTCTT TAACATAAGA GCTTTGGGTA GGCTTTCCCT
TG
SAV1_SE210    AATTATTAGA GCAAATTTGC TCATACATTT TGCTGAATAT TCAAAATAAT
TCATGTTCAC TCCCCCTCTT TAACATAAGA GCTTTGGGTA GGCTTTCCCT
TG

1 1 1 1 1
5 102

ASIAN2_Ema    ACCAAGACAG ATCCTGAGCC TTCGAAGAGG CTGCCACACT GCCCCAGCGG
CCCCACACGC CAGGGGCTGG AAGTGGGCTC GCTTAGGGCA GGGACACCTC
TC
FOR_DS1535    ACCAAGACAG ATCCTGAGCC TTCGAAGAGG CTGCCACACT GCCCCAGCGG
CCCCACACGC CAGGGGCTGG AAGTGGGCTC GCTTAGGGCA GGGRCACCTC
TC
MAM1_SP134    ACCAAGACAG ATCCTGAGCC TTCGAAGAGG CTGCCACACT GCCCCAGCGG

```
             CCCCACACGC CAGGGGYTGG AAGTGGGCTC GCTTAGGGCA GGGACACCTC
             TC
MAST_B287_   ATTAAGATAG ATCCCGAGCC TTCGAAGAGG GTGCCACACT GCCCCAGCGG
             CCCCACACGC CAGGGGCTGG AAGTGGTCTC GCTTAGGGCA GGGACACCTC
             TC
SAV1_SE210   ACCAAGACAG ATCCTGAGCC TTCGAAGAGG CTGCCACACT GCCCCAGCGG
             CCCCACACGC CAGGGRCTGG AAGTGGGCTC GCTTAGGGCA GGGACACCTC
             TC

 1 1 1 1 1
5 102

ASIAN2_Ema   TCCTACACAG CTCAGTTCGC CTGACTGCAG CTTTCTGAGT TCACCTAGTG
             CTTCTCCCAG ACAAAATCAT GACTAAGCAG AGGCAAAATT CATGTTATGC
             TC
FOR_DS1535   TCCTACACAG CTCAGTTCGC CTGACTGCAG CTTTCTGAGT TCACCTAGTG
             CTTCTCCCAG ACAAAATCAT GACTAAGCAA AGGCAAAATT CATGTTATGC
             TC
MAM1_SP134   TCCTACACAG CTCAGTTCGC CTGACTGCAG CTTTCTGAGT TCACCTAGTG
             CTTCTCCCAG ACAAAATCAT GACTAAGCAA AGGCAAAATT CATGTTATGC
             TC
MAST_B288_   TTTTATACAG CTCAGGTCGC CTGACTGCAG CTTTCTGAGT TGACCTAGTG
             CTTCTGCCAG ACAAAATCAT GACTAAGCAA AGGCAAAATT CATGTTATGC
             TC
SAV1_SE210   TCCTACACAG CTCAGTTCGC CTGACTGCRG CTTTCTGAGT TCACCTAGTG
             CTTCTCCCAG ACAAAATCAT GACTAAGCAA AGGCAAAATT CATGTTATGC
             TC
```
