# Supplementary figures and images for: Genomic DNA Sequences from Mastodon and Woolly Mammoth Reveal Deep Speciation of Forest and Savanna Elephants
Source: PLoS Biol. 2010 Dec 21;8(12):e1000564. doi: 10.1371/journal.pbio.1000564 (PMC3006346; doi:10.1371/journal.pbio.1000564)

a)


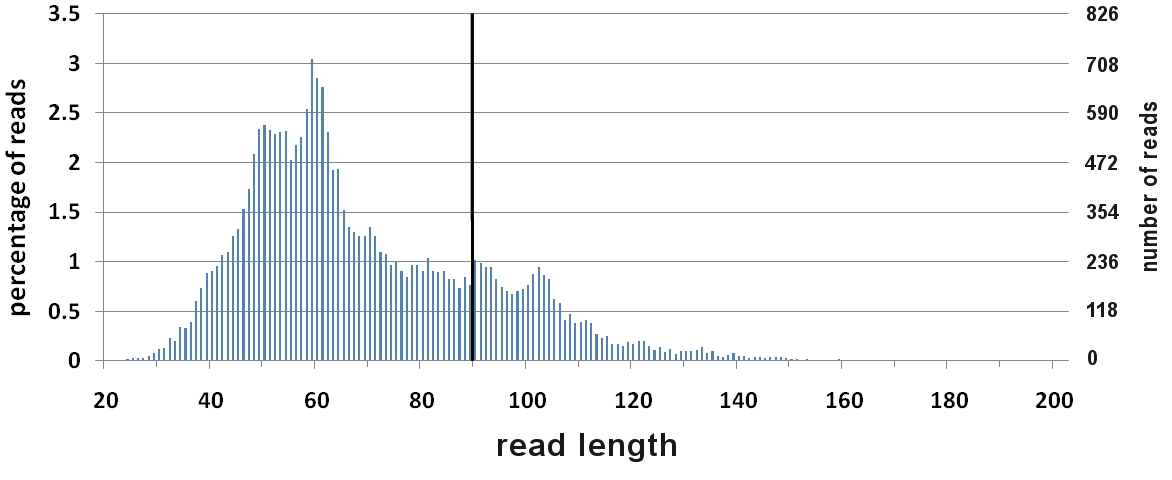


b)


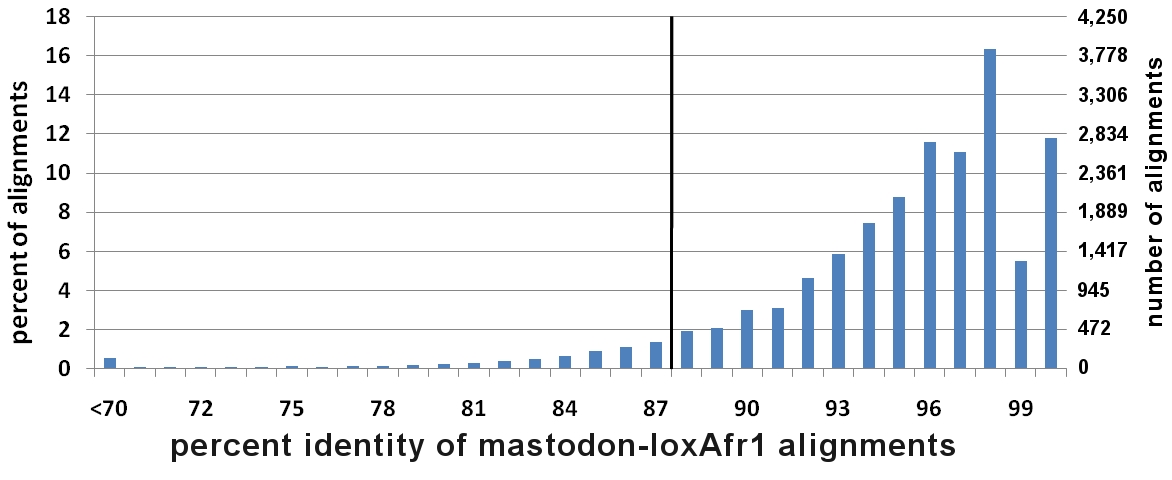

Supplement: Figure S1 — Mastodon shotgun results. (a) A histogram of read length (in nucleotides) of all putative mastodon sequences gathered in this study by shotgun sequencing. The longest sequence is 202 nucleotides long, and only the longer sequences (to the right of the black line) were used for primer design. (b) Percent identity of all mastodon-loxAfr1 alignments. The mean percent identity is 95%. Only sequences with an identity of more than 87% (to the right of the black line) were used for primer design. (0.21 MB DOC) [file pbio.1000564.s004.doc]

**
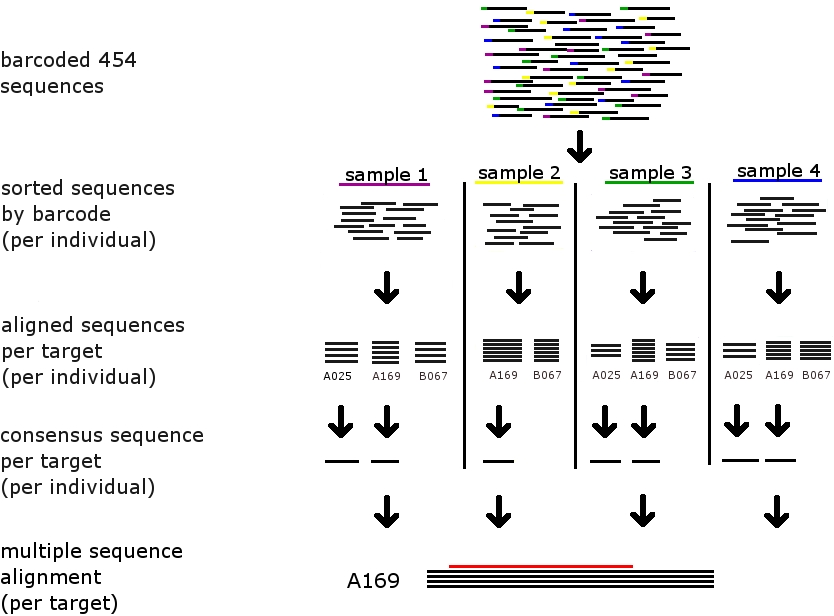
**

Supplement: Figure S2 — Analysis of 454-sequence data to build multiple alignments. Sequences were sorted according to their barcode to identify the sample, and then the sequences (now per individual) were further sorted by the 5′-primer and aligned to the reference (loxAfr1) using a similarity threshold of 80%. Consensus sequences were called per individual and consensus sequences of the various individuals were merged into multiple sequence alignments including the mastodon shotgun sequence (red). (0.14 MB DOC) [file pbio.1000564.s005.doc]

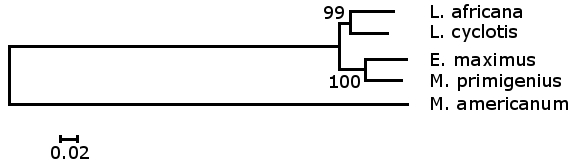

Supplement: Figure S3 — A Neighbor Joining tree built with the software MEGA4 supports the topology (((Savanna, Forest),(Asian, Mammoth)), Mastodon). (0.04 MB DOC) [file pbio.1000564.s006.doc]
