# Supplementary material for: Genomic DNA Sequences from Mastodon and Woolly Mammoth Reveal Deep Speciation of Forest and Savanna Elephants
Source: PLoS Biol. 2010 Dec 21;8(12):e1000564. doi: 10.1371/journal.pbio.1000564 (PMC3006346; doi:10.1371/journal.pbio.1000564)
Supplement: Table S1 — Samples used in this study. (0.04 MB DOC) [file pbio.1000564.s007.doc]

**Table S1: Samples used in this study**

| **ID** | **Scientific name** | **origin** | **Studbook#** | **remarks** |
| --- | --- | --- | --- | --- |
| DS1535 | *Loxodonta cyclotis* | Dzanga-Sangha NP, Central African Republic | Wild |  |
| loxAfr1 | *Loxodonta africana* | Kruger National Park, South Africa | 532 (“Swazi”) | Genome has been sequenced |
| SE2100 | *Loxodonta africana* | Serengeti National Park, Lobo, Tanzania | Wild |  |
| Ema-2 | *Elephas maximus* | National Zoological Park, Washington DC, USA | 165 (“Shanthi”) | Wild born, Sri Lanka |
| Ema-10 | *Elephas maximus* | Burnet Park Zoo, Syracuse NY, USA | 28 (“Siri”) | Wild born possibly Thailand |
| SP1349 | *Mammuthus primigenius* | Kolopatkaya River, Kolyma Lowland, Sakha Yakutia, Russia | n/a (ancient DNA) | Ref. 5 (Römpler et al. 2006) |
| SP1351 | *Mammut americanum* | central Arctic coastal plain of N. Alaska, USA | n/a (ancient DNA) | Ref. 1 (Rohland et al. 2007) |
