# Supplementary material for: Genomic DNA Sequences from Mastodon and Woolly Mammoth Reveal Deep Speciation of Forest and Savanna Elephants
Source: PLoS Biol. 2010 Dec 21;8(12):e1000564. doi: 10.1371/journal.pbio.1000564 (PMC3006346; doi:10.1371/journal.pbio.1000564)
Supplement: Table S2 — Summary of loci that we attempted to amplify. (0.03 MB DOC) [file pbio.1000564.s008.doc]

**Table S2: Summary of loci that we attempted to amplify**

| **experimental round** | **Target IDs** | **# of loci** | **# of loci with sequence information for all 5 taxa *** | **sequenced length in bp** | **isPCR threshold** |
| --- | --- | --- | --- | --- | --- |
| A.1 | A001-213 | 213 | 83 | 8,536 | none |
| A.2 | A214-458 | 245 | 138 | 13,689 | 26 |
| B | B001-288 | 288 | 154 | 17,538 | 2 |
|  | **Total** | **746** | **375** | **39,763** |  |

* Note: for some loci the savanna sequence information was only available from the genome (loxAfr1), resulting in only 347 loci for the MCMCcoal analysis.
