# Supplementary material for: Genomic DNA Sequences from Mastodon and Woolly Mammoth Reveal Deep Speciation of Forest and Savanna Elephants
Source: PLoS Biol. 2010 Dec 21;8(12):e1000564. doi: 10.1371/journal.pbio.1000564 (PMC3006346; doi:10.1371/journal.pbio.1000564)
Supplement: Table S3 — Target performance for different rounds of the experiment. (0.11 MB DOC) [file pbio.1000564.s009.doc]

**Table S3 a-d: Target performance for different rounds of the experiment**

**(a)** A.1 (first round, targets A001-213)

| species | **forest elephant** | **Asian elephant** | | | | | **savanna elephant** | | | **mammoth** | | |
| --- | --- | --- | --- | --- | --- | --- | --- | --- | --- | --- | --- | --- |
| sample | DS1535 | Ema-2 | | | Ema-10 | | SE2100 | | | SP1349 | | SP1349 |
| UDG treated mammoth extract |  |  |  | | |  |  | |  | + | | - |
| targets | A001-213 | A001-213 | | A001-213 | | | A001-213 | | | A001-213 | | A001-213 |
| # of targets | 213 | 213 | | 213 | | | 213 | | | 213 | | 53 |
| # of reads | 18,080 | 20,046 | | 22,348 | | | 14,801 | | | 20,572 | | 1,119 |
| # of alignable reads (>/= 80% identity) | 15,733 | 17,391 | | 19,620 | | | 13,007 | | | 16,753 | | 1,101 |
| # of unalignable reads (<80%identity) | 2,347 | 2,655 | | 2,728 | | | 1,794 | | | 3,819 | | 18 |
| # of targets w/ less than 3 alignable reads | 18 | 20 | | 14 | | | 25 | | | 19 | | 1 |
| # of targets w/ more than 3 alignable reads | 195 | 193 | | 199 | | | 188 | | | 194 | | 52 |
| **sequence coverage** |  |  | |  | | |  |  | |  |  |  |
| # of targets w/ 3-10 alignable reads | 5 | 5 | | 3 | | | 4 | | | 5 | | 8 |
| # of targets w/ 11-40 alignable reads | 27 | 23 | | 26 | | | 45 | | | 28 | | 43 |
| # of targets w/ 41-80 alignable reads | 97 | 91 | | 68 | | | 96 | | | 82 | | 1 |
| # of targets w/ > 80 alignable reads | 66 | 74 | | 102 | | | 43 | | | 79 | | 0 |
| **# of targets with usable consensus sequence** | **85** | **95** | | **92** | | | **89** | | | **91** | | **52** |

**(b)** A.2 (second round, targets A214-458 + some from A.1)

| species | **forest elephant** | | | **Asian elephant** | | **savanna elephant** | | | **mammoth** | | | | | |
| --- | --- | --- | --- | --- | --- | --- | --- | --- | --- | --- | --- | --- | --- | --- |
| sample | DS1535 | | | Ema-2 | | SE2100 | | | SP1349 | | | SP1349 | | |
| UDG treated mammoth extract |  |  | |  |  |  | |  | - | | | - | | |
| targets | A001-213 | A214-458 | | A001-213 | A214-458 | A001-213 | | A214-458 | A001-213 | | A214-458 | A001-213 | | A214-458 |
| # of targets | 43 | 245 | | 43 | 245 | 43 | | 245 | 43 | | 245 | 43 | | 245 |
| # of reads | 12,614 | | | 20,119 | | 15,840 | | | 12,950 | | | 16,083 | | |
| # of alignable reads (>/= 80% identity) | 10,221 | | | 15,761 | | 12,557 | | | 7,589 | | | 9,290 | | |
| # of unalignable reads (<80%identity) | 2,393 | | | 4,358 | | 3,283 | | | 5,361 | | | 6,793 | | |
| # of targets w/ less than 3 alignable reads | 35 | | | 31 | | 23 | | | 71 | | | 62 | | |
| # of targets w/ more than 3 alignable reads | 253 | | | 257 | | 265 | | | 217 | | | 226 | | |
| **sequence coverage** |  | |  |  |  |  |  | |  |  | |  |  | |
| # of targets w/ 3-10 alignable reads | 57 | | | 113 | | 52 | | | 68 | | | 59 | | |
| # of targets with 11-40 alignable reads | 120 | | | 137 | | 121 | | | 92 | | | 94 | | |
| # of targets w/ 41-80 alignable reads | 48 | | | 7 | | 52 | | | 39 | | | 42 | | |
| # of targets w/ > 80 alignable reads | 28 | | | 0 | | 40 | | | 18 | | | 31 | | |
| **# of targets with usable consensus sequence** | **25** | **138** | | **35** | **134** | **24** | | **123** | **21** | | **98** | **16** | | **91** |

**(c) B (third round, targets B001-288 + some from A.1 and A.2)**

| species | **forest elephant** | | **Asian elephant** | | **savanna elephant** | | **mammoth** | | |
| --- | --- | --- | --- | --- | --- | --- | --- | --- | --- |
| sample | DS1535 | | Ema-2 | | SE2100 | | SP1349 | | SP1349 |
| UDG treated mammoth extract |  |  |  |  |  |  | + | - | - |
| targets | A001-458 | B001-288 | A001-458 | B001-288 | A001-458 | B001-288 | A001-458 | B001-288 | B001-288 |
| # of targets | 42 | 288 | 22 | 288 | 56 | 288 | 96 | 288 | 288 |
| # of reads | 9,324 | | 7,316 | | 9,032 | | 16,279 | | 6,724 |
| # of alignable reads (>/= 80% identity) | 8,607 | | 6,690 | | 8,281 | | 13,890 | | 5,498 |
| # of unalignable reads (<80%identity) | 717 | | 626 | | 751 | | 2,389 | | 1,226 |
| # of targets w/ less than 3 alignable reads | 19 | | 18 | | 14 | | 69 | | 41 |
| # of targets w/ more than 3 alignable reads | 311 | | 292 | | 330 | | 315 | | 247 |
| **sequence coverage** |  |  |  |  |  |  |  |  |  |
| # of targets w/ 3-10 alignable reads | 52 | | 59 | | 56 | | 38 | | 52 |
| # of targets with 11-40 alignable reads | 198 | | 209 | | 236 | | 173 | | 174 |
| # of targets w/ 41-80 alignable reads | 57 | | 23 | | 31 | | 68 | | 19 |
| # of targets w/ > 80 alignable reads | 4 | | 1 | | 7 | | 36 | | 2 |
| **# of targets with usable consensus sequence** | **26** | **137** | **14** | **162** | **37** | **165** | **55** | **172** | **171** |

**(d) A&B (fourth round, some targets from A.1, A.2 and B).**

| species | **forest elephant** | | | **Asian elephant** | | | **savanna elephant** | | | **mammoth** | | |
| --- | --- | --- | --- | --- | --- | --- | --- | --- | --- | --- | --- | --- |
| sample | DS1535 | | | Ema-2 | | | SE2100 | | | SP1349 | | |
| UDG treated mammoth extract |  | |  |  | |  |  | |  | + | | + |
| targets | A001-458 | | B001-288 | A001-458 | | B001-288 | A001-458 | | B001-288 | A001-458 | | B001-288 |
| # of targets | 19 | | 128 | 10 | | 104 | 10 | | 89 | 56 | | 172 |
| # of reads | 9,475 | | | 7,262 | | | 7,974 | | | 14,738 | | |
| # of alignable reads (>/= 80% identity) | 5,830 | | | 4,384 | | | 4,993 | | | 8,811 | | |
| # of unalignable reads (<80%identity) | 3,645 | | | 2,878 | | | 2,981 | | | 5,927 | | |
| # of targets w/ less than 3 alignable reads | 9 | | | 9 | | | 3 | | | 28 | | |
| # of targets w/ more than 3 alignable reads | 138 | | | 105 | | | 96 | | | 200 | | |
| **sequence coverage** |  |  | |  |  | |  |  | |  |  | |
| # of targets w/ 3-10 alignable reads | 27 | | | 17 | | | 6 | | | 50 | | |
| # of targets with 11-40 alignable reads | 72 | | | 55 | | | 42 | | | 83 | | |
| # of targets w/ 41-80 alignable reads | 23 | | | 20 | | | 32 | | | 44 | | |
| # of targets w/ > 80 alignable reads | 16 | | | 13 | | | 16 | | | 23 | | |
| **# of targets with usable consensus sequence** | **13** | | **75** | **8** | | **54** | **6** | | **59** | **41** | | **103** |

**Notes:** The number and names of targets that were used in the respective round of the experiment are shown. Beside the number of passed filter 454 reads (# of reads) per individual, the number of reads that could be aligned to the savanna elephant genome sequence within 80% similarity and the number of sequences that could not be aligned within this cut-off are given. Sequence coverage was calculated per target and the numbers of targets per individual with the respective coverage are shown. Targets with less than 3 alignable reads were not analyzed further. The numbers of usable consensus sequences per individual are given in the last row.
