# Supplementary material for: Genomic DNA Sequences from Mastodon and Woolly Mammoth Reveal Deep Speciation of Forest and Savanna Elephants
Source: PLoS Biol. 2010 Dec 21;8(12):e1000564. doi: 10.1371/journal.pbio.1000564 (PMC3006346; doi:10.1371/journal.pbio.1000564)
Supplement: Text S1 — Data collection. (0.07 MB DOC) [file pbio.1000564.s010.doc]

**Text S1: Data Collection**

**Mastodon shotgun sequencing**

We prepared two 454 libraries based on 10 µl (library A) and 7.5 µl (library B) of a previously generated mastodon genomic DNA extract (the same that we previously analyzed in ref. [1] in order to sequence mtDNA). The libraries were prepared in a laboratory dedicated to ancient DNA work and sequenced on a Roche/454 Genome Sequencer (GS) (Figure 1a - main text). For library A, one complete picotiter plate—that is, 16 lanes—was sequenced on the GS20 model of the sequencer. For library B, 19 lanes were sequenced on a 454 GS FLX, distributed over several sequencing runs. Sequences were compared to the African savanna genome (loxAfr1, http://www.broadinstitute.org/ftp/pub/assemblies/mammals/elephant/loxAfr1/), the human genome (hg18), the mouse genome (mm8), the environmental sequence database (env) and all nucleotide sequences (nr) available in GenBank. Sequences with a best hit to the elephant genome were used to build semiglobal alignments of loxAfr1 and the putative mastodon sequences (Figure 1b - main text).

Approximately 519,000 reads were generated (NCBI short read archive SRA010805; library A: SRX015822, library B: SRX015823), resulting in 45 Mb of DNA sequences. Of these, 1.76 Mb (about 24,000 sequences) had their best match to loxAfr1. The average read length of putative mastodon sequences was 69 nucleotides and the average identity of mastodon-loxAfr1 alignments was 95%. The read length distribution of the mastodon sequences is shown in Figure S1a and the percent identity of mastodon-loxAfr1 alignments in Figure S1b.

**Primer design for PCR amplification of targeted loci**

Alignments that showed >87% identity and where the underlying mastodon sequence was 90 nucleotides long or longer were used for primer design (Figure 1b - main text). Primers were designed using loxAfr1 savanna elephant sequences flanking the mastodon sequences, in order to obtain homologous sequence information for mastodon, mammoth and the modern elephants and to capture the maximum amount of sequence overlapping the mastodon read. Potential targets with known repeat elements were removed using the program RepeatMasker (<http://www.repeatmasker.org/>). Primer design utilized the program *Primer3* (<http://primer3.sourceforge.net/>) using default settings, but restricting primer length to 25 nucleotides or shorter, with melting temperature differences between all primers limited to 3 degrees Celsius. This approach resulted in 574 primer pairs and targets (A001-574) for the first mastodon library, with products ranging in length between 133 and 206 bp (including primers; mean 168 bp). For the second mastodon library 1,151 potential primer pairs (B0001-1151) could be generated with product sizes between 150 and 309 bp (mean 215bp) in length.

In the analysis of the initial set of 213 targets (round A.1, primer A001-213) more than half (112) of the targets were found to produce very different sequences for all 4 species, such as sequences below 80% similarity, and/or a mixture of very distinct sequence variants within the 80% similarity cutoff (see below), and/or too many polymorphic positions within the 80% cutoff, resulting in inconclusive consensus sequences. These sequences with low matching rates may reflect primers annealing at the wrong site or at too many loci in the genome. Targets that failed to produce usable data proved in many cases to be repetitive elements, which are known to be especially common in elephantids [2,3], and may not have been filtered out by the RepeatMasker software because it does not incorporate elephantid-specific repeat families.

Based on these observations from the first round of data generation, we modified the bioinformatic protocol used to choose primer pairs for subsequent rounds of PCR. Specifically, the remaining 361 primer pairs (A214-574) from the first mastodon library were filtered by using In-Silico PCR (isPCR, <http://genome.ucsc.edu/cgi-bin/hgPcr>) against the savanna elephant genome (loxAfr1) to eliminate primer pairs that had a high probability of annealing to many different locations in the elephant genome. Each primer was required to align to the loxAfr1 genome no more than 26 times (since it was empirically observed that for all targets that aligned more than 26 times, it was rare for useful sequence information to be retrieved in the first round). There were 245 primer pairs (out of 361) that met these criteria. These primer pairs were used in the second round (A.2) of the project.

From the 1,151 potential primer pairs of the second mastodon library, 288 targets were chosen with the longest underlying mastodon sequences and isPCR results of between 0 and 2 matches, to further reduce the possibility of amplifying many repetitive sites. The products of these targets ranged between 150 and 299 bp in length (including primers, average 225 bp) and were used in the third round of the project (round B).

**PCR Amplification of targeted loci**

A two-step multiplex approach [4] was applied to one mammoth sample (the same sample from which a complete sequence of a nuclear gene had been retrieved [5]), one African savanna elephant, one African forest elephant and one Asian elephant (a second Asian elephant [Ema-10] was only used in round A.1; for sample information see Table S1). The mammoth multiplex amplifications were all carried out in a laboratory dedicated to ancient DNA work (MPI-EVA, Leipzig) or in a laboratory where no elephant DNA had been handled before (Harvard Medical School, Boston). Due to possible damage in ancient DNA [6,7] and low copy numbers of nuclear DNA, the mammoth extract was treated with Uracil-DNA Glycosylase (UDG) before multiplex amplification [8] or the locus was replicated by a second multiplex PCR and sequencing of the subsequent simplex PCR.

Over the course of the project, we amplified elephantid DNA using 746 primer pairs (round A.1: 213, round A.2: 245 [+ redos of A001-213], round B: 288 [+ redos of A001-458]. All primer sequences are provided in Supplementary Online File 1. To increase the efficiency of the experiment, multiplex PCRs were run. Primers were combined into several primer pools with between 22 and 87 primer pairs per pool. These primer pools were used to amplify between 22 and 87 targets in parallel in a multiplex reaction (first step). Primer pairs for which few or no sequences could be obtained during one round of sequencing were used to construct the primer pools of the following round [some A001-213 in A.2 and some A001-458 in B]. The second step of the 2-step multiplex approach used the diluted multiplex product as template for several simplex PCR reactions with just one pair of primers in each (Figure 1c - main text).

Altogether four rounds (A.1, A.2, B, A&B) of multiplex PCR were performed followed by simplex PCR. In order to retrieve as much overlapping data as possible, the fourth round solely consisted of previously amplified targets that had failed or had given missing data for some of the Proboscideans in previous rounds.

Negative controls were included during all rounds of experiments. In total 39 negative multiplex reactions (using water instead of DNA) were performed and 1,426 subsequent simplex PCR reactions were performed using the respective diluted multiplex reaction (90% of all loci were targeted at least once). Negative controls that amplified a product (visualized on agarose gels) were either cloned for Sanger sequencing of at least 3 clones on an ABI 3730, or bar-coded in normalized product pools (see below) and sequenced alongside with the samples on a 454 GS.

Of the 1,426 simplex PCR controls, 18 showed a product of the expected length. Sequences were compared to the non-redundant nucleotide collection (nr) from Genbank using Blastn. For three negative controls, the best matches were to human entries in the database or to cow. We hypothesize that these three contaminants were most likely due to the experimental setup (cow from BSA as a PCR ingredient). For eight products, no significant match could be found in the database; these sequences most likely did not represent elephant contamination, as there was no match to the loxAfr1 genome.

We found elephantid contamination in five products where the best match was to savanna elephant. These were all targets where we did not obtain conclusive consensus sequences for all 4 taxa because primers matched several loci in the genome, and they were not used for further analysis. We do not know how these contaminants could have passed through the laboratory process. However, the very low rate of implied contamination of 0.4% [5/1426] suggests that contamination is expected to have a negligible effect on our inferences.

**Roche/454 sequencing of targeted loci**

As different primer pairs have different efficiencies resulting in different quantities of product, (simplex) PCR products were quantified using SYBR-, Pico- or Eva-Green assays and pooled per individual in equal ratios to achieve even coverage. Roche/454 libraries were prepared using the standard protocol or by attaching a short barcode to the PCR product pool in addition to the 454 adaptor [9,10]. The barcoding was used when small PCR pools of more than one sample were merged for sequencing in the same lane of a 454 run to take advantage of the sequencing capacity. Sequencing was done on a 454 GS20 or on a 454 GS FLX.

**Sequence analysis, consensus calling and multiple sequence alignments**

Sequence analysis was carried out per individual per target, and consensus sequences were called according to Stiller et al. 2009 [10]. The consensus sequences were merged into multiple sequence alignments and divergent sites were extracted only when consensus sequences for all species could be gathered (Figure S2). In detail, trimmed sequences (after removing the 454 adaptor sequences) were sorted by the 5’ primer for each individual independently. After sorting, alignments were built of all sequences matching the reference (loxAfr1), with a similarity threshold of ≥80%, well under the average sequence similarity between mammoth and African elephant of >98% [11]. Sequences with similarities below 80% were not analyzed further. Targets were further analyzed only if a minimum of 3 reliable sequences (representing 3 cloned sequences) were present per target per individual. Heterozygous nucleotides were reported only if each of the two alternative alleles was detected in at least 3 sequences.

A problem that arises when analyzing data from deep sequencing coverage of next generation sequencing data is the appearance of low copy count sequences with mismatches to the consensus sequence. These sequences may result from PCR errors in later rounds of the PCR and/or jumping artifacts during PCR, which would not have been detected by Sanger sequencing. Therefore, sequences that appeared in fewer than 5% of the sequences for the respective targets were removed from the data set. If they appeared more frequently, then the entire target was not used for that particular individual.

Consensus sequences per target per individual were generated using a Perl script that identified bases as ambiguous when less than 80% of the sequences in the alignment agree at the respective position (for further criteria see [10]). Multiple sequence alignments were created if consensus sequence information was available from all taxa (mastodon, mammoth, Asian, savanna and forest elephant). Mammoth sequences were only used when a consensus sequence of targets could be called if either the extract was treated with UDG before multiplex PCR or the target was replicated by a second PCR. We note that about 1x coverage of the mammoth genome has recently been generated by another group [12], and while this is a remarkable resource, we were not able to integrate it into our analysis since at the loci of interest, as there was not enough coverage to distinguish between true SNPs and sequencing errors or ancient DNA degradation (the same problem affects our mastodon data, but is less important for our analysis since we use the mastodon only as an outgroup for identifying the ancestral state at divergent sites, and even a substantial rate of sequencing error does not compromise this type of analysis). When no consensus sequence for the savanna elephant could be called from our sequence data, we only used the sequence from the savanna elephant genome (loxAfr1).

In later rounds of sequencing, several individuals were run on the same lane of a picotiter plate of the 454 sequencer. Individual molecules were identified as originating from a certain individual by using a barcode to differentiate among individuals. The sequences were sorted according to their barcode using the software *untag* (<http://bioinf.eva.mpg.de/pts/>) before sorting according to the 5’ primer and aligning to the reference sequence (see above).

The performance of each the 746 loci through the four experimental rounds together with the sequence coverage is reported in Table S3. Information about loci where we had overlapping data from all five taxa is provided in Table S2. Sequences were deposited in GenBank and have Accession numbers GU557155-558698.
