# Supplementary material for: Genomic DNA Sequences from Mastodon and Woolly Mammoth Reveal Deep Speciation of Forest and Savanna Elephants
Source: PLoS Biol. 2010 Dec 21;8(12):e1000564. doi: 10.1371/journal.pbio.1000564 (PMC3006346; doi:10.1371/journal.pbio.1000564)
Supplement: Text S2 — Error Rate Assessment. (0.04 MB DOC) [file pbio.1000564.s011.doc]

**Text S2: Error Rate Assessment**

**Understanding the effect of sequencing error on the mastodon data**

To determine the error rate in the mastodon shotgun sequence data, we attempted to resequence 50 loci from the first round of PCR amplification (A.1) from the same mastodon DNA extract from which we prepared the shotgun libraries. Since the primer pairs to resequence all the elephantids were designed based on the savanna elephant genome using sequence that was outside the mastodon shotgun sequence, there was a substantial chance that substitutions in the mastodon sequence since divergence from the elephantids would render the PCR primers ineffective. To increase the likelihood of amplifying the same loci from the mastodon extract, the targets were split into two overlapping fragments, in that the existing forward primer (designed solely based on loxAfr1) was combined with a newly designed degenerate reverse primer (e.g. locus A001-α), with its sequence based on all available sequence information (mastodon, modern elephants and mammoth). The already designed reverse primer (based on loxAfr1) was combined with a newly designed forward primer (e.g. locus A001-β), which was also based on all available sequence information.

Fifty loci were randomly chosen from the primer set A.1 and were split into two sets (α and β) consisting of attempts to amplify both halves of each locus. The target fragment lengths varied between 76 and 140 bp (including primers; average 111 bp). All primers were reordered for this experiment to exclude possible cross-contamination from previous experiments on elephantid DNA. The multiplex step was set up in a laboratory where no elephant DNA had been previously handled. The multiplex PCR approach was applied to the mastodon DNA extract for the two fragments covering each target locus (α and β), and we repeated all experiments in duplicate. One negative control each was carried through the entire experimental process. Normalized PCR products from each duplicate experiment were pooled, barcoded and sequenced together with other PCR products on a sixteenth of a picotiter plate on a Roche/454 GS FLX. Sequence analysis was conducted as described for the modern elephantids and mammoth using a similarity threshold for the alignments of 80%.

For 32 of the 100 targeted amplicons, we successfully retrieved consensus sequence for both independent amplifications, resulting in 1,726 bases that overlapped with the mastodon shotgun sequences (GenBank Accession numbers GU558739-558766). Comparison of the 454 shotgun sequences and the PCR derived consensus sequences of the mastodon showed 1,689 bases to be identical (97.9%), while 37 were discrepant.

Twenty-six of the 37 differences were C/G-to-T/A misincorporations, the most prominent miscoding lesion in ancient DNA [6,7]. Two differences appeared to be due to insertion/deletion errors in homopolymer stretches, a well-known error that arises in 454 sequencing [13]. The remaining 9 differences were concentrated in just two amplicons, and they were not consistent with miscoding lesions in the ancient molecule or homopolymer errors. We hypothesize that these differences reflect mismapping of the mastodon shotgun sequence data to the savanna reference genome loxAfr1 for these reads. As evidence for this, we note that the PCR derived mastodon sequences were much more similar to the consensus sequences of the elephants than they were to the shotgun mastodon sequence. The similarity of the mastodon shotgun sequence and the savanna elephant sequence of these two targets was at the lower boundary of the >87% similarity threshold (88% and 90% similarity, respectively).

Based on these observations, we were able to obtain a very approximate correction factor for elephant-mastodon divergence, taking into account the fact that a substantial proportion of the elephantid-mastodon divergent sites in our data set are false-positives due to ancient DNA damage or mismapping, and that we can roughly estimate their rate by this validation data. Table S2.1 shows that at all African-Eurasian divergent sites (sites that are polymorphic among the Elephantidae), the mastodon base from shotgun sequencing is correct. However, only 38% of divergent sites between mastodon and the Elephantidae validate, indicating that we need to correct estimates of mastodon-elephantid divergence in our data by this large factor.

**Table S2.1: Proportion of divergent sites where the mastodon base validates**

|  | **Before validation** | **After validation** | **% validated** |
| --- | --- | --- | --- |
| African-Eurasian differences | 12.8 | 12.8 | 100% |
| Mastodon-Elephantid differences | 51.4 | 19.4 | 38% |

Note: The numbers are not integers since results are averaged over all elephantid genotypes at each nucleotide. (At sites of polymorphism among the elephantids, the contribution to the count may not be an integer.)

**Very low rate of sequencing error in our multiplex PCR following by 454 sequencing**

We estimated the sequencing error rate in the 454 multiplex PCR data by sequencing a subset of the same loci using Sanger technology (ABI 3730) and longer amplicons with non-overlapping primer pairs. We chose a subset of 110 of the 213 target loci from round A.1 for this experiment, and designed primer pairs based on the loxAfr1 sequenced using *Primer3* with an average fragment length of 744 base pairs and the mastodon sequence contained within the fragment. The products were sequenced on an ABI3730 capillary sequencer at the Broad Institute using the re-sequencing production pipeline.

After direct sequencing of these 110 products, we found that many did not give clean sequence data, as indicated by low Phred quality scores. Only 40 of the 110 loci gave sequences with high quality scores in some of the elephantids, indicating the same problem we found for the shorter target size, i.e. that the primer pairs bound to multiple loci in the elephant genome. Of these 40 loci, only 15 overlapped with the multiple sequence alignments where we had gathered sequence information for all 5 taxa using 454 technology; thus, reliable sequence information was available for both sequencing techniques for this sample (GenBank Accession numbers GU558699-558738). These 15 loci added up to 1.8 kb of comparable sequence information, for which no differences between the two sequencing technologies could be detected. These results suggest that our sequencing results from the elephantid taxa have a very low rate of error and that preferential amplification of one allele is unlikely to be substantially biasing our results.
