# Supplementary material for: Genomic DNA Sequences from Mastodon and Woolly Mammoth Reveal Deep Speciation of Forest and Savanna Elephants
Source: PLoS Biol. 2010 Dec 21;8(12):e1000564. doi: 10.1371/journal.pbio.1000564 (PMC3006346; doi:10.1371/journal.pbio.1000564)
Supplement: Text S3 — Genomic distribution of loci. (0.03 MB DOC) [file pbio.1000564.s012.doc]

**Text S3: Genomic distribution of loci**

MCMCcoal assumes free recombination between loci. To verify this, we took the savanna sequence from each locus (347) and aligned it to the publicly available LoxAfr3 sequence (http://genome.ucsc.edu). This unfinished and unannotated genome consists of 2,353 scaffolds that are highly variable in length ranging from 5 kb to 130 Mb. The loci that reliably mapped (e-value < 10^-4^) hit 100 scaffolds, 62 of which were matched only once. Of the remaining 38 scaffolds, the minimum separation of loci on the scaffold is shown in Figure S4.1. All loci but one pair are separated by over 100 kb. The closest pair of loci was separated by 9.9 kb which is sufficiently close that this locus pair may be linked. We believe that the potential linkage disequilibrium between these two loci will not result in a substantial inflation of our variance estimates over a data set of 347 loci, and hence we do not believe it was necessary to remove one of these loci.

**
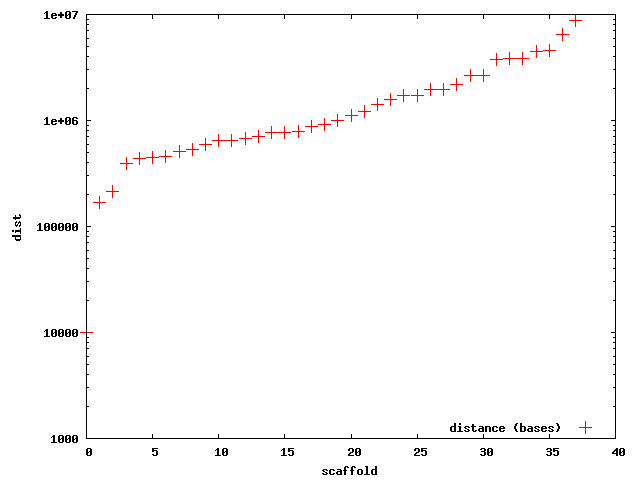
**

**Figure S4.1:** Minimum separation of loci pairs which map to the same loxAfr3 scaffold.
