# Supplementary material for: Genomic DNA Sequences from Mastodon and Woolly Mammoth Reveal Deep Speciation of Forest and Savanna Elephants
Source: PLoS Biol. 2010 Dec 21;8(12):e1000564. doi: 10.1371/journal.pbio.1000564 (PMC3006346; doi:10.1371/journal.pbio.1000564)
Supplement: Text S4 — MCMCcoal analysis to infer population parameters. (0.11 MB DOC) [file pbio.1000564.s013.doc]

**Text S4: MCMCcoal analysis to infer population parameters**

**The model that we fit to the genetic data**

We began by running MCMCcoal on an alignment of 5 sequences, one from each taxon, and tested the phylogeny (((Forest, Savanna), (Asian, Mammoth)) Mastodon), for all the loci for which we had diploid data from all elephantid taxa (n=347; see below). Thereafter, each diploid data set from each elephantid taxon (except for Mastodon, for which we have haploid data) was split into two sequences, producing a 9-way alignment. The splitting into two sequences was done by randomly assigning alleles at sites with ambiguity codes. The phylogeny was: ((((Forest_1_,Forest_2_),(Savanna_1_,Savanna_2_)),((Asian_1_,Asian_2_),(Mammoth_1_,Mammoth_2_)))Mastodon).

As described below, we found that the parameter estimates from the 5-way and 9-way analyses were consistent for the 5 parameters that overlapped between the two runs, and hence we report results from the 9-way analysis in the main text (the 9-way analysis is useful in inferring population sizes after savanna-forest and Asian-mammoth divergence).

**Table S3.1: Selection of prior distributions for demographic parameters**

| **Demographic Parameter** | **5% and 95% bounds of prior interval** | **Reason for choosing this prior distribution** |
| --- | --- | --- |
| *θ*_F_=*θ*_S_=*θ*_A_=*θ*_M_=*θ*_AM_=*θ*_FS_=*θ*_LE_=  *θ*_Elephantid-Mastodon_ | Confidence interval: 0.00057-0.00222 | Based on the range from savanna to forest elephant diversity in present-day populations. |
| *T*_F_=*T*_S_=*T*_A_=*T*_M_ | Confidence interval: 0-0 | Force zero time of split. |
| *T*_Elephantid-Mastodon_ | 24-30 million years | Fossil record of mastodon-elephantid divergence |
| *T*_Lox-Eur_ | 4.2 - 9 million years | Elephantid fossil dates |
| *T*_AM_ | 3.0 – 8.5 million years | Elephantid fossil dates |
| *T*_FS_ | 0.5 – 9 million years | Exploring the full range of uncertainty |

Note: *θ* and *T* are heterozygosity and split times respectively. Further justification for these distributions is provided in Text S5.

MCMCcoal is a Bayesian method, and thus requires prior distributions for each parameter reflecting what we believe are plausible values of the parameters based on the fossil record. Prior distributions for the demographic parameters are as shown in Table S3.1 and are further justified in Text S5. A model of random mutation rate variation among loci was assumed, where the mutation rate for each locus was separately estimated from the data. As described in the MCMCcoal documentation, the prior on the locus mutation rates is a transformed Dirichlet distribution, such that the variance of any locus rate is 1/α where α was set to 2.0 ([13]). We also carried out runs with a fixed rate model and obtained qualitatively similar results (not shown).

Since the mutation rate over the time scale of the analysis is unknown, a trial analysis was performed using a mutation rate *μ* of 0.5×10^-9^ mutations/site/year based on the observation that the mitochondrial mutation rate for proboscideans is approximately half of that of primates [1]. We also explored a range of other mutation rates corresponding to assumed average genetic divergence times for African and Eurasian elephants of *t*_Lox-Eur_ = 7.6-11 Mya (ranging from the mitochondrial DNA divergence estimated in ref. [1] to 2 My older than the maximum *T*_Lox-Eur_ consistent with the fossil record as argued in Text S5), using a generation time *g* of 31 years based on estimates of 17-20 years for females [14,15] and 40-49 years for males [16,17]. MCMCcoal also allows modeling of variable sequencing error rate across samples, and we titrated the error rate for mastodon so that the ratio of the posterior estimates of *t*_Lox-Eur_ and *t*_Elephantid-Mastodon_ is in the range consistent with the fossil record. Intuitively, we expected that assumptions about the error rate in the mastodon would have a minimal effect on the inference of the relative values of the demographic parameters relating elephantids, since the only way that the mastodon sequence affects these inferences is through its fidelity in assigning the ancestral allele (Text S2). Confirming this, we observed no change in these parameter estimates when we compared runs that did and did not model a mastodon sequencing error rate. However, our estimates of the parameters corresponding to elephantid-mastodon divergence (*T*_Elephantid-Mastodon_ and *N*_Elephantid-Mastodon_) were highly dependent on mutation rate assumptions.

To increase the speed of convergence of the Markov chain over iterations, we fine-tuned the acceptance proportion as recommended by the MCMCcoal documentation, which we found did not change our qualitative results (not shown).


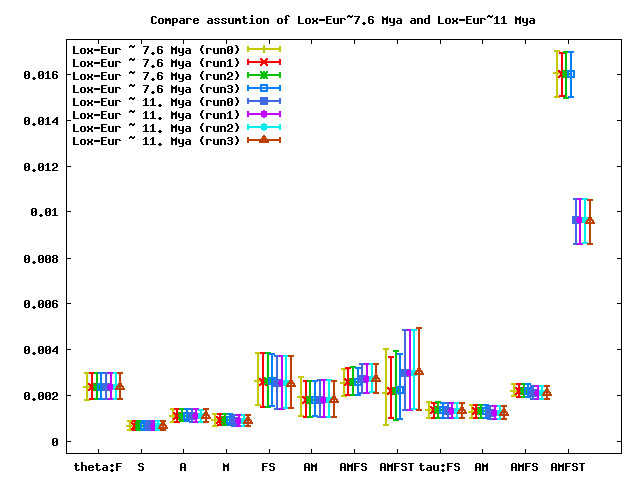


**Figure S3.1:** Consistency of demographic parameter estimates across 8 MCMCcoal runs. All parameters are given in coalescent units. To translate these to the units used in the paper, note that *θ* = 4*Nμg*, and *τ* = *Tμ*.

*τ*_Ele-Mas_

*τ*_Lox-Eur_

*τ*_AM_

*τ*_FS_

*θ*_Ele-Mas_

*θ*_Lox-Eur_

*θ*_AM_

*θ*_FS_

*θ*_M_

*θ*_A_

*θ*_S_

*θ*_F_

**Convergence properties of the Markov Chain Monte Carlo**

For each assumed African-Eurasian split time *T*_Lox-Eur_, the MCMC was run 4 times from different random seeds: one short run with 2,000 burn-in iterations and 10,000 follow-on iterations (run 0) and three subsequent runs with 4,000 burn-in iterations and 100,000 follow-on iterations (runs 1-3). A total of 347 loci were used, after eliminating 28 loci where PCR-based resequencing data was not available from all taxa (we eliminated 28 loci where savanna elephant was only represented by the haploid reference sequence loxAfr1, as we wished to use only diploid elephantid sequences in this analysis). We note that the input data to MCMCcoal are multiple sequence alignments (Supplementary Online File 3), and thus we did not need to remove non-biallelic sites as we did for the summary-statistic based analyses described in the text (multi-allelic sites are modeled as part of the method). Results for the runs were consistent (see Figure S3.1), and also consistent with 5-way runs (data not shown).

**
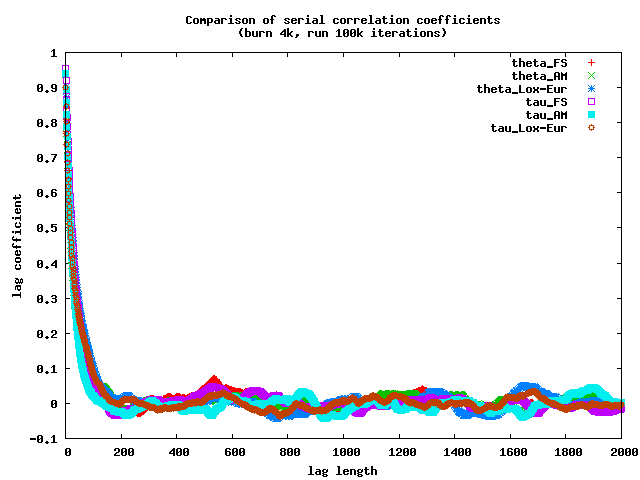
**

**Figure S3.2:** Serial lag coefficients for the *θ* and *τ* parameters for a typical run.

An important characteristic of the Markov Chain Monte Carlo (MCMC) is how uncorrelated the parameters are from iteration to iteration. We monitored the correlation, and found that serial correlation coefficients largely dropped to zero after 200 iterations (Figure S3.2), indicating that the runs of 100,000 iterations that we used for reporting our main results are adequate for establishing 90% credible intervals.

**Effect of the prior distribution on inferences**

The prior distributions on population split times (*T* or scaled coalescent unit τ = *Tμ*, [13]) was set based on information from the fossil record, as described in Table S3.1. It is more difficult to assign prior distributions for effective population sizes (*N* or scaled coalescent unit *θ* = 4*Nμg*, [13]), and we therefore more carefully explored the effect of our assumed prior distribution for *θ*.

Two sets of analyses were performed. In the first (set A), very broad priors for *θ* were used, where the 90% confidence interval ranged from 1/3 of savanna diversity to 3 times forest diversity. In the second (set B), this interval is set to range from savanna diversity (5^th^ percentile point) to forest diversity (95^th^ percentile point). Set B, which we used for the analyses reported in the main paper, still allows substantial variation outside this range if driven by the data. For each run, 4,000 burn-in iterations were performed with 100,000 follow-on iterations. Figure S3.3 shows that the results are consistent within set A and set B, indicating MCMC convergence.


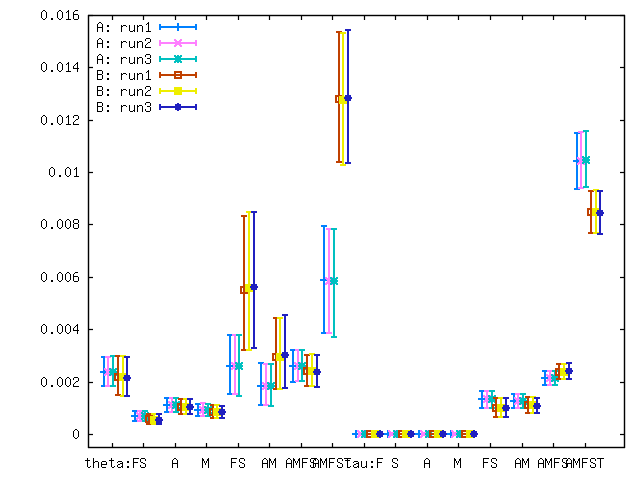
**Figure S3.3:** Dependence of parameter estimates on the prior distribution. Inferences are fairly robust to the prior assumptions except for those that correspond to elephantid-mastodon divergence, and the forest-savanna ancestral population size *θ*_FS_, which is poorly constrained by our data.

*τ*_Ele-Mas_

*τ*_Lox-Eur_

*τ*_AM_

*τ*_FS_

*τ*_M_

*τ*_A_

*τ*_S_

*τ*_F_

*θ*_Ele-Mas_

*θ*_Lox-Eur_

*θ*_AM_

*θ*_FS_

*θ*_M_

*θ*_A_

*θ*_S_

*θ*_F_

The greatest differences that we observe between set A and set B are on the branch leading to mastodon, as expected since there is little information available to differentiate between an ancient population divergence or a large ancestral effective population size. In addition, we observe poor constraint on our estimates of the population size ancestral to the forest-savanna split (*θ*_FS_ or *N*_FS_). There is little data constraining this parameter, and hence the width of uncertainty in the prior distribution is propagated to the posterior.

In Table S3.2, we present the full results of the MCMCcoal analysis shown in Table 3 of the main text, with the scaling all in coalescent units, and also reporting full details of the prior and posterior distributions.

**Table S3.2: Prior and posterior 90% confidence intervals** in a typical run.

| **Parameter** | **(α, β)** | **Prior (90% Interval)** | **Posterior (90% Interval)** |
| --- | --- | --- | --- |
| *θ*_F_ | (6.284, 4900) | 0.00128 (0.00057, 0.00222) | 0.00238 (0.00185, 0.00298) |
| *θ*_S_ | (6.284, 4900) | 0.00128 (0.00057, 0.00222) | 0.00068 (0.00050, 0.00089) |
| *θ*_A_ | (6.284, 4900) | 0.00128 (0.00057, 0.00222) | 0.00113 (0.00087, 0.00142) |
| *θ*_M_ | (6.284, 4900) | 0.00128 (0.00057, 0.00222) | 0.00093 (0.00070, 0.00119) |
| *θ*_AM_ | (6.284, 4900) | 0.00128 (0.00057, 0.00222) | 0.00181 (0.00107,0.00265) |
| *θ*_FS_ | (6.284, 4900) | 0.00128 (0.00057, 0.00222) | 0.00263 (0.00152,0.00384) |
| *θ*_Lox-Eur_ | (6.284, 4900) | 0.00128 (0.00057, 0.00222) | 0.00259 (0.00200, 0.00323) |
| *θ* _Elephantid-Mastodon_ | (6.284, 4900) | 0.00128 (0.00057, 0.00222) | 0.00220 (0.00092, 0.00392) |
| *τ*_F_=*τ*_S_=*τ*_A_=*τ*_M_ | (1, 200000000 ) | 0.0 (0.0, 0.0) | 0.0 (0.0, 0.0) |
| *τ*_Elephantid-Mastodon_ | (218.1, 16500) | 0.01322 (0.01178, 0.01472) | 0.01598 (0.01493, 0.01693) |
| *τ* _Lox-Eur_ | (19.07, 6060) | 0.00315 (0.00206, 0.00442) | 0.00220 (0.00193, 0.00248) |
| *τ*_AM_ | (10.45, 3900) | 0.00268 (0.00148, 0.00417) | 0.00131 (0.00104, 0.00161) |
| *τ*_FS_ | (1.677, 953) | 0.00176 (0.00025, 0.00442) | 0.00135 (0.00102, 0.00170) |

Note: The gamma priors used have the shape (α) and scale (β) parameters shown, and correspond to the resulting prior mean and 90% interval. *τ* and *θ* are measured as the expected number of mutations per site. The priors for the divergence time of the two forest elephant individuals (*τ*_F_), two savanna elephant individuals (*τ*_S_), two Asian elephant individuals (*τ*_A_) and two mammoths (*τ*_M_) are all forced to be 0 with 0 standard deviation, allowing MCMCcoal to run using two individuals from the same population. Quantities listed are for runs with a mutation rate based on assuming an African-Eurasian average genetic divergence of 7.6 million years. For runs using 11 million years for this date, β should be multiplied by 11/7.6 and correspondingly priors for *τ* change. However, posteriors are generally conserved across all runs since these are driven by the data (with the exception of the mastodon-elephantid divergence parameters which are highly affected by the uncertainty about the error rate in the mastodon – see Figure S3.1).
