# Supplementary material for: Genomic DNA Sequences from Mastodon and Woolly Mammoth Reveal Deep Speciation of Forest and Savanna Elephants
Source: PLoS Biol. 2010 Dec 21;8(12):e1000564. doi: 10.1371/journal.pbio.1000564 (PMC3006346; doi:10.1371/journal.pbio.1000564)
Supplement: Text S5 — Justification for prior distributions for MCMCcoal. (0.06 MB DOC) [file pbio.1000564.s014.doc]

**Text S5: Justification for prior distributions for MCMCcoal**

MCMCcoal is a Bayesian method, and thus requires us to provide prior distributions on the estimates of the population divergence times of each of the proboscidean taxa. Our choices are based on comparison to the fossil evidence, and are justified below. We note that our “Minimum” and “Maximum” in the document below refer to the 5^th^ percentile and 95^th^ percentile points of the prior distribution. These are soft bounds, and can accommodate the possibility of population split times outside the range if the data strongly imply such a scenario.

**Elephant-Mastodon: 24-30 Mya**

Minimum: 24 Mya, Sanders and colleagues [18] mention that the Rasmussen and Gutierrez (2009) report of fauna from the Eragaleit Beds of Lothidok includes the earliest definitively known mammutid, dated to between >24-27 Mya [19].

Maximum: 30 Mya, Sanders and colleagues [18] mention a specimen from Chilga, Ethiopia (28-27 Ma) that has some mammutid features superimposed on an otherwise palaeomastodont-type molar. To this and to other estimates of maximum dates we add 2 My (which is also a maximum average for proboscideans species duration in the fossil record [20]), to account for potential difficulty in recognizing differences during an initial period of divergence, and to account for inadequate fossil sampling.

**African-Eurasian: 4.2-9 Mya** (we use “Eurasian” to refer to the *Mammuthus-Elephas* clade)

Minimum: 4.2 Mya. Sanders and colleagues [18] list *Elephas ekorensis*, as “the most ancient unequivocal representative of the genus *Elephas*. Among locations listed for this species, the oldest is 5.0-4.2 Mya. (Note: this minimum assumes that *Loxodonta* is paraphyletic.)

Maximum: 9 Mya. The oldest *Loxodonta* fossil is 6-7 Mya [18,21]. We add 2 Mya to the maximum date for the reasons noted above.

**Asian-Mammoth: 3-8.5 Mya**

Minimum: 3 Mya. The earliest mammoth is *Mammuthus subplanifrons*, but Sanders and colleagues [18] state that “in the absence of associated crania, however, there is no certainty that this species is a mammoth.” The next to appear is *M. africanavus*, and Sanders and colleagues [18] state that this is “the earliest unambiguous evidence of the genus *Mammuthus* in Africa.” In the associated table [18], this species is listed as mid-late Pliocene, with dated locales at 3.5-3.0 Mya. (Note: this minimum assumes that *Elephas* is paraphyletic.)

Maximum: 8.5 Mya. For the questionable *M. subplanifrons*, the oldest date listed by Sanders and colleagues for any date range at a locale is 6 Mya [18]. There is an older “*Elephas nawataensis*” [22] that Sanders and colleagues argue may be mis-assigned to the genus *Elephas* [18]. The *E. nawataensis* dates to 4.2-6.5 Mya [22]. We add 2 Mya to the maximum date for the reasons noted above, yielding the 8.5 Mya maximum.

**Forest-Savanna: 0.5-9 Mya**

Minimum: 0.5 Mya. This is when *L. africana* appears in the fossil record, with modern savanna elephant morphology [18].

Maximum: 9 Mya. Assumes a trichotomy at the *Loxodonta*-Eurasian maximum.

**References for Supplementary Notes and Tables**

1. Rohland N, Malaspinas AS, Pollack JL, Slatkin M, Matheus P, et al. (2007) Proboscidean mitogenomics: chronology and mode of elephant evolution using mastodon as outgroup. PLoS Biol 5: e207.

2. Greenwood AD, Lee F, Capelli C, DeSalle R, Tikhonov A, et al. (2001) Evolution of endogenous retrovirus-like elements of the woolly mammoth (*Mammuthus primigenius*) and its relatives. Mol Biol Evol 18: 840-847.

3. Zhao F, Qi J, Schuster SC (2009) Tracking the past: interspersed repeats in an extinct Afrotherian mammal, Mammuthus primigenius. Genome Res 19: 1384-1392.

4. Roempler H, Dear PH, Krause J, Meyer M, Rohland N, et al. (2006) Multiplex amplification of ancient DNA. Nature Protocols 1: 720-728.

5. Rompler H, Rohland N, Lalueza-Fox C, Willerslev E, Kuznetsova T, et al. (2006) Nuclear gene indicates coat-color polymorphism in mammoths. Science 313: 62.

6. Hofreiter M, Jaenicke V, Serre D, Haeseler Av A, Paabo S (2001) DNA sequences from multiple amplifications reveal artifacts induced by cytosine deamination in ancient DNA. Nucleic Acids Res 29: 4793-4799.

7. Briggs AW, Stenzel U, Johnson PL, Green RE, Kelso J, et al. (2007) Patterns of damage in genomic DNA sequences from a Neandertal. Proc Natl Acad Sci U S A 104: 14616-14621.

8. Briggs AW, Stenzel U, Meyer M, Krause J, Kircher M, et al. (2009) Removal of deaminated cytosines and detection of in vivo methylation in ancient DNA. Nucleic Acids Res.

9. Meyer M, Stenzel U, Hofreiter M (2008) Parallel tagged sequencing on the 454 platform. Nat Protoc 3: 267-278.

10. Stiller M, Knapp M, Stenzel U, Hofreiter M, Meyer M (2009) Direct multiplex sequencing (DMPS)--a novel method for targeted high-throughput sequencing of ancient and highly degraded DNA. Genome Res 19: 1843-1848.

11. Poinar HN, Schwarz C, Qi J, Shapiro B, MacPhee RDE, et al. (2006) Metagenomics to paleogenomics: Large-scale sequencing of mammoth DNA. Science 311: 392-394.

12. Miller W, Drautz DI, Ratan A, Pusey B, Qi J, et al. (2008) Sequencing the nuclear genome of the extinct woolly mammoth. Nature 456: 387-390.

13. Burgess R, Yang Z (2008) Estimation of hominoid ancestral population sizes under bayesian coalescent models incorporating mutation rate variation and sequencing errors. Mol Biol Evol 25: 1979-1994.

14. Moss CJ (2001) The demography of an African elephant (*Loxodonta africana*) population in Amboseli, Kenya Journal of Zoology 255: 145-156.

15. Sukumar R (1989) The Asian Elephant: Ecology and Management. Cambridge: Cambridge University Press.

16. Hollister-Smith JA, Poole JH, Archie EA, Vance EA, Georgiadis NJ, et al. (2007) Age, musth and paternity success in wild male African elephants, Loxodonta africana. Animal Behaviour 74: 287-296.

17. Rasmussen HB, Okello JB, Wittemyer G, Siegismund HR, Arctander P, et al. (2008) Age- and tactile-related paternity success in male African elephants. Behavioral Ecology 19: 9-15.

18. Sanders WJ, Gheerbrant E, Harris JM, Saegusa H, Delmer C (2010) Proboscidea. In: Werdelin L, Sanders WJ, editors. Cenozoic Mammals of Africa. Berkeley: University of California Press.

19. Rasmussen DT, Gutierrez M (2009) A mammalian fauna from the Late Oligocene of northwestern Kenya. Palaeontographica Abteilung a-Palaozoologie-Stratigraphie 288: 1-52.

20. Maglio VJ (1973) Origin and evolution of the Elephantidae. Trans Am Phil Soc Philad, New Series 63: 1-149.

21. Vignaud P, Duringer P, Mackaye HT, Likius A, Blondel C, et al. (2002) Geology and palaeontology of the Upper Miocene Toros-Menalla hominid locality, Chad. Nature 418: 152-155.

22. Leakey MG, Harris JM (2003) Lothagam : the dawn of humanity in eastern Africa. New York: Columbia University Press. vi, 678 p. p.
